# XPO1-mediated TRIM21 nuclear export reprograms TREM2+ macrophage polarization by targeting IRF3 to augment anti-PD-1 efficacy in small cell lung cancer

**DOI:** 10.1038/s41467-026-76862-0

**Published:** 2026-08-22

**Authors:** Qingwu Du, Tingting Qin, Jingya Wang, Yantao Jiang, Qi Xu, Xueyang Li, Huiyan Liu, Junjie Yu, Yi Lu, Luyao Tong, Tao Sun, Peng Chen, Richeng Jiang, Dingzhi Huang

**Affiliations:** 1 Tianjin Medical University Cancer Institute and Hospital, National Clinical Research Center for Cancer; Key Laboratory of Cancer Prevention and Therapy, Tianjin; Tianjin’s Clinical Research Center for Cancer; Department of Thoracic Oncology, Tianjin Lung Cancer Center, Tianjin Cancer Institute & Hospital, Tianjin Medical University, Tianjin, PR China; 2 Department of Internal Medicine, The Affiliated Cancer Hospital of Zhengzhou University & Henan Cancer Hospital, Zhengzhou, People’s Republic of China; Institute of Cancer Research, Henan Academy of Innovations in Medical Science, Zhengzhou, China; 3 State Key Laboratory of Medicinal Chemical Biology and College of Pharmacy, Nankai University, Tianjin, China

**Keywords:** Small-cell lung cancer, Protein transport, Immunosuppression, Tumour immunology

## Abstract

Small cell lung cancer (SCLC) is an aggressive malignancy that responds poorly to immune checkpoint inhibitor (ICI) therapy, due to its immunosuppressive tumor microenvironment. Here, we analyse preclinical mouse tumor models and patient-derived SCLC samples and identify Exportin 1 (XPO1) as a regulator of immune suppression in SCLC. Mechanistically, XPO1 drives the nuclear export of TRIM21, enabling TRIM21-dependent proteasomal degradation of IRF3 and repressing the immunostimulatory cytokine TNFSF15. Loss of TNFSF15 promotes TREM2^+^ macrophage polarization, which subsequently attenuates macrophage-dependent IFN-γ-STAT1 signaling and MHC class I expression in tumor cells. Pharmacological or genetic XPO1 blockade restores TNFSF15, constrains TREM2^+^ macrophage differentiation, reactivates antigen presentation, and enhances anti-PD-1 efficacy in preclinical mouse models. Thus, our findings link XPO1-dependent nuclear export to TREM2+ macrophage polarization and support evaluation of XPO1 inhibition in combination with ICIs in SCLC.

## Abstract (web summary)

Small cell lung cancer (SCLC) is an aggressive malignancy characterized by an immunosuppressive tumor microenvironment (TME) resistant to Immune checkpoint inhibitor (ICI) therapy. Here, the authors define an XPO1-TRIM21-TNFSF15 axis that promotes the polarization of immunosuppressive TREM2+ macrophages, which ultimately orchestrates immune exclusion by impairing antigen presentation. Importantly, targeting XPO1 enhances the efficacy of anti-PD1 immunotherapy in mice.

## Introduction

Lung cancer stands as the malignancy with the highest global incidence and mortality rates attributed to cancer^1^. Small cell lung cancer (SCLC), a notably aggressive subtype, constitutes roughly 15% of all lung cancer cases, and its 5-year survival rate remains below 7%^2^. Platinum-based chemotherapy plus immune checkpoint inhibitor (ICI) therapy is the current standard first-line regimen for extensive-stage SCLC, but survival gains remain modest^3,4^. The relatively limited efficacy of ICI therapy in SCLC compared with non-small cell lung cancer (NSCLC) highlights the need to define immune-evasive mechanisms in SCLC and identify therapeutic vulnerabilities that can be combined with immunotherapy.

Transcription factor-based typing of SCLC (e.g., ASCL1, POU2F3, NEUROD1, and YAP1/Inflamed), or classifications derived from non-negative matrix factorization (NMF) of multi-omics data (nmf1, nmf2, nmf3, and nmf4), although providing investigators with more in-depth insights into SCLC, these typing methods have not yet been consistently validated as predictors of immunotherapy benefit^5–7^. A majority of SCLC patients do not derive durable benefit from  current ICI-based regimens^3,8,9^. This resistance is partly attributed to defective tumor antigen presentation, which leads to ineffective tumor cell recognition by immune cells, reduced immune cell infiltration, and ultimately, the formation of immune desert tumors^9,10^. Therefore, defining the mechanisms that sustain immunosuppression in the SCLC microenvironment may inform strategies to improve immunotherapy outcomes.

Tumor-associated macrophages (TAM), the most abundant immune cell population in the tumor microenvironment (TME), play essential roles in immunomodulation, phagocytosis, and antigen presentation^11,12^. Therapeutic approaches that modulate TAM biology include the inhibition of monocyte recruitment, blockade of immunosuppressive TAM activation and reprogramming toward pro-inflammatory antitumor states^13^. However, clinical strategies that directly target TAMs have had limited success, and the immunoregulatory TAM subsets that are most relevant to SCLC remain incompletely characterized. The classical M1/M2 framework does not capture the phenotypic diversity and functional plasticity of macrophages in tumors^14^. Our single-cell transcriptomic analysis identifies a transcriptionally distinct TREM2^+^ TAM population with immunosuppressive features, supporting further evaluation of this subset in SCLC immune evasion.

Exportin 1 (XPO1) is a key nuclear export receptor that mediates the transport of a wide range of proteins and mRNAs, thereby exerting wide-ranging control over cellular processes. In our prior work, we demonstrated its direct role in driving SCLC progression and drug resistance^15–17^, establishing XPO1 as a therapeutic vulnerability in this malignancy and providing a rationale for evaluating XPO1 inhibition in SCLC. Consistent with this rationale, studies from Rudin and colleagues showed that XPO1 inhibition sensitizes SCLC cells to chemotherapy and impairs neuroendocrine (NE) transformation in lung and prostate cancers^18,19^. In SCLC, the NE phenotype predominates, defined by lineage transcription factors such as ASCL1 and NEUROD1^20^, and is closely associated with immunosuppressive features^21–23^. These findings suggest that targeting XPO1 may affect both tumor-cell-intrinsic and immune-related processes.

Here, we test whether tumor-cell XPO1 shapes the SCLC immune microenvironment and limits responsiveness to PD-1 blockade. Using preclinical SCLC models and patient-derived samples, we show that XPO1 drives TRIM21-dependent IRF3 degradation, suppresses TNFSF15 expression and promotes the accumulation of immunosuppressive TREM2^+^ macrophages, thereby reducing MHC class I expression and CD8^+^ T-cell infiltration. Genetic or pharmacological XPO1 inhibition reverses these immune features and enhances anti-PD-1 activity in mouse models. These findings connect nuclear export to macrophage-mediated immune exclusion in SCLC and support XPO1 blockade as a strategy to improve immunotherapy responses in preclinical SCLC models.

## Results

### Selinexor treatment reduces TREM2^+^ macrophages in murine SCLC allograft tumors

**Fig. 1:**
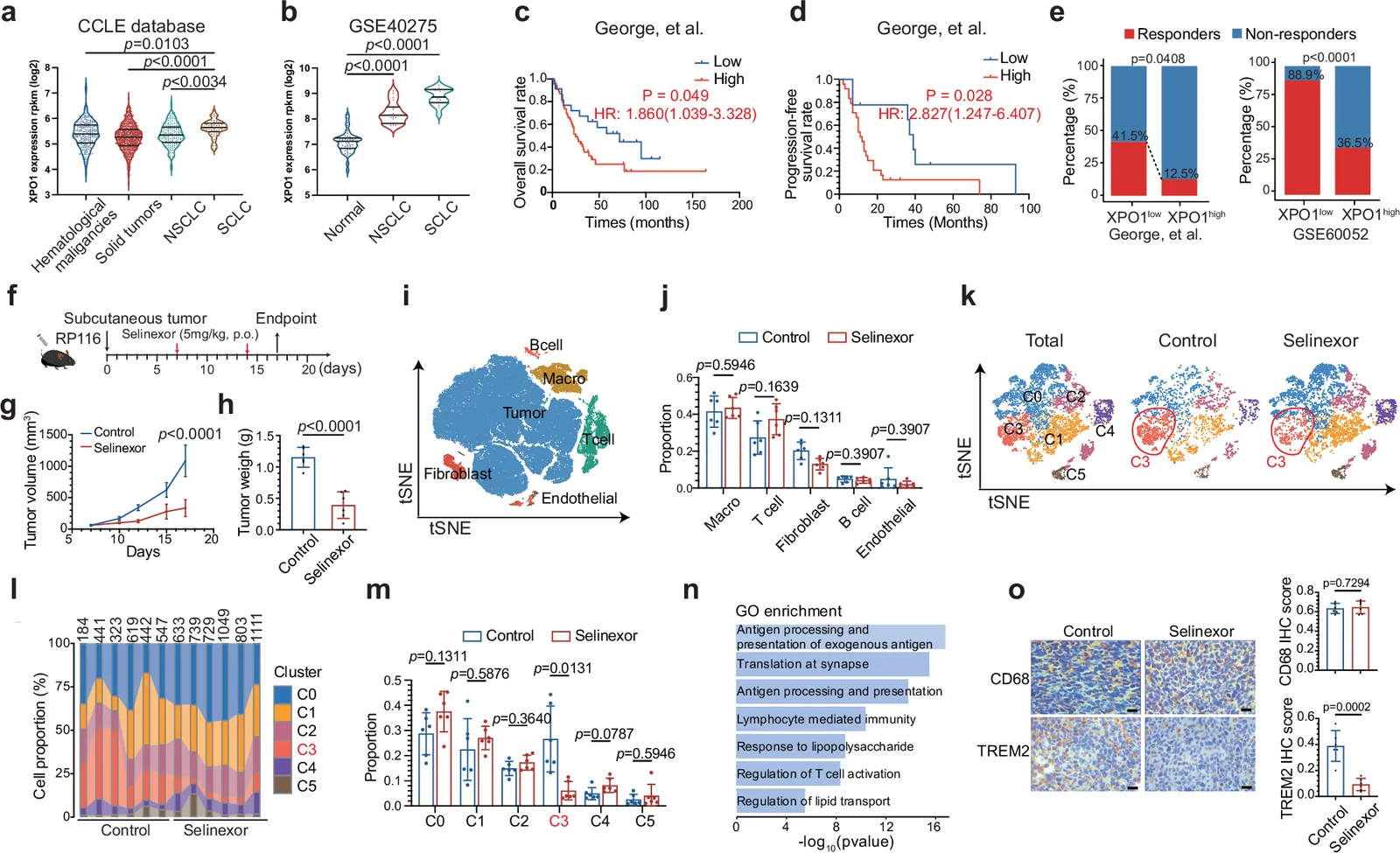
Selinexor treatment decreases TREM2^+^ macrophages in murine SCLC allograft tumors. **a** *XPO1* mRNA expression across Cancer Cell Line Encyclopedia cell lines (RPKM, log2(x + 1); *n* = 1156). **b** *XPO1* mRNA expression in normal lung, NSCLC, and SCLC samples from GSE40275 (RPKM, log2(x + 1); *n* = 77). Kaplan–Meier analyses of overall survival (**c**) and progression-free survival (**d**) in the George et al. cohort stratified by XPO1 expression. **e** Predicted ICI response in XPO1-low and XPO1-high groups in the George et al. cohort (*n* = 81) and GSE60052 (*n* = 79), inferred by TIDE algorithm. **f** Experimental design for RP116 allografts treated with selinexor or vehicle. Tumor growth curves (**g**) and tumor weights at endpoint (**h**) in control and selinexor-treated mice. **i** t-SNE projection of 105,555 single cells from 12 tumors showing six major cell populations. **j** Proportions of each indicated non-tumor cell type in control and selinexor-treated tumors. **k** t-SNE maps of macrophages from all, control and selinexor-treated tumors; cluster 3 is outlined. **l** Proportions of macrophage clusters in control and selinexor-treated tumors. **m** Statistical comparison of the macrophage cluster proportions shown in (**l**). **n** Gene Ontology (GO) enrichment analysis of genes enriched in cluster 3 macrophages. The top eight enriched GO biological process terms ranked by adjusted *P* value are shown. **o** Representative CD68 and TREM2 immunohistochemistry and quantification. Scale bars, 20 μm. Data in (**a**, **b**) were analyzed by one-way ANOVA followed by Tukey’s multiple-comparisons test. Data in (**c**, **d**) were compared using the two-sided log-rank test. Proportions in (**e**) were compared using a two-sided Fisher’s exact test. Data in (**g**) were analyzed by two-way repeated-measures ANOVA followed by Sidak’s multiple-comparisons test. Data in (**h**, **o**) were compared using a two-sided unpaired *t*-tests. Data in (**j**, **m**) were analyzed using two-sided multiple Mann–Whitney tests with Benjamini–Hochberg false discovery rate correction. Data in (**n**) was assessed using a one-sided hypergeometric test with Benjamini–Hochberg correction. Data in (**f**–**o**) are from *n* = 6 mice per group. Data are presented as mean ± s.d. Source data are provided as a Source Data file.

XPO1 expression was increased in SCLC tissues compared with normal lung tissues, NSCLC, other solid tumors, and hematologic malignancies, as shown in public cell-line and patient transcriptomic datasets (Fig. 1a, b). Re-analysis of publicly available SCLC transcriptomic datasets showed that XPO1 expression was broadly distributed across the major SCLC subtypes (SCLC-A, SCLC-N, SCLC-P, and SCLC-Y), without significant enrichment in any single subtype (Supplementary Fig. 1a, b). Additionally, *XPO1* mRNA expression did not correlate significantly with the NE lineage markers *ASCL1* (Pearson *r *= 0.1505, *p *= 0.1854) or *NEUROD1* (Pearson *r *= −0.1658, *p *= 0.1443) in the GSE60052 dataset (Supplementary Fig. 1c, d), suggesting that XPO1 expression is not restricted to a specific SCLC subtype. Survival analyses showed that high XPO1 expression was associated with shorter overall survival (HR = 1.860, 95% CI: 1.039–3.328, *P *= 0.049) and progression-free survival (HR = 2.827, 95% CI: 1.247–6.407, *P *= 0.028) (Fig. 1c, d). Patients with elevated XPO1 levels were also predicted to derive less benefit from immune checkpoint blockade (Fig. 1e, Supplementary Fig. 1e).

To evaluate the effects of XPO1 inhibition on the SCLC immune microenvironment, RP116 syngeneic tumors were treated with selinexor according to the schedule shown in Fig.1f. Endogenous XPO1 expression in the cell models is shown in Supplementary Fig. 1f. Selinexor significantly reduced both tumor volume and weight compared with vehicle controls (Fig. 1g, h, Supplementary Fig. 1g). Single-cell RNA sequencing on 105,555 cells from allograft tumors identified 18 transcriptionally distinct clusters (Supplementary Fig. 1h, i), which were annotated as tumor cells (*Epcam, Krt8*), macrophages (*Cd80, Il1b, Cd86*), T cells (*Cd3d*, *Cd3e*), B cells (*Cd79a*, *Mzb1*), and fibroblasts (*Col1a1*, *Ddr2*) (Fig. 1i, Supplementary Fig. 1j, k). Within the tumor compartment, selinexor treatment was associated with reduced expression of several NE-associated genes, including *Chga, Syp*, and *Bex1* (Supplementary Fig. 1l), whereas the overall NE program was not altered (Supplementary Fig. 1m). Selinexor treatment did not alter the global proportions of major stromal or immune cell populations, including fibroblasts, T cells, macrophages, B cells, and endothelial cells (Fig. 1j and Supplementary Fig. 1n). Re-clustering of T cells identified five subsets and showed a modest increase in the relative abundance of cytotoxic T cells after selinexor treatment (Supplementary Fig. 2a–e). Because broad changes in major T-cell subsets were not observed, this finding alone was not interpreted as evidence of T-cell functional reinvigoration. By contrast, macrophage re-analysis revealed a more prominent and selective reduction in cluster 3 after XPO1 inhibition (Fig. 1k–m and Supplementary Fig. 2f). This population co-expressed *Mrc1* (Cd206) and *Trem2*, together with multiple genes previously associated with immunoregulatory macrophage states, including *Gas6*^24^ and *Maf*^25^, and genes involved in lipid metabolism and antigen processing and presentation (Fig. 1n and Supplementary Fig. 2g–j). Consistent with the single-cell data, immunohistochemistry (IHC) showed comparable overall CD68^+^ macrophage infiltration between selinexor-treated and control tumors, whereas TREM2^+^ macrophage infiltration was reduced after selinexor treatment (Fig. 1o). Given emerging evidence that TREM2^+^ TAMs represent an immunosuppressive myeloid subset linked to T-cell dysfunction and immunotherapy resistance^26,27^, we therefore focused our subsequent analyses on this population.

Consistent with these observations, deconvolution analysis of the George et al. SCLC cohort showed that macrophages constituted the predominant immune population in the SCLC TME, accounting for 31.6% of total immune cells, and that a higher M1/M2 macrophage ratio was associated with more favorable survival (Supplementary Fig. 3a, b). Moreover, XPO1 expression inversely correlated with the M1/M2 ratio (Supplementary Fig. 3c), supporting an association between XPO1 and macrophage polarization. Together, these findings indicate that XPO1 inhibition may reshape macrophage states within the immunosuppressive SCLC microenvironment.

### XPO1 promotes polarization toward a TREM2⁺ immunosuppressive macrophage state in SCLC

**Fig. 2:**
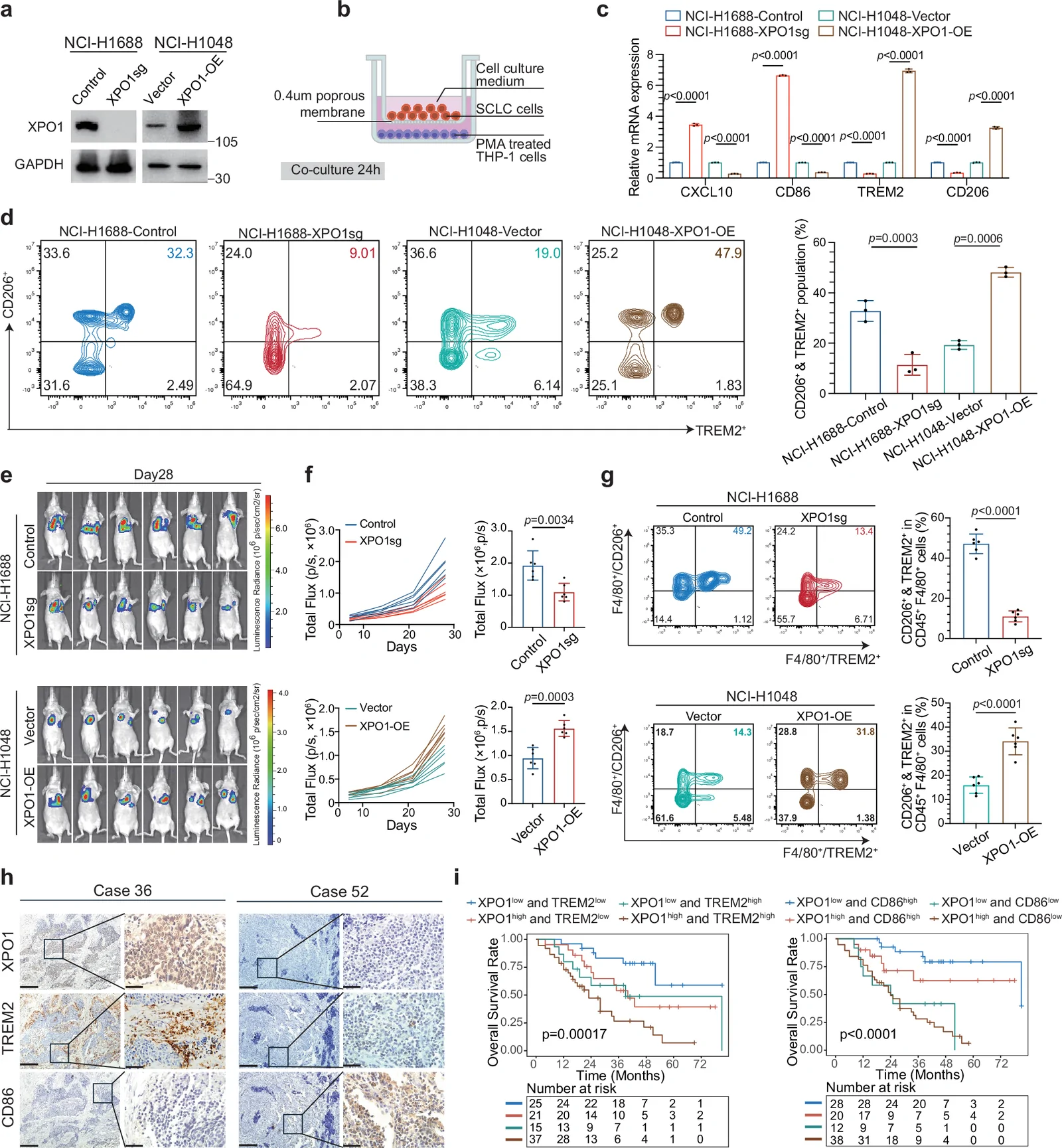
XPO1 drives macrophage polarization toward a TREM2^+^ phenotype in SCLC. **a** XPO1 expression in NCI-H1688 control and XPO1-knockdown cells, and in NCI-H1048 vector-control and XPO1-overexpressing (XPO1-OE) cells, was validated by western blotting. **b** Schematic of the in vitro co-culture system in which PMA-differentiated THP-1-derived macrophages were cultured with the indicated SCLC cells for 24 h. This schematic was created using Adobe Illustrator and contains no third-party material. **c** RT-qPCR analysis of *CD86*, *CXCL10*, *TREM2*, and *CD206* mRNA expression in THP-1-derived macrophages following co-culture with the indicated SCLC cells. **d** Flow cytometric analysis of TREM2 and CD206 surface expression on THP-1-derived macrophages after co-culture with the indicated SCLC cells. Representative flow cytometry plots and quantification are shown. **e** Orthotopic xenograft growth of luciferase-labeled indicated SCLC cells (1 × 10^6^) in BALB/c nude mice. Representative bioluminescence images acquired on day 28. **f** Longitudinal bioluminescence imaging (BLI) quantification of tumor burden in mice. Individual lines represent BLI signal intensity from individual mice over time. Right, quantification of BLI signal intensity at day 28. **g** Flow cytometric analysis of CD206^+^ & TREM2^+^ cells within the F4/80^+^ macrophage compartment isolated from orthotopic tumors. Representative plots (left) and quantification (right) are shown. **h** Representative immunohistochemical staining for XPO1, CD86, and TREM2 in human SCLC tissues (*n* = 98). Scale bars, 200 μm and 40 μm, as indicated. **i** Kaplan–Meier analysis of overall survival in patients with SCLC stratified by XPO1, CD86, and TREM2 expression. Patients were stratified into low- and high-expression groups according to the optimal cut-off value. Data in (**c**) were analyzed using multiple two-sided unpaired *t*-tests with Holm–Sidak correction. Data in (**d**, **f**, **g**) were calculated using two-sided unpaired *t*-tests. Data in (**i**) were analyzed using the two-sided log-rank test. Data in (**a**, **c**, **d**) are from *n* = 3 biologically independent experiments. Data in (**e**–**g**) are from *n* = 6 mice per group. Data are presented as mean ± s.d. Source data are provided with this paper.

To investigate how tumor-cell XPO1 regulates macrophage differentiation, we examined XPO1 expression across a panel of nine SCLC cell lines representing major molecular subtypes. XPO1 was expressed across all subtypes, with the highest level in NCI-H1688 (SCLC-A) and the lowest in NCI-H1048 (SCLC-P) (Supplementary Fig. 4a). We therefore selected these two lines as complementary core models for loss-of-function and gain-of-function studies, respectively (Fig. 2a). To assess the consistency of the observed phenotype across additional subtype contexts, selected key mechanistic experiments were further extended to DMS273 (SCLC-N), DMS114 (SCLC-Y), and NCI-H196 (SCLC-Y) (Supplementary Fig. 4b).

We next established co-culture systems with the indicated SCLC cells and macrophages to determine whether tumor-cell XPO1 directly influences macrophage polarization (Fig. 2b). In this setting, THP-1-derived macrophages co-cultured with XPO1-depletion NCI-H1688 cells displayed increased CD86 expression and a reduced proportion of CD206^+^ & TREM2^+^ macrophages relative to macrophages co-cultured with control cells (Fig. 2c, d and Supplementary Fig. 4c). Conversely, macrophages co-cultured with XPO1-overexpressing NCI-H1048 cells showed reduced CD86 expression and an increased CD206^+^ & TREM2^+^ population (Fig. 2c, d and Supplementary Fig. 4c). These findings were further validated in peripheral blood mononuclear cell (PBMC)-derived macrophages: XPO1 depletion in NCI-H1688 cells reduced, whereas XPO1 overexpression in NCI-H1048 cells increased, the proportion of CD206^+^ & TREM2^+^ macrophages (Supplementary Fig. 4d). To test whether this phenotype extended to an additional subtype context, we analyzed DMS273 cells. Again, XPO1 overexpression in DMS273 cells also promoted the emergence of CD206^+^ & TREM2^+^ macrophages in both THP-1-derived and PBMC-derived co-culture systems (Supplementary Fig. 4e, f). These results indicate that tumor-intrinsic XPO1 serves as a consistent regulator of TREM2^+^ macrophage polarization.

To further assess the immunoregulatory role of TREM2^+^ macrophages, we sorted TREM2^+^ and TREM2^−^ macrophages from co-culture systems. Enzyme-linked immunosorbent assay (ELISA) and reverse transcription quantitative PCR (RT-qPCR) analyses of conditioned media showed that TREM2^+^ macrophages secreted higher levels of IL-10 and TGF-β than TREM2^−^ macrophages (Supplementary Fig. 4g, h). In co-culture assays, CD8^+^ T cells isolated from PBMCs and co-cultured with TREM2^+^ macrophages exhibited lower IFN-γ expression than those co-cultured with TREM2⁻ macrophages (Supplementary Fig. 4i). These findings support the interpretation that TREM2^+^ macrophages represent an immunoregulatory macrophage subset in the SCLC microenvironment.

We next examined this relationship in orthotopic lung xenograft models in BALB/c nude mice. Here, XPO1 depletion significantly reduced tumor burden compared with controls expressing endogenous XPO1, whereas XPO1 overexpression increased tumor burden (Fig. 2e, f). Despite these effects on tumor growth, F4/80-positive macrophage infiltration did not differ significantly between the groups (Supplementary Fig. 4j). Phenotypic analysis of macrophages within the TME showed that XPO1 depletion increased the proportion of CD86-positive macrophages and decreased the proportion of CD206^+^ & TREM2^+^ macrophages (Fig. 2g, Supplementary Fig. 4k–m). Conversely, XPO1 overexpression reduced CD86^+^ macrophages and increased CD206^+^ & TREM2^+^ macrophages (Fig. 2g, Supplementary Fig. 4k–m).

We then assessed whether XPO1 expression and macrophage markers were associated with clinical outcome in SCLC. IHC staining of serial sections from 98 SCLC surgical specimens showed that high tumor XPO1 expression was associated with elevated TREM2 expression and reduced CD86 expression (Fig. 2h and Supplementary Table 1). Patients with XPO1^Low^ & TREM2^Low^ or XPO1^Low^ & CD86^High^ tumors had more favorable overall survival, whereas patients with XPO1^High^ & TREM2^High^ or XPO1^High^ & CD86^Low^ tumors had poorer outcomes (Fig. 2i). Multivariate Cox regression analysis showed that the XPO1^High^ & CD86^Low^ and XPO1^High^ & TREM2^High^ phenotypes were independently associated with poorer overall survival (Supplementary Table 2). These clinical data support an association between elevated tumor XPO1, TREM2-skewed macrophage polarization and adverse prognosis in SCLC.

### XPO1 suppresses TNFSF15 expression by inhibiting IRF3-mediated transcriptional activity

**Fig. 3:**
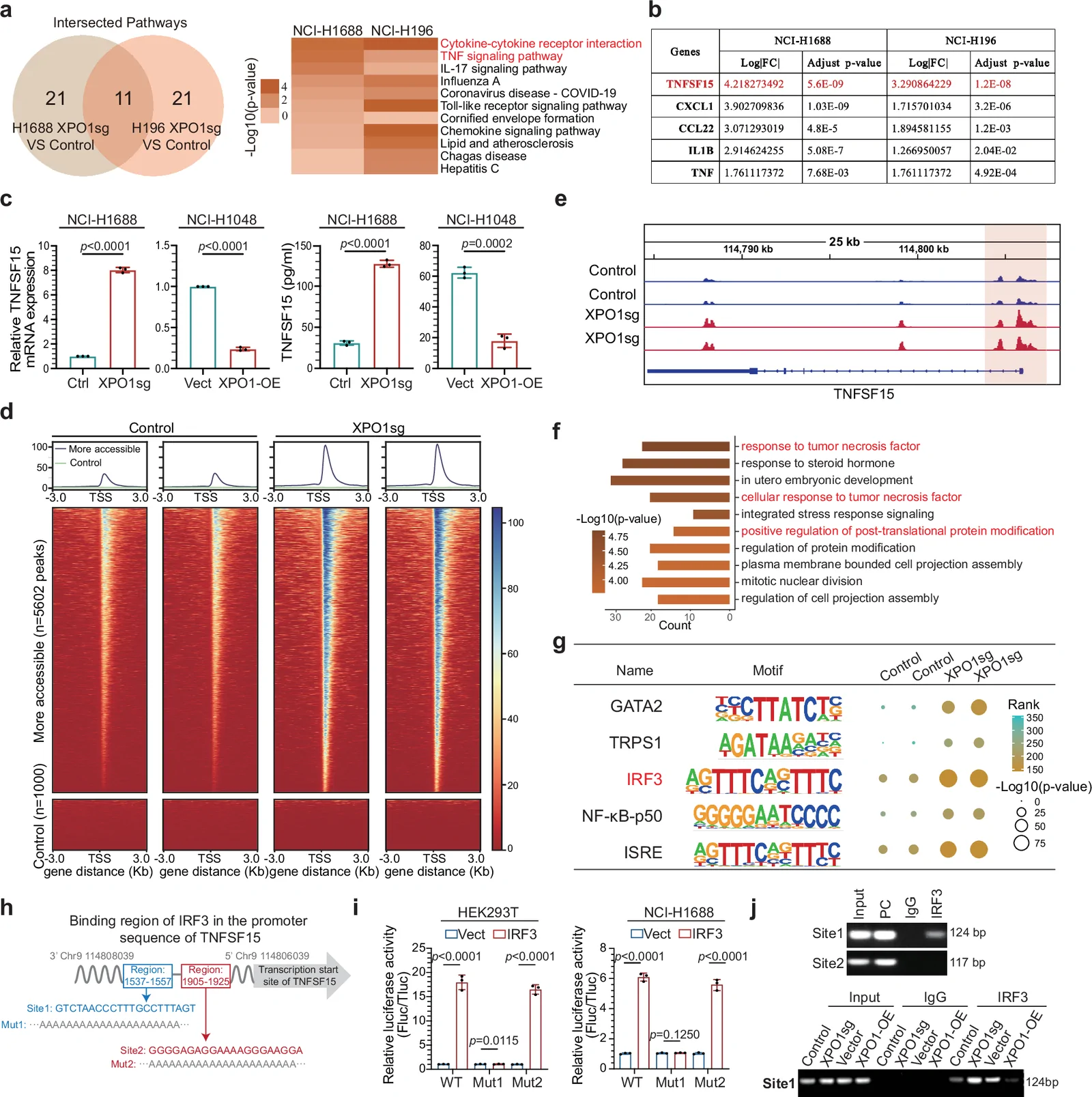
XPO1 suppresses TNFSF15 expression by inhibiting IRF3-mediated transcriptional activation. **a** GSEA of transcriptional changes after XPO1 depletion in NCI-H1688 and NCI-H196 cells, showing overlapping dysfunctional Hallmark gene sets (left) and the 11 shared gene sets (right). **b** Differential expression of five commonly upregulated cytokine- and TNF-related genes in *XPO1*-silenced cells. **c** RT-qPCR and ELISA analysis of TNFSF15 expression and secretion. **d** ATAC-seq signal at regions with increased accessibility after XPO1 depletion, centered within ±3 kb of differential peaks. **e** ATAC-seq tracks at the TNFSF15 locus. **f** GO enrichment analysis of genes associated with increased promoter accessibility following XPO1 depletion. The top ten enriched terms are shown. **g** HOMER motif analysis of differentially accessible chromatin regions and the top five upregulated transcription factors in XPO1-silenced cells. **h** Predicted IRF3-binding sites in the TNFSF15 promoter and the corresponding mutant sequences. This schematic was created by the authors using Adobe Illustrator and contains no third-party material. **i** Dual-luciferase reporter assay in NCI-H1688 cells expressing IRF3 and reporter constructs containing the wild-type *TNFSF15* promoter or promoter variants with mutations in the indicated predicted IRF3-binding sites. **j** ChIP-qPCR analysis of IRF3 occupancy at the TNFSF15 promoter. Representative images are shown. Data in (**a**) were calculated using a sign-specific permutation test based on permutation-generated enrichment-score null distributions; FDR *q* values were estimated within the GSEA framework. Data in (**b**) was analyzed using DESeq2; two-sided Wald-test *P* values were adjusted using the Benjamini–Hochberg method. Data in (**c**, **i**) were analyzed using two-sided unpaired Student’s *t* tests. Data in (**f**) was assessed with GO term enrichment analysis using a one-sided hypergeometric test with Benjamini–Hochberg correction. Data in (**g**) was analyzed using HOMER with GC- and length-matched background sequences. Data in (**a**, **b**) are from *n* = 3 biologically independent samples per group; Data in (**c**, **i**, **j**) are from *n* = 3 biologically independent experiments. ATAC-seq analyses in (**d**, **g**) are from *n* = 2 biologically independent samples per group. Data in (**c**, **i**) are presented as mean±s.d. Source data are provided as a Source Data file.

To define the transcriptional consequences of XPO1 depletion, we performed bulk RNA-seq in two independent XPO1-high SCLC cell lines, NCI-H1688 and NCI-H196, after XPO1 knockdown. Differential expression and pathway enrichment analyses revealed significant changes in cytokine-cytokine receptor interaction and TNF signaling pathways (Fig. 3a). Notably, *TNFSF15* showed the largest increase among these shared genes, suggesting a potential role in immune regulation and macrophage differentiation (Fig. 3b)^28,29^. RT-qPCR and ELISA confirmed that XPO1 depletion increased TNFSF15 expression, whereas XPO1 overexpression suppressed it (Fig. 3c, Supplementary Fig. 5a). Analysis of the GSE60052 dataset also revealed an inverse correlation between *XPO1* and *TNFSF15* expression in SCLC (*r* = −0.46, *p *< 0.001), further supporting a negative regulatory relationship (Supplementary Fig. 5b). In NCI-H1688 cells, either TNFSF15 knockdown in SCLC cells or addition of a TNFSF15-neutralizing antibody to the co-culture system attenuated the inhibitory effect of *XPO1* loss on CD206^+^ & TREM2^+^ macrophage polarization (Supplementary Fig. 5c–e). Conversely, the introduction of exogenous recombinant TNFSF15 or lentiviral TNFSF15 expression in NCI-H1048/XPO1-OE cells counteracted the tendency of XPO1 overexpression to drive macrophage differentiation toward the TREM2-like phenotype (Supplementary Fig. 6a–c). These rescue experiments support a role for tumor-derived TNFSF15 in limiting the immunosuppressive macrophage polarization.

To determine whether TNFSF15 directly activates downstream signaling in macrophages, we treated THP-1-derived M0 macrophages with recombinant TNFSF15 (1 μg/ml, 24 h) and performed bulk RNA-seq. Differential expression and Kyoto Encyclopedia of Genes and Genomes (KEGG) pathway enrichment analyses revealed significant upregulation of genes involved in the TNF, NF-κB, and JAK-STAT signaling pathways (Supplementary Fig. 7a, b), indicating that TNFSF15 induces an inflammatory transcriptional response in macrophages. Consistent with these transcriptomic changes, immunoblot analysis showed increased phosphorylation of p65 (Ser536) and STAT1 (Tyr701) after TNFSF15 stimulation (Supplementary Fig. 7c), indicating that recombinant TNFSF15 can activate downstream inflammatory signaling in macrophages. In parallel, in tumor-macrophage co-culture experiments, macrophages co-cultured with XPO1-knockdown NCI-H1688 cells exhibited increased p-STAT1 and p-p65, whereas those co-cultured with XPO1-overexpressing NCI-H1048 cells showed reduced phosphorylation of these signaling proteins (Supplementary Fig. 7d). These co-culture data are correlative and reflect the combined effects of tumor-cell secretome changes upon XPO1 modulation, with TNFSF15 likely representing an important, but not necessarily exclusive, contributor.

We further assessed expression of the canonical TNFSF15 death receptor 3 (DR3) in our assay system. Western blotting analysis showed that DR3 was detectable in both THP-1-derived and PBMC-derived macrophages, whereas its expression was lower in SCLC cell lines (Supplementary Fig. 7e). Notably, within the macrophage compartment, TREM2^+^ macrophages exhibited a lower frequency of DR3-positive cells than TREM2^-^ macrophages (Supplementary Fig. 7f, g). These data suggest that TNFSF15 responsiveness may differ across macrophage states and support macrophages as the more likely TNFSF15-responsive population in this co-culture system. While these results support the direct activation of NF-κB and STAT pathways by TNFSF15 in macrophages, further studies are needed to fully characterize receptor utilization and assess the relative contributions of TNFSF15 versus other co-culture-derived signals in vivo.

To explore the upstream regulation of TNFSF15, we performed assay for transposase-accessible chromatin using sequencing (ATAC-seq), which identified 5602 peaks with increased chromatin accessibility in *XPO1-*deficient cells relative to the control group (Fig. 3d). Increased accessibility was observed at *TNFSF15* promoter loci (Fig. 3e), suggesting potential activation by transcription factors. Functional enrichment analysis showed upregulation of the TNF response pathway and changes in post-translational protein modification programs in *XPO1*-deficient cells (Fig. 3f). Motif enrichment and bias-corrected transcription factor footprint analyses identified enrichment of IRF3 motifs in regions with increased accessibility, and TOBIAS-based footprinting showed enhanced predicted IRF3 occupancy after *XPO1* loss (Fig. 3g and Supplementary Fig. 8a–d), implicating IRF3 as a candidate mediator of TNFSF15 activation. Functionally, silencing *IRF3* in *XPO1*-deficient NCI-H1688 cells significantly reduced both *TNFSF15* mRNA expression and protein secretion, whereas pharmacological activation of IRF3 with KIN1400 rescued TNFSF15 expression suppressed by XPO1 overexpression in NCI-H1048 cells (Supplementary Fig. 8e, f). Transcription factor binding-site analysis identified two conserved IRF3 binding sites within the *TNFSF15* promoter (Fig. 3h). Luciferase reporter assays showed that IRF3 selectively enhanced transcription through the Site 1, but not the Site 2 (Fig. 3i). Chromatin immunoprecipitation (ChIP) further confirmed IRF3 binding Site 1 and showed increased enrichment in *XPO1*-deficient cells (Fig. 3j and Supplementary Fig. 8g). These findings support a model in which XPO1 represses *TNFSF15* transcription by inhibiting IRF3-mediated promoter activation.

### XPO1-mediated nuclear export of TRIM21 facilitates proteasomal degradation of IRF3

**Fig. 4:**
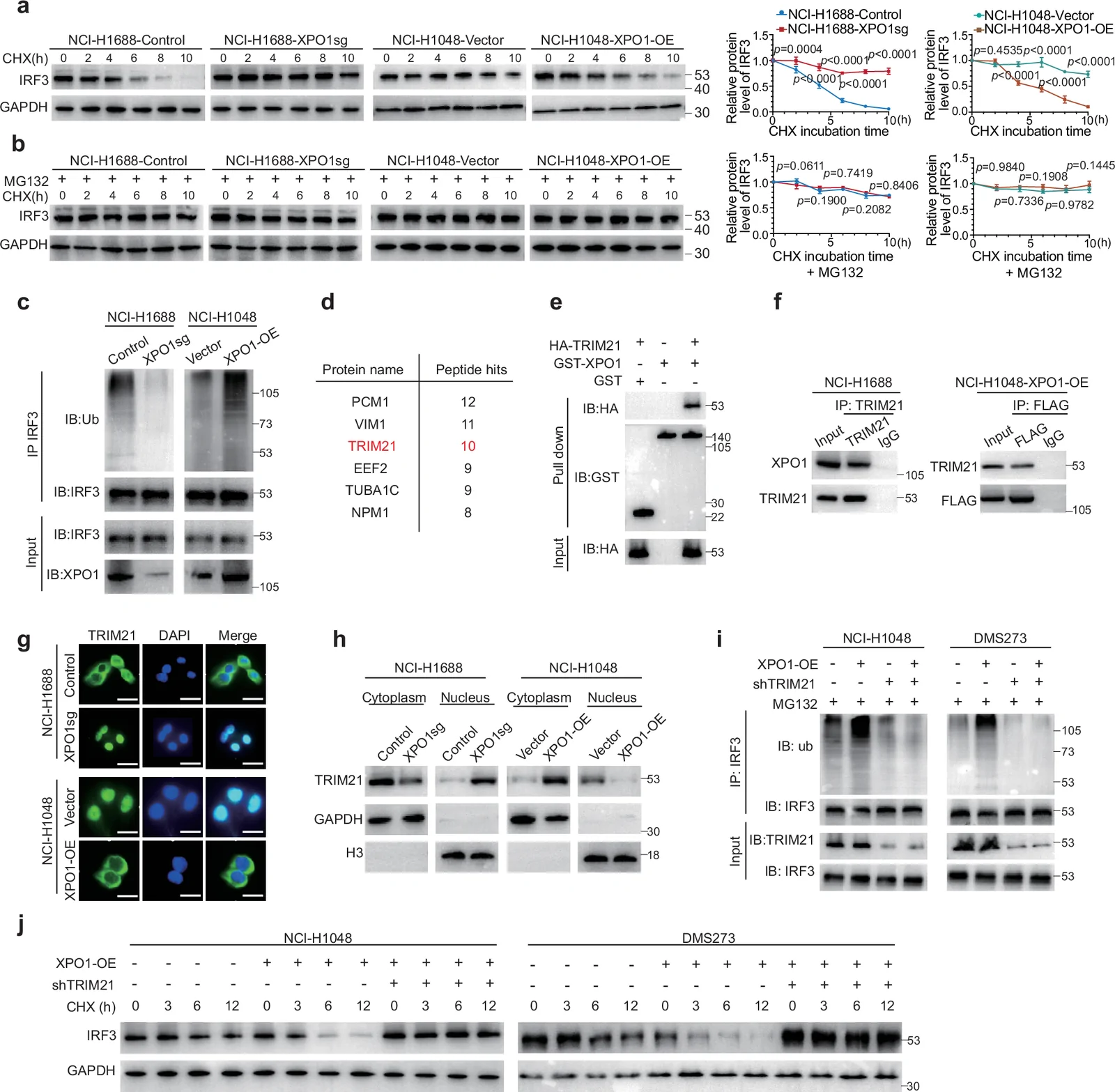
XPO1 promotes TRIM21 cytoplasmic localization and facilitates proteasomal degradation of IRF3. Cycloheximide (CHX)-chase analysis of IRF3 degradation in the indicated SCLC cells without (**a**) or with MG132 pretreatment (**b**); representative immunoblots and quantification are shown. **c** Following MG132 treatment, cells were subjected to immunoprecipitation with an anti-IRF3 antibody, and polyubiquitylated IRF3 was detected by immunoblotting with an anti-ubiquitin antibody. **d** Representative XPO1-interacting proteins identified by mass spectrometry analysis. **e** In vitro GST pull-down using recombinant GST-XPO1 and HA-TRIM21 proteins to verify the direct interaction between XPO1 and TRIM21. **f** Co-immunoprecipitation analysis of the interaction between XPO1 and TRIM21 in NCI-H1688 cells and NCI-H1048 cells expressing XPO1-FLAG. **g** Immunofluorescence analysis of TRIM21 subcellular localization in NCI-H1688/*XPO1*-silenced and NCI-H1048/XPO1-overexpressing cells. Representative images from three biologically independent experiments are shown. Scale bar, 20 μm. **h** Immunoblot analysis of TRIM21 in cytoplasmic and nuclear fractions; GAPDH and histone H3 served as fractionation controls. **i** IRF3 polyubiquitylation in XPO1-overexpressing NCI-H1048 and DMS273 cells with or without TRIM21 silencing. **j** CHX-chase analysis of IRF3 degradation under the same conditions. Representative blots are shown. Data in (**a**, **b**) were assessed by two-way ANOVA followed by Sidak’s multiple-comparisons test. Data in (**a**–**c**) and (**e**–**j**) are representative of *n* = 3 biologically independent experiments. Data are presented as mean ± s.d. Source data are provided as a Source Data file.

To investigate the mechanism by which XPO1 regulates IRF3 expression, we assessed endogenous IRF3 protein levels. Genetic ablation of XPO1 increased IRF3 protein abundance, while XPO1 overexpression significantly reduced it (Supplementary Fig. 9a). In contrast, XPO1 manipulation had no discernible effect on *IRF3* mRNA levels (Supplementary Fig. 9b), nor did it alter IRF3 translation efficiency, as determined by polysome profiling (Supplementary Fig. 9c). These findings point to a post-translational regulatory mechanism. To evaluate whether XPO1 influences IRF3 protein stability, we treated SCLC cells with the protein synthesis inhibitor cycloheximide (CHX) and monitored IRF3 degradation kinetics. *XPO1*-deficient cells exhibited delayed IRF3 turnover, whereas IRF3 degradation was accelerated in XPO1-overexpressing cells (Fig. 4a), confirming that XPO1 promotes IRF3 destabilization. Consistent with proteasome-dependent degradation, treatment with the proteasome inhibitor MG132 abolished the effect of XPO1 knockdown on IRF3 protein stability and rescued the decreased IRF3 protein levels triggered by XPO1 overexpression (Fig. 4b and Supplementary Fig. 9d). In contrast, treatment with chloroquine (Chl) had no significant effect on degradation kinetics of IRF3 in SCLC cells subjected to CHX treatment (Supplementary Fig. 9e), indicating negligible contribution of lysosomal proteolysis to XPO1-mediated IRF3 stabilization. Further supporting a proteasomal mechanism, we observed that XPO1 overexpression enhanced IRF3 polyubiquitination, whereas XPO1 knockdown reduced it (Fig. 4c and Supplementary Fig. 9f). Collectively, these data establish that XPO1 facilitates IRF3 degradation via the ubiquitin-proteasome system.

To elucidate the upstream mediators of this process, we performed mass spectrometry analysis and identified the top six proteins that specifically co-immunoprecipitated with XPO1. Among these candidates, TRIM21, an E3 ubiquitin ligase, is a key regulator within the ubiquitin-proteasome system. This finding, together with our prior evidence implicating ubiquitination pathways, led us to focus on TRIM21 for subsequent mechanistic investigation (Fig. 4d, Supplementary Fig. 10a, and Supplementary Data 1). Sequence analysis identified a putative leucine-rich nuclear export signal (NES)-like motif (ALELLQEVIIVL) in TRIM21 (Supplementary Fig. 10b), and GST pull-down assays demonstrated direct interaction between XPO1 and TRIM21, supporting a model in which TRIM21 may serve as a direct XPO1 cargo (Fig. 4e). Co-immunoprecipitation (Co-IP) confirmed endogenous XPO1 binds TRIM21 in NCI-H1688 cells, and exogenous XPO1 forms a stable complex with TRIM21 in XPO1-overexpressing NCI-H1048 cells (Fig. 4f). Importantly, XPO1 overexpression promoted the nuclear export and cytoplasmic accumulation of TRIM21, whereas XPO1 deletion led to nuclear retention of TRIM21 and its depletion from the cytoplasm (Fig. 4g, h and Supplementary Fig. 10c, d). Consistently, TRIM21 ablation abolished XPO1-driven polyubiquitination and proteasomal degradation of IRF3 in NCI-H1048 and DMS273 cells (Fig. 4i, j). Together, these data define a mechanistic axis wherein XPO1 governs TRIM21 subcellular localization to regulate IRF3 proteostasis through ubiquitin-proteasome-dependent degradation.

### TRIM21 catalyzes K48-linked ubiquitination of IRF3 at lysine 313

**Fig. 5:**
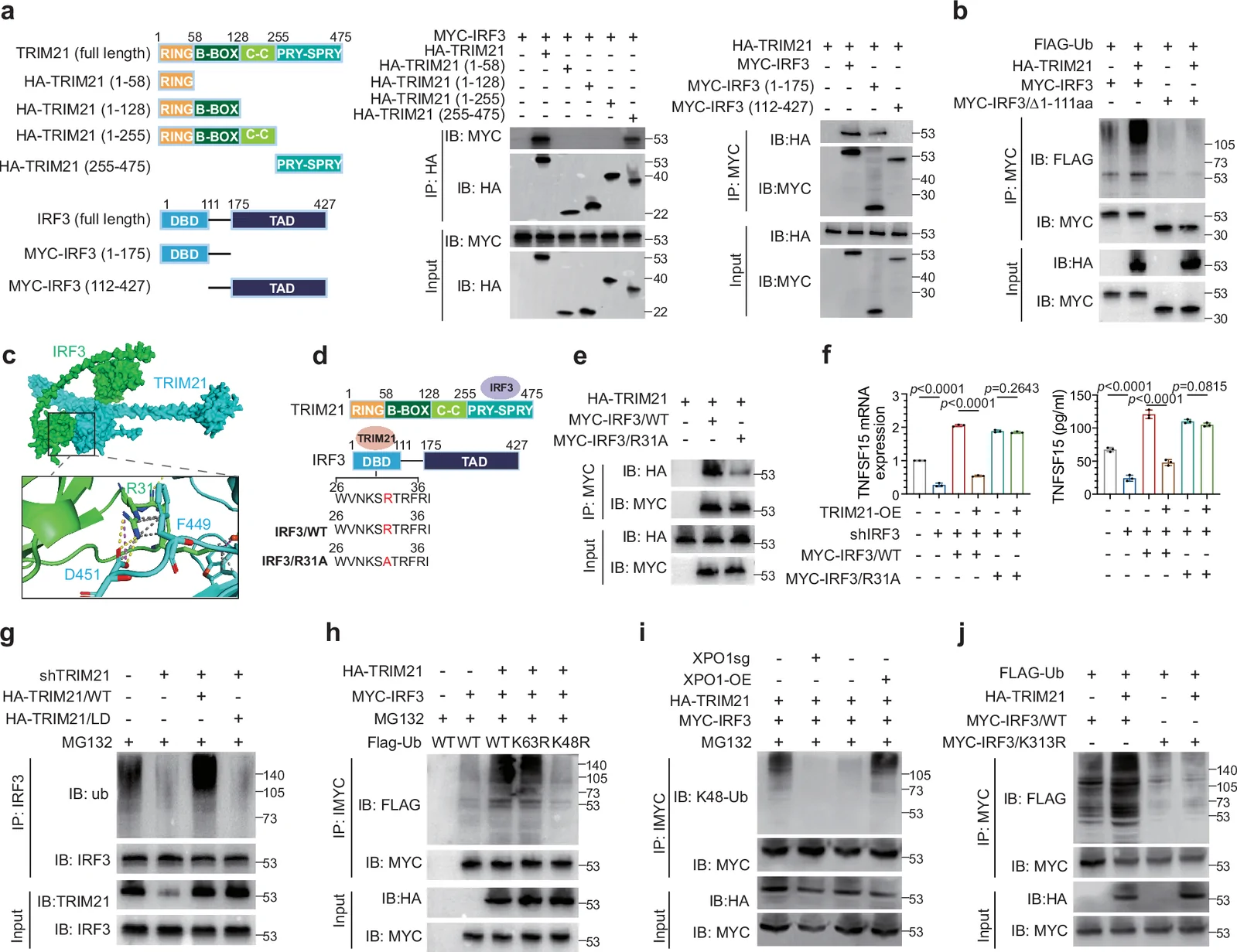
TRIM21 catalyses K48-linked polyubiquitylation of IRF3 at lysine 313. **a** Schematic representation of the domain structures of TRIM21 and IRF3 and the truncation mutants used to map their interaction (left). Immunoprecipitation and immunoblot analysis of lysates from HEK293T cells expressing the indicated deletion mutants (right). This schematic was created by the authors using Adobe Illustrator and contains no third-party material. **b** Deletion of the DNA-binding domain (DBD) of IRF3 impaired TRIM21-mediated polyubiquitylation of IRF3. **c** Modeled TRIM21-IRF3 complex highlighting interactions between IRF3 R31 and TRIM21 F449/D451. Putative polar interactions are indicated by yellow dashed lines. **d** Schematic representation of IRF3 and the indicated mutant constructs. This schematic was created by the authors using Adobe Illustrator and contains no third-party material. **e** Immunoprecipitation and immunoblot analysis of lysates from HEK293T cells expressing HA-TRIM21 together with MYC-IRF3 or MYC-IRF3-R31A. **f** RT-qPCR and ELISA analysis of *TNFSF15* mRNA expression and secreted protein levels, respectively, in the indicated NCI-H1048 cells. **g** Ubiquitylation assay in *TRIM21*-knockdown NCI-H1688 cells reconstituted with wild-type (WT) or ligase-dead (LD) HA-tagged TRIM21. **h** IRF3 polyubiquitylation in HEK293T cells expressing wild-type ubiquitin, Ub-K48R or Ub-K63R. **i** K48-linked polyubiquitylation of IRF3 after XPO1 manipulation. **j** IRF3 polyubiquitylation after re-expression of wild-type or mutant IRF3 in TRIM21-depleted cells with or without TRIM21 rescue. Data in (**f**) was analyzed using two-sided unpaired *t*-tests. Data in (**a**, **b**) and (**e**–**j**) are representative of *n* = 3 biologically independent experiments. Data are presented as mean ± s.d. Source data are provided as a Source Data file.

To determine whether TRIM21 directly ubiquitinates IRF3, we initially confirmed the endogenous interaction between TRIM21 and IRF3 in SCLC cells and validated their exogenous interaction in HEK293T cells (Supplementary Fig. 10e, f). To map the interaction interface, we generated TRIM21 truncation mutants. Co-IP assays showed that a truncation comprising amino acids 1–255 (aa 1–255) failed to interact with IRF3, whereas the PRY-SPRY domain (aa 255–475) retained binding, indicating that the PRY-SPRY region contributes to the TRIM21-IRF3 interaction (Fig. 5a). We then mapped the interacting region on IRF3 using domain-deletion mutants co-transfected with HA-tagged TRIM21 plasmids. The DNA-binding domain (DBD) truncation (aa 1–175), but not the transactivation domain truncation (aa 112–427), retained binding to TRIM21 (Fig. 5a). An IRF3 mutant lacking the DBD (aa 1–111 deletion) was resistant to TRIM21-induced ubiquitination (Fig. 5b), implicating this region in TRIM21-mediated modification. An AlphaFold3 structural docking model predicted interaction between the PRY-SPRY domain of TRIM21 and the DBD of IRF3, mediated in part by contacts between IRF3 R31 and TRIM21 residues F449 and D451 (Fig. 5c). Consistent with this prediction, an IRF3 R31A mutant showed reduced binding to TRIM21 compared with wild-type IRF3 (Fig. 5d, e). In *IRF3*-depletion NCI-H1048 cells, re-expression of IRF3/R31A attenuated TRIM21-mediated suppression of *TNFSF15* transcription and secretion (Fig. 5f). These results support a role for IRF3 in the TRIM21-IRF3 interaction and in regulation of TNFSF15 expression.

In vitro ubiquitination assays using purified proteins showed that TRIM21 directly catalyzed IRF3 ubiquitination (Supplementary Fig. 10g). Depletion of endogenous TRIM21 reduced IRF3 ubiquitination levels, and this effect was not rescued by overexpression of a ligase-dead TRIM21 mutant (TRIM21/LD), supporting dependence on TRIM21 ligase activity (Fig. 5g). Mutation of ubiquitin at Lys48 (Ub-K48R), but not at Lys63 (Ub-K63R), impaired TRIM21-mediated ubiquitination of IRF3 (Fig. 5h). Consistently, K48-linked ubiquitination of IRF3 was significantly reduced in XPO1-knockdown cells, whereas XPO1 overexpression increased K48-linked ubiquitination of IRF3 (Fig. 5i), supporting the involvement of K48-linked polyubiquitination in this context. Mass spectrometry identified a ubiquitinated peptide consistent with modification at K313 in IRF3 (Supplementary Fig. 10h). Mutation of K313 to arginine impaired TRIM21-mediated IRF3 ubiquitination (Fig. 5j), supporting K313 as a major ubiquitination site in this system. Together, these findings indicate that TRIM21 recognizes the DNA-binding domain of IRF3 and promotes its K48-linked ubiquitination at K313, thereby regulating IRF3 stability and transcriptional activity in SCLC.

### XPO1 deficiency induces macrophage-dependent MHC class I upregulation in SCLC cells

**Fig. 6:**
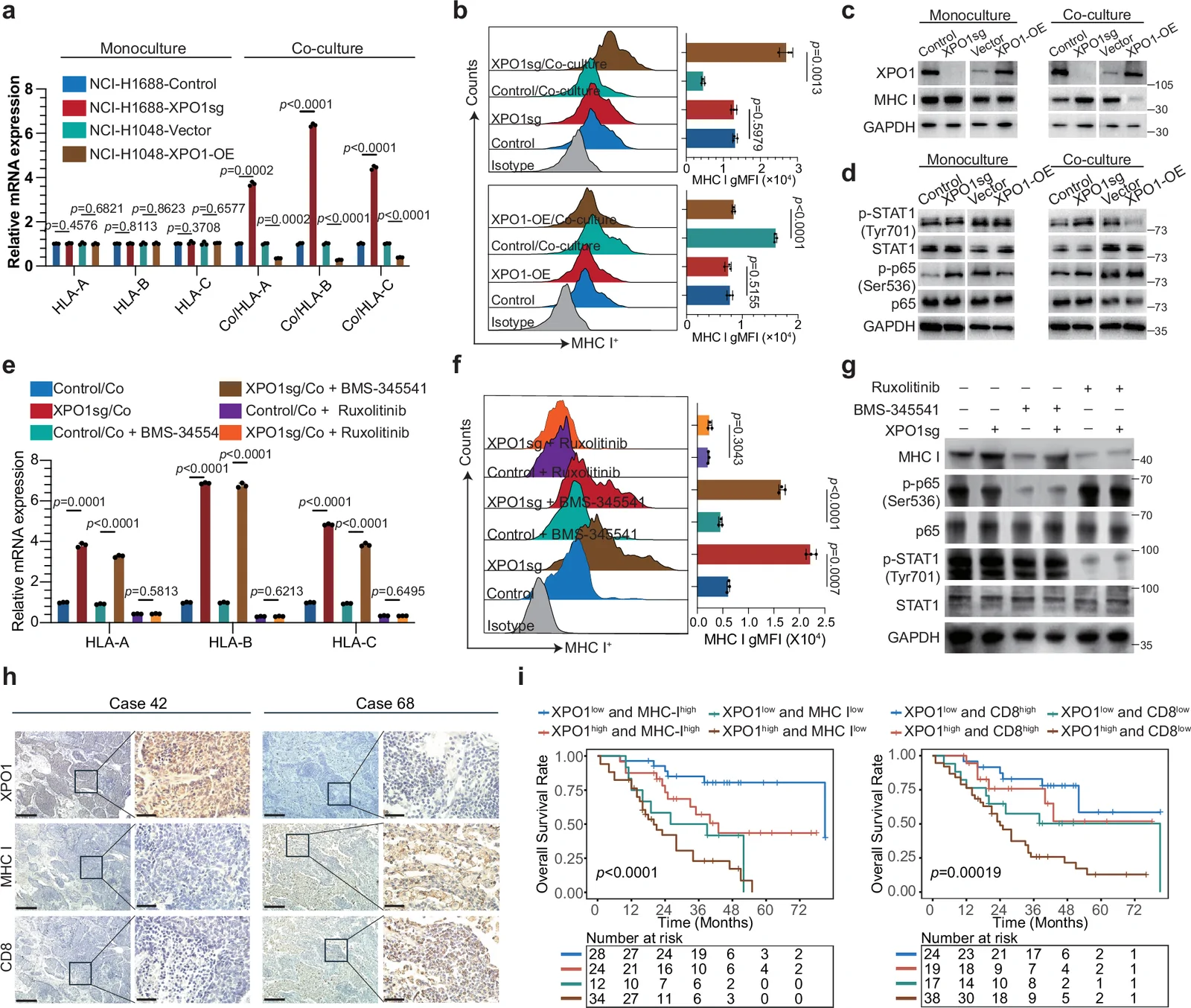
*XPO1*-deficient SCLC cells promote macrophage-dependent antigen presentation in SCLC. RT-qPCR (**a**), flow cytometry (**b**), and immunoblot (**c**) analyses of MHC I expression in the indicated SCLC cell lines cultured alone or co-cultured with THP-1-derived macrophages. Flow cytometry data were quantified by geometric mean fluorescence intensity (gMFI). **d** Immunoblot analysis of total and phosphorylated STAT1 and NF-κB in the indicated SCLC cells. RT-qPCR (**e**), flow cytometry (**f**), and immunoblot (**g**) analyses of MHC I expression in control and NCI-H1688/*XPO1*-silenced cells co-cultured with THP-1-derived macrophages, with or without pretreatment with the JAK1/2 inhibitor ruxolitinib or the IKK inhibitor BMS-345541 for 24 h. Flow cytometry data were quantified by gMFI. **h** Representative immunohistochemistry images of XPO1, CD8, and MHC I staining in human SCLC tumor specimens. Scale bars, 200 μm (left) and 40 μm (right). **i** Kaplan–Meier analysis of overall survival according to XPO1, MHC I, and CD8 expression in the SCLC cohort (*n* = 98 patients). Patients were stratified into low- and high-expression groups according to the optimal cut-off value. Data in (**a**, **b**, **e**, **f**) were analyzed using two-sided unpaired *t*-tests. Data in (**i**) were analyzed using the two-sided log-rank test. Data in (**a**–**g**) are from *n* = 3 biologically independent experiments. Data are presented as mean ± s.d. Source data are provided as a Source Data file.

Analysis of the George et al. dataset showed that *XPO1* expression was inversely correlated with *HLA-A* (*r *= −0.38, *p *< 0.001), *HLA-B* (*r *= −0.33, *p *= 0.003), and *HLA-C* (*r *= −0.33, *p *= 0.002) (Supplementary Fig.11a). Because TREM2^+^ macrophages were associated with antigen processing and presentation (Fig. 1n), we investigated whether macrophages could influence major histocompatibility complex class I (MHC I) expression in SCLC cells. In monoculture systems, neither XPO1 knockdown nor XPO1 overexpression significantly altered MHC I mRNA or protein levels (Fig. 6a–c, Supplementary Fig. 11b, c). In contrast, in macrophage co-culture, XPO1-knockdown SCLC cells showed increased MHC I expression, whereas XPO1-overexpressing SCLC cells showed reduced MHC I expression (Fig. 6a–c, Supplementary Fig. 11b, c), indicating that macrophage-derived cues are required for this regulation. We next evaluated activation of the MHC I transcriptional regulators STAT1 and NF-κB in SCLC cells^30^. In monoculture, XPO1 depletion increased NF-κB phosphorylation without affecting STAT1 activation (Fig. 6d). Under macrophage co-culture conditions, *XPO1* loss was accompanied by increased STAT1 phosphorylation, whereas NF-κB activity did not show a corresponding change (Fig. 6d), suggesting context-dependent signaling. Pretreatment of SCLC cells with the JAK1/2 inhibitor ruxolitinib reduced the MHC class I upregulation induced by *XPO1* loss in co-culture, whereas treatment with the IKK inhibitor BMS-345541 had no comparable effect (Fig. 6e–g). Similarly, siRNA-mediated knockdown of *STAT1* in SCLC cells diminished MHC class I induction after XPO1 loss in co-culture, supporting a tumor-cell-intrinsic role of STAT1 in this response (Supplementary Fig. 11d).

Given that STAT1 is a key mediator of interferon signaling, we next investigated the contribution of IFN-γ to this effect. Neutralizing IFN-γ with an antibody reduced tumor-cell pSTAT1 levels and attenuated the MHC class I increase observed with *XPO1* loss, while p65 phosphorylation remained unchanged (Supplementary Fig. 11e). These findings support a dominant role for IFN-γ-JAK/STAT1 signaling in macrophage-dependent MHC class I upregulation in *XPO1*-deficient SCLC cells in co-culture. Further studies will be necessary to determine the cellular source and temporal dynamics of the interferon signal in this context.

To assess clinical relevance, we performed IHC analysis of 98 resected SCLC specimens. Tumors with low XPO1 expression displayed significantly higher MHC I levels and increased CD8^+^ T-cell infiltration (Fig. 6h and Supplementary Table 1). Patients with concurrent low XPO1 expression and high MHC I or CD8^+^ T-cell levels had longer overall survival (Fig. 6i). Together, these data support a model in which XPO1 restricts antitumor immunity in SCLC by impairing macrophage-dependent antigen presentation and CD8^+^ T-cell recruitment.

### XPO1 inhibition enhances anti-PD-1 activity in humanized and immunocompetent orthotopic models

Limited efficacy of ICI therapy in SCLC is associated with poor immune-cell infiltration, driven in part by defective antigen presentation. Consistent with this concept, SCLC tumor cells exhibited the lowest MHC I expression across the analyzed cohorts, including NSCLC, other solid tumors, and hematological malignancies (Supplementary Fig. 12a). Given the central role of MHC I in antigen presentation and antitumor immune activation (Supplementary Fig. 12b), we hypothesized that XPO1 inhibition could improve immune recognition and sensitize SCLC tumors to PD-1 blockade. Selinexor induced nuclear accumulation of TRIM21 in NCI-H1688 cells, disrupted the TRIM21-IRF3 interaction, attenuated IRF3 ubiquitination and degradation, and increased TNFSF15 expression in a dose-dependent manner (Supplementary Fig. 12c–h). To assess therapeutic efficacy, we established a humanized orthotopic SCLC model by transplanting NCI-H1688 cells into MHC class I and II double-knockout NOD (NCG-MHC I/II dKO) mice reconstituted with human immune cells (Supplementary Fig. 12i). Treatment began seven days after engraftment with anti-PD-1 monotherapy, selinexor monotherapy, or the combination. Combination therapy reduced tumor burden and prolonged survival compared with either monotherapy or control, without overt toxicity as assessed by stable body weight and hematological indices (Supplementary Fig. 12j–o). IHC analysis of tumor tissues showed extensive immune remodeling in the combination group, with increased CD86^+^ macrophage infiltration, decreased TREM2^+^ macrophage infiltration, elevated MHC class I, and increased CD8^+^ T-cell infiltration (Supplementary Fig. 12p, q). These data indicate that XPO1 inhibition promotes antigen presentation and macrophage repolarization and enhances the antitumor activity of anti-PD-1 therapy in this model.

**Fig. 7:**
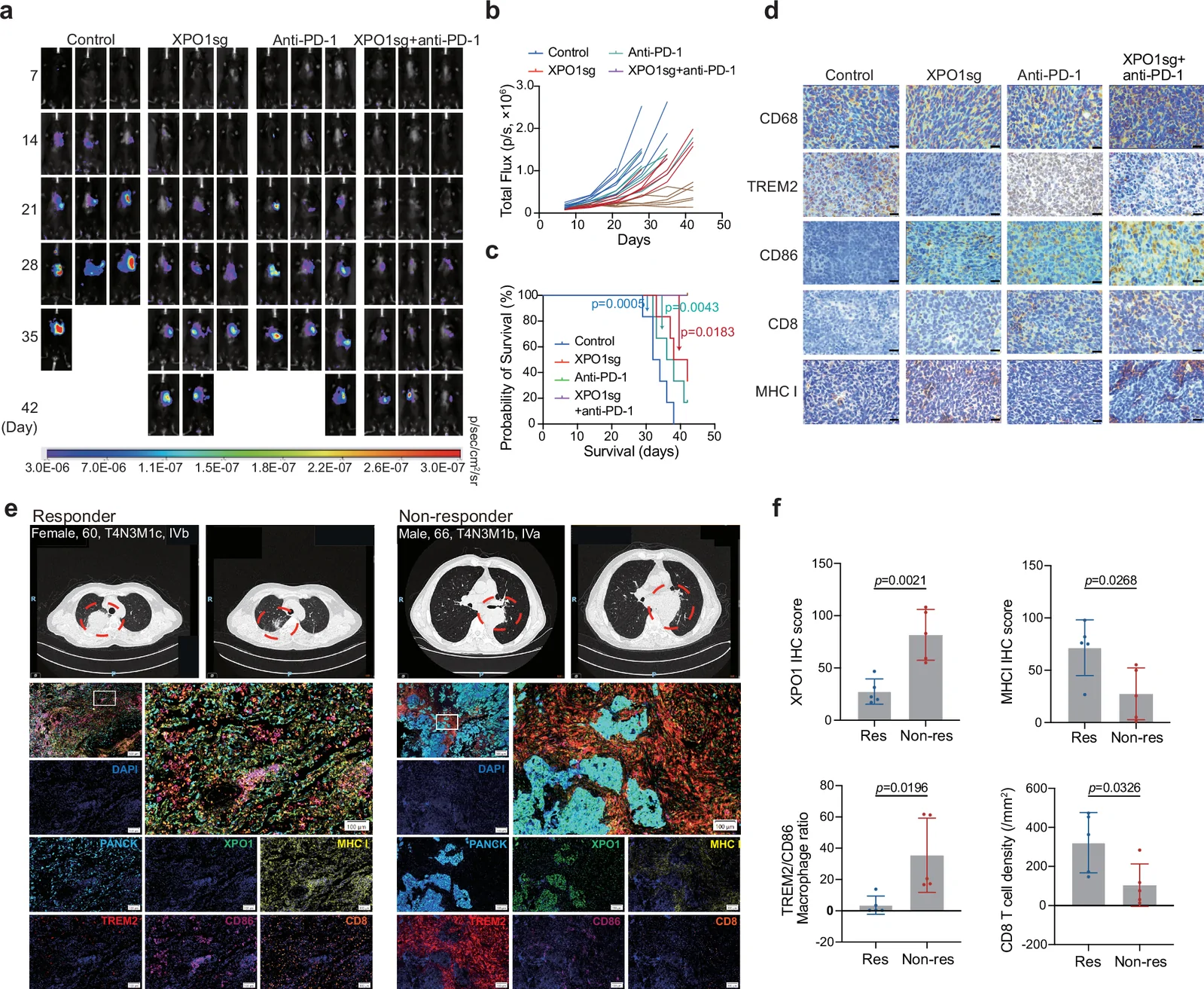
*XPO1* loss sensitizes syngeneic SCLC allograft to anti-PD-1 therapy. **a**, **b** Mice bearing orthotopic allografts of RP116-luc cells transduced with XPO1-silencing or vector-control lentivirus were treated by intraperitoneal injection with anti-PD-1 antibody (200 μg/injection, i.p., q3d) or isotype control antibody. Representative bioluminescence images acquired from day 7 to day 42 (**a**) and longitudinal quantification of bioluminescence signal intensity (**b**) are shown. **c** Kaplan–Meier analysis of survival in mice from the indicated groups. **d** Representative immunohistochemistry images of CD68, TREM2, CD86, CD8, and MHC I staining in allograft SCLC tissues from the indicated groups. Scale bar, 20 μm. **e** Top: representative chest computed tomography (CT) scans from patients with SCLC obtained before and after immune checkpoint inhibitor (ICI) therapy, stratified by clinical response. Bottom: multiplex immunohistochemistry analysis of XPO1, MHC I, TREM2, CD86, and CD8 in tumor samples from ICI responders (*n* = 5) and non-responders (*n* = 5). **f** Quantification of XPO1 and MHC I immunohistochemistry scores, TREM2^+^/CD86^+^ macrophage ratio, and CD8^+^ cell densities in the samples shown in (**e**). Data in (**c**) were compared using the two-sided log-rank test. Data in (**f**) were assessed using two-sided unpaired *t*-test. Data in (**a**–**d**) are from *n* = 6 mice per group. Data are presented as mean ± s.d. Source data are provided as a Source Data file.

To determine whether pharmacological XPO1 inhibition could be recapitulated by genetic XPO1 suppression in an immunocompetent setting, we established syngeneic SCLC allograft tumors in C57BL/6 mice using either control or XPO1-knockdown RP116 cells, followed by treatment with an anti-PD-1 antibody (Fig. 7a). Longitudinal bioluminescence imaging (BLI) showed that XPO1 knockdown enhanced the antitumor effect of PD-1 blockade, resulting in a greater reduction in tumor burden than either XPO1 knockdown alone or anti-PD-1 treatment alone (Fig. 7b). Mice in the combination group also showed the longest survival among the tested groups (Fig. 7c). IHC analysis of endpoint tumors showed comparable CD68^+^ macrophage infiltration across groups, whereas the combination group showed decreased TREM2^+^ macrophage infiltration, increased CD86^+^ macrophage infiltration, enhanced CD8^+^ T-cell infiltration, and elevated MHC class I expression in the combination group (Fig. 7d, Supplementary Fig. 13a). These results provide in vivo evidence that XPO1 contributes to the SCLC immune microenvironment and that XPO1 targeting can improve response to PD-1 blockade.

To explore clinical relevance, we performed multiplex IHC in tumor biopsies from patients with advanced SCLC treated with first-line ICI therapy (Fig. 7e, Supplementary Table 3). Cohort-level analysis, including per-patient quantification of tumor XPO1 and MHC I H-scores and immune-cell densities for TREM2^+^ and CD86^+^ macrophages and CD8^+^ T cells, revealed that responders had significantly lower tumor XPO1 expression (*p *= 0.0021), higher MHC I expression (*p *= 0.0268), a reduced TREM2^+^/CD86^+^ macrophage ratio (*p *= 0.0196), and increased CD8^+^ T-cell infiltration (*p *= 0.0326) compared with non-responders (Fig. 7f). Responders also exhibited a significantly shorter distance between CD8^+^ T cells and tumor cells (*p *= 0.0203, Supplementary Fig. 13b, c). These clinical findings support an association between high XPO1/low MHC I expression, a TREM2-skewed macrophage landscape, and reduced CD8^+^ T-cell presence in non-responders, consistent with a role for XPO1 in an immune-evasive SCLC microenvironment.

## Discussion

SCLC remains one of the most lethal malignancies. Despite decades of effort, therapeutic advances have remained limited: targeted approaches have largely failed to deliver durable survival benefits^31^, whereas the benefits achieved with immunotherapy remain modest, and the gains from immunotherapy are incremental at best^32,33^. ICIs confer only limited survival benefit^9^. Such limited efficacy likely reflects not only the dominance of immune suppression within the tumor ecosystem but also the absence of precision interventions capable of reprogramming it, motivating investigation of the immunomodulatory effects of targeted therapeutic strategies in SCLC.

Our previous work identified XPO1 as a potentially actionable target in SCLC, functionally linked to tumor proliferation and drug resistance^15–17^. Consistent with these findings, Rudin and colleagues showed that XPO1 inhibition enhances SCLC sensitivity to chemotherapy^19^ and suppresses NE transdifferentiation in lung adenocarcinoma and prostate adenocarcinoma^18^. Multiple gastrointestinal NE tumor subtypes also harbor immune microenvironments enriched for immunosuppressive programs^34,35^. In SCLC, the NE phenotype predominates, is defined by lineage transcription factors such as ASCL1 and NEUROD1^20^, and is associated with immunosuppressive features^21–23^. In our study, single-cell transcriptomic analysis showed that selinexor treatment reduced expression of several NE-associated genes, including *Chga*, *Bex1*, and *Syp*, but did not alter the overall NE program. Thus, under the conditions examined here, XPO1 inhibition does not appear to drive a broad NE-to-non-NE lineage transition. Instead, our data suggests that the dominant consequence of XPO1 inhibition is tumor-cell-intrinsic rewiring of immunoregulatory programs that secondarily reshape the surrounding immune microenvironment. This finding does not exclude context-dependent effects of XPO1 inhibition on SCLC lineage plasticity across different subtype backgrounds, treatment durations, or stages of tumor evolution. Within the experimental framework used here, however, the most prominent and reproducible consequence of XPO1 inhibition was depletion of a TREM2^+^ immunoregulatory macrophage population, which we traced mechanistically to tumor-cell-intrinsic regulation of the TRIM21-IRF3-TNFSF15 axis.

**Fig. 8:**
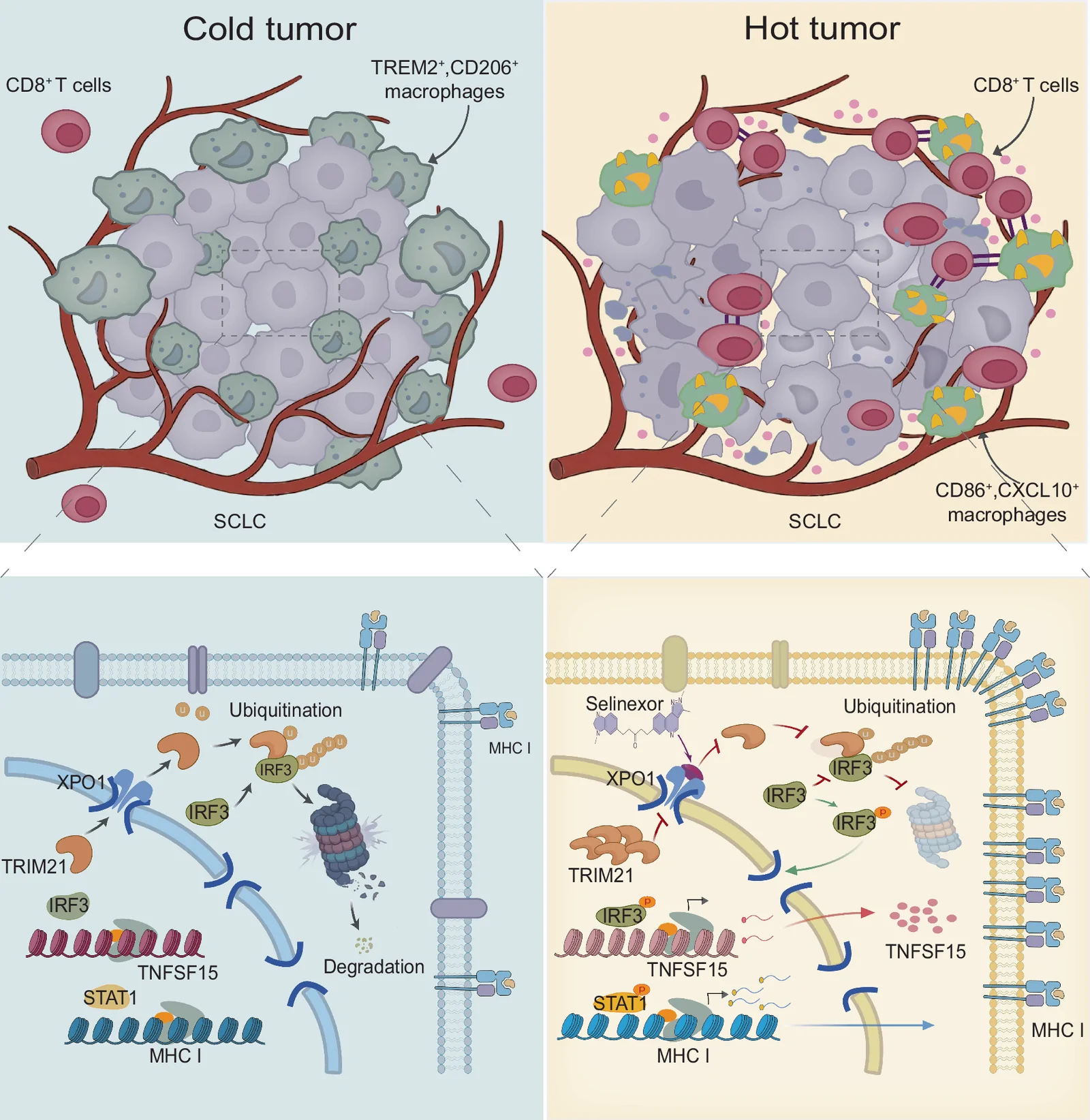
Schematic illustration of the proposed mechanism. XPO1 facilitates the nuclear export of TRIM21, enabling TRIM21-mediated K48-linked ubiquitination and proteasomal degradation of IRF3. This process reduces TNFSF15 expression and contributes to TREM2^+^ macrophage polarization in SCLC. Targeting the XPO1-TRIM21-IRF3 axis may help limit immunosuppressive macrophage reprogramming and enhance anti-PD-1 immunotherapy efficacy in SCLC. This schematic was created by the authors using Adobe Illustrator and contains no third-party material.

Here, we expand the functional scope of XPO1 from a tumor-intrinsic vulnerability to a regulator of the SCLC immune microenvironment. Single-cell transcriptomic analysis showed that pharmacological blockade with selinexor selectively reduced the infiltration of TREM2^+^ macrophages, whereas total macrophage abundance was not changed in murine SCLC allografts. Genetic ablation of XPO1 in vitro and in vivo similarly limited TREM2^+^ macrophage differentiation, restored MHC I expression on tumor cells and improved response to anti-PD-1 therapy. Mechanistically, our data supports a tumor-intrinsic pathway in which XPO1 facilitates TRIM21-mediated nuclear export and proteasomal degradation of IRF3, thereby suppressing TNFSF15. Reduced TNFSF15 favors polarization toward immunosuppressive TREM2^+^ macrophages, which attenuate STAT1 signaling in tumor cells, downregulate MHC I and impair antigen presentation. XPO1 inhibition reverses this cascade by restoring TNFSF15 expression, constraining TREM2^+^ macrophage differentiation and reactivating antitumor T-cell responses (Fig. 8). These findings identify the XPO1-TRIM21-TNFSF15 axis as a molecular pathway that links nuclear export to macrophage-mediated immune evasion and as a candidate therapeutic target for combination with PD-1 blockade.

TNFSF15 can potentiate antitumor immunity by activating antigen-presenting cells and CD8^+^ T cells^36,37^. Activation of the STING pathway can upregulate TNFSF15 in the TME, promote tertiary lymphoid structure formation and enhance pro-inflammatory tumor-infiltrating lymphocyte (TIL) infiltration in melanoma^38^. Zhao et al. showed that TNFSF15 drives myeloid polarization toward an M1-like phenotype via STAT1/3 signaling, thereby suppressing tumor growth in a lung adenocarcinoma mouse model^28^. Our findings extend these observations by linking nuclear export to TNFSF15 repression in SCLC through TRIM21-dependent IRF3 degradation. The identification of TREM2^+^ TAMs as downstream effectors further expands the immunoregulatory scope of this pathway.

TRIM21 has been implicated in cancer biology through ubiquitination of key pro-oncogenic proteins, including NF-κB, p53, and c-Myc, thereby influencing tumor proliferation^39^, epithelial-mesenchymal transition^40^, autophagy induction^41,42^, and immune regulation^43,44^. However, the relationship between TRIM21 and IRF3 remains context dependent. Some studies indicate that TRIM21 interacts with IRF3 through its C-terminal SPRY domain to promote IRF3 protein degradation^45–48^, whereas others suggest that TRIM21 overexpression promotes IRF3 dimerization and increases *IFN-β* transcription during the early stages of viral infection^49–51^. Our findings identify a TRIM21-IRF3 regulatory axis in SCLC, in which TRIM21 targets IRF3 for proteasomal degradation with dependence on the IRF3 DBD and R31 residue. Either deletion of the IRF3 DBD domain or R31 mutation abrogated TRIM21-mediated ubiquitination, leading to TNFSF15 upregulation. Because TRIM21 interacts with ubiquitin-conjugating enzymes in both nuclear and cytoplasmic compartments^52–54^, subcellular localization may determine functional specificity. Our data support XPO1-dependent regulation of TRIM21 nuclear-cytoplasmic localization. However, we did not functionally validate the predicted NES-like motif in TRIM21 by mutagenesis, nor did we compare selinexor with additional canonical nuclear export inhibitors. Thus, while our findings are consistent with TRIM21 being an XPO1 cargo, they do not exclude the possibility that adaptor proteins or other cofactors facilitate this process in cells. Future studies combining NES mutagenesis with orthogonal inhibition of nuclear export will be required to define the export mechanism more rigorously.

Our work also adds to the emerging understanding of TREM2^+^ macrophages, a recently characterized lipid-associated and immunosuppressive myeloid subset reported in diverse pathological contexts^55–58^, including breast cancer metastasis^59^ and hepatocellular carcinoma^60^. In SCLC, we characterized a CD206^+^ & TREM2^+^ macrophage subtype enriched for antigen-presentation-related genes but associated with immune evasion. In vitro and in vivo experiments showed that XPO1 promotes TREM2^+^ macrophage differentiation and that this state is linked to reduced expression of antigen-presentation molecules in tumor cells. These results are consistent with evidence that antigen-presenting macrophages can drive T cells toward terminal exhaustion and promote tumor immune evasion^61^. Targeting TREM2^+^ macrophages has been shown to enhance the efficacy of neoantigen vaccines and ICIs by restoring antitumor T-cell activity^62^. Our results suggest that XPO1 inhibition may provide an approach to modulate this macrophage state in SCLC.

Several limitations should be noted. T-cell cytotoxic function may also be directly or indirectly influenced by XPO1, a possibility that requires further investigation. The impact of XPO1 on macrophage effector functions, including phagocytosis and migration, and the precise mechanisms by which macrophages regulate STAT1 signaling in tumor cells also warrant further mechanistic dissection. Although our data nominate TNFSF15 as an important effector of XPO1 inhibition, additional cytokines induced by *XPO1* loss, such as CXCL1, CCL22, and IL1B, may also contribute and should be evaluated in future studies. Given the marked heterogeneity of SCLC, potential bias arising from cohort size, subtype composition, and prior treatment history may influence the observed associations and limit the generalizability of conclusions drawn from a single clinical cohort.

In conclusion, we identify an immunoregulatory circuit in which XPO1 promotes immune evasion in SCLC by suppressing TNFSF15 through TRIM21-mediated IRF3 degradation, thereby skewing TAMs toward a TREM2^+^ immunosuppressive phenotype. These findings position XPO1 as a regulator of the TREM2^+^ macrophage-associated immune exclusion and support evaluation of selinexor plus PD-1 blockade.

## Methods

### Patients and tissue samples

Between January 2008 and December 2017, patients with SCLC who underwent total lung resection or lobectomy at Tianjin Medical University Cancer Hospital were initially screened. Eligibility criteria included surgery as first-line treatment with no prior antitumor therapy, age 18–75 years, no history of other primary tumors, and complete clinical data. In total, 98 SCLC patients meeting these criteria were included for IHC analysis. Clinicopathological data of SCLC patients, including sex, age, smoking history, pathological diagnosis, and TNM stage, were obtained (Supplementary Table 1). In addition, tumor tissues from 10 patients with histologically confirmed advanced SCLC who received first-line immunotherapy at the Tianjin Cancer Hospital between January 2021 and June 2022 were collected for multiplex IHC (Supplementary Table 3). This study was approved by the ethics committee of the Tianjin Medical University Cancer Institute and Hospital (bc2022279). As this constitutes a retrospective study, the informed consent was waived by the ethics committee of the Tianjin Medical University Cancer Institute and Hospital.

### Cell culture and transfection

Human SCLC cell lines NCI-H1688, NCI-H69, NCI-H446, NCI-H82, NCI-H1048, NCI-H196, SW1271 and DMS273, and the human monocytic leukemia cell line THP-1, were obtained from the Type Culture Collection Committee of the Chinese Academy of Sciences (Shanghai, China). The mouse SCLC cell line RP116^63^ was provided by Kate Sutherland’s group at the University of Melbourne, Australia. It was established from primary lung tumors in a genetically engineered model driven by conditional bi-allelic inactivation of the tumor suppressor genes *Trp53* and *Rb1*. These mice carry conditional alleles for *Rb1* (floxed exon 19)^64^ and *Trp53* (floxed exons 2–10)^65^. Intranasal delivery of an adenoviral to CMV-Cre-recombinase vector to p53Rb mice results in somatic inactivation of *Trp53* and *Rb1* in pulmonary cells and the subsequent development of high-grade NE tumors that are morphologically and phenotypically similar to human SCLC. RP116 cells were cultured in DMEM/F12 medium (Gibco), which was supplemented with 4 μg/mL of Hydrocortisone (Sigma-Aldrich), 5 ng/mL of EGF (Invitrogen), 1% Insulin-Transferrin-Selenium (Life Technologies), and 10% fetal bovine serum (FBS). All cell lines used were free of microbial (including mycoplasma) contamination. Other cells were cultured in DMEM, or RPMI-1640 basic medium supplemented with 10% FBS at 37 °C in a humidified atmosphere of 95% air and 5% CO_2_. Endogenous XPO1 expression was measured across SCLC cell lines. SCLC cells with relatively higher/lower XPO1 expression were selected for subsequent functional assays because of their transfection efficiencies. The expression of XPO1 in the constructed cell lines were shown in Supplementary Fig. 4a. XPO1 was knocked down in cell lines with high endogenous expression (NCI-H1688, NCI-H196, and DMS114), whereas XPO1 was overexpressed in cell lines with low endogenous protein expression (NCI-H1048 and DMS273).

Human full-length *XPO1* (NM_003400), full-length *TRIM21*, the RING, B-BOX, coiled-coil (C-C), and PRY-SPRY domain fragments of *TRIM21*, full-length *IRF3*, the DNA-binding domain (DBD) and transactivation domain (TAD) fragments of *IRF3*, and DBD-deleted *IRF3* were cloned into the pLV expression vector constructed by the PPL (Public Protein/Plasmid Library, China). An empty pLV vector was used as the control. Lentiviral infection was performed according to standard procedures, and puromycin (Solarbio, P8230) was used for selection. For stable knockdown cell lines, shRNA sequences targeting *TNFSF15*, *TRIM21*, and *IRF3* were designed using the Sigma-Aldrich shRNA Designer. Three recommended shRNA sequences for each gene were synthesized and cloned into the pLV-shRNA-bsd vector (Biosettia, USA). Blasticidin (Solarbio, B9300) was used for selection. For stable knockout cell lines, sgRNA sequences targeting *XPO1* were designed and cloned into lentiCRISPR v2 vectors (Addgene). Lentiviral infection was performed according to standard procedures, and puromycin (Solarbio, P8230) was used for selection. To ensure consistent gene editing, single cells were isolated and expanded into monoclonal populations. Knockdown efficiencies were verified by immunoblotting. Scrambled shRNA or non-targeting sgRNA sequences were used as controls. The sequences of sgRNA and shRNA oligonucleotides are provided in Supplementary Table 4.

For cell-based ubiquitylation assays, FLAG-tagged wild-type ubiquitin (FLAG-Ub), FLAG-Ub-K48R, and FLAG-Ub-K63R expression constructs were used. The K48R and K63R constructs are single lysine-to-arginine ubiquitin mutants generated by site-directed mutagenesis. HA-tagged full-length TRIM21 and MYC-tagged full-length IRF3, as well as the indicated IRF3 and TRIM21 mutants were constructed by Miaoling (Wuhan, China). All constructs were verified by Sanger sequencing before use.

### SCLC allograft and xenograft mouse models

Female BALB/c nude mice (GemPharmatech, Cat.D000521), C57BL/6 mice (GemPharmatech, Cat.N000013), and NOD-*Prkdc*^scid^*Il2rg*^null^-(K^b^D^b^)^null^(IA)^null^(NCG-MHC I/II dKO) mice (GemPharmatech, Cat.T050886), were aged 6–8 weeks. All mice were maintained in a temperature-controlled room at 22+/−2 °C with 40–60% relative humidity under a 12 h light/12 h dark cycle. Animals had ad libitum access to water and chow. The exact number of mice used in each experiment are stated in the corresponding figure legends. All animal procedures were approved by the Ethics Committee of Tianjin Medical University Cancer Institute and Hospital (Approval number: AE-2023052) and were conducted in accordance with the NIH Guide for the Care and Use of Laboratory Animals. Following random allocation, animals from the control and experimental groups were co-housed in the same cages under identical environmental conditions. The maximum tumor burden permitted by the institutional animal ethics committee was 2000 mm³ per tumor, and this limit was not exceeded in any animal. Pre-specified humane endpoints for early euthanasia included tumor volume reaching the approved maximum limit, tumor ulceration, necrosis or bleeding, body-weight loss greater than 20% from baseline, impaired mobility, inability to access food or water, or signs of pain or distress, including lethargy, hunching, ruffled fur or labored breathing. Mice were euthanized by CO₂ inhalation followed by cervical dislocation to ensure death, in accordance with protocols approved by the institutional animal ethics committee.

For the subcutaneous tumor model, 1 × 10^6^ RP116 cells were implanted subcutaneously in the right flank of C57BL/6 mice. Tumor growth was monitored at least three times per week using a caliper and calculated by the following formula: Volume = 1/2 L1 × (L2)^2^, where L1 is the long axis and L2 is the short axis of the tumor. Tumors were collected on treatment day 10. Tumor weights were measured and compared between groups. For the orthotopic BALB/c-Nude tumor model, 1 × 10^6^ luciferase-labeled NCI-H1688 or NCI-H1048 cells were implanted. For the humanized xenograft model, 1 × 10^6^ luciferase-expressing NCI-H1688 cells were orthotopically injected into the left lung. For treatment, selinexor (MedChemExpress, HY-17536) was formulated in corn oil and administered by oral gavage at 5 mg/kg once weekly. Anti-PD-1 antibody (Bio X Cell, BE0188) was administered intraperitoneally at 200 μg/injection every 3 days. For the orthotopic C57BL/6 tumor model, 1 × 10^6^ luciferase-expressing RP116 cells were intrapulmonarily injected to establish tumors. Anti-PD-1 antibody (Bio X Cell, BE0146) was administered intraperitoneally at 200 μg/injection every 3 days. On day 7 after tumor implantation, mice were randomized to treatment groups according to BLI signal intensity as the baseline measurement, and weekly again until the experimental endpoint. Tumor tissues were harvested and either processed into single-cell suspensions for flow cytometry or fixed in formalin and embedded in paraffin for immunohistochemical analysis.

### Single-cell RNA-seq library construction and analysis

Single-cell RNA-seq libraries were prepared using the SeekOne DD Single Cell 3’ Library Preparation Kit (SeekGene, Cat. No. K00202) according to the manufacturer’s protocol. Briefly, cells were mixed with reverse-transcription reagents and loaded into the sample wells of SeekOne Chip S3, while barcoded hydrogel beads and partitioning oil were loaded into their corresponding wells. After droplet generation, reverse transcription was carried out at 42 °C for 90 min and terminated at 85 °C for 5 min.The emulsion was then broken, and cDNA was recovered, purified, and PCR-amplified. Amplified cDNA was processed by fragmentation, end repair, A-tailing, and adaptor ligation, followed by indexed PCR to generate sequencing libraries representing the 3’ ends of polyadenylated transcripts and incorporating cell barcodes and unique molecular identifiers (UMIs). Libraries were purified using VAHTS DNA Clean Beads (Vazyme, N411-01), quantified using Qubit (Thermo Fisher Scientific, Q33226), and assessed on a Bio-Fragment Analyzer (Bioptic, Qsep400). Sequencing was performed on an Illumina NovaSeq X Plus platform using PE150 mode.

Cell Ranger was used to convert Illumina base call files to FASTQ files. These FASTQ files were aligned to the mm10 mice reference genome and transcriptome provided by 10X genomics. The gene vs cell count matrix from Cell Ranger (version 5.0.0) was used for downstream analysis. The raw reads were processed using the Cell Ranger pipeline to obtain the UMI. Allograft samples from the same model were pooled together. Cells with fewer than 200 or more than 7500 detected genes were filtered out. Additionally, cells with mitochondrial gene expression levels exceeding 25% were removed from the dataset. After applying these quality control measures, a total of 105,555 single cells from 12 samples were retained for analysis.

Data were normalized using the LogNormalize method in Seurat to reduce the effect of library depth. Highly variable genes were identified using FindVariableFeatures with the variance-stabilizing transformation method. Data were then scaled with ScaleData, and principal component analysis (PCA) was performed with RunPCA. Cell clustering was conducted using FindNeighbors and FindClusters based on the selected principal components. t-distributed stochastic neighbor embedding (t-SNE) was performed using RunTSNE, and the resulting embeddings were used for downstream visualization and analyses.

Cell annotation was conducted based on canonical marker genes. Initially, cells were broadly classified into tumor cells (positive for *Epcam* and *Krt8*), macrophage (positive for *Cd80*, *IL1b* and *Cd86*), T cell (*Cd3d* and *Cd3e*), fibroblasts (positive for *Col1a1* and *Ddr2*), and B cells (positive for *Cd79a* and *Mzb1*). Subsequently, the macrophage and T cell subset was subjected to re-clustering and further annotation, leveraging the expression of canonical marker genes and knowledge of tissue origin to delineate sub-cell types. For the enrichment analysis of each cell type, Gene set enrichment analysis was conducted based on the specifically expressed gene in each cluster identified using FindMarkers algorithm under the criterion of logfc.threshold = 0.25. Top 8 significant pathways were displayed. For the enrichment analysis of the scRNA-seq data, gene sets from the NE-related signature^66^ and the M2 signature were used^67^. Single-cell signature scores were calculated using UCell (version 3.6) to quantify the NE activity of individual tumor cells and the anti-inflammatory activity of individual macrophages.

### Public database analysis

Transcriptomic expression matrices of tumor cell lines were downloaded from the CCLE database (https://sites.broadinstitute.org/ccle). The mRNA expression data and corresponding phenotypic data for 81 SCLC specimens were extracted from the cBioPortal database and preprocessed for subsequent analysis^68^. GEO datasets, including GSE40275^69^ and GSE60052^70^, were downloaded from the GEO database and analyzed. Gene expression data were log2-transformed after normalization. The Tumor Immune Dysfunction and Exclusion (TIDE) algorithm was used to estimate potential benefit from immune checkpoint blockade^71^. Infiltration fractions for 22 immune cell types in each sample were estimated using the CIBERSORT algorithm^72^. Differential cell infiltration between the high and low gene expression groups was compared using the Mann–Whitney U test. Samples with a *P* value < 0.05 were reserved for further analysis.

### IHC and multiplex IHC

In total, IHC staining was performed in paraffin-embedded tissue microarray slides from 98 SCLC patients included in the study. Briefly, paraffin sections were deparaffinized with xylene, rehydrated with graded ethanol, and incubated with 3%H_2_O_2_ in the dark for 20 min to inactivate endogenous peroxidase. Antigen retrieval was done via decloaking chamber heating in antigen repair buffer, followed by overnight primary antibody incubation. After washing, slides were treated with antibody signal enhancer and secondary antibody for 30 min, then conjugated with HRP and Cardassian DAB Chromogen. Two independent observers blindly determined and interpreted the percentage of stained cells. The intensity of staining was divided into four grades: negative scored 0, weak scored 0–1, moderate scored 1–1.5, and strong scored 1.5–3. The H-score of each sample was obtained by multiplying the staining intensity by the area percentage of positive cells (0–100%). According to the H-score, the stained samples were divided into four groups: negative (−; 0), weak (+; 0~1), moderate (++; 1~1.5), and strong (+++; 1.5~3). Samples with a negative or weak H-score were determined to be the low protein expression group, whereas those with a moderate or strong H-score were classified as the high protein expression group.

To assess the spatial organization and contact relationships among tumor cells, macrophages, and CD8^+^ T cells, we performed multiplex IHC using the PANO 7-plex IHC kit (Panovue, China) according to the manufacturer’s instructions. Formalin-fixed paraffin-embedded (FFPE) sections were deparaffinized, rehydrated, and subjected to antigen retrieval. Slides were blocked with antibody diluent/blocking buffer at room temperature for 10 min and incubated overnight at 4 °C with primary antibodies. After washing, sections were incubated with an HRP polymer secondary antibody (anti-mouse/rabbit IgG; Panovue) at room temperature for 10 min, followed by tyramide signal amplification (TSA) fluorophore working solution. Antigens were stained sequentially by repeating cycles of primary antibody incubation, HRP polymer incubation, TSA development, and heat-mediated stripping (microwave treatment) between cycles to remove antibody complexes while preserving the covalently deposited TSA signal. After completion of all staining rounds, nuclei were counterstained with DAPI and slides were mounted. Antibodies and panel information are provided in Supplementary Table 5.

Multispectral images were acquired using the Mantra Quantitative Pathology Imaging System (PerkinElmer, USA) under identical settings across all samples. For each case, regions of interest (ROI) were pre-specified based on tissue quality and tumor content and selected blinded to clinical response. Viable tumor areas were analyzed while excluding necrosis, hemorrhage, tissue folds, and other artifacts. At least four ROIs per case were captured using consistent acquisition parameters across the cohort. Cell segmentation and phenotyping were performed using an automated pipeline in PANO, with nuclei identified by DAPI, tumor cells defined by PAN-CK, and immune cell subsets identified by marker combinations. The densities of TREM2⁺ macrophages, CD86⁺ macrophages, and CD8⁺ T cells, as well as tumor-cell MHC I expression, were quantified. The TREM2/CD86 ratio was calculated for each case. CD8-tumor distance was defined as the mean nearest distance from CD8⁺ T cells to tumor cells. Automated quantification results were further cross-validated by blinded manual review of a subset of images.

### Western blot analysis

Cells were washed twice with PBS, lysed in RIPA buffer with protease inhibitors to extract total protein. Nuclear and cytoplasmic fractions were isolated using a commercial kit (Thermo Fisher Scientific, Cat.78835) according to the manufacturer’s protocol. 20 μg of protein samples were separated on 10% SDS-PAGE gels, transferred to PVDF membranes, and target proteins were detected by Western blotting. Antibodies used are listed in Supplementary Table 5.

### RNA isolation and reverse-transcription quantitative PCR

Total RNA was isolated using the TRIzol reagent according to the manufacturer’s instructions. PCR was performed on a Bio-Rad CFX96 Real-Time PCR System using SYBR Green PCR Mix (Takara, RR820). Primers were synthesised by Tsingke Biotechnology (Beijing, China) and are listed in Supplementary Table 4. Data were normalized to *GAPDH*. Statistical tests and error-bar definitions are reported in the relevant figure legends.

### ELISA

The concentration of TNFSF15 protein in the culture medium of SCLC cells was measured using a Human TNFSF15 ELISA Kit (ab317779; Abcam). The concentration of IL-10 protein in the culture medium of macrophage cells was measured using a Human IL-10 ELISA Kit (Invitrogen; Cat.88-7106-88). The concentration of TGF-β protein in the culture medium of macrophage cells was measured using a Human TGF beta-1 ELISA Kit (Invitrogen; Cat.BMS249-4). The experimental procedures were carried out strictly in accordance with the manufacturer’s guidelines.

### Flow cytometry

To obtain single-cell suspensions, cultured cells were trypsinized and resuspended in PBS. Mouse tumor tissues were minced, digested with collagenase at 37 °C for 1–2 h, and filtered through a 70 μm mesh. Cells were stained with fixable viability dye (BioLegend, 423105), permeabilized with Fixation and Permeabilization Solution (BD Biosciences, 554714), washed thrice with PBS, and incubated with fluorochrome-conjugated antibodies at 4 °C in the dark for 30 min. Flow-cytometry data were acquired using CytoFLEX LX (BECKMAN, USA). Compensation was performed using single-stained controls, and gates were set based on unstained and fluorescence-minus-one controls where appropriate. Data were analyzed using FlowJo v10 software. Antibodies used are summarized in Supplementary Table 5.

### Coculture assays

To establish an in vitro coculture system, SCLC cells and macrophages were seeded in a 24-mm Transwell chamber with a 0.4-μm pore polycarbonate membrane. For THP-1-derived macrophages, THP-1 monocytes were seeded in the lower chamber at 1 × 10⁶ cells/well and differentiated into M0-like macrophage cells with 150 nM phorbol 12-myristate 13-acetate (PMA) for 24 h. Cells were then washed with PBS and cultured in fresh complete medium before coculture.

For PBMC-derived macrophages, human PBMC were isolated from healthy donor blood by Ficoll-Paque density gradient centrifugation. CD14⁺ monocytes were enriched from PBMCs using CD14 MicroBeads (Miltenyi Biotec, Cat.No.130-050-201), according to standard procedures. Briefly, PBMCs were seeded in tissue culture plates and allowed to adhere for 2 h at 37 °C, after which non-adherent cells were removed by gentle washing with PBS. The adherent monocytes were cultured in complete RPMI 1640 medium supplemented with 10% fetal bovine serum, 2 mM L-glutamine, antibiotics, and recombinant human M-CSF (20 ng/mL, Proteintech, Cat.No. 300-25) for five days to generate M0 macrophages. Fresh medium containing M-CSF was replaced every two days. Differentiated PBMC-derived macrophages were washed and used for coculture experiments.

SCLC cells were seeded in the upper chamber at 5 × 10⁵ cells/well. After macrophage differentiation, the upper chamber containing SCLC cells was placed onto the lower chamber to initiate coculture, which was maintained for an additional 24 h. For cytokine rescue experiments, a neutralizing antibody against human TNFSF15 (100 ng/ml, BPS Bioscience, Cat. 101729) or recombinant human TNFSF15 protein (1 μg/ml, MedChemExpress, Cat. HY-P701048) was added to the coculture system. For pathway inhibition experiments, SCLC cells were pretreated with the JAK1/2 inhibitor ruxolitinib (100 nM, MedChemExpress, Cat. HY-50856) or the IKK inhibitor BMS-345541 (1 μM, MedChemExpress, Cat. HY-10519) for 24 h prior to coculture.

For T-cell co-culture experiments, human PBMCs were isolated from healthy donor blood using Ficoll-Paque density-gradient centrifugation. CD8⁺ T cells were purified from PBMCs using a negative selection kit (EasySep Human CD8^+^ T Cell Isolation Kit, StemCell Technologies, Cat. 19258) according to the manufacturer’s instructions, achieving >95% purity. Purified CD8⁺ T cells were pre-activated with low-dose anti-CD3 (0.5 μg/ml, BioLegend, Cat. 317325) and anti-CD28 (0.5 μg/ml, BioLegend, Cat. 302933) antibodies for 6 h in complete RPMI 1640 medium supplemented with 10% fetal bovine serum, 2 mM L-glutamine, and 100 U/ml IL-2. For intracellular IFN-γ detection, a protein transport inhibitor (Brefeldin A, Invitrogen, Cat.00-4506-51) was added during the final 4-6 h of macrophage-CD8⁺ T-cell co-culture.

After 24 h of co-culture with SCLC cells, macrophages were harvested from the lower chamber of Transwell inserts and Fc receptors were blocked using Human TruStain FcX (BioLegend, Cat. 422302) for 10 min at 4 °C. Cells were then incubated with anti-human TREM2 antibody (R&D Systems, Cat. FAB17291A) at 1 μg per 1 × 10^6^ cells for 20 min at 4 °C in the dark. TREM2^+^ cells were enriched with PE MicroBeads (Invitrogen, Cat. 10003D) at 2 μL/1 × 10^6^ cells for 15 min at 4 °C. The flow-through containing the TREM2^-^ fraction was collected as the TREM2^-^ macrophage population. Sorted macrophages were then cultured alone or co-cultured with pre-activated CD8⁺ T cells at a 1:1 ratio in complete RPMI medium for 24 h.

### Immunofluorescence

Cells on glass coverslips were fixed with 4% paraformaldehyde for 15 min, permeabilized with 0.1% Triton X-100 for 10 min, and blocked with 5% fetal bovine serum for 1 h. They were then incubated with primary antibodies at 4 °C overnight in a humidified chamber. Next day, Alexa 488 anti-mouse and Alexa 594 anti-rabbit IgG secondary antibodies (1:1000, Invitrogen, USA) were added. Cell nuclei were stained with 4ʹ,6-diamidino-2-phenylindole for 5 min. Coverslips were mounted on slides with mounting medium and examined under a confocal laser scanning microscope (KEYENCE BZ-X800, Japan). Data were analyzed using ImageJ software. Antibodies used are listed in Supplementary Table 5.

### RNA sequencing and data analysis

Total RNA from SCLC cells/tissues was isolated using TRIzol reagent per standard procedure. After RNA integrity check with Agilent 2100 bioanalyzer, poly-A mRNAs were enriched via oligo (dT)-attached beads and cleaved with Fragmentation Buffer. A cDNA library was built by reverse transcription and PCR amplification of fragmented mRNA, then sequenced on a G400/T7/T10 platform (BGI-Shenzhen, China).

For analysis, differentially expressed genes between the indicated groups were identified using DESeq2 software. Gene Ontology (GO), KEGG, and Reactome pathway enrichment analyses were performed using clusterProfiler package. Gene set enrichment analysis (GSEA) was performed using the “clusterProfiler” package in R. Differentially expressed genes (DEGs) between control and *XPO1* knockdown SCLC cells were listed in Supplementary Data 2.

### ATAC-seq and transcription factor footprinting

A modified Omni-ATAC protocol was performed. Briefly, nuclei from 10^6^ cells were prepared using NE1 buffer (20 mM HEPES pH 8, 10% glycerol, 0.34 M sucrose, 10 mM KCl, 2 mM EDTA, 10 mM sodium butyrate, 1× Roche Complete protease inhibitor, 0.5 mM spermidine). Nuclei were washed once in NE1, then 6.5 × 10^4^ nuclei per replicate were resuspended in tagmentation buffer (25 μL 2× Tagment DNA Buffer (Illumina Cat.20034197), 16.5 μL PBS (filtered), 4.875 μL water, 0.625 μL 8% Tween-20, 0.5 μL 1% digitonin, 2.5 μL TDE1 Tagment DNA Enzyme (Illumina Cat.20034197); 100 nM final Tn5) and incubated at 37 °C for 30 min. Reactions were immediately purified using the QIAquick PCR Purification Kit (Qiagen, Cat.28106) and eluted in 21 μL water. Eluted DNA was amplified for 5 cycles using Nextera i7/i5 primers and NEBNext Ultra II PCR Master Mix (NEB, M0541S) with the following program: 72 °C for 5 min (gap filling), 98 °C for 30 s, then 5 cycles of 98 °C for 10 s and 63 °C for 10 s. The number of additional PCR cycles was determined by qPCR using the KAPA Library Quantification Kit (Roche, KK4854); libraries typically required 7–9 total cycles. Libraries were purified with AMPure XP beads (Beckman Coulter, Cat.A63881) using double-sided size selection (0.6× followed by 1.3×). Samples were pooled and sequenced using 50 bp paired-end reads on an Illumina NextSeq 500.

ATAC-seq peaks were called using MACS2 with standard parameters for ATAC-seq, and a union set of peaks was generated for downstream analyses. De novo and known motif enrichment analyses were performed with HOMER (v4.10.110) using findMotifsGenome.pl (hg38), with motif lengths of 6, 8, 10, and 12. HOMER’s GC- and length-matched background sequences were used as the reference for enrichment testing. For each motif, we report the number of target peaks containing the motif (and the percentage of total peaks), the number/percentage of matched-background sequences containing the motif, and effect sizes quantified as (i) fold-enrichment and (ii) odds ratio (OR) from a 2×2 contingency table, along with 95% confidence intervals computed on the log(OR) scale using the standard error of log(OR). Motif instances were mapped to peaks using HOMER with a motif match threshold of *P *< 1 × 10^−4^. Motif similarity annotations were obtained using TOMTOM against the JASPAR 2022 CORE vertebrates/non-redundant motif database.

To assess whether candidate motifs were preferentially located at peak centers, motif-centered profiles were generated using HOMER annotatePeaks.pl -hist to quantify motif occurrence density as a function of distance to the peak summit/center. In addition, TF occupancy changes were assessed by ATAC-seq footprinting using TOBIAS (v0.17.3). Briefly, Tn5 sequence bias correction was performed with TOBIAS ATACorrect, footprint scores were computed with TOBIAS FootprintScores, and differential binding across conditions was inferred with TOBIAS BINDetect using JASPAR motifs and the union peak set as candidate regulatory regions. The resulting enrichment statistics and footprinting-based differential binding results are reported in Supplementary Data 3 and accompanying figures.

### Prediction and visualization of the TRIM21-IRF3 complex

The canonical amino acid sequences of human TRIM21 and IRF3 were obtained from the UniProt Knowledgebase (UniProtKB; TRIM21, P19474; IRF3, Q14653). The TRIM21-IRF3 complex structure was predicted using the AlphaFold Server, powered by AlphaFold 3, with the full-length amino acid sequences of TRIM21 and IRF3 provided as two protein chains. The predicted complex model was subsequently imported into PyMOL (version 3.0.3) for structural visualization and analysis of the protein-protein interface. Interfacial residues were identified by examining short-range contacts between TRIM21 and IRF3, and atom pairs separated by ≤3.0 Å were displayed as putative intermolecular contacts. Selected contacting residues were shown as sticks, and the corresponding interatomic contacts were indicated by dashed lines. The structural model was used to visualize the predicted TRIM21-IRF3 interaction interface and to identify candidate residues for subsequent mutational analysis.

### Immunoprecipitation and mass spectrometry analysis

Proteins were extracted using Cell Lysis Reagent (Sigma, C2978) supplemented with protease and phosphatase inhibitor cocktail (Sigma, P8340). Cell lysates were collected by centrifugation at 13,000 × *g* for 15 min at 4 °C. Immunoprecipitation was performed using the Dynabeads Protein A Immunoprecipitation Kit (Invitrogen, 10006D) according to the manufacturer’s protocol. Antibodies were added to Dynabeads Protein A, binding to the beads via their Fc-region during incubation. The tube was placed on a magnet to remove supernatant. The bead-bound antibody was used for immunoprecipitation of the target antigen. Antigen-containing samples were added and gently pipetted to resuspend the bead-Ab complex. After incubation and washing, the bead-Ab-Ag complex was eluted with buffer. The precipitant was dissolved in 4× SDS loading buffer and analyzed by Western blotting.

For mass spectrometry, cells were seeded on 10-cm dishes and transfected with Flag-tagged vector or XPO1 plasmid. After 48 h, cells were lysed and immunoprecipitated with an anti-Flag antibody. Successful XPO1 immunoprecipitation was confirmed by silver staining and western blotting. The immunoprecipitant was then analyzed by liquid chromatography-tandem mass spectrometry (LC-MS/MS) for proteomic analysis. For IRF3 ubiquitination mass spectrometry, cells were transfected with the MYC-tagged IRF3 plasmid for 48 h. Cells were lysed in IP buffer supplemented with protease inhibitors, phosphatase inhibitors and N-ethylmaleimide (NEM), and MYC-IRF3 was immunoprecipitated using an anti-MYC antibody. After extensive washing, the immunoprecipitated protein samples were subjected to LC-MS/MS analysis. Raw MS data were processed using Proteome Discoverer and searched against the corresponding UniProt database, with carbamidomethylation of cysteine set as a fixed modification and oxidation of methionine, N-terminal acetylation and diglycine modification on lysine residues as variable modifications. Peptide and protein identifications were filtered at a false discovery rate of <1%. Ubiquitination sites on IRF3 were assigned based on the detection of lysine residues carrying the diglycine remnant. For the ubiquitination assay, cells were transfected with the indicated DNA constructs, and cell lysates were immunoprecipitated with anti-IRF3, followed by western blotting using a primary antibody against FLAG-ubiquitin. The primary antibodies used for western blot and Co-IP assays are summarized in Supplementary Table 5. The detailed information of mass spectrometry was listed in Supplementary Data 1.

### GST pull-down assay

Equal amounts (200 ng/sample) of HA-tagged purified TRIM21 protein were incubated with 100 ng of GST or GST-tagged XPO1 fusion proteins and glutathione agarose beads for 2 h at 4 °C. Subsequently, the beads were washed four times with modified binding buffer and then eluted for Western blotting using the indicated antibodies.

### Chromatin immunoprecipitation assay

ChIP assays were performed using the SimpleChIP Kit (Thermo Scientific, Cat.26157) according to the manufacturer’s protocol. Briefly, ~5 × 10^6^ NCI-H1688, NCI-H1048, and NCI-H196 cells, transfected with IRF3-MYC/WT plasmid for 48 h, were exposed to single or combined drugs for 24 h in the plate and then fixed with formaldehyde. The cross-linked DNA complexes were sheared to 200–1000 base pair fragments and immunoprecipitated with anti-MYC, anti-histone H3 or IgG control antibody as positive and negative control. Immunoprecipitated DNA was then purified and amplified by qPCR using SYBR green. Primers sequences are listed in Supplementary Table 4. Wild-type and mutant TNFSF15 sequences are listed in Supplementary Table 6.

### Dual-luciferase assay

To assess whether IRF3 induces *TNFSF15* transcriptional activity, NCI-H1688 or HEK293T cells were transiently transfected with pLV2-CMV-3xmyc-IRF3 together with pGL3-TNFSF15-WT, pGL3-mut-BS1, or pGL3-mut-BS2 plasmids. After 24 h, promoter activity was measured using the Dual-Luciferase Reporter Assay System (Promega, E1910). Data were normalized to Renilla luciferase activity and expressed as fold induction relative to control vector-transfected cells.

### Data analysis and statistics

Results are presented as mean ± s.d. unless otherwise indicated in the figure legends. Animals were excluded only when objective experimental failure was observed. For molecular analyses, Data were excluded only when a prespecified objective technical failure was documented; no data points were excluded solely on the basis of their numerical value. Experimental groups were matched for age and similar body weight where applicable. Investigators were not blinded unless otherwise stated. For normally distributed data, two-group comparisons were performed using an unpaired *t*-test, and multiple-group comparisons were performed using one-way ANOVA. For non-Gaussian data, Mann–Whitney U tests were used for two-group comparisons, and Kruskal–Wallis tests followed by Dunn post hoc tests were used for multiple comparisons. Pearson’s correlation coefficients and stepwise multiple linear regression analysis were used to assess associations between variables. *P *< 0.05 was considered statistically significant. Analyses were performed using GraphPad Prism Software version 9.5 (GraphPad, San Diego, CA).

Additional methodologies are detailed in Online Supplementary Methods and Tables (Primers for qPCR and sgRNA/shRNA oligos for stable knockdown cells in Supplementary Table 4; antibodies in Supplementary Table 5).

## Supplementary information

Supplementary Information

Transparent Peer Review file

Description of Additional Supplementary Files

Supplementary Data 1

Supplementary Data 2

Supplementary Data 3

## Source data

Source Data

## Data Availability

The raw sequence data of single-cell RNA-seq have been deposited in the Genome Sequence Archive (GSA) under accession code CRA029897. The raw sequence data of ATAC-seq have been deposited in the GSA-Human under accession code HRA013141. The mass spectrometry proteomics data have been deposited to the ProteomeXchange Consortium with identifier PXD068201 (https://www.iprox.cn/page/project.html?id=IPX0013324000). Previously published datasets analyzed in this study are publicly available from the Gene Expression Omnibus under accession codes GSE40275 and GSE60052. Transcriptomic data from the George et al. cohort were obtained from cBioPortal (https://www.cbioportal.org/study/summary?id=sclc_ucologne_2015). Cancer Cell Line Encyclopedia (CCLE) data were obtained from the DepMap portal (https://depmap.org/portal/data_page/?tab=allData; 2019 release). All data are included in the Supplementary Information or available from the authors, as are unique reagents used in this Article. The raw numbers for charts and graphs are available in the Source Data file whenever possible. Source data are provided with this paper.
