# Supplementary material for: XPO1-mediated TRIM21 nuclear export reprograms TREM2+ macrophage polarization by targeting IRF3 to augment anti-PD-1 efficacy in small cell lung cancer
Source: Nat Commun. 2026 Aug 22;17:10050. doi: 10.1038/s41467-026-76862-0 (PMC13594258; doi:10.1038/s41467-026-76862-0)
Supplement: Supplementary file 1 [file 41467_2026_76862_MOESM1_ESM.pdf]

Supplementary Information

XPO1-mediated TRIM21 nuclear export reprograms TREM2+ macrophage polarization by targeting IRF3 to augment anti-PD-1 efficacy in small cell lung cancer

Qingwu Du[1,2,†], Tingting Qin[1,†], Jingya Wang[1,†], Yantao Jiang[1], Qi Xu[1], Xueyang Li[1], Huiyan Liu[1], Junjie Yu[1], Yi Lu[1], Luyao Tong[1], Tao Sun[3], Peng Chen[1,*], Richeng Jiang[1,*], Dingzhi Huang[1,*]

[1] Tianjin Medical University Cancer Institute and Hospital, National Clinical Research Center for Cancer; Key Laboratory of Cancer Prevention and Therapy, Tianjin; Tianjin's Clinical Research Center for Cancer; Department of Thoracic Oncology, Tianjin Lung Cancer Center, Tianjin Cancer Institute & Hospital, Tianjin Medical University, Tianjin, 300060, PR China;

[2] Department of Internal Medicine, The Affiliated Cancer Hospital of Zhengzhou University & Henan Cancer Hospital, Zhengzhou, People's Republic of China; Institute of Cancer Research, Henan Academy of Innovations in Medical Science, Zhengzhou, 450008, China

[3] State Key Laboratory of Medicinal Chemical Biology and College of Pharmacy, Nankai University, Tianjin, 300450, China.

[†]Contributed equally.

*Corresponding author.

*To whom correspondence may be addressed. E-mail: chenpeng@tjmuch.com, jiangricheng@tjmuch.com, huangdingzhi@tjmuch.com.

Department of Thoracic Oncology, Tianjin Lung Cancer Center, Tianjin Cancer Institute & Hospital, Tianjin Medical University, Tianjin 300060, China; Ph: (022) 23340123 extension 3203; Fax: (022) 23537796;

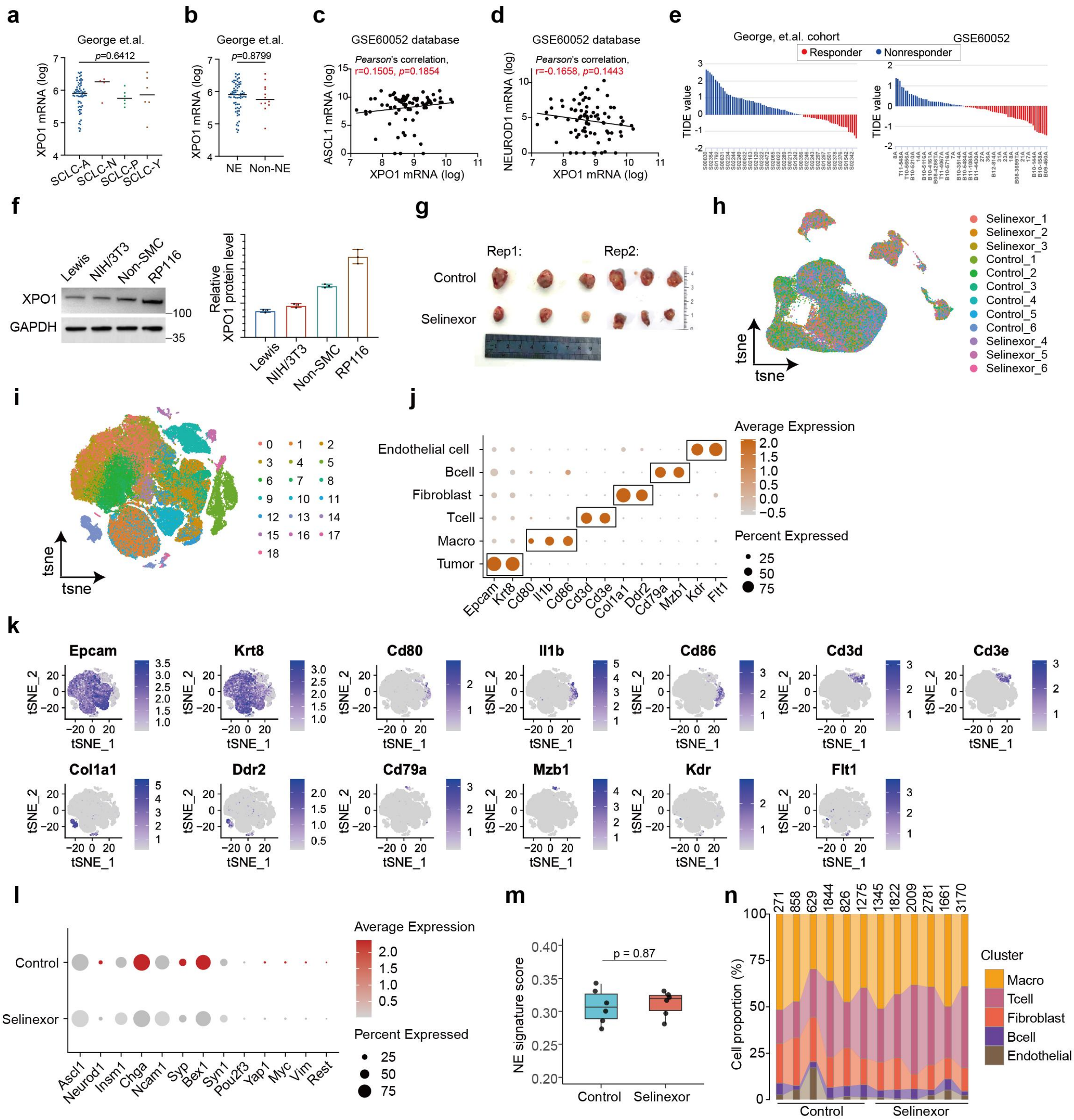

Supplementary Fig. 1. XPO1 expression patterns, immune features and single-cell landscape in SCLC. a *XPO1* mRNA expression across different SCLC molecular subtypes, including SCLC-A, SCLC-N, SCLC-P, and SCLC-Y, in the George et al. cohort. **b** XPO1 mRNA expression in neuroendocrine (NE) and non-neuroendocrine (Non-NE) tumours in the George et al. cohort (n = 81 samples in total). **c,d** Pearson correlation analysis of *XPO1* mRNA expression with the canonical neuroendocrine lineage factors *ASCL1* (**c**) and *NEUROD1* (**d**) in GSE60052 (n = 79 samples in total). **e** Distribution of Tumor Immune Dysfunction and Exclusion (TIDE) scores in patients from the George et al. cohort (n = 81) and the GSE60052 dataset (n = 79). TIDE scores were calculated using the TIDE web server. **f** Endogenous XPO1 protein expression in Lewis, non-SMC, and RP116 cells, with NIH/3T3 cells included as a positive control, as determined by immunoblotting. ImageJ-based densitometric quantification of XPO1 normalized to GAPDH is shown on the right. **g** Representative gross images of SCLC tumours collected

at the experimental endpoint. **h** Distribution of cells from 12 tumour samples after batch-effect correction using the Harmony algorithm, colour-coded by tumour sample. **i** t-SNE plot of single-cell RNA-seq data showing 18 transcriptionally distinct cell clusters. Each dot represents one cell, colored by cluster identity. Cluster labels are shown on the plot. **j** Dot plot showing the expression of canonical marker genes in each cell type. **k** Feature plots showing the expression of marker genes across different cell populations. **l** Dot plot showing the expression of canonical neuroendocrine and non-neuroendocrine lineage markers in tumour cells from control and Selinexor-treated SCLC allograft tumors. Dot size indicates the percentage of cells expressing each gene, and colour indicates the average expression level. Neuroendocrine-associated markers include *Ascl1*, *Neurod1*, *Chga*, *Ncam1*, *Syp*, *Bex1*, and *Syt13*, whereas canonical non-neuroendocrine markers include *Pou2f3*, *Yap1*, *Myc*, and *Rest*. **m** Comparison of NE signature scores in tumour cells from control and Selinexor-treated SCLC allograft tumours. Signature scores were calculated using the UCell algorithm based on a curated set of NE-associated genes[1]. **n** Proportions of each cell type in the control and Selinexor-treated groups. Data in **a** were analysed by one-way ANOVA. Data in **b,m** were analysed using a two-sided unpaired t-test. Data in **c,d** were analysed using Pearson's correlation analysis. Data in **f** are from n = 3 biologically independent experiments. Data in **g-m** are from n = 6 mice per group. Data are presented as mean ± s.d. Source data are provided as a Source Data file.

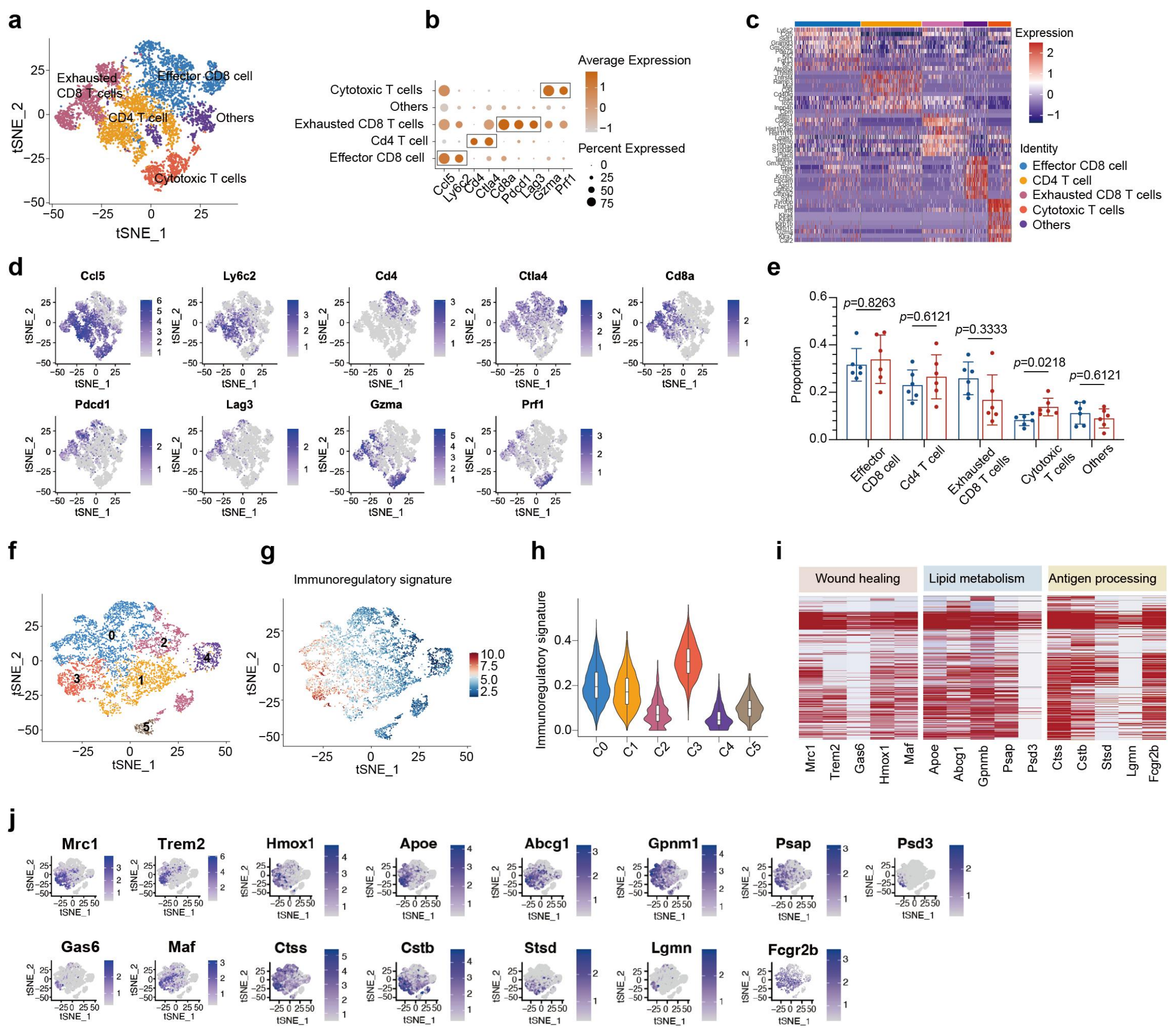

Supplementary Fig. 2. Single-cell characterization of tumor-infiltrating T cells and macrophages in control- and Selinexor-treated SCLC tumors. **a** t-SNE projection of T cells from all tumours, identifying five major T-cell clusters. **b** Dot plot showing the expression of canonical marker genes across the T-cell clusters. **c** Heatmap showing the top 10 differentially expressed genes for each T-cell cluster. **d** Feature plots showing the expression of marker genes across the identified T-cell subtypes. **e** Proportions of each T-cell subtype in control- and Selinexor-treated tumours. **f** t-SNE projection of macrophages from all tumours, identifying six major macrophage clusters. **g** t-SNE projection of macrophages coloured by immunoregulatory signature score, signature scores were calculated using the UCell algorithm based on a curated set of M2-like macrophage genes[2]. **h** Violin plots showing the immunoregulatory signature scores across macrophage clusters in **g**. **i** Expression of selected genes enriched in macrophage cluster 3 and associated with wound healing, lipid metabolism, and antigen processing. **j** Feature plots showing the expression of the genes highlighted in **i** across macrophage clusters. Data in **e** are from n = 6 mice per group and are presented as mean ± s.d. Data in **e** were analysed using multiple Mann-Whitney tests with false discovery rate correction. Source data are provided as a Source Data file.

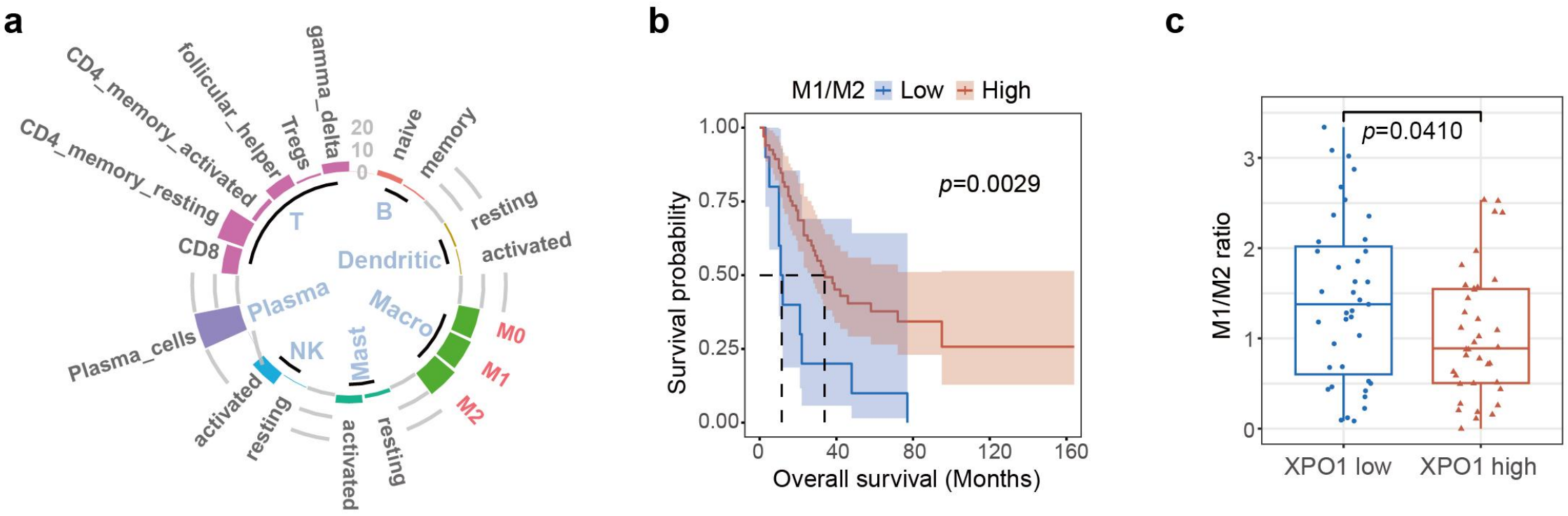

Supplementary Fig. 3 Immune infiltration analysis in SCLC. a Proportion of immune cell subtypes infiltrating the tumour microenvironment (TME) in SCLC. Immune cell proportions were estimated using the CIBERSORT algorithm applied to transcriptomic data from the George et al. cohort (n = 81). **b** Association between the M1/M2 macrophage ratio and overall survival in the George et al. cohort (n = 81). **c** Correlation between *XPO1* expression and the M1/M2 ratio in the George et al. cohort (n = 81). Patients were classified into low- and high-*XPO1* groups based on the median *XPO1* expression value. Data in **b** were compared using the two-sided log-rank test. Data in **c** were analysed using a two-sided unpaired t-test. Data are presented as mean ± s.d. Source data are provided as a Source Data file.

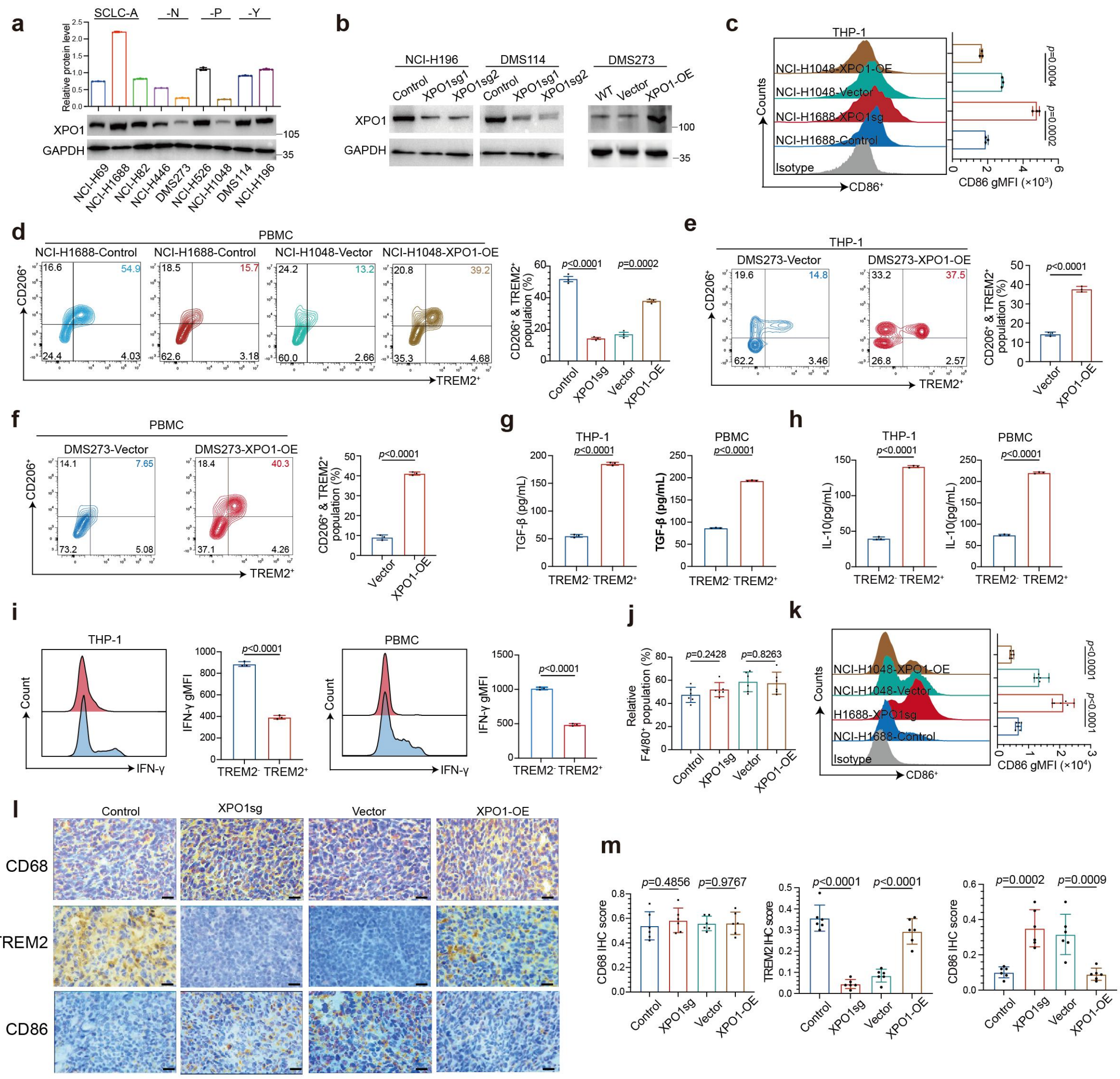

Supplementary Fig. 4 XPO1 drives macrophage polarization toward a TREM2⁺ phenotype in SCLC. a Endogenous XPO1 protein expression in SCLC cell lines, as determined by immunoblotting (bottom), with ImageJ-based quantification shown above (top). **b** Immunoblot analysis of XPO1 expression in SCLC cell lines transduced with lentiviral constructs expressing either *XPO1*-specific sgRNA (XPO1sg) or *XPO1* overexpression (XPO1-OE) constructs. **c** Flow cytometric analysis of CD86 cell surface expression on macrophages co-cultured with SCLC cells. Representative flow cytometry plots and quantification of the geometric mean fluorescence intensity (gMFI). **d** Flow cytometric analysis of TREM2 and CD206 surface expression on PBMC-derived macrophages co-cultured with the indicated SCLC cell lines. **e** Flow cytometric analysis of TREM2 and CD206 surface expression on THP-1-derived macrophages co-cultured with the indicated DMS273 cells. **f** Flow cytometric analysis of TREM2 and CD206 surface expression on PBMC-derived macrophages co-cultured with DMS273 cells. **g,h** ELISA analysis of IL-10 (**g**) and TGF-β (**h**) secretion by THP-1-derived macrophages (left)

and PBMC-derived macrophages (right) after co-culture with the indicated SCLC cells. Culture supernatants were collected from sorted TREM2$^+$ and TREM2$^-$ macrophage populations after 48 h of monoculture following cell sorting. **i** Flow cytometric analysis of IFN-γ expression in CD8$^+$ T cells after co-culture with sorted TREM2$^+$ or TREM2$^-$ macrophages. **j** Quantification of the percentage of F4/80$^+$ macrophages within the CD45$^+$ immune cell population from single-cell suspensions of tumour digests derived from mouse models. **k** Flow cytometry analysis showing the proportion of CD86$^+$ macrophages within the F4/80$^+$ macrophage population in the indicated SCLC xenograft tumors. **l** Representative immunohistochemistry images of CD68, TREM2, and CD86 staining in xenograft SCLC tissues from the indicated groups at the experimental endpoint. Scale bar, 20 µm. **m** Quantification of the staining shown in **l**. Data in **c-k,m** were analysed using two-sided unpaired t-tests. Data in **a-i** are from n = 3 biologically independent experiments. Data in **j-m** are from n = 6 mice per group. Data are presented as mean ± s.d. Source data are provided as a Source Data file.

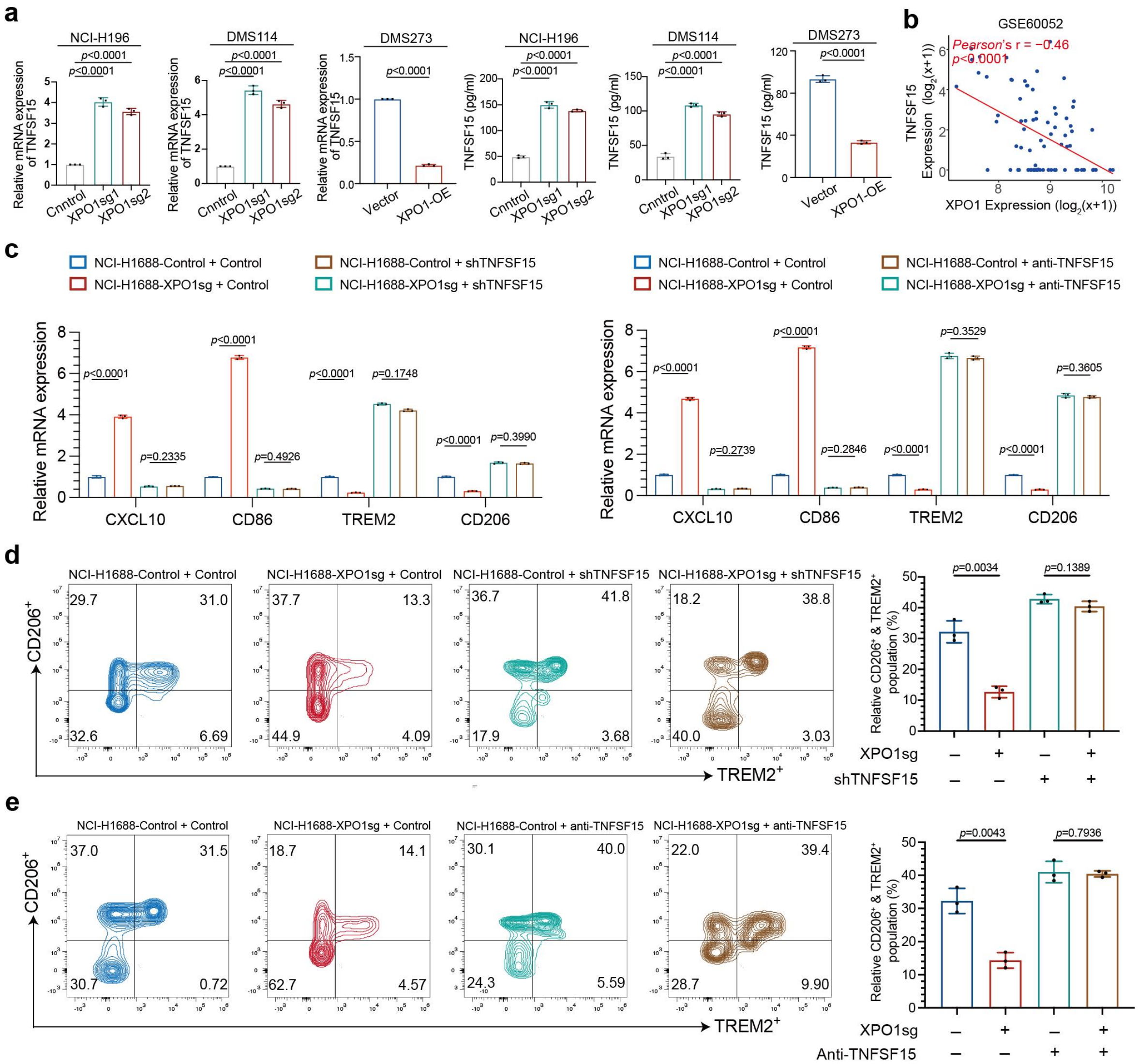

Supplementary Fig. 5 TNFSF15 is required for XPO1 knockdown-mediated suppression of TREM2+ macrophage polarization in SCLC. a RT-qPCR and ELISA analyses of *TNFSF15* mRNA expression and secreted TNFSF15 protein levels, respectively, in the indicated SCLC cell lines. **b** Pearson correlation analysis between *XPO1* and *TNFSF15* mRNA expression in the GSE60052 dataset (log2(x + 1), n = 79 samples). **c** RT-qPCR analysis of *CD86*, *CXCL10*, *TREM2*, and *CD206* expression in macrophages co-cultured with NCI-H1688-XPO1sg cells after *TNFSF15* knockdown by lentiviral shRNA (left) or treatment with a TNFSF15-neutralizing antibody (right). **d,e** Flow cytometric analysis of the proportion of CD206+ & TREM2+ macrophages co-cultured with NCI-H1688-XPO1sg cells following *TNFSF15* knockdown (**d**) or TNFSF15 neutralization (**e**). Data in **a** were analysed by one-way ANOVA. Data in **b** were analysed using Pearson correlation analysis. Data in **c,d,e** were also compared using two-sided unpaired t-tests. Data are from n = 3 biologically independent experiments and are presented as mean ± s.d. Source data are provided as a Source Data file.

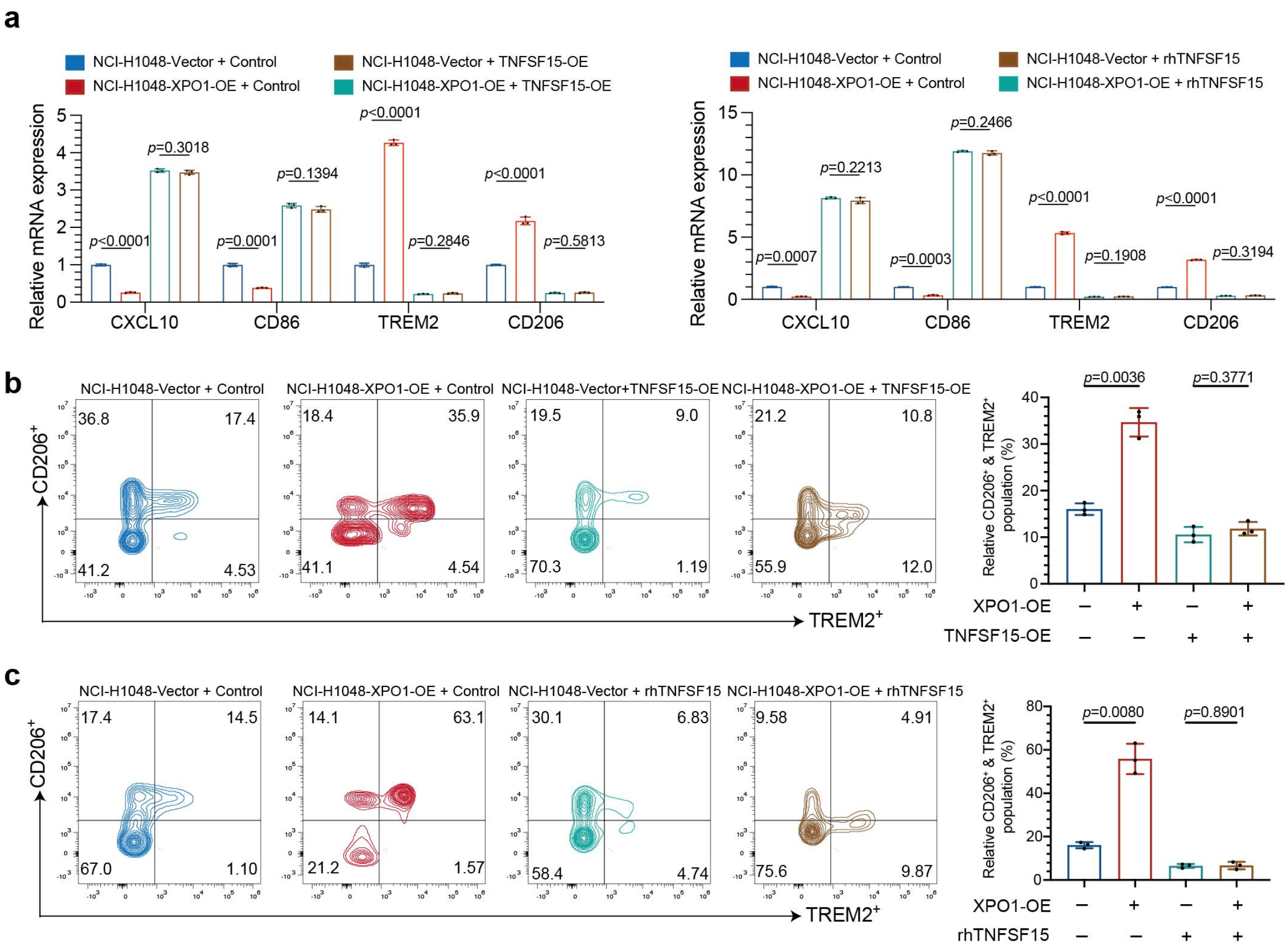

Supplementary Fig. 6 TNFSF15 supplementation attenuates XPO1-driven TREM2$^+$ macrophage polarization in SCLC. a RT-qPCR analysis of *CD86*, *CXCL10*, *TREM2*, and *CD206* expression in macrophages co-cultured with NCI-H1048-XPO1-OE cells following *TNFSF15* overexpression by lentiviral transduction (left) or treatment with recombinant human TNFSF15 (rhTNFSF15) (right). **b,c** Flow cytometric analysis of the proportion of CD206$^+$ & TREM2$^+$ macrophages co-cultured with NCI-H1048-XPO1-OE cells following *TNFSF15* overexpression by lentiviral transduction (**b**) or treatment with recombinant human TNFSF15 (rhTNFSF15) (**c**). Data in **a,b,c** were compared using two-sided unpaired t-tests. Data are from n = 3 biologically independent experiments and are presented as mean ± s.d. Source data are provided as a Source Data file.

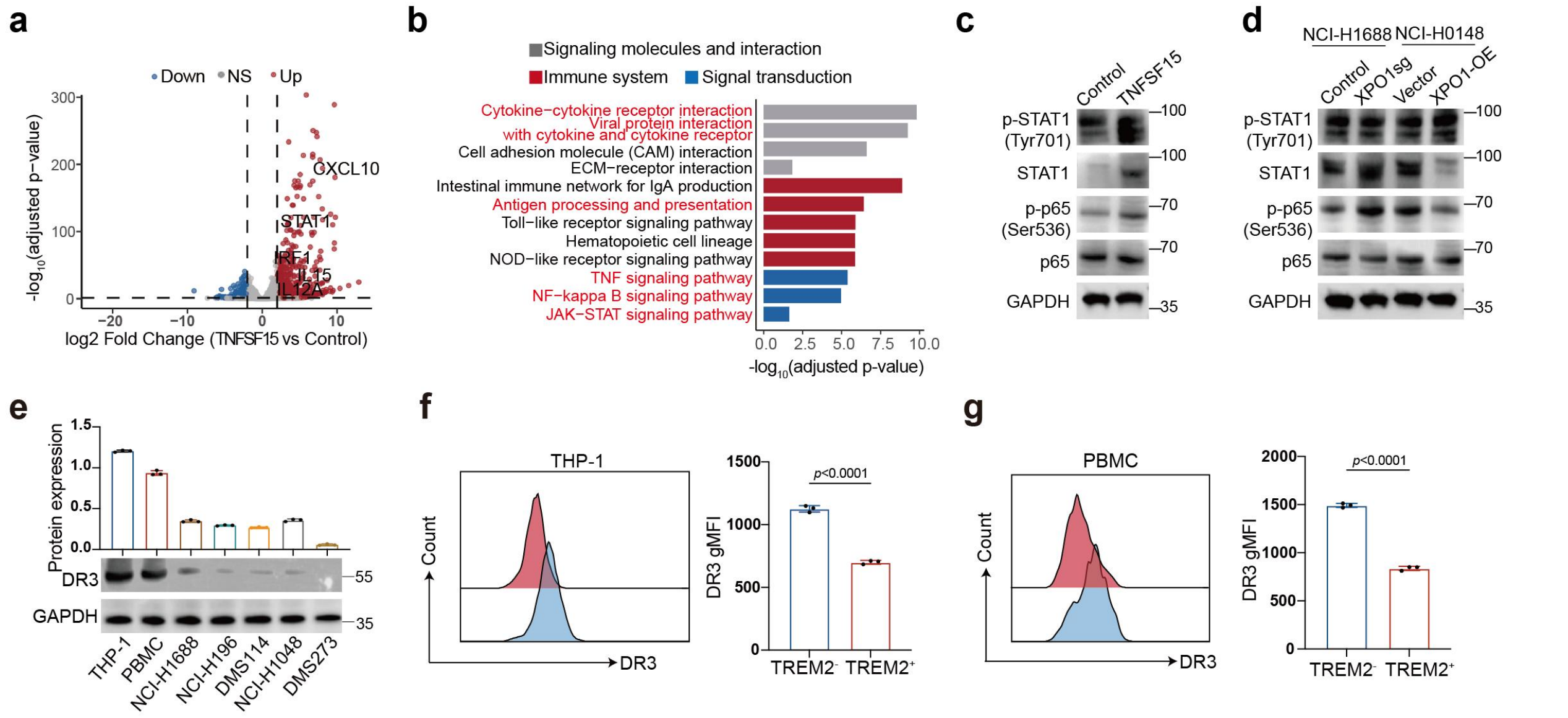

Supplementary Fig. 7 TNFSF15-induced transcriptional changes and downstream STAT1/NF-κB signaling in macrophages. a Differential gene expression analysis between control and recombinant TNFSF15-treated THP-1-derived macrophages (1μg/ml, 24 h). Differential expression analysis was performed using edgeR, with |log2(fold change)| > 2 and adjusted P < 0.05 as the thresholds. **b** KEGG pathway enrichment analysis of the 1,780 upregulated genes shown in panel **a**. Pathways are ranked by −log10(adjusted p value). **c** Immunoblot analysis of total STAT1, phospho-STAT1 (Tyr701), total NF-κB p65, and phospho-NF-κB p65 (Ser536) in THP-1-derived M0 macrophages treated with recombinant TNFSF15 (1μg/ml, 24 h) or vehicle control. **d** Immunoblot analysis of total STAT1, phospho-STAT1 (Tyr701), total NF-κB p65, and phospho-NF-κB p65 (Ser536) in THP-1-derived M0 macrophages co-cultured with NCI-H1688-XPO1sg or NCI-H1048-XPO1-OE SCLC cells. **e** Immunoblot analysis of endogenous DR3 expression across THP-1-derived macrophages, PBMC-derived macrophages, and SCLC cell lines. **f,g** Flow cytometric analysis of DR3 expression in TREM2^{-} and TREM2^{+} macrophages derived from THP-1 cells (**f**) or PBMCs (**g**) after co-culture with the indicated SCLC cells. Representative flow cytometry plots are shown on the left, and quantification of the geometric mean fluorescence intensity (gMFI) is shown on the right. Data in **f,g** were analysed using two-sided unpaired t-tests. Data are from n = 3 biologically independent experiments and are presented as mean ± s.d. Source data are provided as a Source Data file.

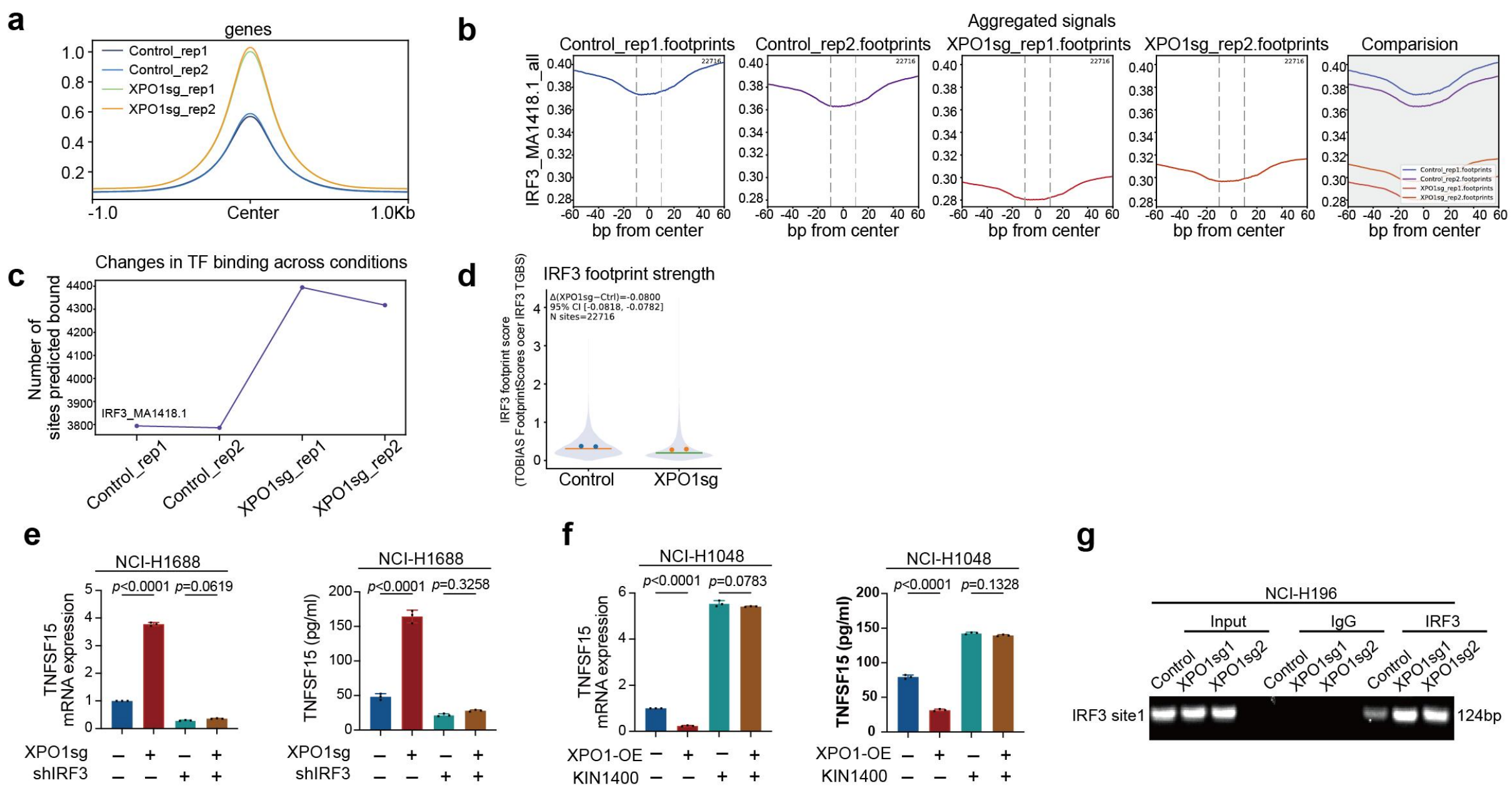

Supplementary Fig. 8 *XPO1* loss enhances IRF3 occupancy and promotes IRF3-dependent *TNFSF15* transcription in SCLC cells. a Aggregate ATAC-seq accessibility metaplot across the analyzed peak set in control and *XPO1*-deficient samples, showing central enrichment of accessibility and increased peak-center signal in *XPO1*-deficient samples. **b** Bias-corrected genome-wide ATAC footprinting profiles generated using TOBIAS, shown for individual biological replicates and aggregated signals, comparing control and *XPO1*-deficient samples. **c** Number of predicted IRF3-bound sites across biological replicates in control and *XPO1*-deficient samples. **d** IRF3 footprint scores at predicted IRF3 transcription factor-binding sites (TFBSs) in control and *XPO1*-deficient samples. *XPO1* deficiency induces a shift in footprint score ($\Delta = -0.0800$; negative values indicate increased occupancy), consistent with enhanced IRF3 engagement and transcriptional activation of downstream targets including *TNFSF15*. **e** RT-qPCR (left) and ELISA (right) analyses of *TNFSF15* mRNA expression and secreted TNFSF15 protein levels, respectively, in NCI-H1688 cells transfected with *XPO1* sgRNA, shIRF3, or both. **f** RT-qPCR (left) and ELISA (right) analyses of *TNFSF15* mRNA expression and secreted TNFSF15 protein levels, respectively, in NCI-H1048-XPO1-OE cells treated with the IRF3 agonist KIN1400. **g** ChIP-PCR assays showing IRF3 binding to the *TNFSF15* promoter in *XPO1*-knockdown NCI-H196 cells. Data in **e,f** were analysed by one-way ANOVA. Data in **e,f,g** are from n = 3 biologically independent experiments. Data in **e,f** are presented as mean ± s.d. Source data are provided as a Source Data file.

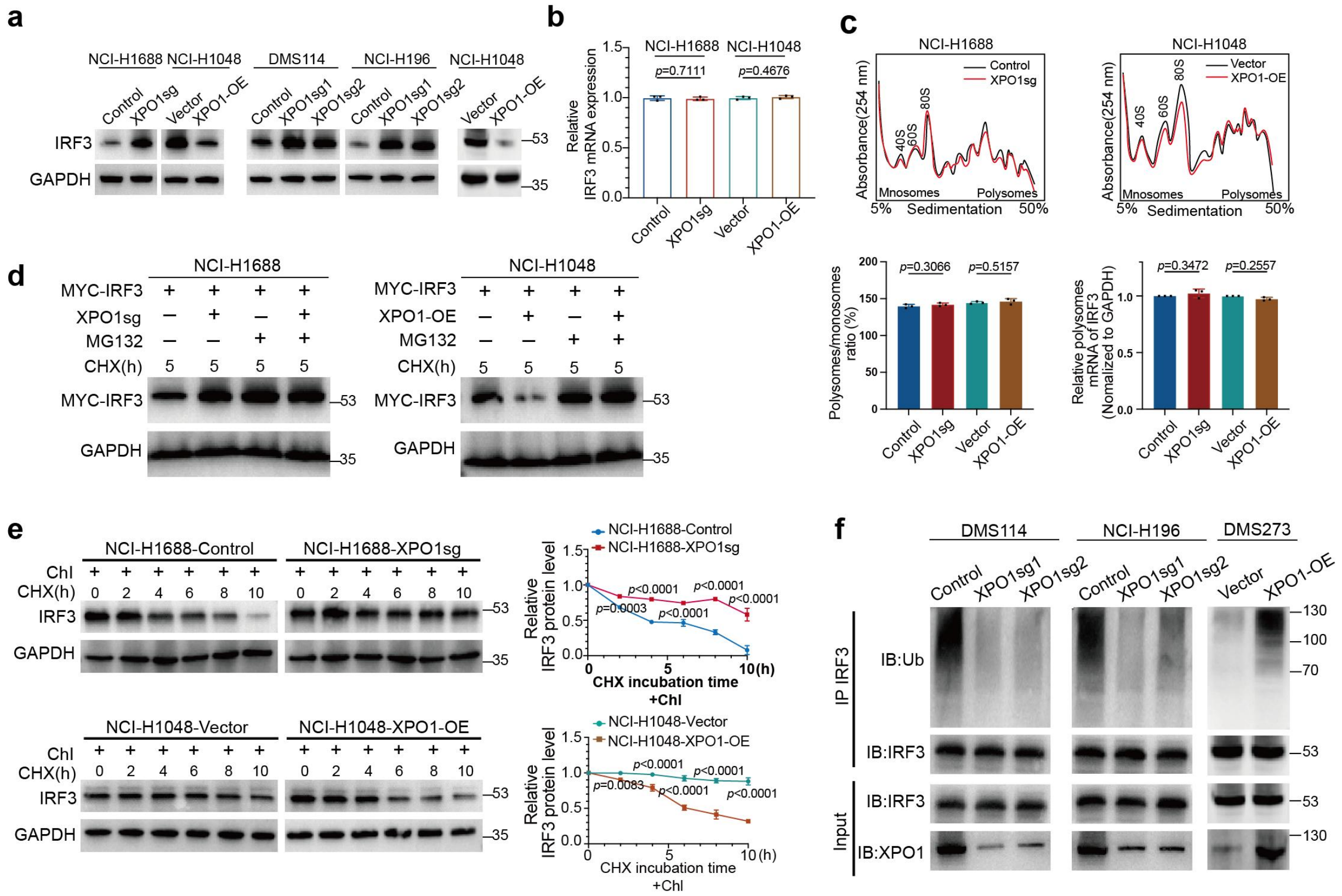

Supplementary Fig. 9 XPO1 suppresses IRF3 protein expression by promoting its degradation in SCLC cells. **a** Immunoblot analysis of IRF3 protein levels in SCLC cells with *XPO1* knockdown or overexpression. **b** RT-qPCR analysis of *IRF3* mRNA levels in SCLC cells with *XPO1* knockdown or overexpression. **c** Polysome profiling analysis in NCI-H1688-control/XPO1sg and NCI-H1048-vector/XPO1-OE cells. Cytoplasmic extracts were separated on 5-50% sucrose gradients and UV absorbance at 254 nm was continuously monitored. The positions of the 40S, 60S, and 80S ribosomal subunits and monosomes are indicated (top). Quantification of the polysome-to-monosome ratio is shown below. **d** Immunoblot analysis of MYC-IRF3 protein stability in the indicated cell lines. Cells were transfected with MYC-IRF3, treated with MG132 for 5 h, and then incubated with cycloheximide (CHX) plus MG132 for 5 h before immunoblotting with the indicated antibodies. **e** Immunoblot analysis of IRF3 protein stability in the indicated cell lines after treatment with chloroquine (Chl) for 12 h followed by CHX chase for the indicated times. Densitometric quantification of IRF3 protein levels and the corresponding decay curves are shown. **f** Ubiquitylation assay in *XPO1*-knockdown SCLC cells. Cells were treated with MG132 for 5 h, followed by immunoprecipitation with an anti-IRF3 antibody and immunoblotting with an anti-ubiquitin antibody to detect polyubiquitylated IRF3. Representative images are shown. Data in **b,c** were analysed using a two-sided unpaired t-test. Data in **e** were analysed by two-way ANOVA followed by Sidak's multiple-comparisons test. Data are from n = 3 biologically independent experiments. Data in b,e are presented as mean ± s.d. Source data are provided as a Source Data file.

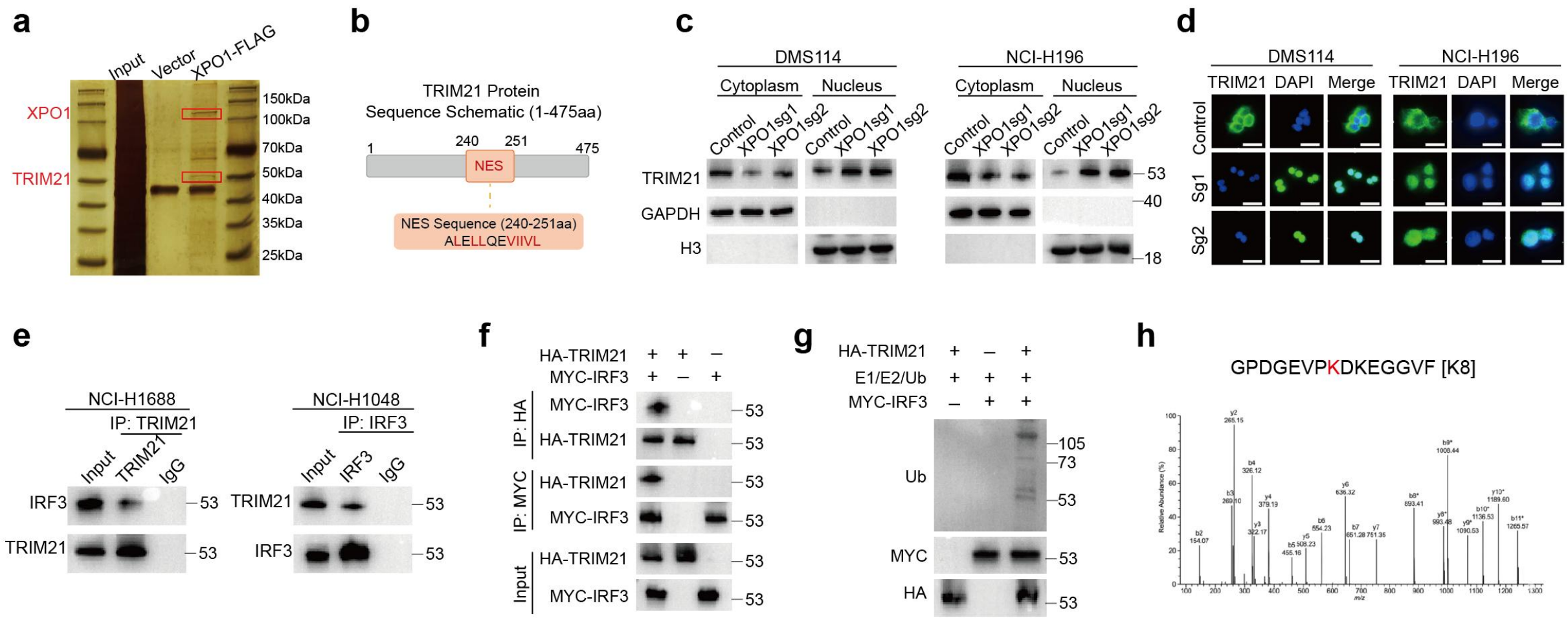

Supplementary Fig. 10 XPO1 interacts with TRIM21 and promotes TRIM21-mediated ubiquitylation of IRF3. **a** Affinity purification and mass spectrometry analysis of FLAG-tagged XPO1-associated protein complexes. Whole-cell lysates from NCI-H1048 cells transfected with empty vector or XPO1-FLAG were subjected to anti-FLAG affinity purification. Bound proteins were resolved by SDS-PAGE and visualized by silver staining, and the remaining eluates were analysed by mass spectrometry. Black arrows indicate XPO1 and TRIM21. **b** Schematic representation of a predicted leucine-rich nuclear export signal (NES) in TRIM21 at amino acids 240-251. The putative NES sequence, ALELLQEVIIVL, is shown, with hydrophobic residues highlighted in red. This schematic was created by the authors using Adobe Illustrator and contains no third-party material. **c** Immunoblot analysis of TRIM21 distribution in the cytoplasmic and nuclear fractions of the indicated SCLC cell lines. GAPDH and histone H3 were used as markers for the cytoplasmic and nuclear fractions, respectively. **d** Immunofluorescence analysis of TRIM21 subcellular localization in the indicated SCLC cell lines following *XPO1* knockdown. Scale bar, 20 μm. **e** Co-immunoprecipitation analysis showing the interaction between endogenous IRF3 and endogenous TRIM21 in NCI-H1688 and NCI-H1048 cells. **f** Co-immunoprecipitation analysis showing the interaction between exogenous MYC-IRF3 and HA-TRIM21 in HEK293T cells. **g** In vitro ubiquitylation assay performed by incubating HA-TRIM21 and MYC-IRF3 in the presence of E1 (UBE1), E2 (UbcH5c), and ubiquitin (FLAG-Ub). **h** Representative tandem mass spectrum of the IRF3 peptide GPDGEVPKDKKEGGVF containing a diglycine-modified lysine, indicating ubiquitination at IRF3 K313 (the eighth residue lysine within the identified peptide). The assigned b and y fragment ions support confident localization of the modified site. Representative images are shown. Data in **a,c-g** are from n = 3 biologically independent experiments. Source data are provided as a Source Data file.

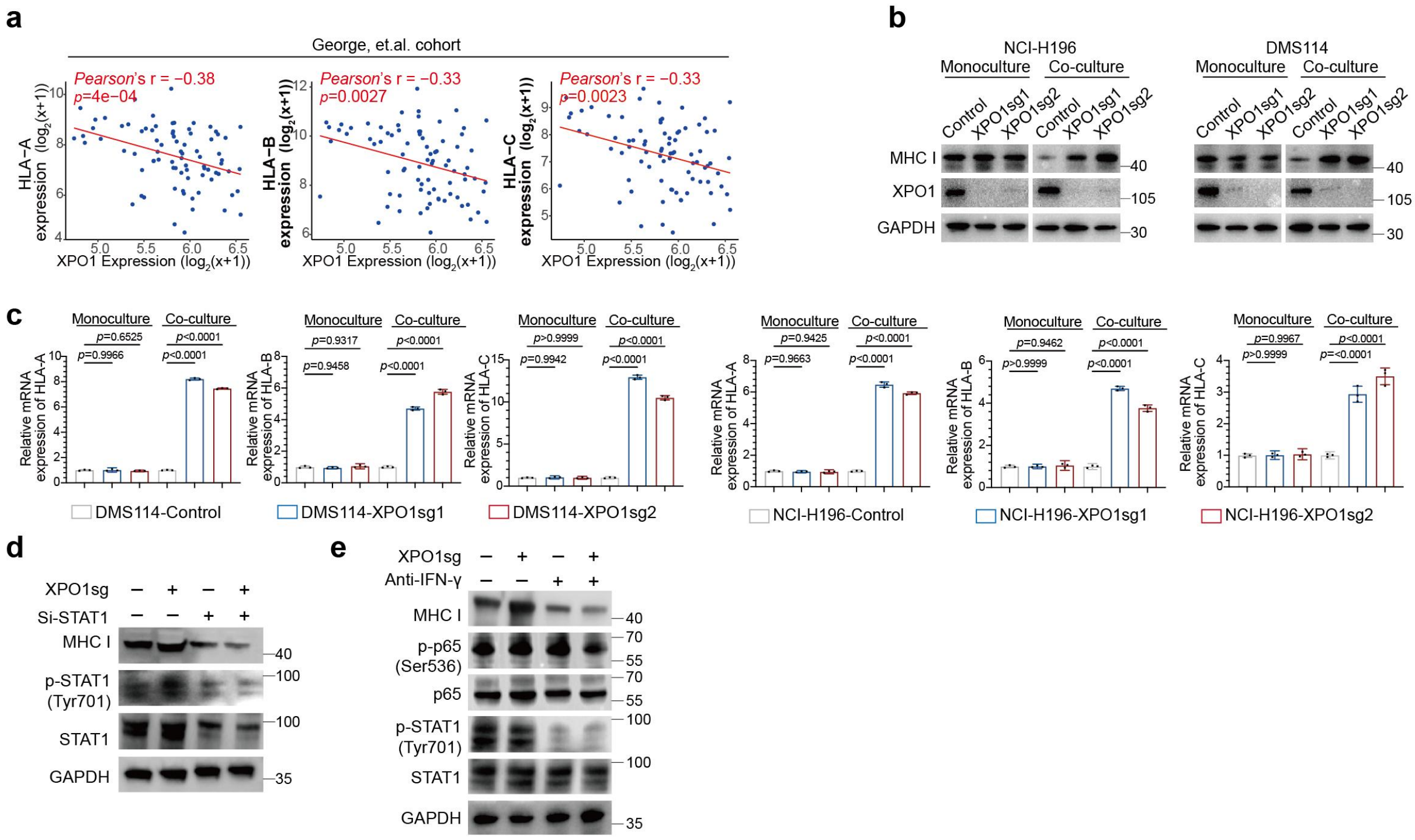

Supplementary Fig. 11 *XPO1* deficiency induces upregulation of MHC I expression in SCLC cells upon co-culture with macrophages. a Pearson correlation analysis between *XPO1* and *HLA-A*, *HLA-B*, and *HLA-C* mRNA expression in the George's et al. dataset (log2(x + 1), n = 81 samples). The correlation coefficient is displayed for each gene pair. **b** Immunoblot analysis of MHC I protein levels in the indicated SCLC cell lines cultured alone or co-cultured with macrophages. **c** RT-qPCR analysis of *MHC I* mRNA levels in the indicated SCLC cells cultured alone or co-cultured with macrophages. **d** Immunoblot analysis of MHC I levels in the indicated SCLC cell lines co-cultured with THP-1-derived macrophages. **e** Immunoblot analysis of MHC I levels in the indicated SCLC cell lines co-cultured with THP-1-derived macrophages and treated with an IFN-γ-neutralizing antibody. Representative images are shown. Data in **b-e** are from n = 3 biologically independent experiments. Data in **a** were analysed using Pearson correlation analysis. Data in **c** were analysed by one-way ANOVA. Data are presented as mean ± s.d. Source data are provided as a Source Data file.

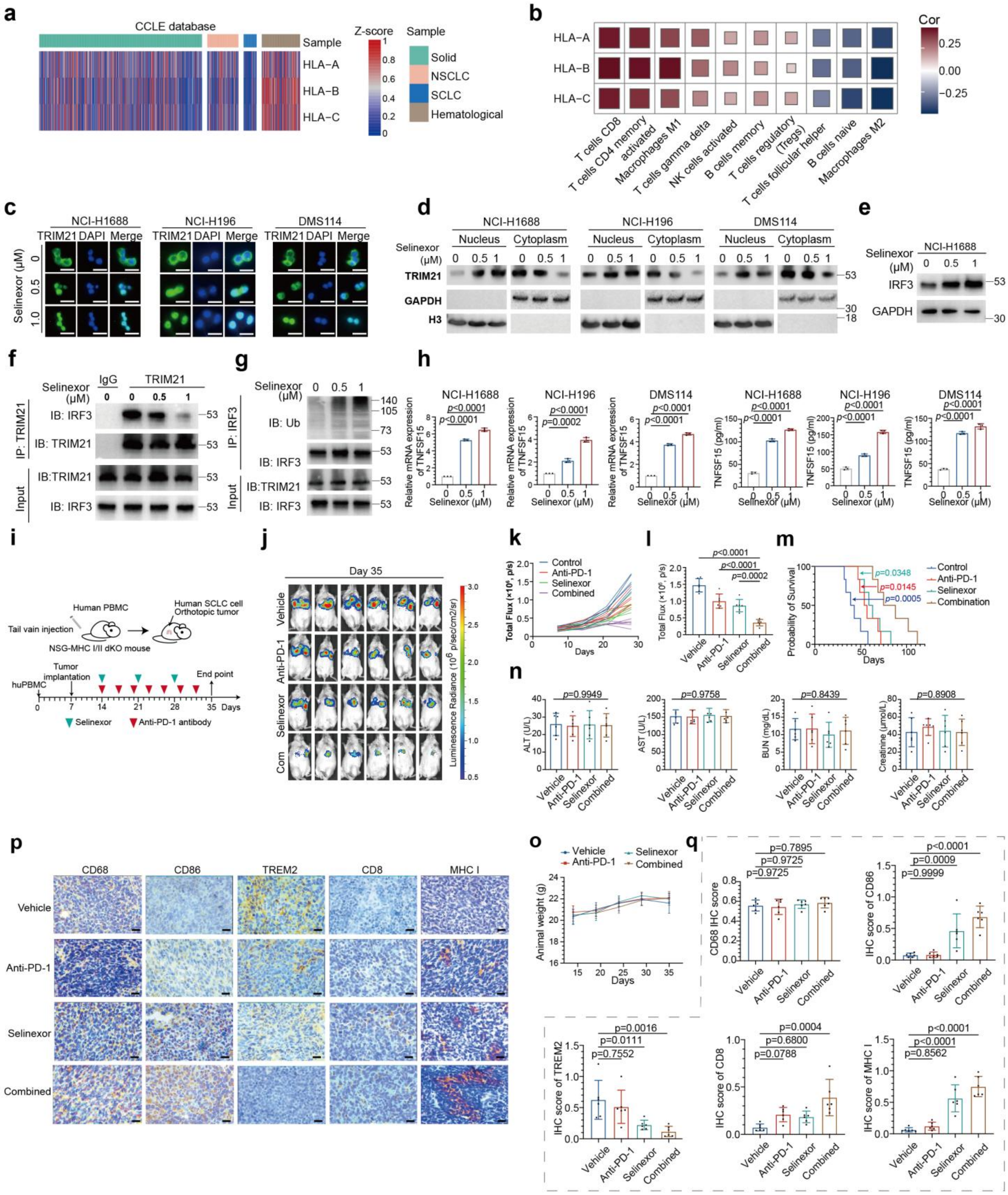

Supplementary Fig. 12 Effects of selinexor treatment in SCLC xenograft models and the impact on immune modulation. **a** Heatmap showing the expression levels of *HLA-A*, *HLA-B*, and *HLA-C* across individual cells, categorized by tumour type. Gene expression data were obtained from the CCLE database and normalized using z-scores. **b** Correlation analysis between *MHC I* expression (*HLA-A*, *HLA-B*, and *HLA-C*) and immune cell infiltration levels in the George et al. cohort, as estimated by the CIBERSORT algorithm. The size of each square corresponds to the absolute value of the correlation coefficient. This schematic was created by the authors using R

programming. **c** Immunofluorescence images showing the relocation of TRIM21 from the cytoplasm to the nucleus in SCLC cell lines following selinexor treatment. Scale bar, 20 µm. **d** Immunoblot analysis of TRIM21 distribution in the cytoplasm and nucleus following selinexor treatment in SCLC cell lines. **e** Immunoblot analysis of IRF3 protein levels in NCI-H1688 cells treated with selinexor. **f** Immunoprecipitation assay showing the interaction between TRIM21 and IRF3 in NCI-H1688 cells treated with selinexor. **g** Ubiquitylation assay showing endogenous IRF3 ubiquitylation in NCI-H1688 cells after treatment with selinexor. **h** RT-qPCR and ELISA analyses of *TNFSF15* transcript levels and secreted protein levels, respectively, in SCLC cells following selinexor treatment. **i** Schematic representation of the construction of a humanized orthotopic SCLC mouse model. 1×10^7 human peripheral blood mononuclear cells (PBMCs) were injected into NCG-MHC I/II-2 dKO mice. **j** Representative bioluminescence imaging (BLI) of mice from the indicated treatment groups at the experimental endpoint. **k** Longitudinal quantification of BLI signal intensity in mice over time. **l** Quantification of tumour burden based on BLI signal intensity at day 28. **m** Kaplan-Meier analysis of survival in mice from the indicated groups. **n** Assessment of liver and kidney function in humanized mice after treatment with anti-PD-1 and selinexor. **o** Mouse body weight across treatment groups at the experimental endpoint. **p** Representative immunohistochemistry images of CD68, TREM2, CD86, CD8, and MHC I staining in xenograft SCLC tissues from the indicated treatment groups. Scale bar, 20 µm. **q** Quantification of the staining shown in **p**. Representative images are shown. Data in **h,l,n,q** were analysed by one-way ANOVA. Data in **m** were compared using the two-sided log-rank test. Data in **c-h** are from n = 3 biologically independent experiments. Data in **j-q** are from n = 6 mice per group. Data are presented as mean ± s.d. Source data are provided as a Source Data file.

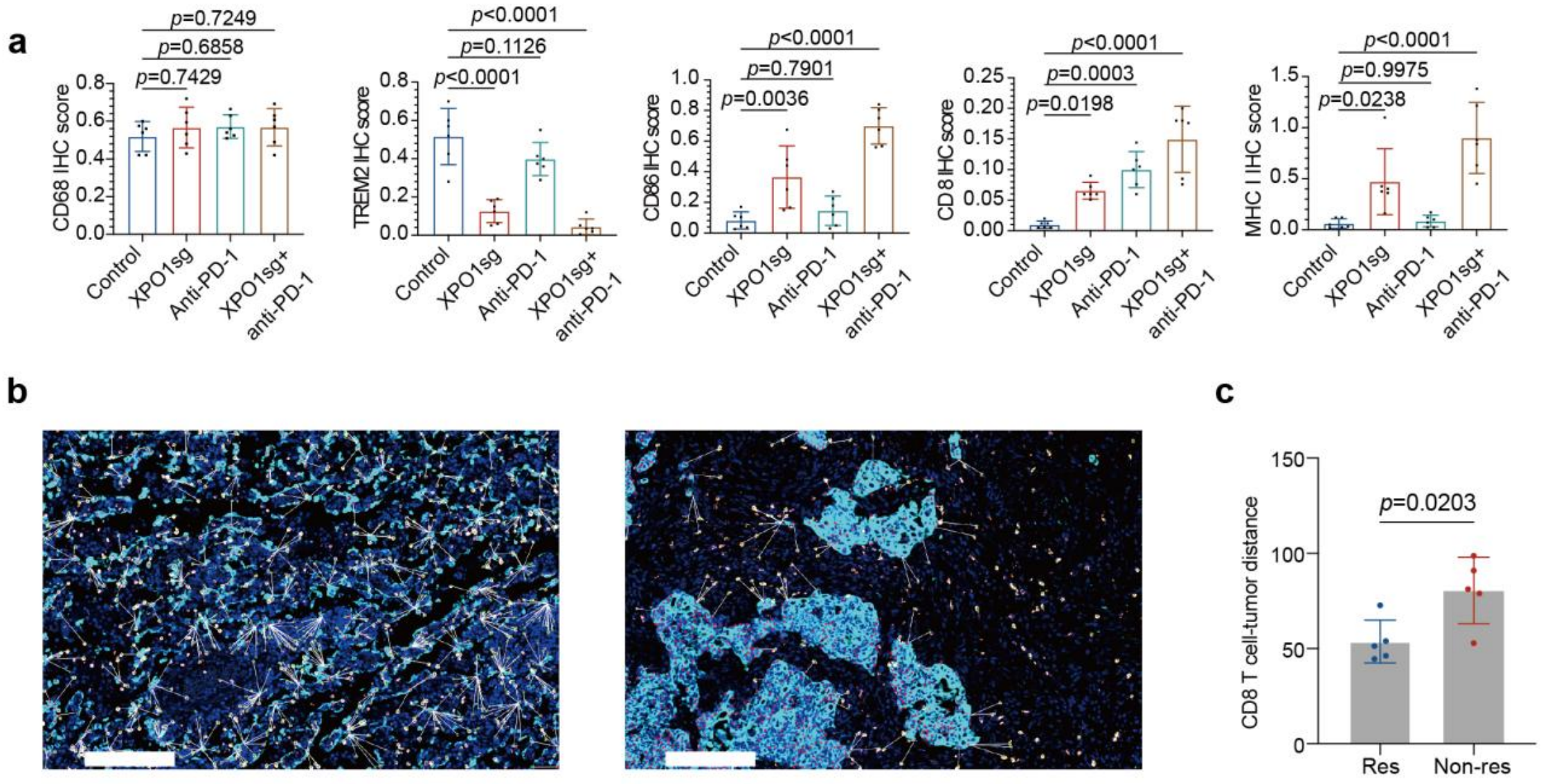

Supplementary Fig. 13. Proximity of CD8⁺ T cells to tumour cells in SCLC. a Quantification CD68, TREM2, CD86, CD8, and MHC I staining in allograft SCLC tissues from the indicated groups. **b** Representative images showing the distance between CD8⁺ T cells (yellow) and tumour cells (ice blue) in tumour samples from immune checkpoint inhibitor (ICI) responders (n = 5) and non-responders (n = 5). Arrows indicate the closest proximity between CD8⁺ T cells and tumour cells. Scale bar, 200 µm. **c** Quantification of the mean nearest distance from CD8⁺ T cells to tumour cells, as shown in **b**. Data in **a** were assessed by one-way ANOVA followed by Sidak's multiple-comparisons test. Data in **c** were analysed using two-sided unpaired t-tests. Data are presented as mean ± s.d. Source data are provided as a Source Data file.

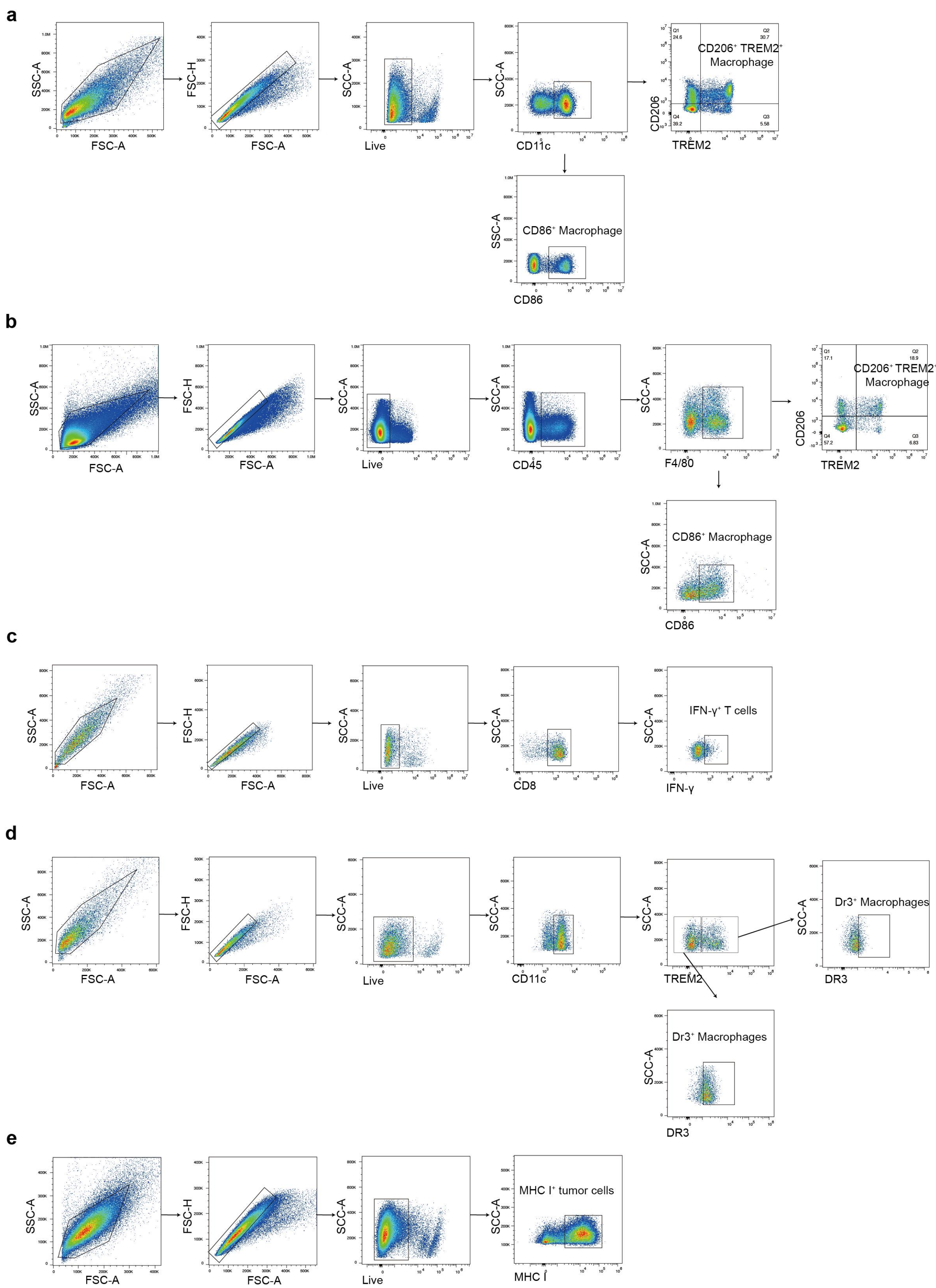

Supplementary Fig. 14. Flow-cytometry gating strategies used for cell analysis and sorting. a Gating strategy used to identify or sort CD11c^{+}CD206^{+}TREM2^{+} macrophages and CD11c^{+}CD86^{+} macrophages in in vitro

co-culture assays, corresponding to data shown in Fig. 2d, Supplementary Fig. 4c-f, Supplementary Fig. 5d, e and Supplementary Fig. 6b, c. **b** Gating strategy used to identify or sort $CD45^+F4/80^+CD206^+TREM2^+$ macrophages and $CD45^+F4/80^+CD86^+$ macrophages from in vivo BALB/c nude mouse tumors, corresponding to data shown in Fig. 2g and Supplementary Fig. 4k. **c** Gating strategy used to identify IFN- γ^+CD8^+ T cells in the in vitro T-cell co-culture assay, corresponding to data shown in Supplementary Fig. 4i. **d** Gating strategy used to identify $CD11c^+TREM2^+DR3^+$ and $CD11c^+TREM2^-DR3^+$ macrophages in the in vitro co-culture assay, corresponding to data shown in Supplementary Fig. 7f, g. **e** Gating strategy used to identify MHC class I^+ SCLC cells in the in vitro macrophage-tumor cell co-culture assay, corresponding to data shown in Fig. 6b, f.

Supplementary Table 1. The association between XPO1 expression and clinicopathological characteristics of small cell lung cancer patients.

Characteristics	Overall<br>N = 98	XPO1 high<br>N = 58	XPO1 low<br>N = 40	P value
Age				0.624
<=60	40 (40.8%)	22 (37.9%)	18 (45.0%)	
>60	58 (59.2%)	36 (62.1%)	22 (55.0%)	
Gender				0.59
Male	82 (83.7%)	50 (86.2%)	32 (80.0%)	
Female	16 (16.3%)	8 (13.8%)	8 (20.0%)	
Smoke				0.906
No	13 (13.3%)	7 (12.1%)	6 (15.0%)	
Yes	85 (86.7%)	51 (87.9%)	34 (85.0%)	
ECOG				0.48
0-1	89 (90.8%)	54 (93.1%)	35 (87.5%)	
>=2	9 (9.18%)	4 (6.90%)	5 (12.5%)	
Stage				0.663
I/II	55 (56.1%)	31 (53.4%)	24 (60.0%)	
III/IV	43 (43.9%)	27 (46.6%)	16 (40.0%)	
Compound				1
Pure	6 (6.12%)	4 (6.90%)	2 (5.00%)	
Combined	92 (93.9%)	54 (93.1%)	38 (95.0%)	
CD86:				**<0.001**
High	49 (50.0%)	20 (34.5%)	29 (72.5%)	
Low	49 (50.0%)	38 (65.5%)	11 (27.5%)	
TREM2:				**0.018**
High	52 (53.1%)	37 (63.8%)	15 (37.5%)	
Low	46 (46.9%)	21 (36.2%)	25 (62.5%)	
MHC I:				**0.01**
High	52 (53.1%)	24 (41.4%)	28 (70.0%)	
Low	46 (46.9%)	34 (58.6%)	12 (30.0%)	
CD8:				**0.014**
High	43 (43.9%)	19 (32.8%)	24 (60.0%)	
Low	55 (56.1%)	39 (67.2%)	16 (40.0%)	

Data were based on tissue IHC assay. Statistical data on XPO1 expression in relation to clinic-histopathologic features for surgical small cell lung cancer specimens. P values were calculated using the $\chi 2$ or fisher exact test. Statistically significant ($p<0.05$).

Supplementary Table 2. Univariate and multivariate Cox regression analyses of overall survival in the small cell lung cancer cohort.

Characteristics	Univariate	CD86			TREM2		
		Multivariate			Multivariate		
	P	HR	95% HR	P	HR	95% HR	P
**Age**							
<=60	Reference						
>60	0.17	1.57	0.85-2.89	0.15	1.72	0.90-3.27	0.10
**Gender**							
Female	Reference						
Male	0.92	0.86	0.34-2.15	0.74	1.11	0.44-2.78	0.83
**Smoke**							
NO	Reference						
Yes	0.03	2.80	0.93-8.44	0.07	**3.89**	**1.21-12.50**	**0.02**
**ECOG**							
0-1	Reference						
>=2	0.18	1.55	0.63-3.82	0.34	2.13	0.82-5.54	0.12
**AJCC 7th Stage**							
I/II	Reference						
III/IV	**0.01**	**2.09**	**1.16-3.78**	**0.01**	**2.49**	**1.37-4.53**	**<0.01**
**Type**							
Pure	Reference						
Combined	0.18	1.69	0.56-5.09	0.35	1.99	0.66-6.03	0.22
**XPO1&CD86**							
$XPO1^{Low}$ & $CD86^{High}$	Reference						
Others	0.12	3.82	1.36-10.71	**0.01**	NA	NA	NA
$XPO1^{High}$ & $CD86^{Low}$	**0.03**	7.96	3.03-20.92	**<0.01**	NA	NA	NA
**XPO1&TREM2**							
$XPO1^{Low}$ & $TREM2^{Low}$	Reference						
Others	0.21	NA	NA	NA	**3.90**	**1.40-10.85**	**0.01**
$XPO1^{High}$ & $TREM2^{High}$	**0.03**	NA	NA	NA	**9.47**	**3.48-25.71**	**<0.01**

Data were based on tissue immunohistochemistry (IHC) assays. Univariate and multivariate Cox proportional hazards regression analyses were performed to estimate hazard ratios (HRs) and 95% confidence intervals (CIs). P values < 0.05 were considered statistically significant.

Supplementary Table 3. Clinicopathological characteristics of small cell lung cancer patients treated with first-line immune checkpoint blockade.

Characteristics	Responder N = 5	Non-responder N = 5
Age, years	61.4 ± 8.21	63.6 ± 7.72
Gender		
Male	3 (60%)	4 (80%)
Female	2 (40%)	1 (20%)
Smoke		
No	2 (40%)	1 (20%)
Yes	3 (60%)	4 (80%)
ECOG		
0-1	5 (100%)	5 (100%)
>=2	0 (0%)	0 (0%)
Treatment response		
Complete response	1 (20%)	NA
Partial response	4 (80%)	NA
Stable disease	NA	3 (60%)
Progressive disease	NA	2 (40%)

Supplementary Table 4. The primers and RNA sequence used in this study.

qPCR		
ID	Primer	5' to 3'
Human *GAPDH*	Forward	TCGGAGTCAACGGATTTGGT
	Reverse	TTCCCGTTCTCAGCCTTGAC
Human *CD86*	Forward	TAGGTCACAGCAGAAGCAGC
	Reverse	AATCAAAACTTGTGCGGCCC
Human *CXCL10*	Forward	AGCTCTACTGAGGTGCTATGT
	Reverse	GTACCCTTGGAAGATGGGAAAG
Human *TREM2*	Forward	CCAGCCTGCATACTTGCCA
	Reverse	GGCAGAGTAGTCTCTTGCCAG
Human *CD206*	Forward	GGACGTGGCTGTGGATAAAT
	Reverse	ACCCAGAAGACGCATGTAAAG
Human *TNFSF15*	Forward	CACCACATACCTGCTTGTCAGC
	Reverse	TCTCCGTCTGCTCTAAGAGGTG
Human *IRF3*	Forward	AGAGGCTCGTGATGGTCAAG
	Reverse	AGGTCCACAGTATTCTCCAGG
Human *HLA-A*	Forward	ACCCTCGTCCTGCTACTCTC
	Reverse	CTGTCTCCTCGTCCCAATACT
Human *HLA-B*	Forward	CAGTTCGTGAGGTTCGACAG
	Reverse	CAGCCGTACATGCTCTGGA
Human *HLA-C*	Forward	CAAGGACAGCTAGGACAACC
	Reverse	CTGGGGAAGTCACCCTACC
Ch-IP		
Primer NC	Forward	TCGGAGTCAACGGATTTGGT
	Reverse	TTCCCGTTCTCAGCCTTGAC
Primer Site 1	Forward	AGGTTCAAAATGACAGAGGGCT
	Reverse	GGAGTGTGCTTCTGGAAGTGA
Primer Site 2	Forward	TCTGTTGTAGGCGGTGCATT
	Reverse	GCTCTCACAGTGGGTGACTT
sgRNA		
ID	Sequence	5' to 3'
XPO1	sgRNA sequence 1	GTGGTGAATTGCTTATACCA
	sgRNA sequence 2	TCGACTCTTGTCCAAGCATC
	sgRNA sequence 3	GCTAACATTGTCATAATTGC
shRNA		
ID	Sequence	5' to 3'
TNFSF15	shRNA target sequence 1	CAGCAAGTTTATGCACCTCTT
	shRNA target sequence 2	GCCATGTTCTCCTTGCAAGAA
TRIM21	shRNA target sequence 1	AGTATCAGCCACGGATTGG
	shRNA target sequence 2	TCCAGAGTGAAAGTGCTGG
IRF3	shRNA target sequence 1	GATCTGATTACCTTCACGGAA

	shRNA target sequence 2	GCAGGAGGATTTCGGAATCTT
siRNA		
STAT1	siRNA sense sequence 1	GCACCUGCAAUUGAAAGAATT
	siRNA antisense sequence 1	UUCUUUCAAUUGCAGGUGCTT
	siRNA sense sequence 2	GCUGGAUGAUCAAUAUAGUTT
	siRNA antisense sequence 2	ACUAUAUUGAUCAUCCAGCTT

Supplementary Table 5. Main antibodies and reagents used in this study.

Antibody	Cat.No.	Company	Dilution
WB			
Anti-human-GAPDH	2118	CST	1/1000
anti-human-Histone H3	4499	CST	1/2000
Anti-human-XPO1	46249	CST	1/1000
Anti-human-IRF3	4302	CST	1/1000
Anti-human-TRIM21	12108-1-AP	Proteintech	1/5000
anti-human-Ubiquitin	58395	CST	1/1000
Anti-human-FLAG tag	F1804	sigma	1/1000
Anti-human-GST tag	ab184804	abcam	1/1000
Anti-human-MYC tag	ab32	abcam	1/1000
Anti-human-HA-tag	ab9110	abcam	1/4000
Anti-human-MHC-I	Ab70328	abcam	1/500
Anti-human-NF-κB	8242	CST	1/1000
Anti-human-Phospho-NF-κB	3033	CST	1/1000
Anti-human-STAT1	14994	CST	1/1000
Anti-human-Phospho-STAT1	9167	CST	1/1000
Anti-human-DR3	4591	CST	1/1000
IHC			
Anti-human-XPO1	46249	CST	1/300
Anti-human-CD86	ab220188	abcam	1/100
Anti-human-TREM2	ab318262	abcam	1/100
Anti-human-MHC I	Ab70328	abcam	1/20000
Anti-human-CD8	ab237709	abcam	0.25μg/ml
Anti-human-CD68	ab955	abcam	1/3000
Anti-mouse-CD86	19589	CST	1/1000
Anti-mouse-TREM2	ab305103	abcam	1/500
Anti-mouse-MHC-I	76828	CST	1/250
Anti-mouse-CD8	ab217344	abcam	1/2000
Anti-mouse-CD68	ab283654	abcam	1/100
IF			
Anti-human-TRIM21	67136-1-Ig	Proteintech	1/400
FC			
PE/Cyanine7 anti-mouse CD45 Antibody	147703	BioLegend	1.25μl/million
PerCP/Cyanine5.5 anti-mouse-F4/80 antibody	123117	BioLegend	5μl/million
FITC anti-mouse-CD86 antibody	105005	Biolegend	2μl/million
APC anti-mouse CD206 (MMR) Antibody	141707	Biolegend	2.5μl/million
PE anti-mouse-TREM2 antibody	824805	Biolegend	1.25μl/million
PerCP/Cyanine5.5 anti-human CD86 antibody	374215	Biolegend	5μl/million
FITC anti-human CD206 (MMR) Antibody	321103	Biolegend	5μl/million
APC anti-human-TREM2 antibody	FAB17291A	R&D	1/100
PE/Cyanine7 anti-human-HLA-A,B,C antibody	311429	BioLegend	5μl/million

FITC anti-human CD11c Antibody	337213	BioLegend	5μl/million
PE anti-human DR3 (TRAMP) Antibody	307105	BioLegend	5μl/million
PE anti-human IFN- γ Antibody	502508	BioLegend	5μl/million
FITC anti-human CD8 Antibody	344703	BioLegend	5μl/million

Supplementary Table 6. The promoter of *TNFSF15*.

**Promoter/Upstream by 2000 bases**
***TNFSF15* promoter-binding site WT**
TGAGTAACCATGGGACATCAAAATCATCCTTTGCTAGAATTCTTTGAAAATACGTTAGA<br>TGCTTATTTTAGTAGATGGTAATGAGTCTATTGAATAACAGTTAGGAGCAATAGTCATG<br>GGGAAGGGTTATGACTTAGTACCTGCTCTCCCAGACCTGATGGTCTAATTGGGGAGAC<br>AGAAGGAATGGTCAAACATAGTCTAGTAGGAGGTATTCAATGATAGGGAAGAAAGGG<br>AAGCTATAGGAACATAGAATATGATCATCTTACCCAATCTTGGAGGCAGGCGGATACA<br>AAAATGGTTTCTACACACAATTAAGGAACAGGAGCATAGAAAAGAAAGGTTAAGTTA<br>CAATAGCAGAGATTTAGGTTAGTCTCAAAGACATTTTTTAATAGATTTGTTAAACCAAG<br>AATGAGTAGCCATGGGACATGAAAATCATCCTTTGCTAGAATGAGCATAGGAGCATAG<br>TGATATGAAGGAAGAGGACTGGACACTTCCATTTTGCAACAGTACAGCTAGTCAATAG<br>TGTATCCAGGGAACTGCAGCTTGGGAAAGTAGTAAAAGTTGAGGCTGGACACTGGGGA<br>CGGGTGACAGTGAGCAGGGACCAAGCCATGAAAGGCCTTTCCAAGTAGCCTGAAGAG<br>TCCAGGCTGTGACATAACCAGAAGTAGCAACTGGATGGAGGCGCTAGTAAAACTGGTT<br>TCTGCCGTACATCACAGGCCATTCCTTAAACTTTCCTATTATTTCTAGAACCCATCTTTT<br>CCCAGGGTCCTTTACTTTCCTCTGAGATCAACCCTCCCTGAGCTCTAAGGCCGAGTTCC<br>AACACCACCTCTTTCTCCAGGAAGCCCCCTCAACCCCCTCACTGCAGGGTCTTACCCAC<br>TCCTGGGTAAAGACAGTGCAGGTATTTATGAACATGCATGATCCAGCAGTACCTATGG<br>TGAAGTGTGATGGTGAAGAAGCCAGCAGCCAGCCTGCCTGAGTCCCAATCCTGGCTCT<br>ACCACTTGCCGTCTGTGCTTTATTTTCTTATGTGTAAAATGAGAATGATAGCTTTATTGT<br>CACAAAATTTATCTCAGGATTGAATAATTTTATTGATGTATTTAACTTAGAGCAGTGAT<br>TGGCACAAAGTAAGCACTTAATAAATATAATCTTTTATTACATGTTGTGGCCCCCACAC<br>CCAACTGCAGGCAAAGCACATCACATGATCAATTAACTTTAGACTAATCAGGGAGTAG<br>TGGTTAATGTTCTACCCTCACAGACAGTCACCTCGATCTGTGGCCTCATACTCTGATTA<br>AACAATGGTCAATGCAGACTGGAAATACTTTCCTTTCATGGGCAGTCATGTGCTAGAG<br>GTTACAACTCCAAGCCGTGATTTAGAACTAGGTCATCTTCCTAGCTCCTCTGTCTTCATC<br>CTTGGATGACCTAGGACAAGTGCCTTCACCTTTTAGAACTTTGCTATTCCCATCTGTAC<br>ACTGAGAATGAATTTCTATGAGGTTCAAAATGACAGAGGGCTAGGCAGCACTTTGCAA<br>AGAGCTGATGCAAATG**TGATTTCCGTTTCCCAATCTG**CAAACCACACACCTCTCATC<br>CCTCACTTTCACTTCCAGAAGCACACTCCCCAGACTGGCACAGCTGGTAAGCCTCCAGT<br>GCAGGGAACAGGTATATTTTAATTTGCCCCACCCACTGACTTGATGAATTCTTTCTCTC<br>CTCCTTCAGGGACTTTCCTAACTTCCTTCTGTAAACGAAGGAAGGAGCACATGAGCATA<br>CTGTATATAAATATGCTTGGGAAAGCTTTCTTCTTCTCCTTCTTCTTCTTTTTTTTTTTTT<br>TTTTTTTTTCCTCAAACCAAACTTGGTTTCTGTTGTAGGCGGTGCATTCTCTAGCAGGGT<br>AGGGCCACAGTGTGCTCTTGAAGAGGGGA**GGGGAGAGGAAAAGGGAAGGA**GGAGA<br>CTGAGTGATTAAGTCACCCACTGTGAGAGCTGGTCTTCTATTTAATGGGGGCTCTCTCT<br>GCCCAGGAGTC
***TNFSF15* promoter-binding site MUT 1**
TGAGTAACCATGGGACATCAAAATCATCCTTTGCTAGAATTCTTTGAAAATACGTTAGA<br>TGCTTATTTTAGTAGATGGTAATGAGTCTATTGAATAACAGTTAGGAGCAATAGTCATG<br>GGGAAGGGTTATGACTTAGTACCTGCTCTCCCAGACCTGATGGTCTAATTGGGGAGAC<br>AGAAGGAATGGTCAAACATAGTCTAGTAGGAGGTATTCAATGATAGGGAAGAAAGGG<br>AAGCTATAGGAACATAGAATATGATCATCTTACCCAATCTTGGAGGCAGGCGGATACA<br>AAAATGGTTTCTACACACAATTAAGGAACAGGAGCATAGAAAAGAAAGGTTAAGTTA

CAATAGCAGAGATTTAGGTTAGTCTCAAAGACATTTTTTAATAGATTTGTTAAACCAAG<br>AATGAGTAGCCATGGGACATGAAAATCATCCTTTGCTAGAATGAGCATAGGAGCATAG<br>TGATATGAAGGAAGAGGACTGGACACTTCCATTTTGCAACAGTACAGCTAGTCAATAG<br>TGTATCCAGGGAACTGCAGCTTGGGAAAGTAGTAAAAGTTGAGGCTGGACACTGGGGA<br>CGGGTGACAGTGAGCAGGGACCAAGCCATGAAAGGCCTTTCCAAGTAGCCTGAAGAG<br>TCCAGGCTGTGACATAACCAGAAGTAGCAACTGGATGGAGGCGCTAGTAAAACTGGTT<br>TCTGCCGTACATCACAGGCCATTCCTTAAACTTTCCTATTATTTCTAGAACCCATCTTTT<br>CCCAGGGTCCTTTACTTTCCTCTGAGATCAACCCTCCCTGAGCTCTAAGGCCGAGTTCC<br>AACACCACCTCTTTCTCCAGGAAGCCCCCTCAACCCCCTCACTGCAGGGTCTTACCCAC<br>TCCTGGGTAAAGACAGTGCAGGTATTTATGAACATGCATGATCCAGCAGTACCTATGG<br>TGAAGTGTGATGGTGAAGAAGCCAGCAGCCAGCCTGCCTGAGTCCCAATCCTGGCTCT<br>ACCACTTGCCGTCTGTGCTTTATTTTCTTATGTGTAAAATGAGAATGATAGCTTTATTGT<br>CACAAAATTTATCTCAGGATTGAATAATTTTATTGATGTATTTAACTTAGAGCAGTGAT<br>TGGCACAAAGTAAGCACTTAATAAATATAATCTTTTATTACATGTTGTGGCCCCCACAC<br>CCAACTGCAGGCAAAGCACATCACATGATCAATTAACTTTAGACTAATCAGGGAGTAG<br>TGGTTAATGTTCTACCCTCACAGACAGTCACCTCGATCTGTGGCCTCATACTCTGATTA<br>AACAATGGTCAATGCAGACTGGAAATACTTTCCTTTCATGGGCAGTCATGTGCTAGAG<br>GTTACAACTCCAAGCCGTGATTTAGAACTAGGTCATCTTCCTAGCTCCTCTGTCTTCATC<br>CTTGGATGACCTAGGACAAGTGCCTTCACCTTTTAGAACTTTGCTATTCCCATCTGTAC<br>ACTGAGAATGAATTTCTATGAGGTTCAAAATGACAGAGGGCTAGGCAGCACTTTGCAA<br>AGAGCTGATGCAAATG**AAAAAAAAAAAAAAAAAAAA**CAAACCACACACCTCTCATCCC<br>TCACTTTCACTTCCAGAAGCACACTCCCCAGACTGGCACAGCTGGTAAGCCTCCAGTGC<br>AGGGAACAGGTATATTTTAATTTGCCCCACCCACTGACTTGATGAATTCTTTCTCTCCTC<br>CTTCAGGGACTTTCCTAACTTCCTTCTGTAAACGAAGGAAGGAGCACATGAGCATACT<br>GTATATAAATATGCTTGGGAAAGCTTTCTTCTTCTCCTTCTTCTTCTTTTTTTTTTTTTTT<br>TTTTTTCCTCAAACCAAACTTGGTTTCTGTTGTAGGCGGTGCATTCTCTAGCAGGGTAG<br>GGCCACAGTGTGCTCTTGAAGAGGGGAGGGGAGAGGAAAAGGGAAGGAGGAGACTG<br>AGTGATTAAGTCACCCACTGTGAGAGCTGGTCTTCTATTTAATGGGGGCTCTCTCTGCC<br>CAGGAGTC
***TNFSF15* promoter-binding site MUT 2**
TGAGTAACCATGGGACATCAAAATCATCCTTTGCTAGAATTCTTTGAAAATACGTTAGA<br>TGCTTATTTTAGTAGATGGTAATGAGTCTATTGAATAACAGTTAGGAGCAATAGTCATG<br>GGGAAGGGTTATGACTTAGTACCTGCTCTCCCAGACCTGATGGTCTAATTGGGGAGAC<br>AGAAGGAATGGTCAAACATAGTCTAGTAGGAGGTATTCAATGATAGGGAAGAAAGGG<br>AAGCTATAGGAACATAGAATATGATCATCTTACCCAATCTTGGAGGCAGGCGGATACA<br>AAAATGGTTTCTACACACAATTAAGGAACAGGAGCATAGAAAAGAAAGGTTAAGTTA<br>CAATAGCAGAGATTTAGGTTAGTCTCAAAGACATTTTTTAATAGATTTGTTAAACCAAG<br>AATGAGTAGCCATGGGACATGAAAATCATCCTTTGCTAGAATGAGCATAGGAGCATAG<br>TGATATGAAGGAAGAGGACTGGACACTTCCATTTTGCAACAGTACAGCTAGTCAATAG<br>TGTATCCAGGGAACTGCAGCTTGGGAAAGTAGTAAAAGTTGAGGCTGGACACTGGGGA<br>CGGGTGACAGTGAGCAGGGACCAAGCCATGAAAGGCCTTTCCAAGTAGCCTGAAGAG<br>TCCAGGCTGTGACATAACCAGAAGTAGCAACTGGATGGAGGCGCTAGTAAAACTGGTT<br>TCTGCCGTACATCACAGGCCATTCCTTAAACTTTCCTATTATTTCTAGAACCCATCTTTT<br>CCCAGGGTCCTTTACTTTCCTCTGAGATCAACCCTCCCTGAGCTCTAAGGCCGAGTTCC<br>AACACCACCTCTTTCTCCAGGAAGCCCCCTCAACCCCCTCACTGCAGGGTCTTACCCAC<br>TCCTGGGTAAAGACAGTGCAGGTATTTATGAACATGCATGATCCAGCAGTACCTATGG

TGAAGTGTGATGGTGAAGAAGCCAGCAGCCAGCCTGCCTGAGTCCCAATCCTGGCTCT
ACCACTTGCCGTCTGTGCTTTATTTTCTTATGTGTAAAATGAGAATGATAGCTTTATTGT
CACAAAATTTATCTCAGGATTGAATAATTTTATTGATGTATTTAACTTAGAGCAGTGAT
TGGCACAAAGTAAGCACTTAATAAATATAATCTTTTATTACATGTTGTGGCCCCCACAC
CCAACTGCAGGCAAAGCACATCACATGATCAATTAACTTTAGACTAATCAGGGAGTAG
TGGTTAATGTTCTACCCTCACAGACAGTCACCTCGATCTGTGGCCTCATACTCTGATTA
AACAATGGTCAATGCAGACTGGAAATACTTTCCTTTCATGGGCAGTCATGTGCTAGAG
GTTACAACTCCAAGCCGTGATTTAGAACTAGGTCATCTTCCTAGCTCCTCTGTCTTCATC
CTTGGATGACCTAGGACAAGTGCCTTCACCTTTTAGAACTTTGCTATTCCCATCTGTAC
ACTGAGAATGAATTTCTATGAGGTTCAAAATGACAGAGGGCTAGGCAGCACTTTGCAA
AGAGCTGATGCAAATGTGATTTCCGTTTCCCAATCTGCAAACCACACACCTCTCATCCC
TCACTTTCACTTCCAGAAGCACACTCCCCAGACTGGCACAGCTGGTAAGCCTCCAGTGC
AGGGAACAGGTATATTTTAATTTGCCCCACCCACTGACTTGATGAATTCTTTCTCTCCTC
CTTCAGGGACTTTCCTAACTTCCTTCTGTAAACGAAGGAAGGAGCACATGAGCATACT
GTATATAAATATGCTTGGGAAAGCTTTCTTCTTCTCCTTCTTCTTCTTTTTTTTTTTTTTTT
TTTTTTCCTCAAACCAAACTTGGTTTCTGTTGTAGGCGGTGCATTCTCTAGCAGGGTAG
GGCCACAGTGTGCTCTTGAAGAGGGGAAAAAAAAAAAAAAAAAAAAAAGGAGACTG
AGTGATTAAGTCACCCACTGTGAGAGCTGGTCTTCTATTTAATGGGGGCTCTCTCTGCC
CAGGAGTC

Supplementary references:

1 Ireland, A. S. *et al.* MYC Drives Temporal Evolution of Small Cell Lung Cancer Subtypes by Reprogramming Neuroendocrine Fate. *Cancer Cell* **38**, 60-78 e12 (2020). https://doi.org/10.1016/j.ccell.2020.05.001

2 Azizi, E. *et al.* Single-Cell Map of Diverse Immune Phenotypes in the Breast Tumor Microenvironment. *Cell* **174**, 1293-1308 e1236 (2018). https://doi.org/10.1016/j.cell.2018.05.060
