# Supplementary material for: XPO1-mediated TRIM21 nuclear export reprograms TREM2+ macrophage polarization by targeting IRF3 to augment anti-PD-1 efficacy in small cell lung cancer
Source: Nat Commun. 2026 Aug 22;17:10050. doi: 10.1038/s41467-026-76862-0 (PMC13594258; doi:10.1038/s41467-026-76862-0)
Supplement: Supplementary file 2 [file 41467_2026_76862_MOESM2_ESM.pdf]

nature portfolio

Peer Review File

XPO1-mediated TRIM21 nuclear export reprograms TREM2+ macrophage polarization by targeting IRF3 to augment anti-PD-1 efficacy in small cell lung cancer

Corresponding Author: Professor Dingzhi huang

This file contains all reviewer reports in order by version, followed by all author rebuttals in order by version.

Version 0:

Reviewer comments:

Reviewer #1

(Remarks to the Author)
Strengths:
Connects a tumor intrinsic feature to TME alteration
Investigates macrophage role in SCLC, which is underexplored.
The proposed XPO1–TRIM21–IRF3 axis controlling TNFSF15 appears to be novel.
Detailed biochemical analyses.
Weakness:
How does XPO+/- subgroups relate to the known SCLC subtypes?
The models used are P subtype cell line, which is a rare IO unresponsive subtype. Would be important to verify key findings in a more generalizable cell line
Selinexor has many off target effects, so would be important to do the key IO combo experiment with more selective agents or XPO KO.
Specific comments:
1. Fig 1 I. Add canonical nonNE genes – REST, YAP, MYC etc;
Fig. 1k . show absolute number of macrophage subtypes (in addition to proportions)
Fig 1l. Show additional pathways to confirm that XPO blockade is indeed immunosuppressive (beyond APM) . Please provide cluster costimulatory/inhibitory profiling to establish that TREM2+population presents antigen in a tolerogenic manner.
2. Fig2. THP1 is a leukemia cell line which is used in coculture. Consider alternate source of macrophage to verify
H1048 is a Pou2f3 cell line, one of the few ones of this category of rare P subtype, which is inherently IO unresponsive. Consider a different cell line as well.
3. In Extended 4 c . Error M1c instead of repeating M1a
4. Table 8: Please report both # targets (and % of peaks) and effect size (fold-enrichment/odds ratio with CI) against a GC/length-matched background, plus central-enrichment/footprinting.
5. Fig 6 d: fludarabine and BAY 11-7082 have notable off-targets. To confirm STAT1 specificity with please provide one genetic perturbation (STAT1 or JAK1 siRNA), a clean JAK inhibitor control (ruxolitinib), IFN-γ neutralization in co-culture, and matched pathway readouts (pSTAT1↑, stable NF-κB).
9. Fig. 7f shows good images but could reflect a single field. Please provide cohort-level quantification: per-patient XPO1 and MHC-I H-scores, TREM2⁺ and CD86⁺ macrophage densities (and CD86/TREM2 ratio), CD8⁺ density and CD8–tumor distance, with violin/box plots (N, effect size, 95% CI), correlations, and a multivariable model for response. ROIs should be pre-specified, selected blinded to outcome, analyzed by automated segmentation, and stratified/adjusted for biopsy site. Include segmentation masks and raw counts in the supplement.

Reviewer #2

(Remarks to the Author)
This manuscript is an impressive attempt to understand and mechanistically clarify the immunoregulatory circuit of Exportin1

(XPO1). This is also a translationally relevant investigation for the treatment of small cell lung cancer indication especially with the limited success in the treatment of SCLC with anti-PD1 antibodies. The authors propose that XPO1 participates in the transport of TRIM21 from nucleus to cytoplasm. TRIM21 is presumed to be held in the nucleus when XPO1 transport is blocked with Selinexor reversing the ubiquitin ligase mediated degradation of IRF3 in the cytoplasm. The authors also use ATAC-Seq and ChIP assays to demonstrate that IRF3 binds to the promoter region of a distinct cytokine, TNFSF15. The recent increased interest in targeting TNFSF15 (TL1A) for immunotherapy amplifies the importance of this manuscript. Together, the collective data points to the role of XPO1 in transcriptional regulation of TNFSF15 through IRF3-mediated promoter activation and indirectly modulating macrophage functionality in SCLC TME.

Favorable evaluation/commendation:
1)The figures /data are convincingly able to link the proposed mechanism of action including TRIM21 cytoplasmic relocalization upon overexpression of XPO1 (Figures 4G-H), ubiquitination loss when using a point mutant (Figure 5J), and IRF3 ChIP at TNFSF15 promoter (Figure 3J)
a.Adequate data strength in demonstrating XPO1–TRIM21–IRF3 axis: Extended Data Figs. 11–13 show cycloheximide/MG132 chase assays, IP of endogenous TRIM21–IRF3, and ubiquitination blots. These provide stronger evidence that XPO1 promotes IRF3 degradation through TRIM21. Extended Data Fig. 16 shows Selinexor reversing TRIM21 nuclear export and reducing IRF3 ubiquitination, directly supporting the main mechanistic model.
2)The authors are able to bridge the molecular biology aspects with therapeutic relevance. IHC correlations of XPO1, TREM2, CD86, Class I in patient tumors, and functional synergy of XPO1 inhibition (Selinexor) in combination with anti-PD-1 in mouse models.
3)Using multiple orthogonal methods like scRNAseq, mouse in-vivo models, biochemical assays, and extending some observations with clinical samples, the authors add strength to their interpretation of results

Feedback for Manuscript Improvement
1)Previous reports have shown that XPO1 inhibition, specifically using Selinexor, has been shown to skew the ratios of macrophage subsets within the tumor microenvironment (TME). Abundance shifts in TREM2/CD206-positive TAMs is not a direct evidence that these macrophages are suppressive in SCLC. The interpretation that these macrophages drive immune exclusion is based on correlative transcriptomic and IHC data.
2)The claim "XPO1 inhibition suppresses TREM2+ macrophage differentiation in murine [syngeneic] tumors" is not substantiated with data. Specifically, XPO1 inhibition significantly reduced the proportion of "M2c" macrophages that co-express CD206 and TREM2. The pseudotime trajectory analyses (Fig. 1m) is suggestive of this but is not an experimental evidence for suppression of differentiation. PS: The broader consensus in the field is to avoid the use of M1, M2, etc. macrophage nomenclature

a.Causal function needs to be experimentally evaluated or the language appropriately modified to indicate correlation or that the available data is suggestive. Transcriptomic signatures alone do not prove suppressive function. Note that the authors already show no variation in T cell (effector/memory/exhausted) subsets after XPO1 inhibition (Extended Data Fig. 3a-e)
b.The scRNA data show TREM2$^+$ macrophages are transcriptionally distinct and enriched for antigen-presentation genes, yet the manuscript claims they are immunosuppressive and drive immune exclusion. The discrepancy in "antigen presentation" gene set narrative should be clarified.
c.For example, how would the subset of TAMs behave in T-cell suppressive assays / what would be their functional activity (cytokine secretion /protein markers) ex-vivo/in-vitro?
d.Reproducibility - Reviewing Figure 1K or Extended Data Figure 2a, one would assume that it is a sample size of 3 animals per group. The effect size seems to be low to convincingly demonstrate a differential effect on M2c variation (1K) or direct anti-tumor effect (1e, 1f) without having an understanding about the variability and sample size tested. On the other hand, n=6 per group (should be clearly marked in text or figure legends) as shown in Figure 2e and 2f seems convincing. Strongly recommend that the authors explicitly state the number of biological /technical replicates and number of animals used per group in all experiments. If possible and only where feasible, data in Figure 2 could be repeated with additional animals.

3)Tumor TNFSF15 levels alter macrophage state in co-culture (Figure 2). Given the emerging interest in TL1A/DR3 biology, it is critical to understand the expression of the receptors for TNFSF15 (e.g., DR3/TNFRSF25, DcR3) on the different subsets of TAMs vs tumor cells themselves.
a.To confirm the mechanism and rule out any indirect effect of other secreted factors, it would be great to evaluate and present the data on the expression of TNFSF15 receptors on the macrophages used in the assay system (THP-1 derived as well as primary TAMs) by FACS.
b.Does recombinant TNFSF15 activate downstream signaling in macrophages?

4)XPO1 knockdown in SCLC cells results in upregulation of not just TNFSF15 but also CXCL1, CCL22, IL1B, and TNF (Figure 3b and Extended Data Fig. 7b) along with several other pathways. The authors should discuss why they focused only on TNFSF15 and not other factors. If they followed up on any other genes /pathways with no association found, those should be outlined or mentioned.
a.Primarily the authors had access to or created various cell lines to test. XPO1 knockdown in lines with high endogenous expression (NCI-H1688, NCI-H196, and DMS114) or overexpression of XPO1 in cell lines with low endogenous protein expression (NCI-H1048 and DMS273). They use NCI-H1688 and NCI-1048 primarily in most studies as prototypical examples. However, they also use additional cell lines in some experiments but not all (e.g., NCI-H196 identify TNFSF15 as a DEG). Given that these cell lines are representative SCLC subtypes under different transcriptional control, it would be ideal to consistently evaluate all different cell lines or provide justification for the selective use.
b.In Extended Data Figure 6a – Include what A/N/P/I denote for the cell lines listed (SCLC subtypes: SCLC-A, SCLC-N, SCLC-P, and SCLC-I?). Normalize to GAPDH and plot XPO1 levels for relative quantitation (e.g., NCI-H1688 vs NCI-

H1048)

5)Given the unimodal nature (single peak) of histograms, it is suggested that Fig 6 C, D, G should be represented as gMFI instead of % positive cells (also Extended Data Figure 6). Fluorescence intensity would be a more precise and informative metric.
6)It is important for the authors to include detailed information about Selinexor -where was it purchased /procured from and how was it formulated and dosed. Please include all the relevant details for anyone else trying to reproduce the results or extend their findings. Did the authors test the pharmacokinetics of Selinexor (as a single agent or in combination) in their in-vivo models? If so, the details and data should be included.
7)The authors use ubiquitin mutants (K48R/K63R) and functional assays to infer that TRIM21 catalyzes the K48-linked ubiquitination of IRF3 at lysine 313. Additional details of the constructs used in these experiments would be helpful.
a.It becomes critical to list the technical replicate experiments conducted to confirm reduction in ubiquitination with K313R mutant. The blot data presented in Figure 5J (and original blot images attached in the Supplemental data package) is smeared at this particular lane and makes it difficult to interpret.
b.In fact, it would make the data more definitive if the authors had done mass-spectrometry studies to evaluate ubiquitinated lysine (di-Gly peptide).
c.The use of linkage-specific reagents (e.g., K48 specific Ub antibody) or additional constructs (replacing other lysines except for K48) would be helpful to solidify this data.
d.Include additional details including the enzyme used in the in vitro ubiquitination assay. The E2 enzyme identity often directs linkage type and is important for reproducibility.

8)Selinexor causes TRIM21 nuclear accumulation and XPO1 overexpression increases cytoplasmic TRIM21. Have the authors evaluated whether TRIM21 contains a canonical NES or if TRIM21 is exported as a complex? Use of other canonical nuclear export inhibitor (other than Selinexor) would have been helpful to clarify this point.
9)Explicitly discuss any sample bias with the SCLC clinical samples and how this might impact generalizability of conclusions.
a.The authors could validate the association between XPO1/TREM2/class I in independent public datasets. This would add strength to this manuscript.

10)To ensure statistical robustness, please report exact n (biological replicates) for all experiments, tests done, if any, for normality, and state whether data met test assumptions, In addition, use appropriate multiple testing correction (FDR) for high-dimensional data and report exact p-values, confidence intervals, and when appropriate, effect sizes.

Other comments:
1) In Figure 1e, Y axis – is it SEM or SD
2) Extended Data Figure 2h: Y-axis relabel as “Proportion of non-tumor cells” instead of “Proportion in immune cells”
3) Supplementary Table 5 – correct huaman as human
4) Two anti-human XPO1 listed from CST – Cat # 4302 should be corrected as anti-IRF3 instead anti-XPO1
5) In Supplementary Methods file, line 26 “neutralizing antibody against human TNFSF15 (Cat. 78811, BPS Bioscience) is noted. However, BPS Biosciences Cat. 78811 is aTL1A repsonsive Jurkat reporter line. Please correct this error.
6) Fig 8: degration to degradation

Reviewer #3

(Remarks to the Author)
In the current manuscript authors explore the role of XPO1 inhibition in SCLC models. The subcutaneous tumors shown in figure 1 are treated with selinexor. It would be useful to have information on the dosage and administration of the drug at least in the figure legend. Does the cell line used express XPO1 as analyzed by WB or IHC?. Some of the results shown using scRNAseq should be validated using IHC to show differences in the infiltration by macrophages and their phenotype. In figure 1H the colors seems not to be coded correctly since the green observed in the Selinexor group is not included in the legend. In figure 1i the differences are hard to see, maybe different coding would be more useful.
In figure 2 (or figure legend) add information on the co-culture system, showing the length of the experiment.
The difference in growth in the nude mice caused by XPO-1 KO or overexpression is shown only in day 28 of the experiment. Being the bioluminescence system designed to allow the longitudinal analysis of tumoral growth, authors should show the complete data and how the different tumors grow over time. Also, to better evaluate the infiltration by macrophages in the orthotopic mouse model, information on the infiltration of the macrophages by IHC should be added to figure 2.
Authors mention that treating mice with Selinexor had no observable toxicity, which is in contrast to other reports, and clinical data, with Selinexor inducing weight loss. How are the doses of Selinexor and anti-PD-1 chosen in this setting? In the survival experiment, it would be interesting to see the changes in the tumoral microenvironment by IHC when mice succumb to the tumors. The survival of humanized mice using human PBMCs has been described to be no longer than 8 weeks, here the animals live way longer, can the authors provide details on the mechanisms that avoid the GvHD effect? The confirmation of the results using syngeneic/immunocompetent mice models of SCLC would made the conclusions more solid.

Minor
Please label the figures including figure number.

Version 1:

Reviewer comments:

Reviewer #2

(Remarks to the Author)
Commending the authors for their exhaustive efforts in revising this manuscript. It is clear that significant time and energy were devoted to addressing the reviewers' feedback. The inclusion of critical new data, most notably the second in-vivo replicate and the syngeneic model addresses previous gaps in the study's validation. This high level of responsiveness has resulted in a much more impactful and scientifically sound paper.

Reviewer #3

(Remarks to the Author)
I appreciate the changes and additional experiments performed by the authors. However, further changes and additional data would be desirable for clarity.

The expression of XPO1 by WB in the cell lines should be tested in an experiment including a positive control.
All IHC analysis should include a pan-macrophage marker and images showing different amplifications.

Regarding the addition of longitudinal bioluminiscence data, I appreciate the new data showed. However, the results would be easier to interpret if the different groups compared were included in the same plot and appropriate statistical test applied. If no significant differences are observed, it should be stated in the main text.

Reviewer #4

(Remarks to the Author)
The authors have successfully addressed all of my comments. The article has been significantly strengthened. I recommend the article for publication.

Version 2:

Reviewer comments:

Reviewer #3

(Remarks to the Author)
Authors have addressed all the comments in the previous review.

Open Access This Peer Review File is licensed under a Creative Commons Attribution 4.0 International License, which permits use, sharing, adaptation, distribution and reproduction in any medium or format, as long as you give appropriate credit to the original author(s) and the source, provide a link to the Creative Commons license, and indicate if changes were made.
In cases where reviewers are anonymous, credit should be given to 'Anonymous Referee' and the source.
The images or other third party material in this Peer Review File are included in the article's Creative Commons license, unless indicated otherwise in a credit line to the material. If material is not included in the article's Creative Commons license and your intended use is not permitted by statutory regulation or exceeds the permitted use, you will need to obtain permission directly from the copyright holder.
To view a copy of this license, visit https://creativecommons.org/licenses/by/4.0/

Dear Reviewers:

We sincerely thank the Reviewers for the careful and constructive evaluation of our manuscript, entitled "**Targeting TRIM21 nuclear export reprograms TREM2$^+$ macrophage polarization to augment anti-PD-1 efficacy in small cell lung cancer.**" We are grateful for the opportunity to revise our work.

In this point-by-point response letter, the original comments from the review are presented in black, our responses are highlighted in blue, and the corresponding changes marked in the manuscript are indicated in red.

Point-by-point responses to Reviewers' comments

Reviews comments：

Reviewer #1：

Strengths:

Connects a tumor intrinsic feature to TME alteration

Investigates macrophage role in SCLC, which is underexplored.

The proposed XPO1–TRIM21–IRF3 axis controlling TNFSF15 appears to be novel.

Detailed biochemical analyses.

Comment 1: How does XPO+/- subgroups relate to the known SCLC subtypes?

Response: We thank the Reviewer for this important question. We agree that clarifying the relationship between XPO1-high/XPO1-low tumors and established SCLC transcriptional subtypes is important for interpreting the generalizability of our findings.

To address this, we re-analyzed publicly available SCLC transcriptomic datasets and annotated tumors according to the reported subtype framework (including SCLC-A, SCLC-N, SCLC-P, and SCLC-Y, depending on dataset annotation). Across these datasets, XPO1 expression was distributed across multiple subtype categories and did not show significant enrichment in any single subtype ($p = 0.6412$ and $p = 0.8799$) (Fig. R1a,b). Consistently, in

the GSE60052 dataset, XPO1 mRNA expression did not significantly correlate with the canonical neuroendocrine lineage factors ASCL1 (Pearson r = 0.1505, p = 0.1854) or NEUROD1 (Pearson r = -0.1658, p = 0.1443) (Fig. R1c,d).

These results indicate that XPO1-high/XPO1-low status is not confined to a single established molecular subtype and therefore should not be considered a surrogate marker for any single subtype. Rather, XPO1 appears to represent a cross-subtype feature of SCLC.

Changes in the manuscript: The revised content has been added to page 4 lines 24-29.

"Re-analysis of publicly available SCLC transcriptomic datasets showed that XPO1 expression was uniformly distributed across the major SCLC subtypes (SCLC-A, SCLC-N, SCLC-P, and SCLC-Y), without significant enrichment in any specific subtype (Supplementary Fig. 1a,b). Additionally, XPO1 mRNA expression did not correlate significantly with the NE lineage markers ASCL1 (Pearson r = 0.1505, p = 0.1854) or NEUROD1 (Pearson r = -0.1658, p = 0.1443) in the GSE60052 dataset (Supplementary Fig. 1c,d), suggesting that XPO1 expression is a cross-subtype feature of SCLC rather than being subtype-specific."

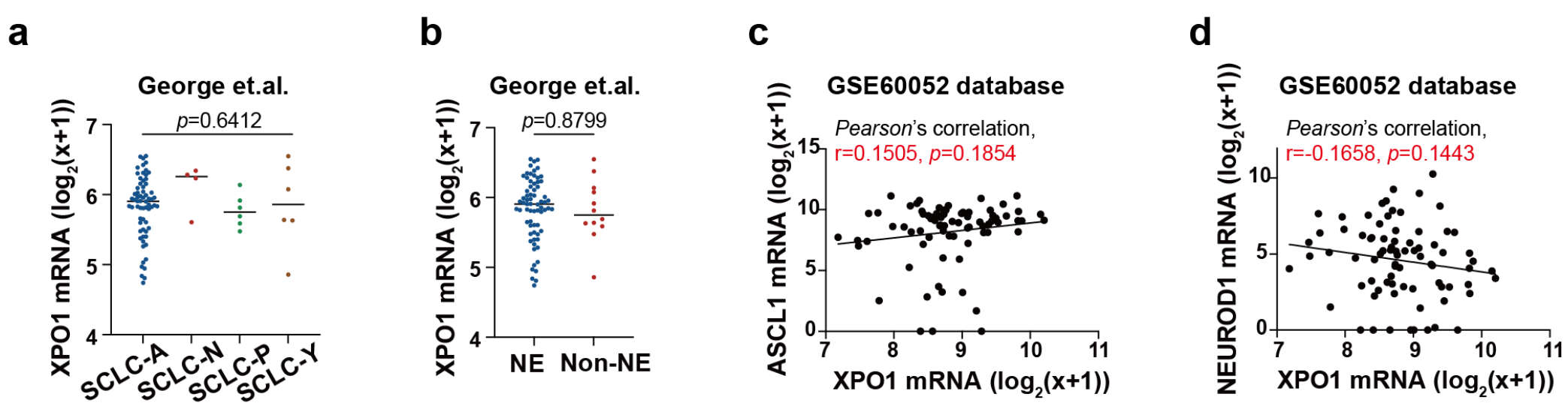

Fig. R1. **a** XPO1 mRNA expression across different SCLC molecular subtypes, including SCLC-A, SCLC-N, SCLC-P, and SCLC-Y, in the George et al. cohort. **b** XPO1 mRNA expression in neuroendocrine (NE) and non-neuroendocrine (Non-NE) tumours in the George et al. cohort (n = 81 samples in total). **c,d** Pearson correlation analysis of XPO1 mRNA expression with the canonical neuroendocrine lineage factors ASCL1 (**c**) and NEUROD1 (**d**) in GSE60052 (n = 79 samples in total). Data in **a** was determined by one-way ANOVA. Data in **b** was compared using a two-tailed unpaired t-test. For **c,d** correlations were assessed using Pearson's correlation analysis. Data are presented as mean ± s.d. Source data are provided as a Source Data file.

Comment 2: The models used are P subtype cell line, which is a rare IO unresponsive subtype. Would be important to verify key findings in a more generalizable cell line

Response: We thank the reviewer for this important point and agree that validation in a more generalizable cell line is essential.

Our mechanistic studies were designed around models with contrasting endogenous XPO1 expression rather than around a single transcriptional subtype. The two primary models used in the study, NCI-H1688 and NCI-H1048, were selected because they provide a robust contrast in XPO1 abundance. In addition, as shown in the original manuscript, key mechanistic features of the XPO1–TRIM21–IRF3–TNFSF15 axis were also validated in additional SCLC models, including DMS114 (SCLC-Y) and NCI-H196 (SCLC-Y).

To directly address the concern regarding subtype generalizability, we have now extended the functional validation to DMS273, an SCLC-N cell line (Fig. R2a). In the revised manuscript, we show that XPO1 overexpression in DMS273 cells promoted the emergence of $CD206^{+}$ & $TREM2^{+}$ macrophages in co-culture systems using both THP-1-derived macrophages and primary human PBMC-derived macrophages (Fig. R2b,c). In the same model, RT–qPCR and ELISA analyses confirmed that XPO1 reduced TNFSF15 expression at both the transcript and secreted-protein levels (Fig. R2d). In addition, ubiquitylation and protein stability assays showed that XPO1 overexpression enhanced TRIM21-dependent ubiquitylation and degradation of IRF3 (Fig. R2e-g), consistent with the mechanism observed in the original models.

Together, these new data demonstrate that the key tumor cell–intrinsic and macrophage-polarizing effects of XPO1 are reproducible in an additional, more generalizable SCLC subtype context, and are therefore not confined to the SCLC-P model.

Changes in the manuscript: The revised content has been added to page 6 lines 21-25.

"To assess whether this phenotype was reproducible in a more generalizable subtype context, we extended the analysis to DMS273 cells. Again, XPO1 overexpression in DMS273 cells promoted the emergence of $CD206^{+}$ & $TREM2^{+}$ macrophages in both THP-1-derived and PBMC-derived co-culture systems (Supplementary Fig. 4e,f). These results demonstrate that tumor-intrinsic XPO1 serves as a conserved orchestrator of $TREM2^{+}$ macrophage polarization."

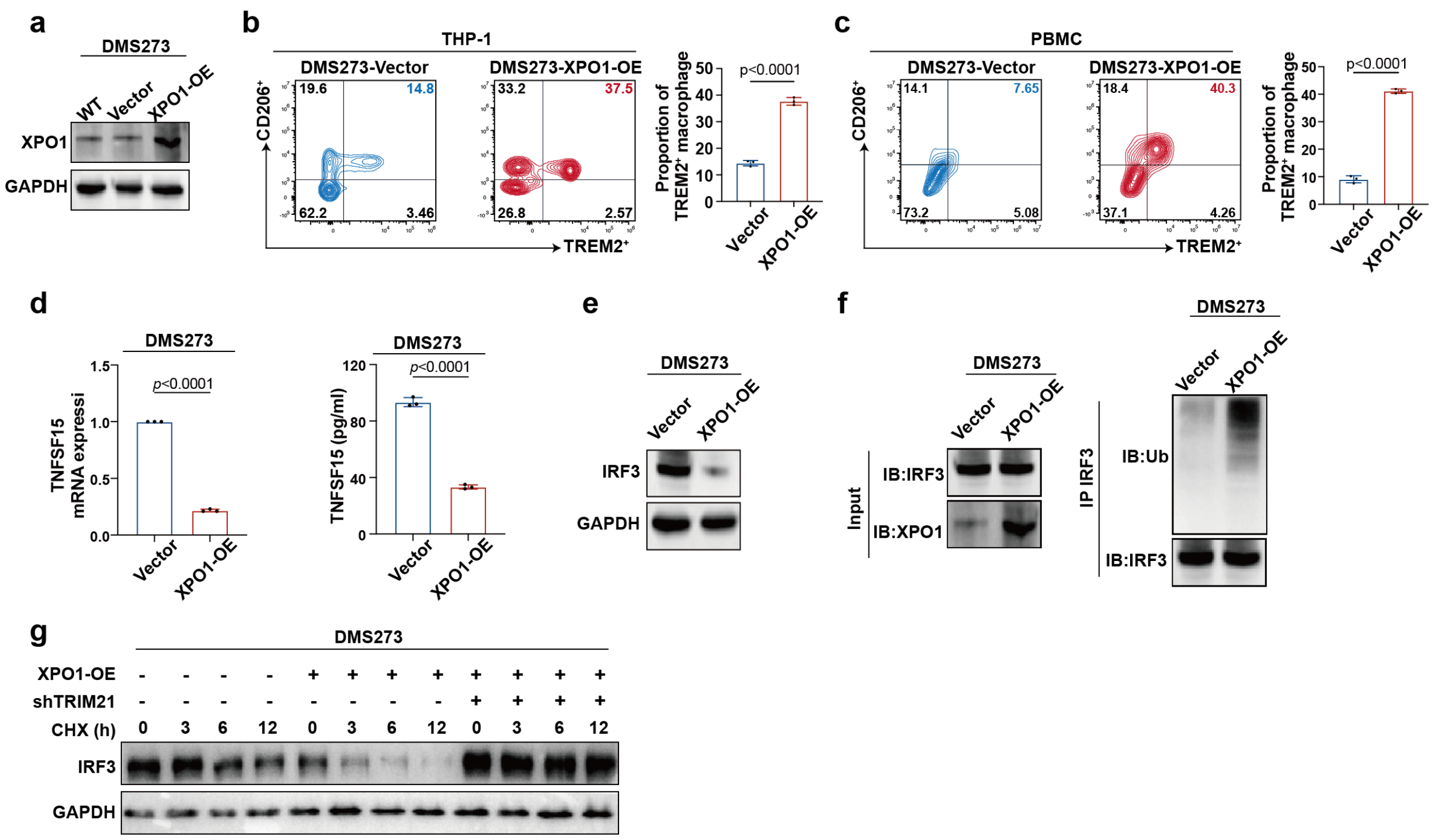

Fig. R2. a Western blot analysis of XPO1 expression in SCLC cell lines transduced with lentiviral constructs expressing XPO1 (XPO1-OE) constructs. **b** Flow cytometric analysis of TREM2 and CD206 surface expression on THP-1-derived macrophages co-cultured with the indicated DMS273 cells. Representative flow cytometry plots (left) and quantification (right) are shown. **c** Flow cytometric analysis of TREM2 and CD206 surface expression on PBMC-derived macrophages co-cultured with DMS273 cells. Representative flow cytometry plots (left) and quantification (right) are shown. **d** qRT–PCR and ELISA analyses of TNFSF15 mRNA expression and secreted TNFSF15 protein levels, respectively, in DMS273 cells with XPO1 overexpression. **e** Immunoblot analysis of IRF3 protein levels in DMS273 cells with XPO1 overexpression. **f** Ubiquitination assay in XPO1-overexpression SCLC cells. Cells were treated with MG132 for 5 h, followed by immunoprecipitation with an anti-IRF3 antibody and immunoblotting with an anti-ubiquitin antibody to detect polyubiquitinated IRF3. **g** TRIM21 knockdown attenuated IRF3 degradation induced by XPO1 overexpression. DMS273-vector/XPO1-OE cells were infected with control lentivirus or lentivirus expressing shTRIM21 for 48 h and then treated with CHX for the indicated times, followed by immunoblotting for IRF3 and GAPDH. Representative blots are shown. Data in **b-d** were analyzed using a two-tailed unpaired t-test. Data are from n=3 biologically independent experiments. Data in b-d are presented as mean ± s.d. Source data are provided as a Source Data file.

Comment 3: Selinexor has many off target effects, so would be important to do the key IO combo experiment with more selective agents or XPO KO.

Response: We thank the Reviewer for this important point and agree that Selinexor, as a pharmacologic XPO1 inhibitor, may exert effects beyond XPO1 blockade. We therefore performed the key immunotherapy combination experiment using a genetic XPO1-loss model in an immunocompetent syngeneic orthotopic setting.

Specifically, we established orthotopic lung tumors in C57BL/6 mice using RP116 cells with stable XPO1 knockdown, and evaluated the effect of anti-PD-1 treatment with serial in vivo bioluminescence imaging (Fig. R3a). In this model, the XPO1sg + anti-PD-1 group showed markedly greater tumor suppression than either treatment alone or the corresponding control groups (Fig. R3b). Consistent with this, the combination group also showed the longest survival (Fig. R3c).

To further assess whether the immune microenvironmental effects observed with Selinexor were recapitulated in the genetic model, we analyzed endpoint tumor tissues by IHC. These analyses showed that the XPO1sg + anti-PD-1 group had significantly reduced $TREM2^+$ macrophages, increased $CD86^+$ macrophages, increased $CD8^+$ T-cell infiltration, and increased MHC I expression, compared with the control groups (Fig. R3d,e). Thus, the key tumor-control and immune-remodeling effects observed in the pharmacologic combination setting were reproduced using genetic XPO1 suppression.

Together, these data may address the Reviewer's concern by demonstrating that the enhancement of anti-PD-1 efficacy is not solely dependent on Selinexor as a pharmacologic agent, but can also be reproduced by genetic XPO1 inhibition in vivo.

Changes in the manuscript: The revised content has been added to page 13 lines 30-31 and page 14 lines1-9. "To determine whether the effects of pharmacologic XPO1 inhibition could be recapitulated by genetic XPO1 suppression in an immunocompetent setting, we established syngeneic SCLC allograft tumors in C57BL/6 mice using either control or XPO1-knockdown RP116 cells, followed by treatment with an anti-PD-1 antibody (Fig. 7a). Longitudinal bioluminescence imaging (BLI) showed that XPO1 knockdown significantly enhanced the antitumor effect of PD-1 blockade, resulting in a greater reduction in tumor burden than either XPO1 knockdown alone or anti-PD-1 treatment alone (Fig. 7b). Consistent with this finding, mice in the combination group showed the most favorable survival outcome among all groups (Fig. 7c). IHC analysis of endpoint tumors further revealed decreased $TREM2^+$ macrophage infiltration, increased $CD86^+$ macrophage infiltration, enhanced $CD8^+$ T-cell infiltration, and elevated MHC class I expression in the combination group (Fig. 7d,e). Together, these results

provide in vivo evidence that XPO1 contributes to shaping the immune microenvironment of SCLC and suggest that XPO1 targeting may improve the therapeutic response to PD-1 blockade.”

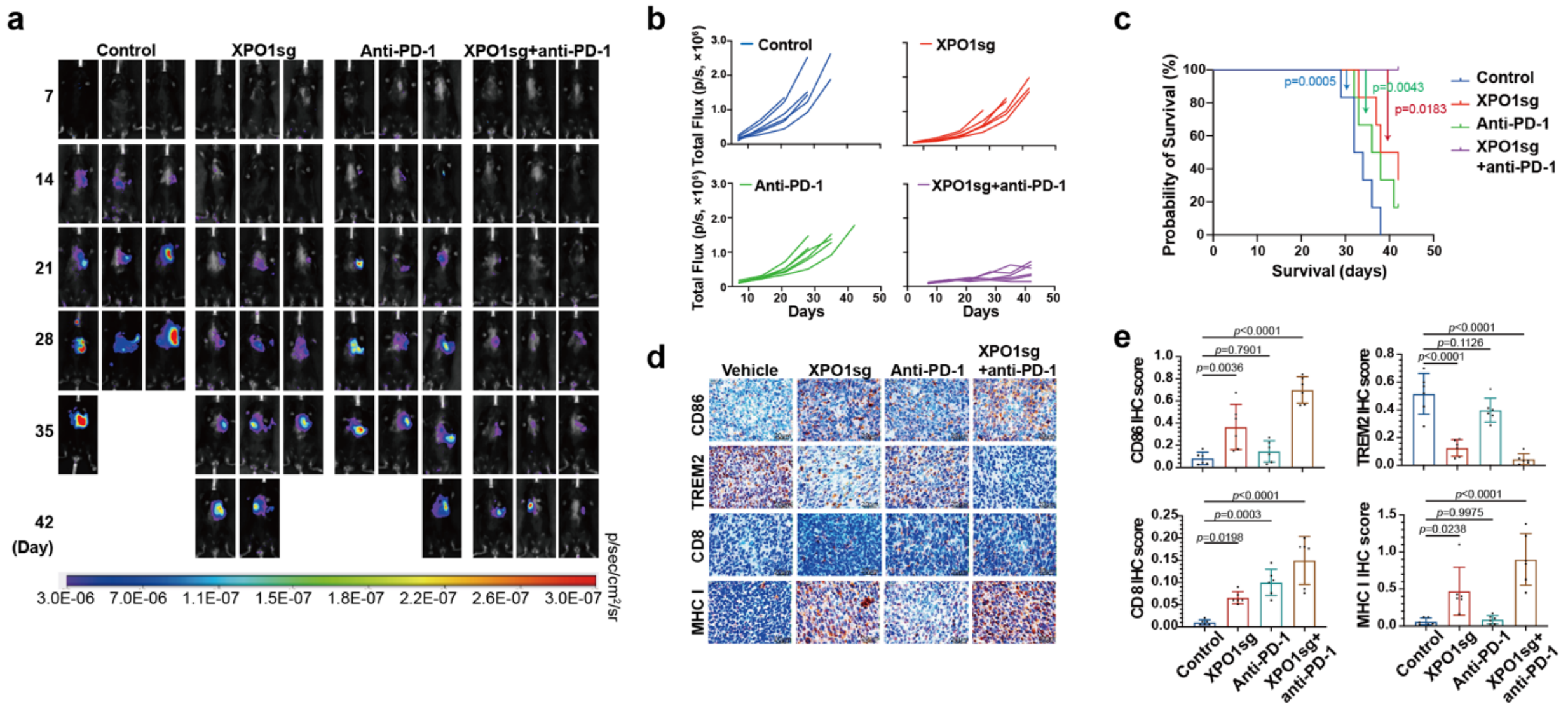

Fig. R3. XPO1 loss sensitizes syngeneic SCLC allograft to anti-PD-1 therapy. a,b Mice bearing orthotopic allografts of RP116-luc cells transduced with XPO1-silencing or vector-control lentivirus were treated by intraperitoneal injection with anti-PD-1 antibody (200 μg/injection, i.p., q3d) or isotype control antibody. Representative bioluminescence images acquired from day 7 to day 42 (**a**) and longitudinal quantification of bioluminescence signal intensity (**b**) are shown. **c** Kaplan–Meier analysis of survival in mice from the indicated groups. **d** Representative immunohistochemistry images of TREM2, CD86, CD8, and MHC I staining in xenograft SCLC tissues from the indicated groups. Scale bar, 20 μm. **e** Quantification of the staining shown in **d**. Data in **c** were compared using the two-sided log-rank test. Data in **e** were assessed by one-way ANOVA. Data are presented as mean ± s.d. Data are from n = 6 mice per group. Source data are provided as a Source Data file.

Specific comments:

Comment 4: Fig 1 I. Add canonical nonNE genes – REST, YAP, MYC etc;

Response: We thank the reviewer for this insightful suggestion. To address it, we expanded our analysis to include canonical non-neuroendocrine (non-NE) markers, including REST, YAP1, and MYC. Single-cell transcriptomic profiling of tumors following XPO1 inhibition showed a reduction in several NE-associated genes, such as Chga, Bex1, and Syp (Fig. R4a). Notably, canonical non-NE markers were not consistently induced, and the overall neuroendocrine program remained stable (Fig. R4b).

This finding indicates that XPO1 inhibition does not trigger a clear NE-to-non-NE lineage switch under the conditions examined. One possible explanation is that the observed decrease in NE marker expression may reflect

a broader treatment-related suppression of tumour cell state rather than a true lineage switch. Importantly, rather than supporting a broad shift in SCLC lineage identity, our data suggest that the dominant consequence of XPO1 inhibition in this setting is tumour-cell-intrinsic rewiring of immunoregulatory programs that secondarily reshape the surrounding immune microenvironment. Mechanistically, this is reflected in the selective depletion of a $TREM2^{+}$ immunoregulatory macrophage population, which we traced to tumour-intrinsic regulation of the TRIM21 – IRF3 – TNFSF15 axis.

Changes in the manuscript: The revised content has been added to page 5 lines 9-12 and page 15 lines 5-18. “Within the tumour compartment, Selinexor treatment was associated with reduced expression of several NE-associated genes, including Chga, Syp, and Bex1 (Supplementary Fig. 1l); however, the overall NE program was not markedly altered (Supplementary Fig. 1m).”

“In our study, single-cell transcriptomic analysis showed that Selinexor treatment reduced the expression of several NE-associated genes, including Chga, Bex1, and Syp. However, the overall NE program remained unchanged, indicating that XPO1 inhibition does not drive a clear neuroendocrine-to-non-neuroendocrine lineage transition under the conditions examined here. Rather than supporting a broad shift in SCLC lineage identity, our data suggest that the dominant consequence of XPO1 inhibition in this setting is tumour-cell-intrinsic rewiring of immunoregulatory programs that secondarily reshape the surrounding immune microenvironment. Importantly, this does not exclude a role for XPO1 in SCLC lineage plasticity. It remains possible that XPO1 inhibition influences neuroendocrine cell-state transitions in a context-dependent manner, for example across distinct subtype backgrounds, treatment durations, or stages of tumour evolution. However, within the experimental framework used here, the more prominent and reproducible consequence of XPO1 inhibition was depletion of a $TREM2^{+}$ immunoregulatory macrophage population, which we traced mechanistically to tumour-cell-intrinsic regulation of the TRIM21-IRF3-TNFSF15 axis. These findings therefore position XPO1 not as a regulator of tumour proliferation or lineage features, but as a tumour-cell-intrinsic driver of immunosuppressive niche formation in SCLC.”

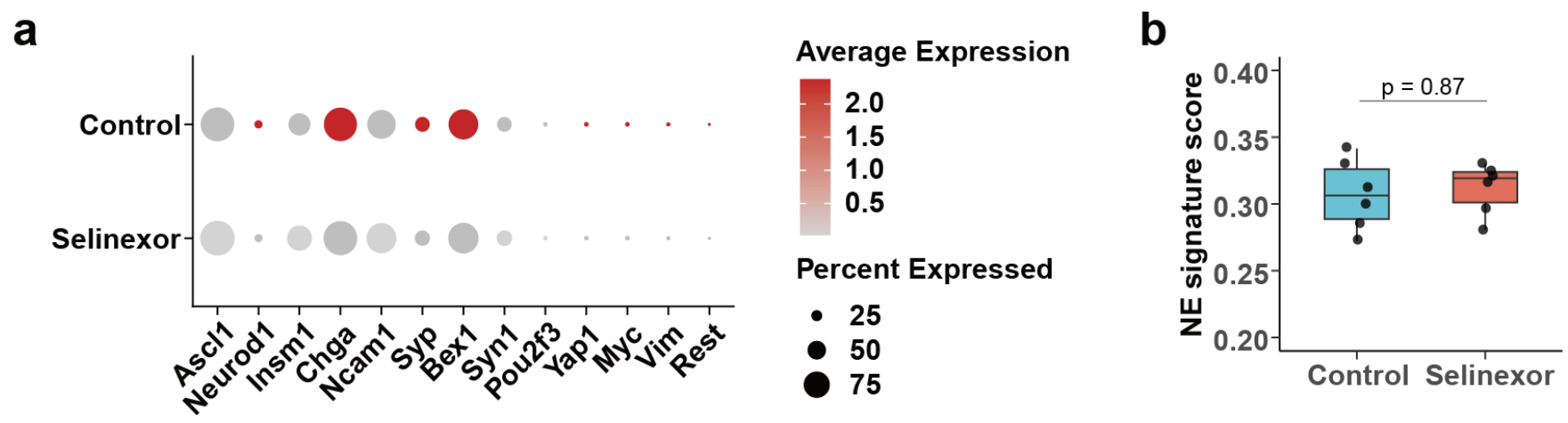

Fig. R4. a Dot plot showing the expression of canonical neuroendocrine and non-neuroendocrine lineage markers in tumour cells from control and Selinexor-treated SCLC allograft tumors. Dot size indicates the percentage of cells expressing each gene, and colour indicates the average expression level. Neuroendocrine-associated markers include Ascl1, Neurod1, Chga, Ncam1, Syp, Bex1, and Syt13, whereas canonical non-neuroendocrine markers include Pou2f3, Yap1, Myc, and Rest. **b** Comparison of neuroendocrine signature scores in tumour cells from control and Selinexor-treated SCLC allograft tumours. Signature scores were calculated using the UCell algorithm based on a curated set of NE-associated genes[1]. Data are from n = 6 mice per group. Source data are provided as a Source Data file.

Comment 5: Fig. 1k. show absolute number of macrophage subtypes (in addition to proportions)

Response: We thank the reviewer for this valuable suggestion. In the revised manuscript, we have included the absolute numbers of macrophage subtypes in addition to the proportions. Specifically, Fig. R5a now presents a bar chart displaying the absolute counts of each macrophage subtype across 12 samples.

Changes in the manuscript: The revised content has been added to Fig.1l.

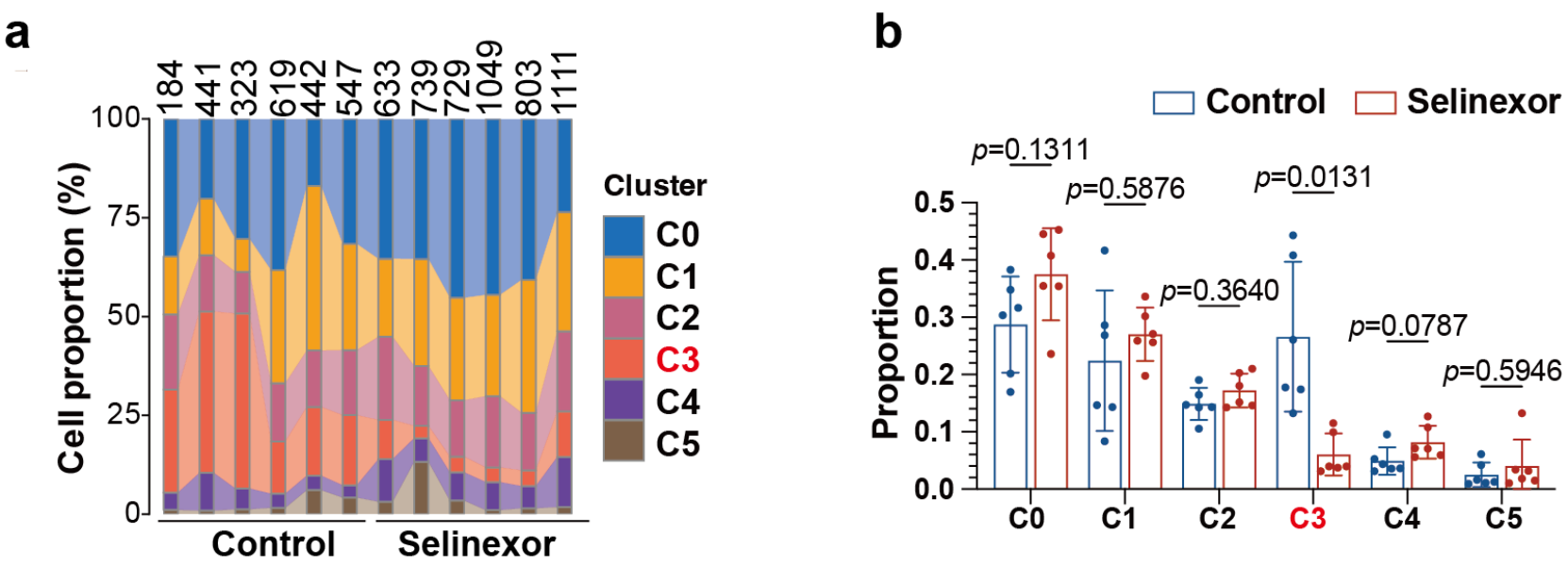

Fig. R5. a Proportions of macrophage clusters in control and Selinexor-treated tumors. Numbers above the plot indicate the total number of macrophages detected in each tumor. **b** Statistical comparison of the macrophage cluster proportions shown in **a**. Data were analysed by one-way ANOVA followed by Tukey's multiple-comparisons test. Data are from n = 6 mice per group. Data are presented as mean ± s.d. Source data are provided as a Source Data file.

Comment 6: Fig 1l. Show additional pathways to confirm that XPO blockade is indeed immunosuppressive (beyond APM). Please provide cluster costimulatory/inhibitory profiling to establish that TREM2^{+} population presents antigen in a tolerogenic manner.

Reponse: We thank the reviewer for this insightful suggestion. We agree that enrichment of antigen-presentation-related genes alone is insufficient to define the functional state of the TREM2^{+} macrophage population, and that broader pathway-level and cluster-level characterization is needed.

To address this point, we performed additional analyses using the expanded scRNA-seq dataset. Beyond antigen-presentation machinery (APM)-related programs, pathway analyses showed that cluster 3 (TREM2^{+}) macrophages were enriched for broader immune-regulatory programs, including lymphocyte-mediated immunity, regulation of T-cell activation, response to lipopolysaccharide, and regulation of lipid transport (Fig. R6a,b). At the cluster level, the TREM2^{+} macrophage population displayed a transcriptional profile consistent with a lipid-associated, immunoregulatory state. In addition to Mrc1 (CD206) and Trem2, this cluster co-expressed Gas6[2] and Maf[3], together with lipid metabolism-associated genes such as Apoe, Abcg1, Gpnmb, and Psap[4]. At the same time, these cells expressed antigen-processing genes including Ctss, Cstb, and Lgmn, while also expressing the inhibitory Fc receptor Fcgr2b[5] (Fig. R6c,d). We interpret this combination of features as being consistent with a tolerogenic-like antigen-presenting phenotype rather than a pro-inflammatory antigen-presenting state.

Importantly, to avoid overinterpretation based on transcriptomic features alone, we complemented these analyses with newly added functional assays. Purified TREM2^{+} macrophages produced higher levels of the immunosuppressive cytokines IL-10 and TGF-β compared with TREM2^{-} macrophages (Fig. R6e,f), and in co-culture assays, TREM2^{+} macrophages reduced the proportion of IFN-γ^{+} CD8^{+} T cells (Fig. R6g). Together, these data support the interpretation that the TREM2^{+} macrophage population identified in our system is functionally immunoregulatory and compatible with a tolerogenic mode of antigen presentation.

Accordingly, we have revised the manuscript to describe this population more precisely as a transcriptionally and functionally immunoregulatory macrophage state enriched for antigen-processing genes, rather than inferring function from APM enrichment alone.

Changes in the manuscript: The revised content has been added to page 5 lines 17-25, page 6 lines 27-31, and

page 7 lines 1-3 .

“By contrast, macrophage re-analysis revealed a more prominent and selective reduction in cluster 3 following XPO1 inhibition (Fig. 1k-m and Supplementary Fig. 2f). This population co-expressed Mrc1 (CD206) and Trem2, together with multiple genes previously associated with immunoregulatory macrophage states, including Gas6 and Maf, as well as genes involved in lipid metabolism and antigen processing and presentation (Fig. 1n and Supplementary Fig. 2g-j). Consistent with the single-cell data, immunohistochemistry (IHC) confirmed reduced TREM2$^+$ macrophage infiltration in Selinexor-treated tumours relative to controls (Fig. 1o). Given emerging evidence that TREM2$^+$ tumour-associated macrophages represent a functionally distinct immunosuppressive myeloid subset linked to T-cell dysfunction and resistance to immunotherapy, we therefore focused our subsequent analyses on this population.”

“To further assess the immunoregulatory role of TREM2$^+$ macrophages, we sorted TREM2$^+$ and TREM2$^-$ macrophages from co-culture systems. enzyme-linked immunosorbent assay (ELISA) and reverse transcription quantitative polymerase chain reaction (RT-qPCR) analyses of conditioned media revealed that TREM2$^+$ macrophages secreted higher levels of IL-10 and TGF-β compared to TREM2$^-$ macrophages (Supplementary Fig. 4g,h). Additionally, in co-culture assays with CD8$^+$ T cells isolated from PBMCs, TREM2$^+$ macrophages reduced the proportion of IFN-γ$^+$ CD8$^+$ T cells relative to TREM2$^-$ macrophages (Supplementary Fig. 4i). These findings collectively support the notion that TREM2$^+$ macrophages represent an immunoregulatory subset in the SCLC microenvironment.”

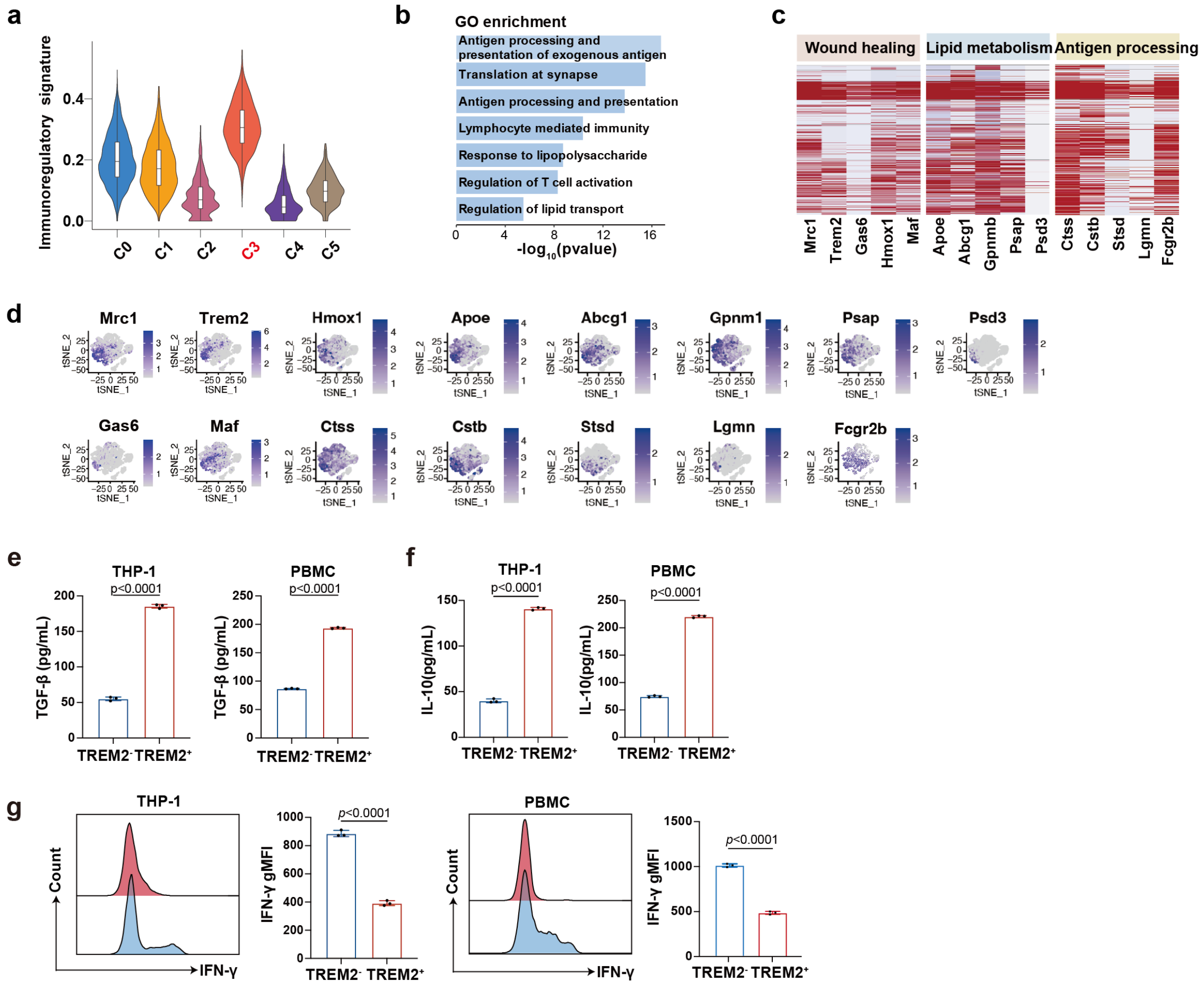

Fig. R6. a Violin plots showing immunoregulatory macrophage signature scores across macrophage clusters. Signature scores were calculated using the UCell algorithm based on a curated set of M2-like macrophage genes[6]. **b** Gene set enrichment analysis (GSEA) of genes enriched in cluster 3 macrophages, showing the top enriched immune-regulatory pathways ranked by false discovery rate (FDR) q value. **c** Expression of selected genes enriched in macrophage cluster 3 and associated with wound healing, lipid metabolism, and antigen processing. **d** Feature plots showing the expression of the genes highlighted in **c** across macrophage clusters. **e,f** ELISA analysis of IL-10 (**e**) and TGF-β (**f**) secretion by THP-1-derived macrophages (left) and PBMC-derived macrophages (right) after co-culture with the indicated SCLC cells. Culture supernatants were collected from sorted TREM2^{+} and TREM2$^{−}$ macrophage populations after 48 h of monoculture following cell sorting. **g** Flow cytometric analysis of IFN-γ expression in CD8^{+} T cells after co-culture with sorted TREM2^{+} or TREM2$^{−}$ macrophages. Purified human CD8^{+} T cells were pre-activated and then co-cultured with macrophages at a 1:1 ratio for 24 h. Representative flow cytometry plots are shown on the left, and quantification of the geometric mean fluorescence intensity (gMFI) is shown on the right. Data in **e–g** are from n = 3 biologically independent experiments. Data in **e–g** were analysed using two-sided unpaired t-tests. Data are presented as mean ± s.d. Source data are provided as a Source Data file.

Comment 7: Fig2. THP1 is a leukemia cell line which is used in coculture. Consider alternate source of macrophage to verify. H1048 is a Pou2f3 cell line, one of the few ones of this category of rare P subtype, which is inherently IO unresponsive. Consider a different cell line as well.

Response: We thank the Reviewer for this important suggestion and fully agree that validation using both an alternative macrophage source and an additional SCLC cell line would strengthen the generalizability of our findings.

To address the first point, we performed additional co-culture experiments using primary human PBMC-derived macrophages in parallel with THP-1-derived macrophages. Consistent with the original THP-1 co-culture system, XPO1 knockdown in NCI-H1688 cells reduced, whereas XPO1 overexpression in NCI-H1048 cells increased, the proportion of $CD206^+$ & $TREM2^+$ macrophages (Fig. R7a). These data indicate that the macrophage-polarizing effect of tumor-cell XPO1 is reproducible in a primary macrophage source and is not restricted to THP-1-derived macrophages.

To address the second point, we extended the analysis to DMS273, an SCLC-N cell line representing a more generalizable subtype context. In this model, XPO1 overexpression similarly promoted the emergence of $CD206^+$ & $TREM2^+$ macrophages in co-culture with both THP-1-derived and PBMC-derived macrophages (Fig. R2b,c). In addition, in DMS273 cells, XPO1 increased TNFSF15 expression at both the transcript and secreted-protein levels and enhanced IRF3 ubiquitination, consistent with the mechanism observed in the original models (Fig. R2d-g).

Together, these new data show that the effects of tumor-cell XPO1 on macrophage polarization are reproducible in both a primary macrophage system and an additional SCLC subtype, and are therefore not confined to the THP-1/H1048 co-culture setting.

Changes in the manuscript: The revised content has been added to page 6 lines 18-25.

"We further validated these findings using PBMC-derived macrophages. Consistent with the THP-1 co-culture system, XPO1 knockout in NCI-H1688 cells reduced, whereas XPO1 overexpression in NCI-H1048 cells increased, the proportion of $CD206^+$ & $TREM2^+$ macrophages (Supplementary Fig. 4d). To assess whether this phenotype was reproducible in a more generalizable subtype context, we extended the analysis to DMS273 cells.

Again, XPO1 overexpression in DMS273 cells promoted the emergence of CD206$^+$ & TREM2$^+$ macrophages in both THP-1-derived and PBMC-derived co-culture systems (Supplementary Fig. 4e,f). These results demonstrate that tumor-intrinsic XPO1 serves as a conserved orchestrator of TREM2$^+$ macrophage polarization."

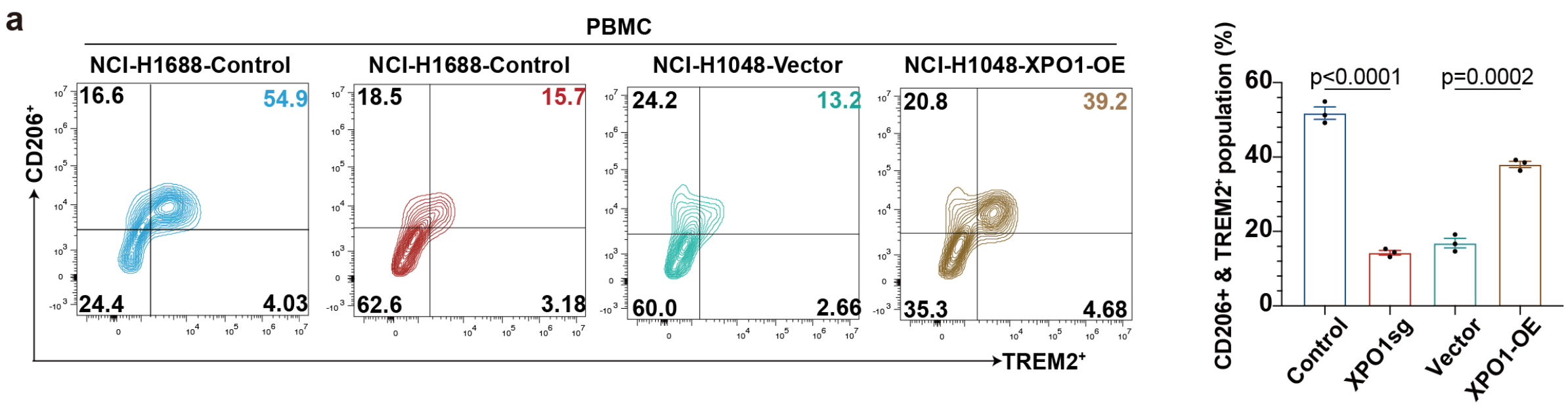

Fig. R7. a Flow cytometric analysis of TREM2 and CD206 surface expression on PBMC-derived macrophages co-cultured with the indicated SCLC cell lines. Representative flow cytometry plots (left) and quantification (right) are shown. Data were analyzed using a two-tailed unpaired t-test. Data are from n = 3 biologically independent experiments. Data are presented as mean ± standard deviation (s.d.). Source data are provided as a Source Data file.

Comment 8: In Extended 4 c . Error M1c instead of repeating M1a

Response: We sincerely thank the reviewer for pointing out the error in Extended Data Fig. 4c, we have labeled the cluster as Cluster 0 instead of repeating M1a.

Comment 9: Table 8: Please report both # targets (and % of peaks) and effect size (fold-enrichment/odds ratio with CI) against a GC/length-matched background, plus central-enrichment/footprinting.

Response: We thank the Reviewer for this important suggestion. In response, we substantially revised Supplementary Data 8 and added the requested enrichment and footprinting analyses to improve both transparency and quantitative interpretability.

First, for each enriched motif/transcription factor, we now report both the absolute number of target peaks containing the motif and the corresponding percentage relative to the full target peak set, providing a clearer description of motif prevalence across differentially accessible regions.

Second, we added effect-size estimates using a GC- and length-matched background. Background regions were

generated to match the target peak set with respect to both sequence length and GC content. Against this matched background, we now report fold-enrichment and odds ratios with 95% confidence intervals for each motif, allowing assessment of enrichment magnitude rather than statistical significance alone.

Third, aggregate ATAC-seq accessibility metaplots across the analyzed peak set showed central enrichment at peak centers and increased central accessibility in XPO1-deficient samples relative to controls (Fig. R8a,b). To more directly assess transcription factor occupancy, we performed bias-corrected ATAC footprinting using TOBIAS. These analyses revealed increased predicted IRF3 occupancy following XPO1 loss, with consistent footprint profiles across biological replicates. The genome-wide shift in IRF3 footprint score (Δ = -0.0800; 95% CI, -0.0818 to -0.0782; n = 22,716 IRF3 TFBSs) indicates a reproducible change in predicted TF engagement across a large set of sites. This scale of the absolute magnitude is typical for genome-wide ATAC footprinting analyses using TOBIAS[7]. The narrow confidence interval reflects low sampling variability across the large number of sites examined, and the direction of the change (negative Δ values indicating increased predicted occupancy in XPO1-deficient samples) is consistent with enhanced IRF3-dependent transcriptional activation observed in our functional assays.

Together, these analyses indicate that IRF3 is not only enriched at the sequence level within differentially accessible regions, but also shows increased positional occupancy, consistent with a functional role in mediating the transcriptional response to XPO1 inhibition (Fig. R8c,d).

Changes in the manuscript: The revised content has been added to page 9 lines 12-16 and Supplementary data 8.
"To identify candidate transcriptional regulators downstream of these chromatin changes, we next performed motif enrichment and bias-corrected transcription factor footprint analyses. IRF3 motifs were enriched in regions with increased accessibility, and TOBIAS-based footprint further showed enhanced predicted IRF3 occupancy following XPO1 loss (Fig. 3g and Supplementary Fig. 8a-d), implicating IRF3 may be as a candidate mediator of TNFSF15 activation."

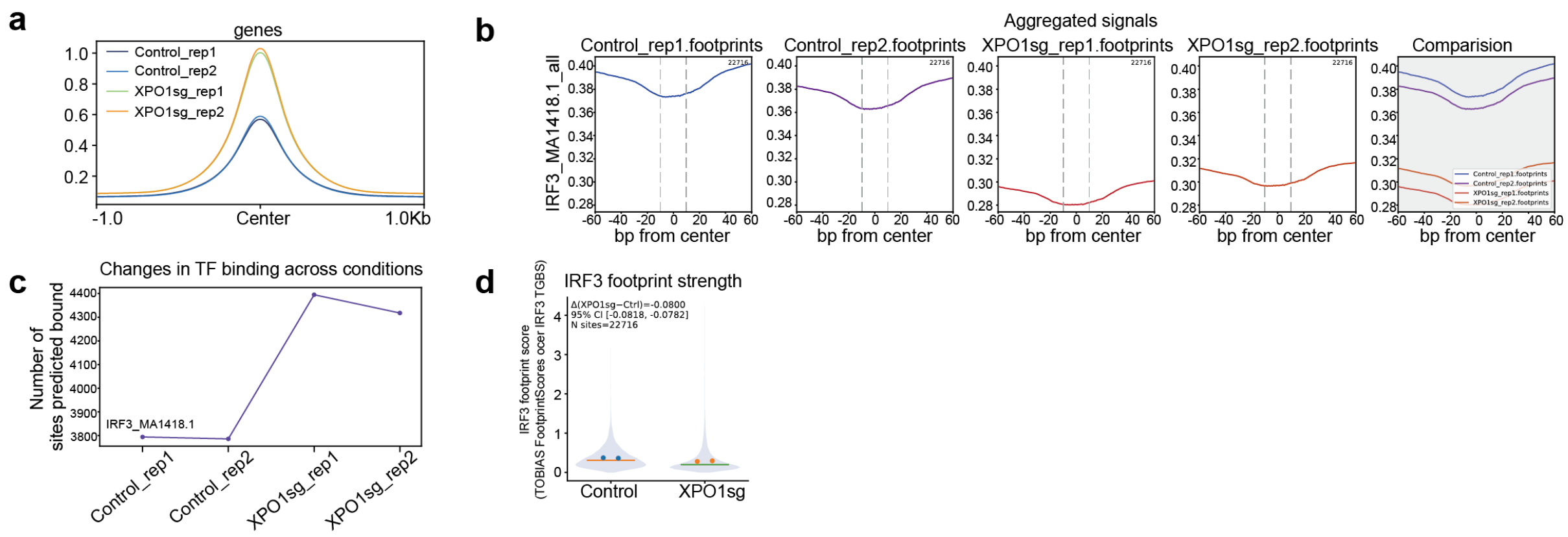

Fig. R8. **a** Aggregate ATAC-seq accessibility metaplot across the analyzed peak set in control and XPO1-deficient samples, showing central enrichment of accessibility and increased peak-center signal in XPO1-deficient samples. **b** Bias-corrected genome-wide ATAC footprinting profiles generated using TOBIAS, shown for individual biological replicates and aggregated signals, comparing control and XPO1-deficient samples. **c** Number of predicted IRF3-bound sites across biological replicates in control and XPO1-deficient samples. **d** IRF3 footprint scores at predicted IRF3 transcription factor-binding sites (TFBSs) in control and XPO1-deficient samples. XPO1 deficiency induces a shift in footprint score ($\Delta = -0.0800$; negative values indicate increased occupancy), consistent with enhanced IRF3 engagement and transcriptional activation of downstream targets including TNFSF15. Source data are provided as a Source Data file.

Comment 10: Fig 6 d: fludarabine and BAY 11-7082 have notable off-targets. To confirm STAT1 specificity with please provide one genetic perturbation (STAT1 or JAK1 siRNA), a clean JAK inhibitor control (ruxolitinib), IFN-γ neutralization in co-culture, and matched pathway readouts (pSTAT1↑, stable NF-κB).

Response： We thank the Reviewer for this important point and agree that the original use of fludarabine and BAY 11-7082 was not sufficiently selective to establish pathway specificity with confidence. To address this concern, we replaced these agents with more selective pharmacologic and genetic approaches and added the requested upstream neutralization and matched signaling readouts.

First, we introduced ruxolitinib as a selective JAK1/2 inhibitor and BMS-345541 as a selective IKK inhibitor to distinguish JAK/STAT from NF-κB pathway involvement. In the tumor–macrophage co-culture system, ruxolitinib abrogated the increase in tumor-cell MHC I induced by XPO1 knockdown, whereas BMS-345541 had no appreciable effect on this response (Fig. R9a). These data support a dominant role for JAK/STAT signaling,

rather than IKK/NF-κB, in mediating the co-culture-dependent MHC I induction.

Second, to directly test tumor-intrinsic STAT1 dependency, we performed siRNA-mediated STAT1 knockdown in SCLC cells. STAT1 silencing prevented the MHC I upregulation triggered by XPO1 knockdown in the co-culture setting, demonstrating that tumor-cell STAT1 is required for this response (Fig. R9b).

Third, to test whether IFN-γ is the relevant upstream cue in the co-culture system, we added an IFN-γ-neutralizing antibody. IFN-γ neutralization reduced tumor-cell p-STAT1 (Tyr701) and blocked the XPO1 knockdown-induced increase in MHC I, while p65 phosphorylation (Ser536) remained unchanged under the same conditions (Fig. R9c). These matched signaling readouts further support that the effect is mediated predominantly through an IFN-γ–JAK/STAT1 axis, with no corresponding evidence for enhanced NF-κB activation in this setting.

Together, these new pharmacologic, genetic, and neutralization experiments provide a substantially cleaner demonstration of pathway specificity and support the conclusion that macrophage-dependent MHC I upregulation in XPO1-deficient SCLC cells is driven predominantly by JAK/STAT1 signaling.

Changes in the manuscript: The revised content has been added to page 12 lines 18-29.

"Pretreatment of SCLC cells with ruxolitinib, a JAK1/2 inhibitor, significantly reduced the MHC class I upregulation induced by XPO1 loss in co-culture, whereas treatment with the IKK inhibitor BMS-345541 had no notable effect under the same conditions (Fig. 6e-g). Similarly, siRNA-mediated knockdown of STAT1 in SCLC cells diminished MHC class I induction following XPO1 loss in co-culture, reinforcing the tumor-cell-intrinsic role of STAT1 in this response (Supplementary Fig. 11d).

Given that STAT1 is a key mediator of interferon signaling, we next investigated the contribution of IFN-γ to this effect. Neutralizing IFN-γ with an antibody reduced tumor-cell pSTAT1 levels and attenuated the MHC class I increase observed with XPO1 loss, while p65 phosphorylation remained unchanged (Supplementary Fig. 11e). These findings support a dominant role for IFN-γ-JAK/STAT1 signaling in macrophage-dependent MHC class I upregulation in XPO1-deficient SCLC cells in co-culture. Further studies will be necessary to determine the cellular source and temporal dynamics of the interferon signal in this context."

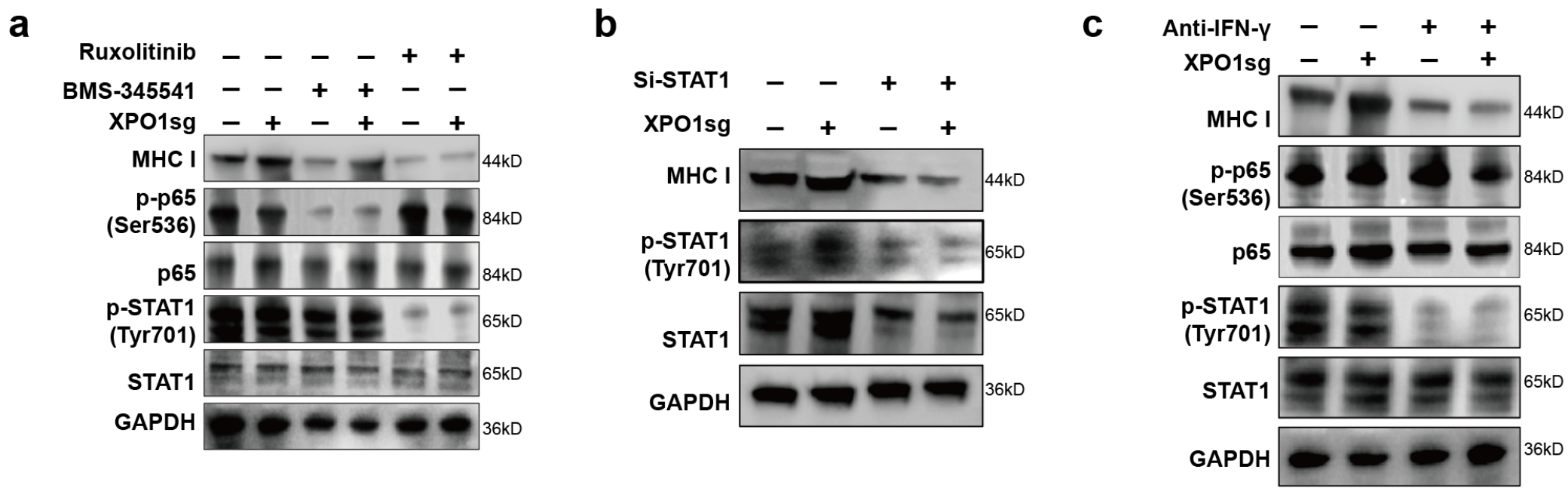

Fig. R9. a immunoblot of MHC I expression in control and NCI-H1688/XPO1-silenced cells co-cultured with THP-1-derived macrophages, with or without pretreatment with the JAK1/2 inhibitor ruxolitinib or the IKK inhibitor BMS-345541 for 24 h. **b** Immunoblot analysis of MHC I levels in the indicated SCLC cell lines co-cultured with THP-1-derived macrophages. **c** Immunoblot analysis of MHC I levels in the indicated SCLC cell lines co-cultured with THP-1-derived macrophages and treated with an IFN-γ-neutralizing antibody. Representative images are shown. Data are from n = 3 biologically independent experiments. Source data are provided in the Source Data file.

Comment 11: Fig. 7f shows good images but could reflect a single field. Please provide cohort-level quantification: per-patient XPO1 and MHC I H-scores, TREM2$^+$ and CD86$^+$ macrophage densities (and CD86/TREM2 ratio), CD8+ density and CD8–tumor distance, with violin/box plots (N, effect size, 95% CI), correlations, and a multivariable model for response. ROIs should be pre-specified, selected blinded to outcome, analyzed by automated segmentation, and stratified/adjusted for biopsy site. Include segmentation masks and raw counts in the supplement.

Response: We thank the reviewer for this valuable suggestion. We agree that cohort-level quantification is important to strengthen the robustness of our findings. In response, we performed per-patient quantification of tumor-cell XPO1 and MHC I H-scores, as well as TREM2$^+$ and CD86$^+$ macrophage densities, the CD86/TREM2 ratio, CD8$^+$ T-cell density, and CD8–tumor distance in tumor biopsies from advanced SCLC patients treated with ICIs. These data are now presented with violin/box plots together with the sample size, effect size, and 95% confidence intervals in the revised figures and supplementary materials.

Our analysis showed that, compared with non-responders, responders had lower XPO1 expression, higher MHC I expression, a lower TREM2$^+$/CD86$^+$ macrophage ratio, increased CD8$^+$ T-cell infiltration, and a shorter CD8–tumor distance (Fig. R10a-d). We have also included representative segmentation masks and raw cell-count

data in the Source data.

Multispectral images were acquired under identical settings across all samples. ROIs were pre-specified based on tissue quality and tumor content, selected blinded to clinical outcome, and analyzed using an automated segmentation pipeline, with manual review of a subset of images for quality control. We have added these details to the revised Methods.

We also agree that multivariable modeling would provide additional insight into the independent association of these variables with treatment response. Given the limited size of the current cohort (5 responders and 5 non-responders), we believe that such an analysis would be best interpreted as exploratory and would require validation in a larger dataset. Accordingly, in the present revision, we focused on robust per-patient quantification and group-wise comparisons, while recognizing that future studies in larger, independent cohorts will be important to enable multivariable modeling and more fully assess the predictive value of these features.

Changes in the manuscript: The revised content has been added to page 14 lines 11-20 and page 23 lines 6-15.

"To explore clinical relevance, we performed multiplex IHC in tumor biopsies from advanced SCLC patients treated with first-line ICIs (Supplementary data 4). Cohort-level analysis, including per-patient quantification of tumor XPO1 and MHC-I H-scores, as well as immune cell densities for $TREM2^+$ and $CD86^+$ macrophages and $CD8^+$ T cells, revealed that responders had significantly lower tumor XPO1 expression ($p = 0.0021$), higher MHC I expression ($p = 0.0268$), a reduced $TREM2^+/CD86^+$ macrophage ratio ($p = 0.0196$), and increased $CD8^+$ T-cell infiltration ($p = 0.0326$) compared to non-responders. Notably, responders also exhibited a significantly shorter distance between $CD8^+$ T cells and tumor cells ($p = 0.0203$, Supplementary Fig. S13a,b). These clinical findings support an association between high XPO1/low MHC I expression, a TREM2-skewed macrophage landscape, and reduced $CD8^+$ T-cell presence in non-responders. Together, these observations suggest that XPO1 contributes to an immune-evasive microenvironment in SCLC."

"Multispectral images were acquired using the Mantra Quantitative Pathology Imaging System (PerkinElmer, USA) under identical settings across all samples. For each case, ROIs were pre-specified based on tissue quality and tumor content and selected blinded to clinical response. Viable tumor areas were analyzed while excluding necrosis, hemorrhage, tissue folds, and other artifacts. At least four ROIs per case were captured using consistent acquisition parameters across the cohort. Cell segmentation and phenotyping were performed using an automated

pipeline in PANO, with nuclei identified by DAPI, tumor cells defined by PAN-CK, and immune cell subsets identified by marker combinations. The densities of TREM2^{+} macrophages, CD86^{+} macrophages, and CD8^{+} T cells, as well as tumor-cell MHC I expression, were quantified. The CD86/TREM2 ratio was calculated for each case. CD8-tumor distance was defined as the mean nearest distance from CD8^{+} T cells to tumor cells. Automated quantification results were further cross-validated by blinded manual review of a subset of images."

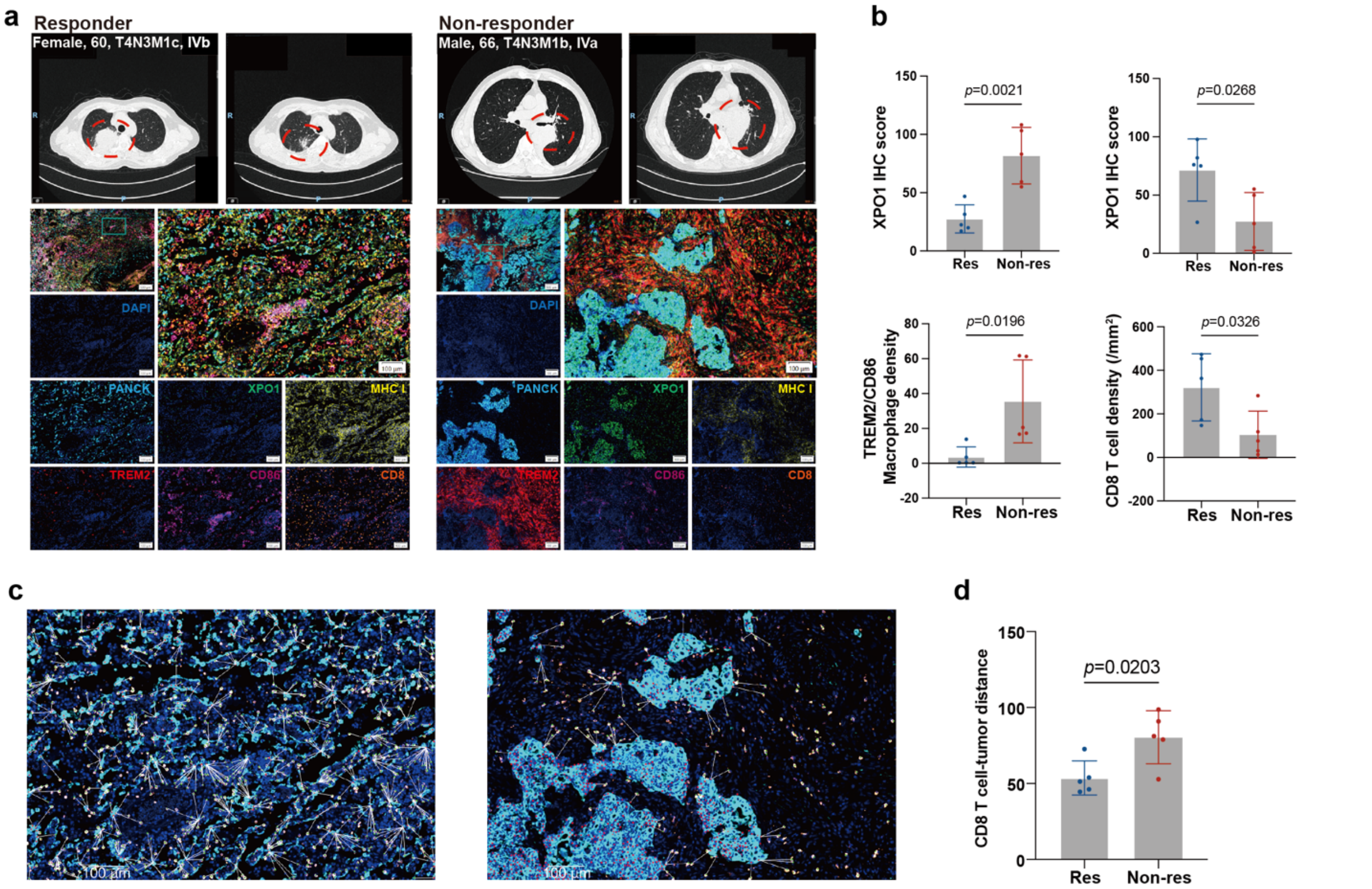

Fig. R10. a Top: representative chest computed tomography (CT) scans from patients with SCLC obtained before and after immune checkpoint inhibitor (ICI) therapy, stratified by clinical response. Bottom: multiplex immunohistochemistry analysis of XPO1, MHC I, TREM2, CD86, and CD8 in tumour samples from ICI responders (n = 5) and non-responders (n = 5). **b** Quantification of XPO1 and MHC I immunohistochemistry scores, TREM2^{+}, CD86^{+}, and CD8^{+} cell densities, and the nearest distance from CD8^{+} cells to tumour cells in the samples shown in **a**. **c** Representative images showing the distance between CD8^{+} T cells (yellow) and tumour cells (ice blue) in tumour samples from immune checkpoint inhibitor (ICI) responders (n = 5) and non-responders (n = 5). Arrows indicate the closest proximity between CD8^{+} T cells and tumour cells. Scale bar, 100 μm. **d** Quantification of the mean nearest distance from CD8^{+} T cells to tumour cells, as shown in **c**. Data in **b,d** were analysed using two-sided unpaired t-tests. Data are presented as mean ± s.d. Source data are provided as a Source Data file.

Reviewer #2 (Remarks to the Author):

This manuscript is an impressive attempt to understand and mechanistically clarify the immunoregulatory circuit of Exportin1 (XPO1). This is also a translationally relevant investigation for the treatment of small cell lung cancer indication especially with the limited success in the treatment of SCLC with anti-PD1 antibodies. The authors propose that XPO1 participates in the transport of TRIM21 from nucleus to cytoplasm. TRIM21 is presumed to be held in the nucleus when XPO1 transport is blocked with Selinexor reversing the ubiquitin ligase mediated degradation of IRF3 in the cytoplasm. The authors also use ATAC-Seq and ChIP assays to demonstrate that IRF3 binds to the promoter region of a distinct cytokine, TNFSF15. The recent increased interest in targeting TNFSF15 (TL1A) for immunotherapy amplifies the importance of this manuscript. Together, the collective data points to the role of XPO1 in transcriptional regulation of TNFSF15 through IRF3-mediated promoter activation and indirectly modulating macrophage functionality in SCLC TME.

Favorable evaluation/commendation:

1)The figures /data are convincingly able to link the proposed mechanism of action including TRIM21 cytoplasmic relocalization upon overexpression of XPO1 (Figures 4G-H), ubiquitination loss when using a point mutant (Figure 5J), and IRF3 ChIP at TNFSF15 promoter (Figure 3J)

a.Adequate data strength in demonstrating XPO1–TRIM21–IRF3 axis: Extended Data Figs. 11–13 show cycloheximide/MG132 chase assays, IP of endogenous TRIM21–IRF3, and ubiquitination blots. These provide stronger evidence that XPO1 promotes IRF3 degradation through TRIM21. Extended Data Fig. 16 shows Selinexor reversing TRIM21 nuclear export and reducing IRF3 ubiquitination, directly supporting the main mechanistic model.

2)The authors are able to bridge the molecular biology aspects with therapeutic relevance. IHC correlations of XPO1, TREM2, CD86, Class I in patient tumors, and functional synergy of XPO1 inhibition (Selinexor) in combination with anti-PD-1 in mouse models.

3)Using multiple orthogonal methods like scRNAseq, mouse in-vivo models, biochemical assays, and extending some observations with clinical samples, the authors add strength to their interpretation of results

Feedback for Manuscript Improvement

Comment 1: Previous reports have shown that XPO1 inhibition, specifically using Selinexor, has been shown to

skew the ratios of macrophage subsets within the tumor microenvironment (TME). Abundance shifts in TREM2/CD206-positive TAMs is not a direct evidence that these macrophages are suppressive in SCLC. The interpretation that these macrophages drive immune exclusion is based on correlative transcriptomic and IHC data.

Response: We thank the Reviewer for this important point. We agree that changes in the abundance of TREM2/CD206-positive tumour-associated macrophages, together with correlative transcriptomic and IHC analyses, are not in themselves sufficient to establish suppressive function in SCLC. We also agree that our original wording regarding immune exclusion was stronger than warranted by the evidence available in the initial submission.

To directly address this concern, we performed additional functional experiments using purified TREM2$^+$ and TREM2$^-$ macrophage populations isolated from the co-culture system. TREM2$^+$ macrophages produced higher levels of the immunoregulatory cytokines IL-10 and TGF-β than their TREM2$^-$ counterparts (Fig. R11a,b). In parallel, in co-culture assays with PBMC-derived CD8$^+$ T cells, TREM2$^+$ macrophages reduced the proportion of IFN-γ$^+$ CD8$^+$ T cells relative to TREM2$^-$ macrophages (Fig. R11c). These newly added data provide direct functional support that the TREM2$^+$ macrophage population identified in our system is not merely phenotypically distinct, but has immunoregulatory properties consistent with a suppressive macrophage state.

We additionally re-analysed the expanded scRNA-seq dataset. Beyond antigen-presentation-related programs, the Trem2$^+$ macrophage cluster was enriched for broader immune-regulatory pathways, including regulation of T-cell activation, lymphocyte-mediated immunity, response to lipopolysaccharide, and lipid transport (Fig. R11d,e). At the transcriptional level, this cluster co-expressed Trem2 and Mrc1 together with genes associated with lipid-associated and immunoregulatory macrophage states, including Gas6[2], Maf[3], Apoe, Abcg1, Gpnmb, and Psap[4], while also expressing antigen-processing genes such as Ctss, Cstb, and Lgmn, as well as the inhibitory Fc receptor Fcgr2b[5] (Fig. R11f,g). We therefore interpret this population as an immunoregulatory, lipid-associated macrophage state rather than a conventional pro-inflammatory antigen-presenting population.

Accordingly, we have revised the manuscript to avoid overstatement. Rather than stating that these macrophages drive immune exclusion, we now describe them more precisely as a functionally supported immunoregulatory macrophage population whose abundance and activity are consistent with a contribution to immune tolerance in

the SCLC microenvironment.

Changes in the manuscript: The revised content has been added to page 5 lines 17-27, page 6 lines 27-31, and page 7 lines 1-3.

"By contrast, macrophage re-analysis revealed a more prominent and selective reduction in cluster 3 following XPO1 inhibition (Fig. 1k–m and Supplementary Fig. 2f). This population co-expressed Mrc1 (CD206) and Trem2, together with multiple genes previously associated with immunoregulatory macrophage states, including Gas6[24] and Maf[25], as well as genes involved in lipid metabolism and antigen processing and presentation (Fig. 1n and Supplementary Fig. 2g-j). Consistent with the single-cell data, immunohistochemistry (IHC) confirmed reduced $TREM2^+$ macrophage infiltration in Selinexor-treated tumours relative to controls (Fig. 1o). Given emerging evidence that $TREM2^+$ tumour-associated macrophages represent a functionally distinct immunosuppressive myeloid subset linked to T-cell dysfunction and resistance to immunotherapy[26,27], we therefore focused our subsequent analyses on this population."

"To further assess the immunoregulatory role of $TREM2^+$ macrophages, we sorted $TREM2^+$ and $TREM2^-$ macrophages from co-culture systems. enzyme-linked immunosorbent assay (ELISA) and reverse transcription quantitative polymerase chain reaction (RT–qPCR) analyses of conditioned media revealed that $TREM2^+$ macrophages secreted higher levels of IL-10 and TGF-β compared to $TREM2^-$ macrophages (Supplementary Fig. 4g,h). Additionally, in co-culture assays with $CD8^+$ T cells isolated from PBMCs, $TREM2^+$ macrophages reduced the proportion of IFN-γ^+ $CD8^+$ T cells relative to $TREM2^-$ macrophages (Supplementary Fig. 4i). These findings collectively support the notion that $TREM2^+$ macrophages represent an immunoregulatory subset in the SCLC microenvironment."

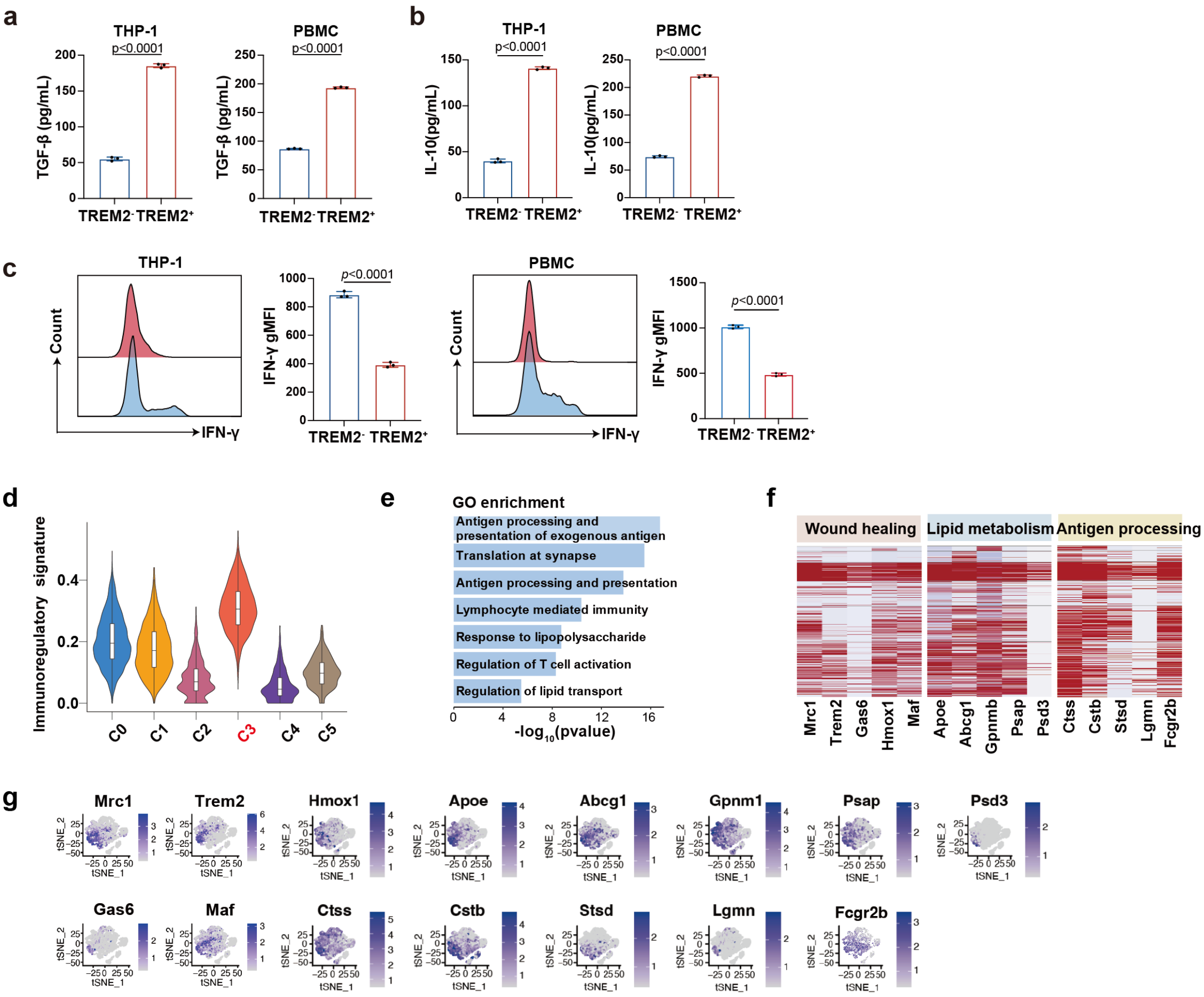

Fig. R11. a,b ELISA analysis of IL-10 (**a**) and TGF-β (**b**) secretion by THP-1-derived macrophages (left) and PBMC-derived macrophages (right) after co-culture with the indicated SCLC cells. Culture supernatants were collected from sorted TREM2$^+$ and TREM2$^-$ macrophage populations after 48 h of monoculture following cell sorting. **c** Flow cytometric analysis of IFN-γ expression in CD8$^+$ T cells after co-culture with sorted TREM2$^+$ or TREM2$^-$ macrophages. Purified human CD8$^+$ T cells were pre-activated and then co-cultured with macrophages at a 1:1 ratio for 24 h. Representative flow cytometry plots are shown on the left, and quantification of the geometric mean fluorescence intensity (gMFI) is shown on the right. **d** Violin plots showing immunoregulatory macrophage signature scores across macrophage clusters. Signature scores were calculated using the UCell algorithm based on a curated set of M2-like macrophage genes[4]. **e** Gene set enrichment analysis (GSEA) of genes enriched in cluster 3 macrophages, showing the top enriched immune-regulatory pathways ranked by false discovery rate (FDR) q value. **f** Expression of selected genes enriched in macrophage cluster 3 and associated with wound healing, lipid metabolism, and antigen processing. **g** Feature plots showing the expression of the genes highlighted in **c** across macrophage clusters. Data in **a–c** are from n = 3 biologically independent experiments. Data in **a–c** were analysed using two-sided unpaired t-tests. Data are presented as mean ± s.d. Source data are provided as a Source Data file.

Comment 2: The claim "XPO1 inhibition suppresses TREM2$^+$ macrophage differentiation in murine [syngeneic] tumors" is not substantiated with data. Specifically, XPO1 inhibition significantly reduced the proportion of "M2c" macrophages that co-express CD206 and TREM2. The pseudotime trajectory analyses (Fig. 1m) is suggestive of this but is not an experimental evidence for suppression of differentiation. PS: The broader consensus in the field is to avoid the use of M1, M2, etc. macrophage nomenclature

Response: We thank the Reviewer for this important point and agree that our previous wording was too strong. In particular, we agree that pseudotime trajectory analysis is inferential and does not constitute direct experimental evidence that XPO1 inhibition suppresses TREM2$^+$ macrophage differentiation. Our data support a selective reduction in the abundance of the CD206$^+$/TREM2$^+$ macrophage population following XPO1 inhibition in murine tumours, but do not justify a definitive claim regarding impaired differentiation per se. We have therefore revised the text throughout the manuscript to reflect this distinction more precisely.

We also agree with the Reviewer's point regarding macrophage nomenclature. In line with current practice in the field, we have removed M1/M2/M2c terminology throughout the manuscript and now refer to macrophage populations by cluster identity, marker expression, and transcriptional features. This change avoids overinterpretation and better reflects the resolution provided by the single-cell data.

Changes in the manuscript: The title on page 4, line 21 has been revised from: "XPO1 inhibition suppresses TREM2$^+$ macrophage differentiation in murine." to: "Selinexor treatment decreases TREM2$^+$ macrophages in murine SCLC allograft tumours."

a. Causal function needs to be experimentally evaluated or the language appropriately modified to indicate correlation or that the available data is suggestive. Transcriptomic signatures alone do not prove suppressive function. Note that the authors already show no variation in T cell (effector/memory/exhausted) subsets after XPO1 inhibition (Extended Data Fig. 3a-e)

Response: We thank the Reviewer for this important comment and agree that transcriptomic signatures alone do not establish suppressive function or causality. As described above in our response to Comment 1, we have now performed additional functional experiments showing that purified TREM2$^+$ macrophages display

immunoregulatory properties in vitro, including increased IL-10 and TGF-β production and suppression of IFN-γ expression in $CD8^+$ T cells (Fig. R11a-c). These data provide direct functional support for the interpretation of this population as immunoregulatory.

At the same time, we fully agree that these in vitro findings do not by themselves establish that this macrophage subset is solely responsible for the in vivo immune effects of Selinexor. We have therefore revised the manuscript throughout to distinguish more clearly between functional evidence, correlative in vivo observations, and inference from transcriptional state. Specifically, we have replaced causal or overinterpreted wording with more precise language such as “a macrophage population with an immunoregulatory transcriptional profile” or “features consistent with a lipid-associated, immunoregulatory macrophage state.”

We also appreciate the Reviewer’s point regarding the T-cell analysis. In the original scRNA-seq dataset, which included three tumours per group, we did not detect broad differences in T-cell subsets following Selinexor treatment. After expanding the cohort to six tumours per group, we observed a modest increase in the relative abundance of cytotoxic T cells in the Selinexor-treated group (Fig. R12a,b). We interpret this as a shift in T-cell state composition at improved sampling depth, rather than as direct evidence of T-cell functional reinvigoration. The revised manuscript now reflects this point more explicitly and cautiously.

Changes in the manuscript: The revised content has been added to page 5 lines 12-17.

“At the global level, Selinexor treatment also did not significantly alter the overall proportions of major stromal and immune cell populations, including fibroblasts, T cells, macrophages, B cells, and endothelial cells (Fig. 1j and Supplementary Fig. 1n). Re-clustering of T cells identified five distinct subsets, with a modest increase in the relative abundance of cytotoxic T cells following Selinexor treatment (Supplementary Fig. 2a-e). However, given the absence of broad changes in major T-cell subsets, we do not interpret this finding alone as evidence of T-cell functional reinvigoration.”

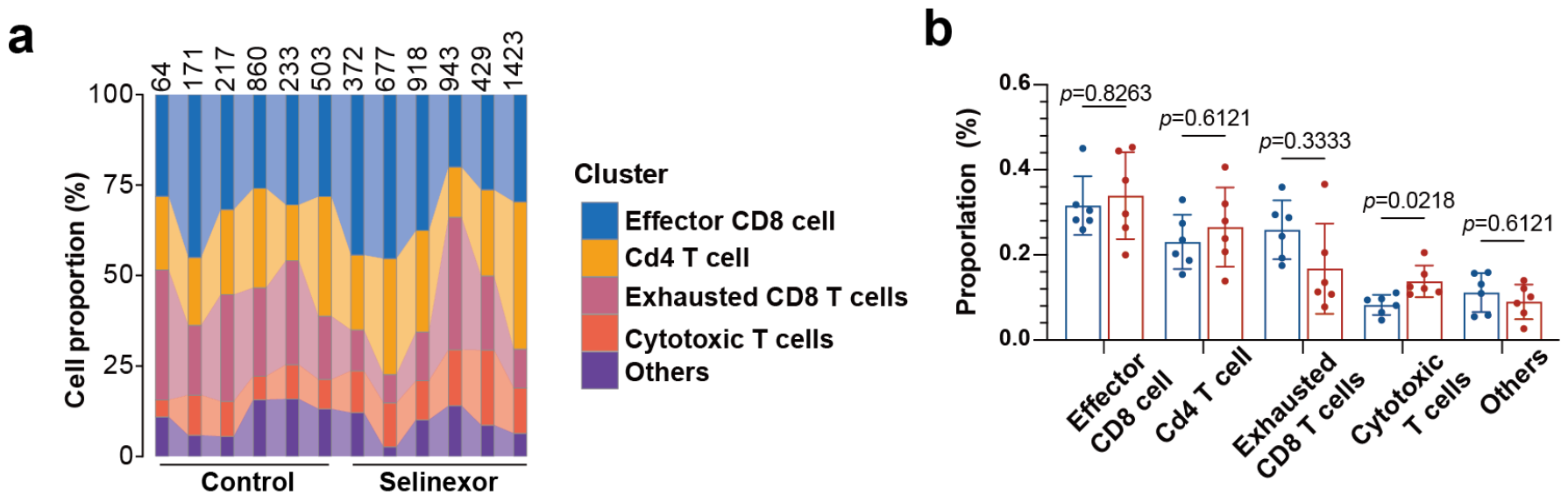

Fig.R12. a Proportions of macrophage clusters in control and Selinexor-treated tumours. Numbers above the plot indicate the total number of macrophages detected in each tumour. **b** Statistical comparison of the macrophage cluster proportions shown in **a**. Data in **a** were analysed using multiple Mann–Whitney tests with false discovery rate correction. Data are from n = 6 mice per group. Data are presented as mean ± s.d. Source data are provided as a Source Data file.

b. The scRNA data show TREM2^{+} macrophages are transcriptionally distinct and enriched for antigen-presentation genes, yet the manuscript claims they are immunosuppressive and drive immune exclusion. The discrepancy in "antigen presentation" gene set narrative should be clarified.

Response: We thank the Reviewer for this important point and agree that the original text did not sufficiently distinguish between antigen-processing/presentation-related transcriptional features and the functional immunological consequence of those features. We have now clarified this point substantially in the revised manuscript.

Specifically, the TREM2^{+} macrophage cluster was enriched for genes involved in antigen processing and presentation, including Ctss, Cstb, and Lgmn. However, this same population also co-expressed multiple genes associated with immunoregulatory and lipid-associated macrophage states, including Gas6[2], Maf[3], Apoe, Abcg1, Gpnmb, and Psap[4], as well as the inhibitory Fc receptor Fcgr2b[5] (Fig. R11d-g). We therefore do not interpret this cluster as a conventional pro-inflammatory antigen-presenting population. Rather, we interpret it as a macrophage state that combines antigen-processing capacity with inhibitory and immunoregulatory features, which is more consistent with a tolerogenic mode of antigen presentation than with productive anti-tumour immune activation.

This distinction is important to our interpretation. In the revised manuscript, we no longer imply that antigen-presentation signatures alone establish immune exclusion. Instead, we describe the TREM2^{+} population more precisely as a lipid-associated, immunoregulatory macrophage state enriched for antigen-processing and

inhibitory programs. This framing better reconciles the transcriptional data with the functional validation added in the revision.

Changes in the manuscript: The revised content has been added to page 5 lines 17-21.

"By contrast, macrophage re-analysis revealed a more prominent and selective reduction in cluster 3 following XPO1 inhibition (Fig. 1k–m and Supplementary Fig. 2f). This population co-expressed Mrc1 (CD206) and Trem2, together with multiple genes previously associated with immunoregulatory macrophage states, including Gas6[24] and Maf[25], as well as genes involved in lipid metabolism and antigen processing and presentation (Fig. 1n and Supplementary Fig. 2g-j)."

c.For example, how would the subset of TAMs behave in T-cell suppressive assays / what would be their functional activity (cytokine secretion /protein markers) ex-vivo/in-vitro?

Response: We thank the Reviewer for this constructive suggestion. As described above in our response to Comment 1, we have now directly evaluated the functional properties of purified TREM2^{+} and TREM2^{-} macrophages isolated from the co-culture system. These experiments showed that TREM2^{+} macrophages secrete higher levels of IL-10 and TGF-β and suppress the proportion of IFN-γ^{+} CD8^{+} T cells relative to TREM2^{-} macrophages (Fig. R11a-c), supporting the interpretation that this subset has immunoregulatory activity in vitro.

We have incorporated these data into the revised manuscript and now use them as functional support for the designation of TREM2^{+} macrophages as an immunoregulatory subset. At the same time, we have kept the interpretation appropriately cautious and do not overextend these in vitro results to claim definitive in vivo causality.

Changes in the manuscript: The revised content has been added to page 6 lines 27-31, and page 7 lines 1-3.

"To further assess the immunoregulatory role of TREM2^{+} macrophages, we sorted TREM2^{+} and TREM2^{-} macrophages from co-culture systems. enzyme-linked immunosorbent assay (ELISA) and reverse transcription quantitative polymerase chain reaction (RT-qPCR) analyses of conditioned media revealed that TREM2^{+} macrophages secreted higher levels of IL-10 and TGF-β compared to TREM2^{-} macrophages (Supplementary Fig. 4g,h). Additionally, in co-culture assays with CD8^{+} T cells isolated from PBMCs, TREM2^{+} macrophages reduced the proportion of IFN-γ^{+} CD8^{+} T cells relative to TREM2^{-} macrophages (Supplementary Fig. 4i). These findings

collectively support the notion that TREM2$^+$ macrophages represent an immunoregulatory subset in the SCLC microenvironment."

d. Reproducibility - Reviewing Figure 1K or Extended Data Figure 2a, one would assume that it is a sample size of 3 animals per group. The effect size seems to be low to convincingly demonstrate a differential effect on M2c variation (1K) or direct anti-tumor effect (1e, 1f) without having an understanding about the variability and sample size tested. On the other hand, n=6 per group (should be clearly marked in text or figure legends) as shown in Figure 2e and 2f seems convincing. Strongly recommend that the authors explicitly state the number of biological /technical replicates and number of animals used per group in all experiments. If possible and only where feasible, data in Figure 2 could be repeated with additional animals.

Response: We thank the reviewer for this important comment and fully agree that clear reporting of sample size, biological replicates, and animal numbers is essential for evaluating reproducibility, variability, and effect size.

To address this concern, we have revised the manuscript throughout to explicitly report the exact number of biological replicates and the number of animals used per group in the main text, figure legends, and Methods. In particular, for the in vivo experiments associated with Fig. 1 and the corresponding scRNA-seq analysis, we expanded the cohort by adding three additional animals per group, resulting in a total of n = 6 mice per group. In the expanded cohort, Selinexor still significantly reduced tumour volume and tumour weight compared with vehicle-treated controls (Fig. R13a-c), and the key single-cell findings, including the selective reduction of the Cluster 3 (TREM2$^+$) immunoregulatory macrophage population, remained evident after batch correction and integrated analysis (Fig. R13d-l).

For the Fig. 2 orthotopic experiments, these were already performed at n=6 per group as noted in the original submission (now more clearly indicated in the figure legends). More broadly, we have standardized replicate reporting throughout the revised manuscript such that animal studies are explicitly annotated with the number of mice per group and in vitro studies are annotated with the number of biologically independent experiments wherever applicable.

Changes in the manuscript: We have revised the figure legends and Methods to explicitly indicate the number of biologically independent experiments and the number of mice per group for each assay. In addition, the in vivo

experiments and scRNA-seq analyses associated with Fig. 1 were expanded to n = 6 mice per group, and the revised Results and figure legends now reflect these updated sample sizes.

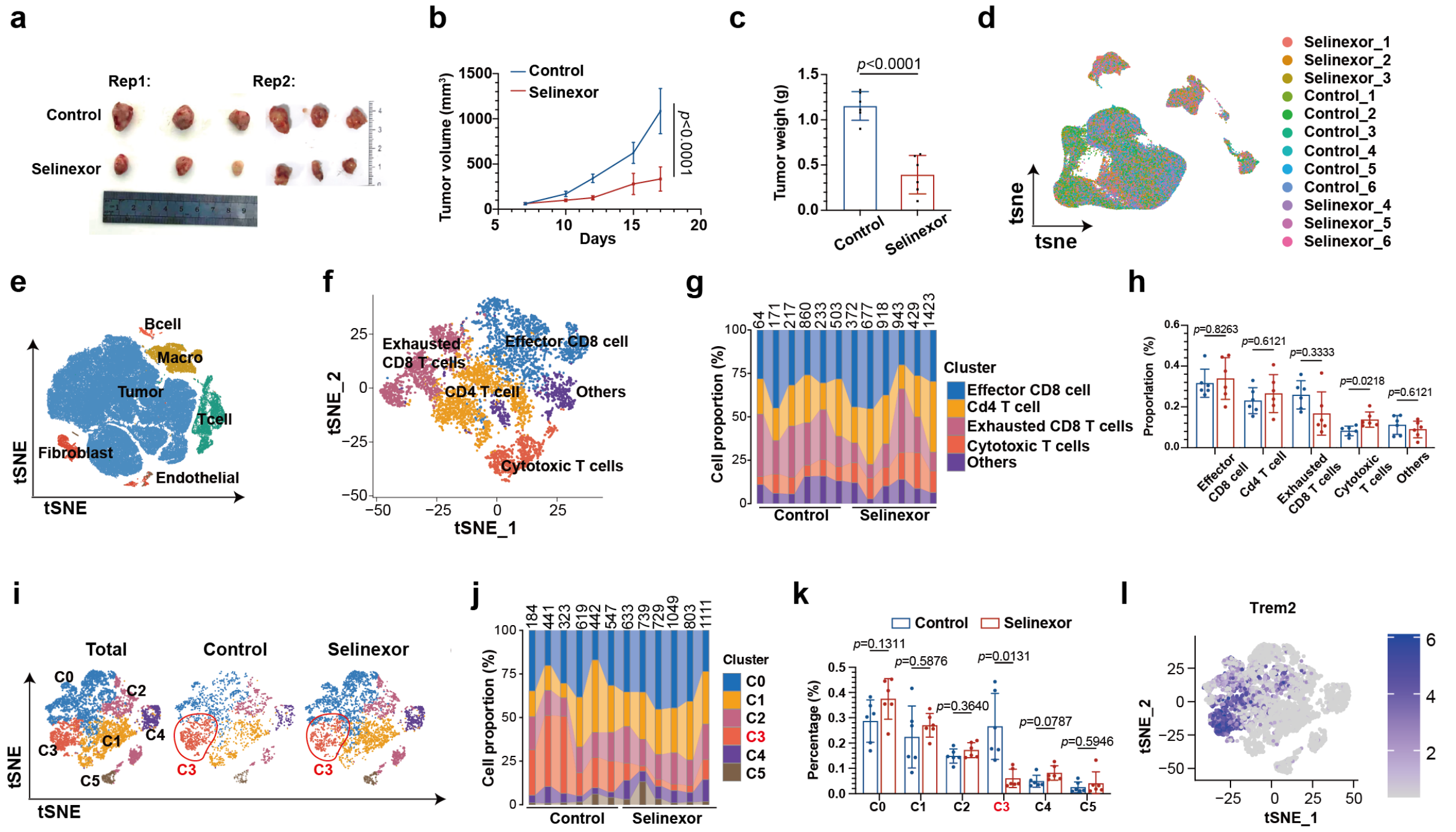

Fig.R13. a RP116 murine SCLC cells (1 × 10^6) were injected subcutaneously into C57BL/6 mice. Mice were randomized into two groups and treated with Selinexor (5 mg/kg, p.o., once weekly) or vehicle control. Representative gross images of SCLC tumours collected at the experimental endpoint. **b,c** Tumour growth curves (**b**) and tumour weights at endpoint (**c**) in control and Selinexor-treated mice. **d** Distribution of cells from 12 tumour samples after batch-effect correction using the Harmony algorithm, colour-coded by tumour sample. **e** t-SNE projection of 105,555 single cells from 12 tumours showing six major cell populations: tumour cells, B cells, T cells, macrophages, fibroblasts, and endothelial cells. Each dot represents one cell and is coloured by annotated cell type. **f** t-SNE projection of T cells from all tumours, identifying five major T-cell clusters. **g** Proportions of T cell clusters in control and Selinexor-treated tumours. Numbers above the plot indicate the total number of T cells detected in each tumour. **h** Statistical comparison of the T cell cluster proportions shown in **g. i** t-SNE projection of macrophages from all tumours (left), control tumours (middle), and Selinexor-treated tumours (right). Cluster 3 macrophages are outlined in red. **j** Proportions of macrophage clusters in control and Selinexor-treated tumours. Numbers above the plot indicate the total number of macrophages detected in each tumour. **k** Statistical comparison of the macrophage cluster proportions shown in **g. l** Feature plots showing the expression of Trem2 across macrophage clusters. Data in **b** were analysed by two-way repeated-measures ANOVA followed by Sidak's multiple-comparisons test. Data in **c** were compared using a two-sided unpaired t-tests. Data

in **h,k** were analysed using multiple Mann–Whitney tests with false discovery rate correction. Data are from n = 6 mice per group. Data are presented as mean ± s.d. Source data are provided as a Source Data file.

Comment 3: Tumor TNFSF15 levels alter macrophage state in co-culture (Figure 2). Given the emerging interest in TL1A/DR3 biology, it is critical to understand the expression of the receptors for TNFSF15 (e.g., DR3/TNFRSF25, DcR3) on the different subsets of TAMs vs tumor cells themselves.

a. To confirm the mechanism and rule out any indirect effect of other secreted factors, it would be great to evaluate and present the data on the expression of TNFSF15 receptors on the macrophages used in the assay system (THP-1 derived as well as primary TAMs) by FACS.

Response: We sincerely thank the reviewer for this important suggestion. We agree that defining the expression of TNFSF15 receptors in the relevant cellular compartments is critical for clarifying the mechanism and for determining whether the observed effects are mediated directly through macrophages rather than indirectly through tumour cells.

To address this point, we examined endogenous DR3 expression in THP-1-derived macrophages, PBMC-derived macrophages, and the SCLC cell lines used in this study. DR3 expression was substantially higher in macrophages than in tumour cells, suggesting that macrophages are likely the more relevant TNFSF15-responsive population in our experimental system (Fig. R14a).

In addition, we evaluated surface expression of the canonical TNFSF15 signaling receptor DR3 (TNFRSF25) by flow cytometry in the macrophage populations used in our assay system, including both THP-1-derived macrophages and PBMC-derived macrophages. We further stratified these macrophages according to TREM2 expression status. In both systems, DR3 was readily detectable on macrophages, and $TREM2^{+}$ macrophages exhibited a significantly lower frequency of DR3-positive cells than $TREM2^{-}$ macrophages (Fig. R14b,c). Together, these data support a macrophage-directed mechanism in our co-culture model, while also indicating potential heterogeneity in receptor availability among macrophage subsets.

In the current revision, we focused on DR3/TNFRSF25 because it is the canonical signaling receptor for TNFSF15 and most directly addresses receptor availability in the macrophage assay system. We agree, however,

that evaluation of DcR3 would provide additional insight, particularly with respect to ligand sequestration or buffering mechanisms in the tumour microenvironment. Although DcR3 was not examined in the present study, we now acknowledge this as an important limitation and future direction.

Changes in the manuscript: The revised content has been added to page 8 lines 27-31 and page 9 lines 1-5. "Given the emerging interest in TL1A/TNFSF15 receptor biology, we further assessed expression of the canonical TNFSF15 receptor DR3 (TNFRSF25) in our assay system. Flow cytometry analysis showed that DR3 was detectable in both THP-1-derived and PBMC-derived macrophages, whereas its expression was substantially lower in normal SCLC cell lines (Supplementary Fig. 7e). Notably, within the macrophage compartment, TREM2^{+} macrophages exhibited a lower frequency of DR3-positive cells than TREM2^{-} macrophages (Supplementary Fig. 7f and g). These data suggest that TNFSF15 responsiveness may differ across macrophage states and support macrophages as the more likely TNFSF15-responsive population in this co-culture system. While these results support the direct activation of NF-κB and STAT pathways by TNFSF15 in macrophages, further studies are needed to fully characterize receptor utilization and assess the relative contributions of TNFSF15 versus other co-culture-derived signals in vivo."

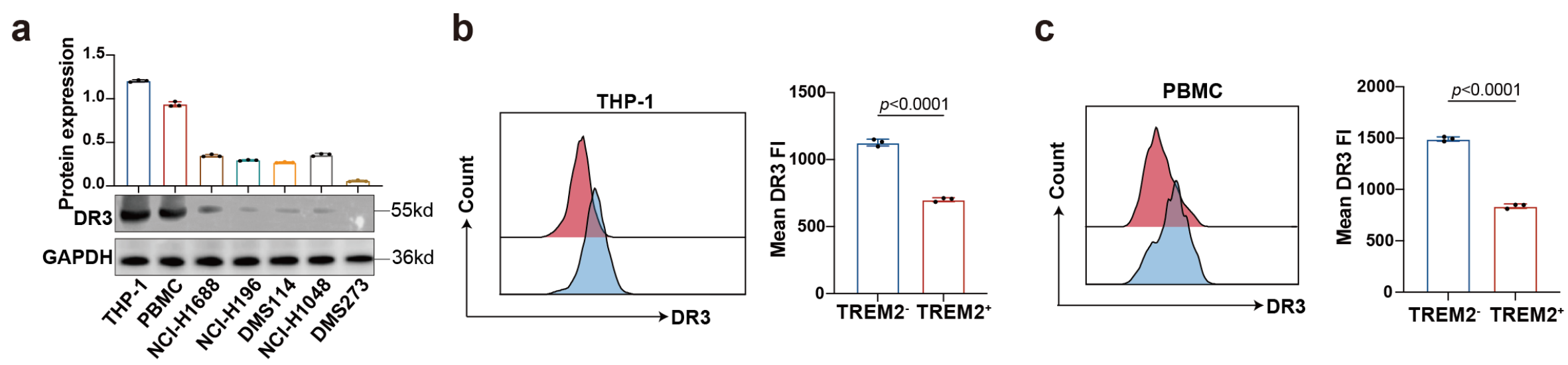

Fig. R14. **a** Immunoblot analysis of endogenous DR3 expression across THP-1-derived macrophages, PBMC-derived macrophages, and SCLC cell lines. **b,c** Flow cytometric analysis of DR3 expression in TREM2^{-} and TREM2^{+} macrophages derived from THP-1 cells (**b**) or PBMCs (**c**) after co-culture with the indicated SCLC cells. Representative flow cytometry plots are shown on the left, and quantification of the geometric mean fluorescence intensity (gMFI) is shown on the right. Data in **b,c** were analysed using two-sided unpaired t-tests. Data are from n = 3 biologically independent experiments and are presented as mean ± s.d. Source data are provided as a Source Data file.

b, Does recombinant TNFSF15 activate downstream signaling in macrophages?

Response: We thank the Reviewer for this valuable suggestion. To directly determine whether recombinant

TNFSF15 is sufficient to activate downstream signaling in macrophages, we treated THP-1-derived M0 macrophages with recombinant TNFSF15 (1μg/ml, 24 h) and examined both transcriptional and biochemical responses.

Bulk RNA-seq analysis showed that TNFSF15 stimulation induced broad inflammatory and immune-related transcriptional changes in macrophages (Fig. R15a). KEGG pathway enrichment analysis of upregulated genes revealed significant enrichment of the TNF signaling, NF-κB signaling, and JAK–STAT signaling pathways (Fig. R15b), indicating activation of downstream inflammatory signaling programs. To validate these pathway-level findings biochemically, we performed immunoblot analysis and observed increased phosphorylation of p65 and STAT1 following TNFSF15 treatment (Fig. R15c). Together, these data demonstrate that recombinant TNFSF15 is sufficient to directly activate NF-κB- and STAT-associated signaling in macrophages.

In addition, we examined macrophage signaling responses in tumour–macrophage co-culture systems under conditions of XPO1 perturbation in tumour cells. Macrophages co-cultured with XPO1-knockdown tumour cells showed increased p-STAT1 and p-p65, whereas macrophages co-cultured with XPO1-overexpressing tumour cells showed reduced phosphorylation of these signaling proteins (Fig. R15d). These co-culture data are correlative and reflect the combined effects of tumour-cell secretome changes upon XPO1 modulation, with TNFSF15 likely representing an important, but not necessarily exclusive, contributor.

Changes in the manuscript: The revised content has been added to page 8 lines 13-25.

"To determine whether TNFSF15 directly activates downstream signaling in macrophages, we treated THP-1-derived M0 macrophages with recombinant TNFSF15 (1μg/ml, 24 h) and performed bulk RNA-seq. Differential expression and KEGG pathway enrichment analyses revealed significant upregulation of genes involved in the TNF, NF-κB, and JAK-STAT signaling pathways (Supplementary Fig. 7a,b), indicating that TNFSF15 induces a broad inflammatory transcriptional response in macrophages. Consistent with these transcriptomic changes, immunoblot analysis showed increased phosphorylation of p65 (Ser536) and STAT1 (Ser701) following TNFSF15 stimulation (Supplementary Fig. 7c), demonstrating that recombinant TNFSF15 is sufficient to directly activate downstream inflammatory signaling in macrophages. In parallel, in tumour-macrophage co-culture experiments, macrophages co-cultured with XPO1-knockdown NCI-H1688 cells exhibited increased p-STAT1 and p-p65, whereas those co-cultured with XPO1-overexpressing NCI-H1048 cells showed reduced phosphorylation of these signaling proteins (Supplementary Fig. 7d). These co-culture data are

correlative and reflect the combined effects of tumour-cell secretome changes upon XPO1 modulation, with TNFSF15 likely representing an important, but not necessarily exclusive, contributor.”

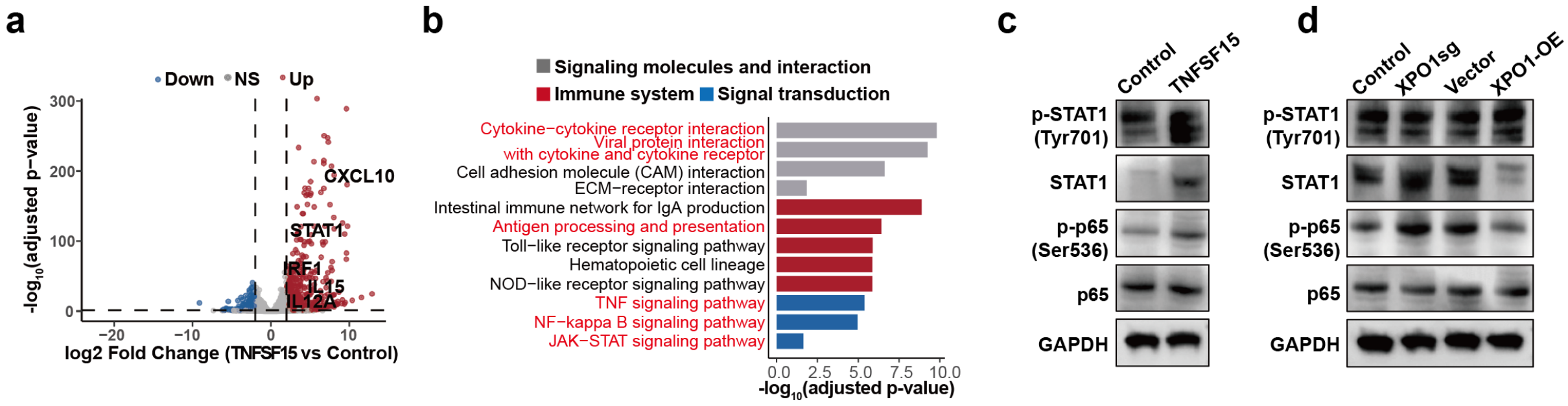

Fig. R15. TNFSF15-induced transcriptional changes and downstream STAT1/NF-κB signaling in macrophages. a Differential gene expression analysis between control and recombinant TNFSF15-treated THP-1-derived macrophages (1μg/ml, 24 h). Differential expression analysis was performed using edgeR, with |log2(fold change)| > 2 and adjusted P < 0.05 as the thresholds. **b** KEGG pathway enrichment analysis of the 1,780 upregulated genes shown in panel **a**. Pathways are ranked by −log10(adjusted p value). **c** Immunoblot analysis of total STAT1, phospho-STAT1 (Tyr701), total NF-κB p65, and phospho-NF-κB p65 (Ser536) in THP-1-derived M0 macrophages treated with recombinant TNFSF15 (1μg/ml, 24 h) or vehicle control. **d** Immunoblot analysis of total STAT1, phospho-STAT1 (Tyr701), total NF-κB p65, and phospho-NF-κB p65 (Ser536) in THP-1-derived M0 macrophages co-cultured with XPO1-deficient or XPO1-overexpressing SCLC cells. Data are from n = 3 biologically independent experiments. Source data are provided as a Source Data file.

c, XPO1 knockdown in SCLC cells results in upregulation of not just TNFSF15 but also CXCL1, CCL22, IL1B, and TNF (Figure 3b and Extended Data Fig. 7b) along with several other pathways. The authors should discuss why they focused only on TNFSF15 and not other factors. If they followed up on any other genes /pathways with no association found, those should be outlined or mentioned.

Response: We sincerely thank the Reviewer for this thoughtful comment. We agree that XPO1 knockdown in SCLC cells induced multiple immune-related factors, including TNFSF15, CXCL1, CCL22, IL1B, and TNF (Figure 3b and Extended Data Fig. 7b), and that it is important to clarify why we prioritized TNFSF15 for further study.

Among these candidates, TNFSF15 showed the most consistent and robust upregulation across the SCLC cell

lines analyzed, and it also emerged prominently in pathway enrichment analyses related to TNF-associated inflammatory signaling. In addition, TNFSF15 was particularly compelling because of its known relevance to immune regulation and myeloid cell biology[8,9], which aligned closely with the macrophage-centered phenotypes observed in our study. For these reasons, we selected TNFSF15 as the primary candidate for mechanistic follow-up.

At the same time, we agree with the reviewer that other upregulated factors, including CXCL1, CCL22, IL1B, and TNF, may also contribute to the immune remodeling induced by XPO1 inhibition. However, their individual or cooperative roles were not systematically dissected in the current study. We now explicitly acknowledge this point in the revised Discussion as a limitation of the present work and as an important direction for future investigation.

To address the reviewer's concern, we have revised the manuscript to clarify the rationale for focusing on TNFSF15 and to note that additional XPO1-regulated cytokines and inflammatory pathways may also participate in shaping the SCLC immune microenvironment.

Changes in the manuscript: The revised content has been added to page 7 lines 29-31, page 8 line 1 and page 17 lines 18-20 .

"Differential expression and pathway enrichment analyses revealed significant changes in cytokine–cytokine receptor interaction and TNF signaling pathways (Fig. 3a). Notably, TNFSF15 was the most prominently upregulated gene in these pathways, suggesting a potential role in immune regulation and macrophage differentiation (Fig. 3b)[28,29]."

"While our data nominate TNFSF15 as a key downstream effector of XPO1 inhibition, we cannot exclude additional contributions from other induced cytokines, such as CXCL1, CCL22, and IL1B, which remain to be explored in future studies."

Comment 4: Primarily the authors had access to or created various cell lines to test. XPO1 knockdown in lines with high endogenous expression (NCI-H1688, NCI-H196, and DMS114) or overexpression of XPO1 in cell lines with low endogenous protein expression (NCI-H1048 and DMS273). They use NCI-H1688 and NCI-1048 primarily in most studies as prototypical examples. However, they also use additional cell lines in some experiments but not all (e.g., NCI-H196 identify TNFSF15 as a DEG). Given that these cell lines are

representative SCLC subtypes under different transcriptional control, it would be ideal to consistently evaluate all different cell lines or provide justification for the selective use.

Response: We thank the reviewer for this important comment regarding the consistency of cell line usage across the study. We agree that systematic evaluation across all available SCLC models would be ideal, particularly given the marked molecular heterogeneity of SCLC subtypes.

In the current study, we adopted a strategy using representative core models complemented by additional validation models. Specifically, we selected NCI-H1688 and NCI-H1048 as the primary models because they represent distinct SCLC subtype contexts and opposite endogenous XPO1 expression states: NCI-H1688 is an XPO1-high SCLC-A model, whereas NCI-H1048 is an XPO1-low SCLC-P model. This pairing enabled complementary loss-of-function and gain-of-function approaches and provided a practical framework to interrogate XPO1-dependent phenotypes across biologically distinct subtype settings.

To assess whether the observed effects were not restricted to these two lines, we extended selected key experiments to additional SCLC models, including NCI-H196, DMS114, and DMS273, which represent distinct transcriptional subtype contexts. For example, we performed XPO1 knockdown and transcriptomic profiling in both NCI-H1688 and NCI-H196, demonstrating that TNFSF15 induction upon XPO1 depletion was reproducible across independent XPO1-high SCLC models. This finding was further supported in XPO1-overexpressing NCI-H1048 cells by RT–qPCR and ELISA. Likewise, selected mechanistic observations were further evaluated in DMS114 and DMS273, supporting the broader relevance of our findings across multiple SCLC subtype contexts.

At the same time, we acknowledge that not every assay was performed in every cell line. Rather than applying the full experimental workflow uniformly across all models, we prioritized the most representative and complementary cell lines for in-depth mechanistic studies and used additional lines for orthogonal validation of key findings. We have now clarified this rationale in the revised manuscript and more explicitly distinguished experiments performed in the core discovery models from those included for cross-model validation. We also acknowledge this as a limitation of the current study and note that future work across a broader panel of subtype-defined SCLC models will be important to further establish the generalizability of XPO1-dependent immune regulatory mechanisms.

Changes in the manuscript: The revised content has been added to page 6 lines 5-11 and page 7 lines 28-29.

"To investigate how tumour-cell XPO1 regulates macrophage differentiation, we first examined XPO1 expression across a panel of nine SCLC cell lines representing major molecular subtypes. XPO1 was expressed across all subtypes, with the highest level in NCI-H1688 (SCLC-A) and the lowest in NCI-H1048 (SCLC-P) (Supplementary Fig. 4a). We therefore selected these two lines as complementary core models for loss-of-function and gain-of-function studies, respectively (Fig. 2a). To assess the consistency of the observed phenotype across additional subtype contexts, selected key mechanistic experiments were further extended to DMS273 (SCLC-N), DMS114 (SCLC-Y), and NCI-H196 (SCLC-Y) (Supplementary Fig. 4b)."

"To define the transcriptional consequences of XPO1 depletion, we performed bulk RNA-seq in two independent XPO1-high SCLC cell lines, NCI-H1688 and NCI-H196, following XPO1 knockdown."

a. In Extended Data Figure 6a – Include what A/N/P/I denote for the cell lines listed (SCLC subtypes: SCLC-A, SCLC-N, SCLC-P, and SCLC-I?). Normalize to GAPDH and plot XPO1 levels for relative quantitation (e.g., NCI-H1688 vs NCI-H1048)

Response: We sincerely thank the reviewer for this constructive suggestion. We agree that it is important to clearly indicate the SCLC subtype annotation for each cell line shown in Extended Data Figure 6a. In the revised figure, we have now explicitly defined A, N, P, and I as SCLC-A, SCLC-N, SCLC-P, and SCLC-I, respectively (Fig. R16a).

In addition, as suggested, we have normalized XPO1 protein expression to GAPDH and added relative quantification to facilitate comparison across the indicated cell lines (Fig. R16a). This revision makes the differences in endogenous XPO1 expression clearer, including the relatively high expression in NCI-H1688 and low expression in NCI-H1048. We believe these changes improve the clarity of the figure and provide a more informative overview of XPO1 expression across subtype-defined SCLC models.

Changes in the manuscript: We revised Extended Data Figure 6a and its legend to define A/N/P/I as SCLC-A/SCLC-N/SCLC-P/SCLC-I, and added GAPDH-normalized relative quantification of XPO1 protein levels across the indicated cell lines.

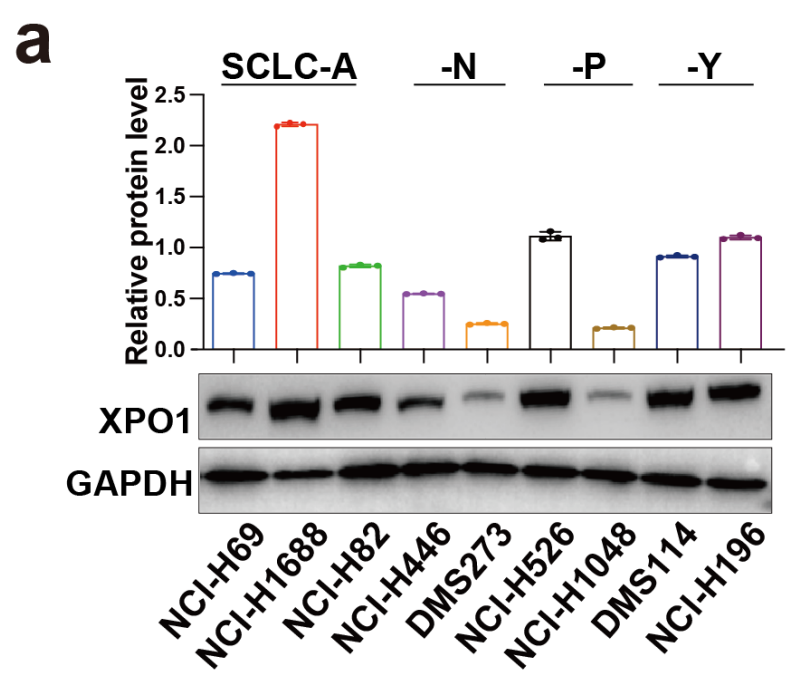

Fig. R16. a Endogenous XPO1 protein expression in SCLC cell lines, as determined by immunoblotting (bottom) with ImageJ-based quantification shown above (top). Data in are from n = 3 biologically independent experiments. Data are presented as mean ± standard deviation (s.d.). Source data are provided as a Source Data file.

Comment 5: Given the unimodal nature (single peak) of histograms, it is suggested that Fig 6 C, D, G should be represented as gMFI instead of % positive cells (also Extended Data Figure 6). Fluorescence intensity would be a more precise and informative metric.

Response: We sincerely thank the reviewer for this valuable suggestion. We agree that, for markers showing a largely unimodal fluorescence distribution, geometric mean fluorescence intensity (gMFI) provides a more informative and quantitative readout than percentage of positive cells, as it better captures shifts in signal intensity without relying on an arbitrary positivity threshold.

In response, we have revised Fig. 6c,d,g and Extended Data Fig. 6 to present the flow cytometry quantification as gMFI rather than % positive cells. The representative plots remain consistent with the original trends, and the revised quantification now provides a more accurate comparison of marker expression levels across the indicated conditions (Fig. R17a-e). We believe this change improves the precision and interpretability of these data.

Changes in the manuscript: Fig. 6c,d,g and Extended Data Fig. 6 have been revised to show gMFI values instead of % positive cells for the corresponding flow cytometry analyses.

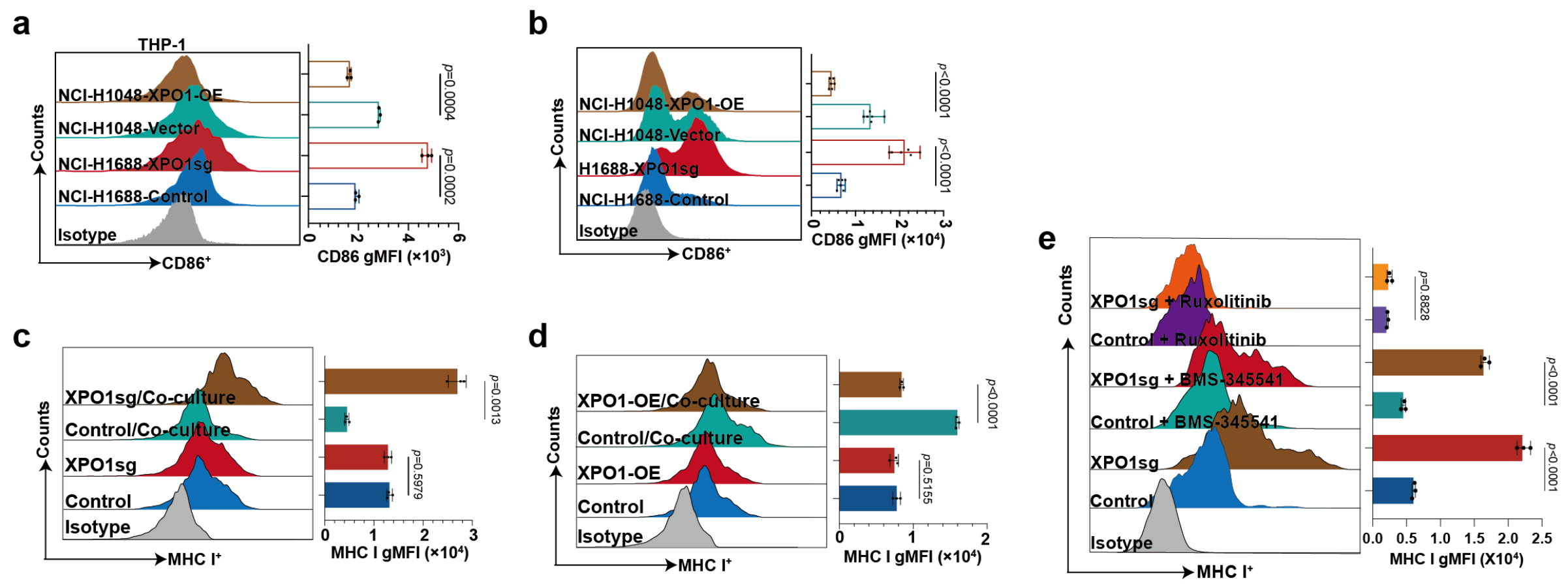

Fig. R17. a Flow cytometry analysis of CD86 cell surface expression on macrophages co-cultured with SCLC cells. Representative flow cytometry plots are shown on the left, and quantification of the geometric mean fluorescence intensity (gMFI) is shown on the right. **b** Flow cytometry analysis showing the proportion of $CD86^+$ macrophages within the $F4/80^+$ macrophage population in the indicated SCLC xenograft tumors. Representative flow cytometry plots are shown on the left, and quantification of the gMFI is shown on the right. **c,d** flow cytometry analyses of MHC I expression in the indicated SCLC cell lines cultured alone or co-cultured with THP-1-derived macrophages. Representative flow cytometry plots are shown on the left, and quantification of the gMFI is shown on the right. **e** Flow cytometr analyses of MHC I expression in control and NCI-H1688/XPO1-silenced cells co-cultured with THP-1-derived macrophages, with or without pretreatment with the JAK1/2 inhibitor ruxolitinib or the IKK inhibitor BMS-345541 for 24 h. Representative flow cytometry plots are shown on the left, and quantification of the gMFI is shown on the right. Data were analyzed using a two-tailed unpaired t-test. Data in panels **a,c,d,e** are from n = 3 biologically independent experiments. Data in **b** are from n = 6 mice per group. Data are presented as mean ±s.d.. Source data are provided as a Source Data file.

Comment 6: It is important for the authors to include detailed information about Selinexor -where was it purchased /procured from and how was it formulated and dosed. Please include all the relevant details for anyone else trying to reproduce the results or extend their findings. Did the authors test the pharmacokinetics of Selinexor (as a single agent or in combination) in their in-vivo models? If so, the details and data should be included.

Response: We thank the reviewer for this important comment and agree that detailed information regarding Selinexor procurement, formulation, dosing, and pharmacokinetic evaluation is essential for reproducibility.

In the revised manuscript, we have now provided the full experimental details for Selinexor treatment, including the vendor, catalogue number, route of administration, formulation, dose, and treatment schedule. Specifically, Selinexor was purchased from MedChemExpress (HY-17536) and administered by oral gavage at 5 mg/kg once weekly (QW). The exact vehicle composition and dosing schedule have now been added to the Methods section and the relevant figure legends.

The 5 mg/kg once-weekly oral dosing regimen was selected on the basis of tolerability and antitumour activity in our pilot experiments[10,11], together with reference to prior published in vivo studies using Selinexor in SCLC models[12,13]. In our initial testing, a higher oral dose (10 mg/kg, once weekly) was less well tolerated in some mice, as indicated by body weight loss. We therefore adopted the lower 5 mg/kg oral regimen for the current study, which maintained antitumour activity and allowed evaluation of immune effects with acceptable tolerability (Supplementary Fig. 12m,n).

We did not perform dedicated pharmacokinetic analyses of Selinexor, either as a single agent or in combination, in the in vivo models used in this study. We agree that such data would be valuable for further mechanistic and translational interpretation.

Changes in the manuscript: The revised content has been added to page 20 lines 7-10 and corresponding figure legends.

"For treatment, Selinexor (MedChemExpress, HY-17536) was formulated in corn oil and administered by oral gavage at 5 mg/kg once weekly (QW). Anti-PD-1 antibody (Bio X Cell, BE0146) was administered intraperitoneally at 200 μg/injection every 3 days. Treatment was initiated on day 7 after implantation and continued until the experimental endpoint."

Comment 7: The authors use ubiquitin mutants (K48R/K63R) and functional assays to infer that TRIM21 catalyzes the K48-linked ubiquitination of IRF3 at lysine 313. Additional details of the constructs used in these experiments would be helpful.

Response: We sincerely thank the reviewer for these thoughtful and constructive comments. We agree that additional experimental detail and stronger orthogonal evidence are important for supporting the conclusion that TRIM21 catalyzes K48-linked polyubiquitination of IRF3 at lysine 313. In response, we have clarified the

construct and assay details, repeated the key ubiquitination experiments, and added orthogonal evidence from mass spectrometry and linkage-specific ubiquitination analyses.

Changes in the manuscript: The revised content has been added to page 19 lines 18-22.

"For cell-based ubiquitylation assays, FLAG-tagged wild-type ubiquitin (FLAG-Ub), FLAG-Ub-K48R, and FLAG-Ub-K63R expression constructs were used. The K48R and K63R constructs are single lysine-to-arginine ubiquitin mutants generated by site-directed mutagenesis. HA-tagged full-length TRIM21 and MYC-tagged full-length IRF3, as well as the indicated IRF3 and TRIM21 mutant constructs, were used as specified in each experiment. All constructs were verified by Sanger sequencing before use."

a. It becomes critical to list the technical replicate experiments conducted to confirm reduction in ubiquitination with K313R mutant. The blot data presented in Figure 5J (and original blot images attached in the Supplemental data package) is smeared at this particular lane and makes it difficult to interpret.

Response: We agree that the original blot shown in Fig. 5j was not sufficiently clear, particularly in the K313R lane, and that this limited interpretability. To address this, we repeated the ubiquitination assay and replaced Fig. 5j with a clearer representative result from repeated experiments. Across three replicate experiments, the K313R mutant consistently showed reduced TRIM21-mediated ubiquitination relative to wild-type IRF3 (Fig. R18a). We have now clarified the number of biologically independent experiments in the figure legend and included the updated source data/original blot images in the revised submission.

b. In fact, it would make the data more definitive if the authors had done mass-spectrometry studies to evaluate ubiquitinated lysine (di-Gly peptide).

Response: We agree that mass spectrometry-based identification of ubiquitinated lysine residues provides much stronger evidence. To strengthen this point, we performed mass spectrometry analysis and identified a ubiquitinated peptide consistent with modification at IRF3 K313. This result nominated K313 as a major candidate ubiquitination site. We then generated the K313R mutant for functional validation and found that mutation of K313 markedly reduced TRIM21-mediated ubiquitination of IRF3. Together, the mass spectrometry and mutagenesis data support K313 as a major TRIM21-dependent ubiquitination site on IRF3 (Fig. R18b).

c. The use of linkage-specific reagents (e.g., K48 specific Ub antibody) or additional constructs (replacing other lysines except for K48) would be helpful to solidify this data.

Response: We also agree that additional evidence regarding ubiquitin linkage specificity is important. In addition to the ubiquitin mutant experiments using FLAG-Ub-K48R and FLAG-Ub-K63R, we performed immunoblot analysis with a K48-linkage-specific ubiquitin antibody. These experiments showed that XPO1-mediated ubiquitination of IRF3 was strongly dependent on K48 linkage (Fig. R18c). In parallel, K48-linked ubiquitination of IRF3 was reduced in XPO1-knockdown cells and increased in XPO1-overexpressing cells, consistent with the proposed XPO1–TRIM21–IRF3 axis. Together, these data further support the conclusion that TRIM21 promotes K48-linked polyubiquitination of IRF3.

d. Include additional details including the enzyme used in the in vitro ubiquitination assay. The E2 enzyme identity often directs linkage type and is important for reproducibility.

Response: We appreciate the reviewer's request for additional technical detail regarding the in vitro ubiquitination assay. We have now specified the key assay components in the revised Methods and figure legends. Briefly, the in vitro ubiquitination reactions were performed using recombinant TRIM21 and MYC-IRF3 in the presence of E1 (UBE1), E2 (UbcH5c), and FLAG-tagged ubiquitin. We agree that the identity of the E2 enzyme is important for reproducibility, and this information is now explicitly provided in the revised manuscript.

Taken together, these revisions substantially strengthen the evidence supporting TRIM21-mediated K48-linked polyubiquitination of IRF3, with K313 representing a major ubiquitination site in our system.

Changes in the manuscript: We replaced Fig. 5j with a clearer repeated experiment and clarified the number of biologically independent replicates in the corresponding figure legend. We also added mass spectrometry data identifying a ubiquitinated peptide consistent with modification at IRF3 K313, together with revised text describing K313 as a major TRIM21-dependent ubiquitination site. In addition, we included K48-linkage-specific ubiquitination data and expanded the Methods/figure legends to specify the constructs and in vitro ubiquitination assay components, including E1 (UBE1), E2 (UbcH5c), and FLAG-tagged ubiquitin.

The revised content has been added to page 11 lines 25-31:

"Consistently, K48-linked ubiquitination of IRF3 was significantly reduced in XPO1-knockdown cells, whereas XPO1 overexpression increased K48-linked ubiquitination of IRF3 (Fig. 5i), further supporting the importance of K48-linked polyubiquitination in this context. To identify the TRIM21-dependent ubiquitination site(s) on IRF3, we performed mass spectrometry and detected a ubiquitinated peptide consistent with modification at K313 (Supplementary Fig. 10h). Subsequent ubiquitination assays showed that mutation of K313 to arginine markedly impaired TRIM21-mediated IRF3 ubiquitination (Fig. 5j), supporting K313 as a major ubiquitination site in our system."

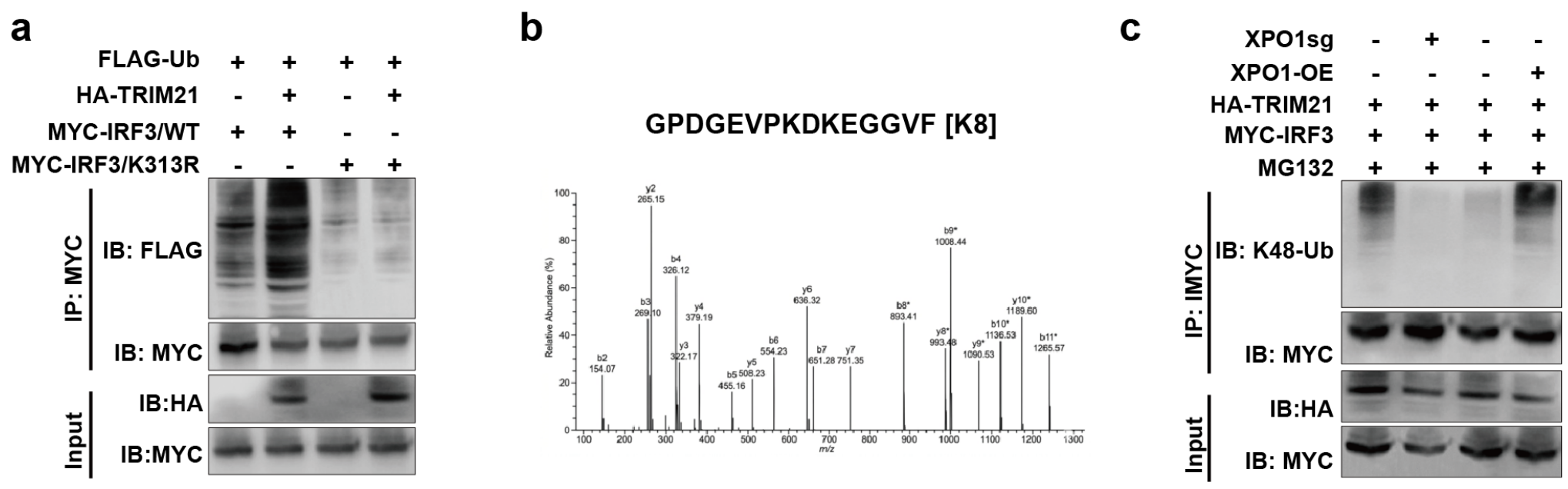

Fig. R18. a K313R mutation of IRF3 impaired TRIM21-mediated polyubiquitylation. Endogenous TRIM21 was depleted in HEK293T cells, followed by transfection with vector or HA-TRIM21 together with MYC-IRF3 or the indicated MYC-IRF3 mutants. Cell lysates were subjected to immunoprecipitation with an anti-MYC antibody, and polyubiquitylated IRF3 was detected by immunoblotting with an anti-FLAG antibody. **b** Representative tandem mass spectrum of the IRF3 peptide GPDGEVPKDKKEGGVF containing a diglycine-modified lysine, indicating ubiquitination at IRF3 K313 (the eighth residue lysine within the identified peptide). The assigned b and y fragment ions support confident localization of the modified site. **c** Following MG132 treatment, cells were subjected to immunoprecipitation with an anti-MYC antibody, and K48-linked polyubiquitylated IRF3 was detected by immunoblotting with an anti-K48-linkage-specific ubiquitin antibody. Data are representative of n = 3 biologically independent experiments. Source data are provided as a Source Data file.

Comment 8: Selinexor causes TRIM21 nuclear accumulation and XPO1 overexpression increases cytoplasmic TRIM21. Have the authors evaluated whether TRIM21 contains a canonical NES or if TRIM21 is exported as a complex? Use of other canonical nuclear export inhibitor (other than Selinexor) would have been helpful to clarify this point.

Response: We thank the Reviewer for this insightful comment. To further investigate the mechanism underlying TRIM21 nuclear export, we analysed the TRIM21 amino acid sequence and identified a leucine-rich hydrophobic

motif (ALELLQEVIIVL) consistent with a putative nuclear export sequence (NES) (Fig. R19a). In addition, GST pull-down assays showed that GST-tagged XPO1 directly interacts with HA-tagged TRIM21 (Fig. R19b), supporting the possibility that TRIM21 may associate directly with XPO1.

We agree that the use of an additional canonical nuclear export inhibitor, beyond Selinexor, would provide valuable orthogonal support for a general XPO1-dependent export mechanism. We also agree that our current data do not definitively establish whether TRIM21 is exported as a canonical direct cargo through its predicted NES-like motif, or whether adaptor proteins and/or additional cofactors facilitate this process in cells.

Importantly, in addition to Selinexor treatment, we observed concordant changes in TRIM21 subcellular localization following both XPO1 knockdown and XPO1 overexpression. These genetic perturbation data support the conclusion that TRIM21 nuclear－cytoplasmic distribution is regulated by XPO1. However, we have revised the manuscript to clarify that such evidence does not by itself distinguish between direct cargo recognition and indirect regulation through XPO1-associated export complexes. Accordingly, we now state more precisely that our data are consistent with TRIM21 being a direct XPO1 cargo, while not excluding the possibility that adaptor proteins participate in facilitating this interaction in cells.

We further agree that functional validation of the predicted NES-like motif by mutagenesis represents an important next step. We now explicitly note in the revised manuscript that future studies using NES mutagenesis, together with comparison to additional canonical nuclear export inhibitors, will be required to define the export mechanism more rigorously.

Changes in the manuscript: The revised content has been added to page 10 lines 18-21 and page 16 lines 23-30.

"Sequence analysis identified a putative leucine-rich NES-like motif (ALELLQEVIIVL) in TRIM21 (Supplementary Fig. 10b), and GST pull-down assays demonstrated direct interaction between XPO1 and TRIM21, supporting a model in which TRIM21 may serve as a direct XPO1 cargo (Fig. 4e)."

"Given that TRIM21 interacts with ubiquitin-conjugating enzymes in both the nucleus and cytoplasm compartments[52-54], its subcellular localization may be an important determinant of functional specificity. In this regard, our data support XPO1-dependent regulation of TRIM21 nuclear-cytoplasmic localization. However, we did not functionally validate the predicted NES-like motif in TRIM21 by mutagenesis, nor did we compare

Selinexor with additional canonical nuclear export inhibitors. Thus, while our findings are consistent with TRIM21 being a direct XPO1 cargo, they do not exclude the possibility that adaptor proteins or other cofactors facilitate this process in cells. Future studies combining NES mutagenesis with orthogonal inhibition of nuclear export will be required to define the export mechanism more rigorously.”

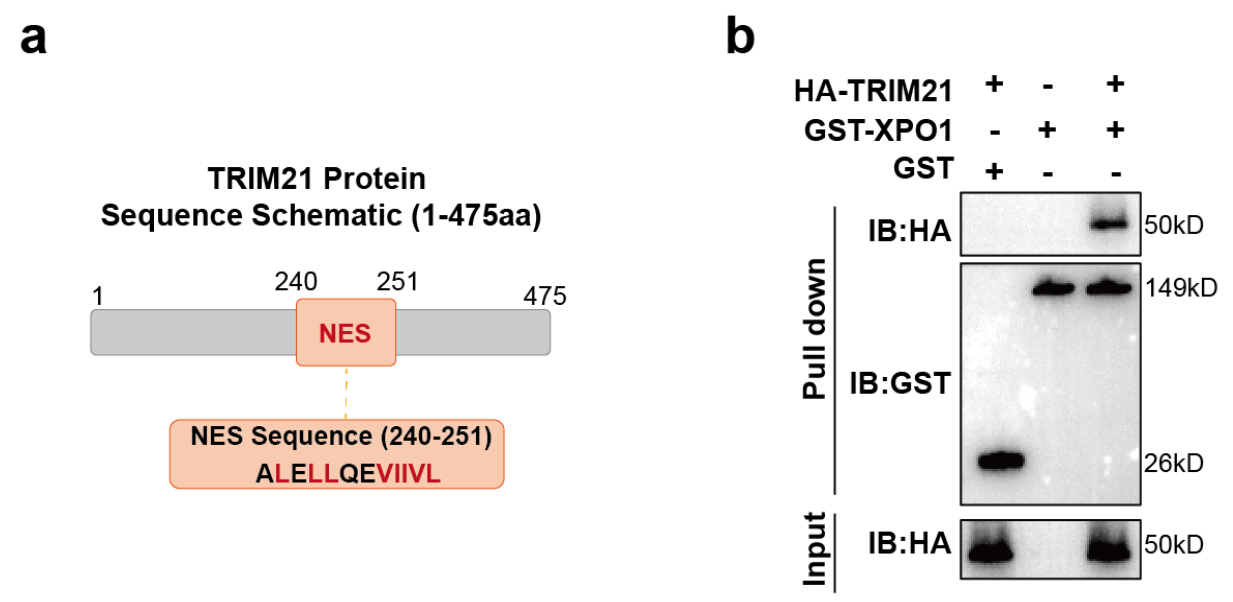

Fig. R19. a Schematic representation of a predicted leucine-rich nuclear export signal (NES) in TRIM21 at amino acids 240–251. The putative NES sequence, ALELLQEVIIVL, is shown, with hydrophobic residues highlighted in red. **b** In vitro GST pull-down using recombinant GST-XPO1 and HA-TRIM21 proteins to verify the direct interaction between XPO1 and TRIM21. Data are representative of n = 3 biologically independent experiments. Source data are provided as a Source Data file.

Comment 9: Explicitly discuss any sample bias with the SCLC clinical samples and how this might impact generalizability of conclusions.

a. The authors could validate the association between XPO1/TREM2/class I in independent public datasets. This would add strength to this manuscript.

Response: We thank the reviewer for this insightful suggestion. We agree that potential sample bias in the clinical SCLC cohort and the generalizability of the observed associations should be explicitly discussed. To address this point, we have both clarified this limitation in the revised Discussion and performed additional validation analyses in independent public SCLC transcriptomic datasets.

We acknowledge that the clinical specimens used in our study may be subject to several sources of bias, including differences in molecular subtype composition, tumour purity, immune-cell infiltration, and prior treatment history. Given the marked heterogeneity of SCLC, these factors may influence the measured expression of XPO1, TREM2, TNFSF15, and MHC class I genes, and therefore may affect the generalizability of correlation-based findings derived from a single cohort.

To strengthen the manuscript, we interrogated independent public SCLC bulk RNA-seq datasets. In these analyses, we observed a significant inverse correlation between XPO1 and TNFSF15 expression in GSE40275 (Fig. R20a), and a significant inverse correlation between XPO1 and HLA-A, HLA-B, and HLA-C expression in the George et al. Dataset (Fig. R20b). These findings are consistent with our proposed model that XPO1 negatively regulates TNFSF15 expression and is associated with reduced antigen-presentation-related programs in SCLC.

By contrast, we did not observe a robust correlation between XPO1 and TREM2 in the available bulk RNA-seq datasets. We interpret this discrepancy cautiously. Because TREM2 is primarily expressed in tumour-associated myeloid cells rather than tumour cells, bulk RNA-seq may not adequately capture this relationship, particularly in cohorts with variable immune-cell content or tumour purity. In addition, differences in sequencing depth, preprocessing pipelines, and sample composition across studies may further obscure context-dependent associations involving microenvironmental cell states.

Overall, these public-dataset analyses support the generalizability of the XPO1–TNFSF15 and XPO1–MHC class I associations, while suggesting that the XPO1–TREM2 relationship may be more dependent on tumour microenvironment context and therefore less readily resolved in bulk tumour datasets. We have revised the manuscript accordingly to explicitly discuss these issues as an important limitation of the current clinical correlative analyses.

Changes in the manuscript: The revised content has been added to page 8 lines 3-5, page 12 lines 5-6, and page 17 lines 20-22.

"Additionally, analysis of the GSE60052 dataset revealed an inverse correlation between XPO1 and TNFSF15 expression ($r = -0.46$, $p < 0.001$), further supporting a negative regulatory relationship (Supplementary Fig. 5b)."

"Analysis of the George et al. dataset showed that XPO1 expression was inversely correlated with HLA-A ($r = -0.38$, $p < 0.001$), HLA-B ($r = -0.33$, $p = 0.003$), and HLA-C ($r = -0.33$, $p = 0.002$) (Supplementary Fig.11a)."

"Given the marked heterogeneity of SCLC, potential bias arising from cohort size, subtype composition, and prior treatment history may influence the observed associations and limit the generalizability of conclusions drawn from a single clinical cohort."

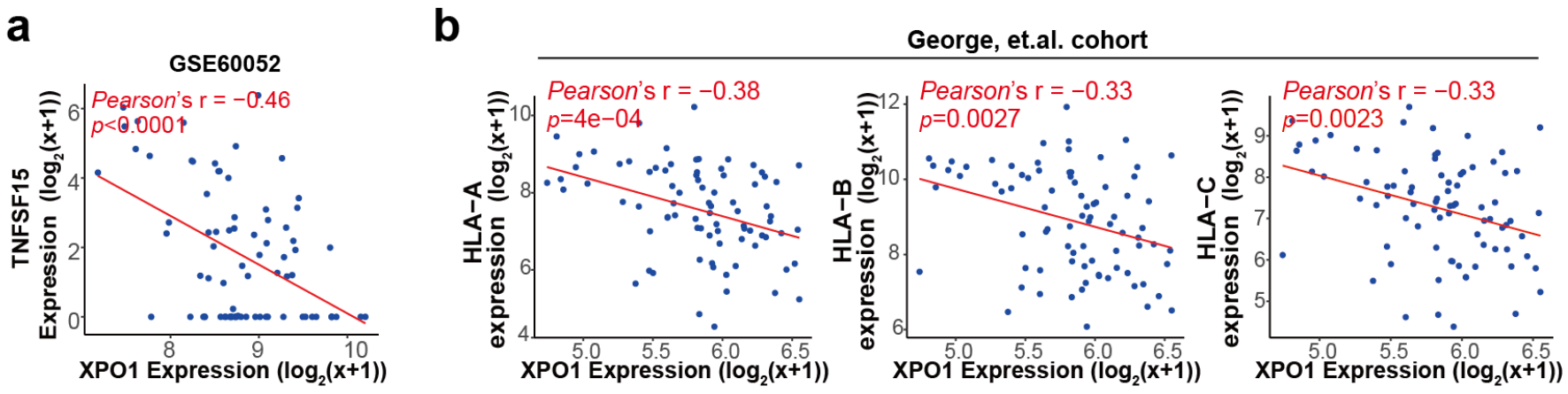

Fig.R20. a Pearson correlation analysis between XPO1 and TNFSF15 mRNA expression in the GSE40275 dataset (log2(x + 1), n = 79 samples). **b** Pearson correlation analysis between XPO1 and HLA-A, HLA-B, and HLA-C mRNA expression in the George et al. dataset (log2(x + 1), n = 81 samples). The correlation coefficient is shown for each gene pair. Data were analysed using Pearson correlation analysis. Source data are provided as a Source Data file.

Comment 10: To ensure statistical robustness, please report exact n (biological replicates) for all experiments, tests done, if any, for normality, and state whether data met test assumptions, In addition, use appropriate multiple testing correction (FDR) for high-dimensional data and report exact p-values, confidence intervals, and when appropriate, effect sizes.

Response: We thank the reviewer for this important comment and fully agree that clear and rigorous statistical reporting is essential to ensure robustness and reproducibility.

In response, we have systematically revised the manuscript to improve the transparency of statistical analyses throughout. Specifically, we have now: (1) explicitly reported the exact number of biological replicates (n) for all experiments in the figure legends and/or Methods; (2) specified the statistical tests used for each analysis, including whether tests were two-sided where applicable; (3) clarified our approach to assessing data distribution and selecting parametric versus nonparametric tests; (4) applied false discovery rate (FDR) correction for high-dimensional analyses, including transcriptomic and single-cell comparisons, where appropriate; and (5) added more complete statistical reporting in the revised figures, legends, source data, and supplementary tables, including exact P values where practical and appropriate.

For experiments involving small sample sizes, we have taken care to use statistical approaches appropriate to the data structure and distribution, and we now state these choices more explicitly in the revised manuscript. For high-dimensional datasets, we report adjusted significance values based on FDR correction. Where relevant and

feasible, we have also expanded the statistical information provided in the source data files to improve interpretability.

We believe these revisions substantially strengthen the statistical rigor and transparency of the study.

Changes in the manuscript: The revised content has been added to the figure legends or methods.

Other comments:

Comment 11: In Figure 1e, Y axis – is it SEM or SD

Response: Thank you for pointing this out. The Y-axis in Figure 1e represents standard deviation (SD), not the standard error of the mean (SEM). We have updated the figure legend to reflect this correction.

Comment 12: Extended Data Figure 2h: Y-axis relabel as "Proportion of non-tumor cells" instead of "Proportion in immune cells"

Response: We appreciate the suggestion. The Y-axis label in Extended Data Figure 2h has been corrected to "Proportion".

Comment 13: Supplementary Table 5 – correct huaman as human

Response: Thank you for catching this typo. We have corrected "huaman" to "human" in Supplementary data 5.

Comment 14: Two anti-human XPO1 listed from CST – Cat # 4302 should be corrected as anti-IRF3 instead anti-XPO1

Response: Thank you for pointing out this error. We have verified that CST Cat # 4302 is indeed an anti-IRF3 antibody, not an anti-XPO1 antibody. This has been corrected in the Supplementary data 6.

Comment 15: In Supplementary Methods file, line 26 "neutralizing antibody against human TNFSF15 (Cat. 78811, BPS Bioscience) is noted. However, BPS Biosciences Cat. 78811 is aTL1A repsonsive Jurkat reporter line. Please correct this error.

Response: We appreciate the reviewer's careful attention to detail. We have verified that BPS Bioscience Cat. 78811 is indeed a TL1A-responsive Jurkat reporter line and not an antibody against TNFSF15. We have corrected

the error and updated with “BPS Bioscience, Cat.101729” in the methods accordingly.

Comment 16: Fig 8: degration to degradation

Response: Thank you for catching this typographical error. We have corrected “degration” to “degradation” in Figure 8.

Reviewer #3 (Remarks to the Author):

Comment 1: In the current manuscript authors explore the role of XPO1 inhibition in SCLC models. The subcutaneous tumors shown in figure 1 are treated with Selinexor. It would be useful to have information on the dosage and administration of the drug at least in the figure legend. Does the cell line used express XPO1 as analyzed by WB or IHC?. Some of the results shown using scRNAseq should be validated using IHC to show differences in the infiltration by macrophages and their phenotype. In figure 1H the colors seem not to be coded correctly since the green observed in the Selinexor group is not included in the legend. In figure 1i the differences are hard to see, maybe different coding would be more useful.

Response:

1) Dosage and administration of Selinexor

We thank the reviewer for this important comment and agree that detailed information regarding Selinexor procurement, formulation, and dosing should be clearly provided for reproducibility. In the revised manuscript, we have now added the full treatment details to both the Methods section and the relevant figure legends. Specifically, Selinexor was purchased from MedChemExpress (HY-17536), formulated in corn oil, and administered by oral gavage at 5 mg/kg once weekly (QW).

2) Cell line XPO1 expression

We also appreciate the reviewer’s request to clarify the XPO1 expression status of the murine cell lines used for tumour modelling. To address this, we performed immunoblot analysis in the mouse cell lines Lewis/LLS, non-SMC, and RP116. These data showed that XPO1 is expressed in the murine SCLC cell lines used in our in vivo studies, including RP116, and that its expression is higher than in the non-small cell lung cancer line LLS (Fig. 21a).

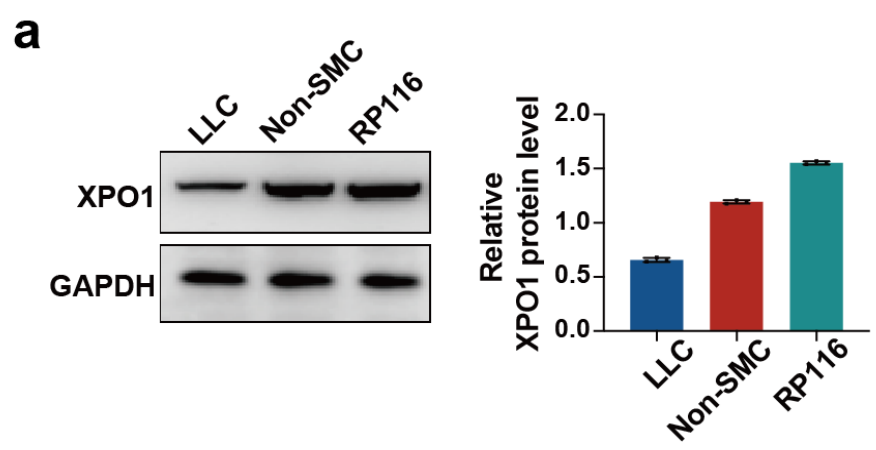

Fig. R21. a Endogenous XPO1 protein expression in Lewis/LLS, non-SMC, and RP116 cells, as determined by immunoblotting (left), with ImageJ-based quantification shown on the right. Data are from n = 3 biologically independent experiments. Data are presented as mean ± s.d. Source data are provided as a Source Data file.

3) Validation of macrophage infiltration and phenotype by IHC

We appreciate the reviewer's comment regarding the validation of macrophage infiltration and phenotype by IHC. In the revised manuscript, we have now added immunohistochemistry-based validation in the subcutaneous allograft tumours used for single-cell RNA-seq analysis. These additional IHC analyses confirmed a reduction in TREM2^{+} macrophages under XPO1 inhibition, consistent with the single-cell transcriptomic results (Fig. R22a).

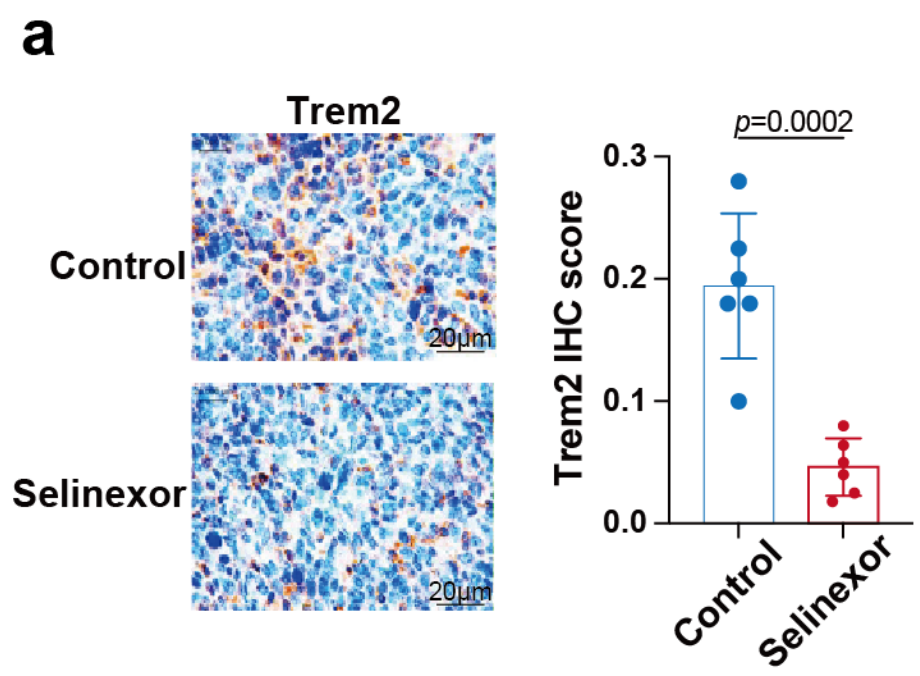

Fig. R22. a Representative immunohistochemistry images of Trem2 staining in SCLC allograft tumors with control and Selinexor treatment. Scale bars, 20 μm. Data was compared using a unpaired two-side t-test. n = 6 independent experiments per group. Data are presented as mean ± s.d. Source data are provided as a Source Data file.

4) Figure 1H and 1i visualization

We thank the reviewer for this insightful comment. We agree that the visualization in the original version of Fig. 1h and Fig. 1i was not sufficiently clear. In the revised manuscript, we updated the presentation of Fig. 1h and i to improve interpretability. Specifically, rather than relying only on the original t-SNE-based display, we expanded the scRNA-seq cohort from 3 to 6 tumours per group and added a dot plot summarizing the normalized expression of representative canonical neuroendocrine and non-neuroendocrine lineage markers (Fig. R4a), together with a boxplot comparing neuroendocrine (NE) signature scores between tumour cells from control and Selinexor-treated

tumours (Fig. R4b).

In the expanded dataset, tumour cells from the Selinexor-treated group showed reduced expression of several neuroendocrine-associated genes, including Chga, Bex1, and Syp, compared with controls (Fig. R4a). However, the overall neuroendocrine score was not significantly decreased (Fig. R4b). Thus, although Selinexor treatment appears to attenuate expression of some neuroendocrine-associated genes, the current data do not support a firm conclusion that XPO1 inhibition induces a neuroendocrine-to-non-neuroendocrine lineage transition. One possible explanation is that the observed decrease in NE marker expression may reflect a broader treatment-related suppression of tumour cell state rather than a true lineage switch. Importantly, rather than supporting a broad shift in SCLC lineage identity, our data suggest that the dominant consequence of XPO1 inhibition in this setting is tumour-cell-intrinsic rewiring of immunoregulatory programs that secondarily reshape the surrounding immune microenvironment. Mechanistically, this is reflected in the selective depletion of a TREM2+ immunoregulatory macrophage population, which we traced to tumour-intrinsic regulation of the TRIM21–IRF3–TNFSF15 axis.

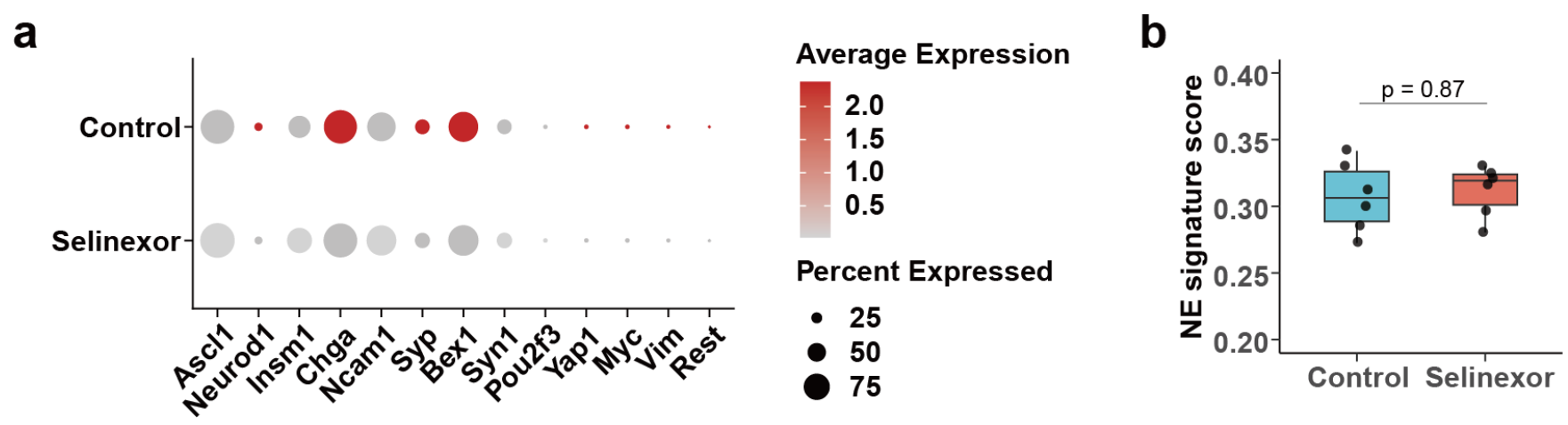

Fig. R4. a Dot plot showing the expression of canonical neuroendocrine and non-neuroendocrine lineage markers in tumour cells from control and Selinexor-treated SCLC allograft tumors. Dot size indicates the percentage of cells expressing each gene, and colour indicates the average expression level. Neuroendocrine-associated markers include Ascl1, Neurod1, Chga, Ncam1, Syp, Bex1, and Syt13, whereas canonical non-neuroendocrine markers include Pou2f3, Yap1, Myc, and Rest. **b** Comparison of neuroendocrine (NE) signature scores in tumour cells from control and Selinexor-treated SCLC allograft tumours. Signature scores were calculated using the UCell algorithm based on a curated set of NE-associated genes[1]. Data are from n = 6 mice per group. Source data are provided as a Source Data file.

Changes in manuscript:

The dosage and administration of Selinexor has been added to the figure legend of fig. 1f: **“f** Schematic of the in vivo experiment. RP116 murine SCLC cells (1 × 10^6) were injected subcutaneously into C57BL/6 mice. Mice were randomized into two groups and treated with Selinexor (5 mg/kg, p.o., once weekly) or vehicle control.”

We also added new immunoblot data showing endogenous XPO1 expression in the murine cell lines used for tumour modelling (Supplementary Fig. 1f).

To validate the single-cell findings at the tissue level, we included new immunohistochemistry data showing reduced Trem2^{+} macrophage infiltration in Selinexor-treated subcutaneous allograft tumours (Fig. 1o).

In addition, we expanded the scRNA-seq cohort from 3 to 6 tumours per group and revised the presentation of the tumour-cell state analysis by adding a dot plot of canonical neuroendocrine and non-neuroendocrine lineage markers together with a boxplot of neuroendocrine signature scores (Supplementary Fig. 1l,m). The revised content has been added to page 5 lines 9-12 and page 15 lines 5-18: "Within the tumour compartment, Selinexor treatment was associated with reduced expression of several NE-associated genes, including Chga, Syp, and Bex1 (Supplementary Fig. 1l); however, the overall NE program was not markedly altered (Supplementary Fig. 1m)."

"In our study, single-cell transcriptomic analysis showed that Selinexor treatment reduced the expression of several NE-associated genes, including Chga, Bex1, and Syp. However, the overall NE program remained unchanged, indicating that XPO1 inhibition does not drive a clear neuroendocrine-to-non-neuroendocrine lineage transition under the conditions examined here. Rather than supporting a broad shift in SCLC lineage identity, our data suggest that the dominant consequence of XPO1 inhibition in this setting is tumour-cell-intrinsic rewiring of immunoregulatory programs that secondarily reshape the surrounding immune microenvironment. Importantly, this does not exclude a role for XPO1 in SCLC lineage plasticity. It remains possible that XPO1 inhibition influences neuroendocrine cell-state transitions in a context-dependent manner, for example across distinct subtype backgrounds, treatment durations, or stages of tumour evolution. However, within the experimental framework used here, the more prominent and reproducible consequence of XPO1 inhibition was depletion of a TREM2^{+} immunoregulatory macrophage population, which we traced mechanistically to tumour-cell-intrinsic regulation of the TRIM21-IRF3-TNFSF15 axis. These findings therefore position XPO1 not as a regulator of tumour proliferation or lineage features, but as a tumour-cell-intrinsic driver of immunosuppressive niche formation in SCLC."

Comment 2: In figure 2 (or figure legend) add information on the co-culture system, showing the length of the experiment. The difference in growth in the nude mice caused by XPO-1 KO or overexpression is shown only in

day 28 of the experiment. Being the bioluminescence system designed to allow the longitudinal analysis of tumoral growth, authors should show the complete data and how the different tumors grow over time. Also, to better evaluate the infiltration by macrophages in the orthotopic mouse model, information on the infiltration of the macrophages by IHC should be added to figure 2.

Authors mention that treating mice with Selinexor had no observable toxicity, which is in contrast to other reports, and clinical data, with Selinexor inducing weight loss. How are the doses of Selinexor and anti-PD-1 chosen in this setting? In the survival experiment, it would be interesting to see the changes in the tumoral microenvironment by IHC when mice succumb to the tumors. The survival of humanized mice using human PBMCs has been described to be no longer than 8 weeks, here the animals live way longer, can the authors provide details on the mechanisms that avoid the GvHD effect? The confirmation of the results using syngeneic/immunocompetent mice models of SCLC would made the conclusions more solid.

Response: We thank the reviewer for these thoughtful and constructive comments. We agree that additional experimental detail and in vivo validation improve the clarity, rigor, and translational relevance of the study. We have addressed each point as follows.

1) Length of the co-culture experiment

We agree that the duration of the co-culture experiment should be explicitly stated. We have now clarified in the figure legend that PMA-differentiated THP-1-derived macrophages or PBMC-derived macrophages were co-cultured with the indicated SCLC cells for 24 h (Fig. R23a).

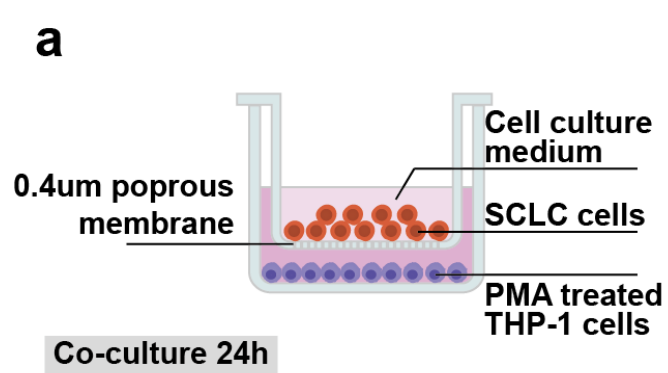

Fig. R23. a Schematic of the in vitro co-culture system in which PMA-differentiated THP-1-derived or PBMC-derived macrophages were cultured with the indicated SCLC cells for 24 h.

2) Longitudinal monitoring of orthotopic tumour growth

We appreciate the reviewer's suggestion to provide the full longitudinal bioluminescence data rather than only the endpoint measurement. In the revised manuscript, we now include representative bioluminescence images together with longitudinal quantification of total flux over time for all experimental groups (Fig. R24a-d). These data more clearly show tumour growth dynamics in the orthotopic model and allow better assessment of the

impact of XPO1 knockdown or overexpression on tumour progression.

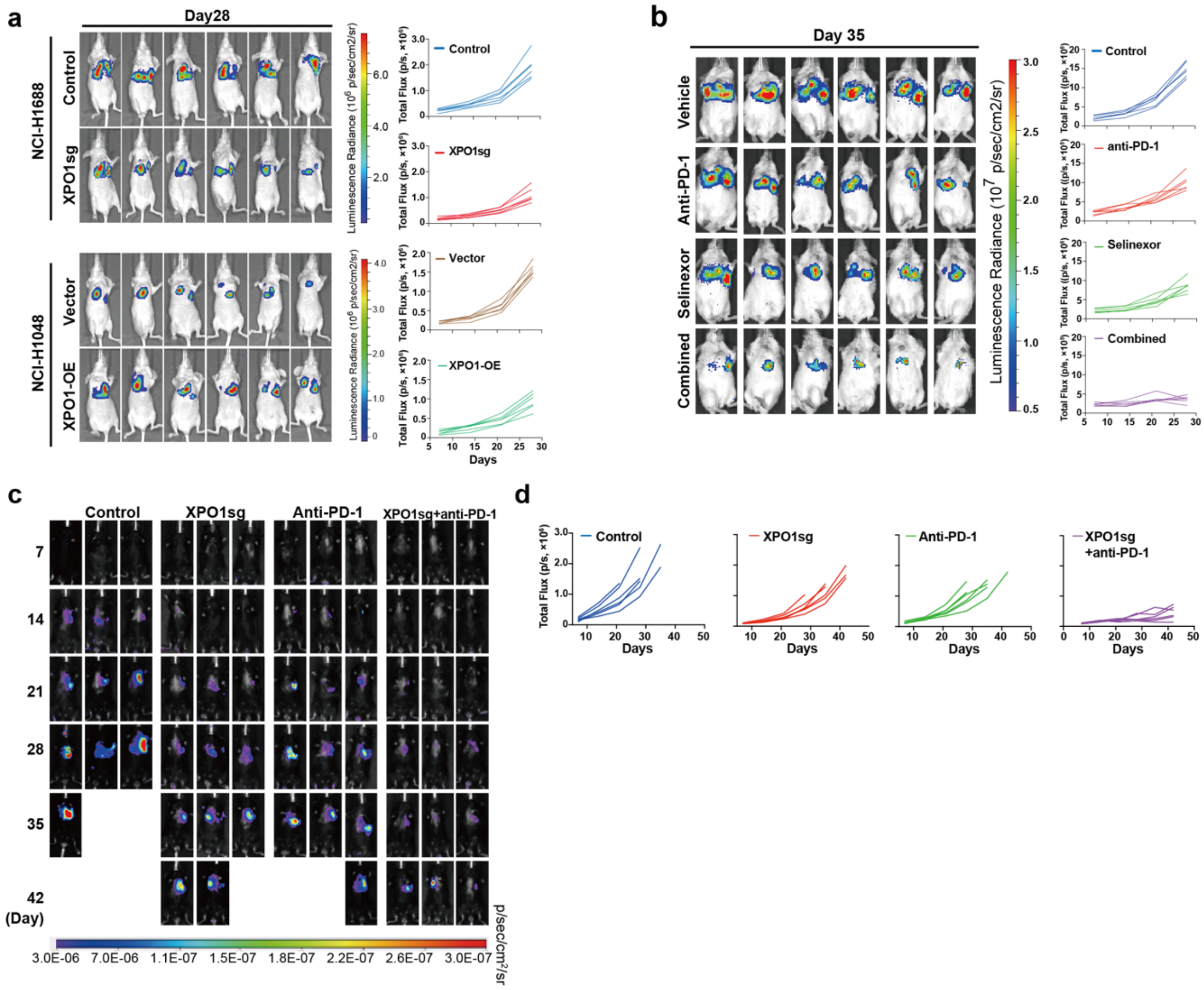

Fig. R24. a Orthotopic xenograft growth of luciferase-labelled indicated SCLC cells (1×10^6) in BALB/c nude mice. Representative bioluminescence images (BLI) acquired on day 28 (left), longitudinal measurements of bioluminescence intensity (middle), and quantification of signal intensity at day 28 (right) are shown. **b** Human peripheral blood mononuclear cells (PBMCs) were injected into NOG-MHC I/II-2 KO mice, followed by intrapulmonary injection of 1×10^6 luciferase-expressing NCI-H1688 cells to establish tumors. Mice were then treated with Selinexor (5 mg/kg, p.o., once weekly), anti-PD-1 antibody (200 μg/injection, i.p., every 3 days), or both. Representative BLI from the indicated treatment groups at the experimental endpoint are shown. **c,d** Mice bearing orthotopic xenografts of RP116-luc (1×10^6) cells transduced with XPO1-silencing or vector-control lentivirus were treated by intraperitoneal injection with anti-PD-1 antibody (200 μg/injection, i.p., q3d) or isotype control antibody. Representative BLI acquired from day 7 to day 42 (**a**) and longitudinal quantification of bioluminescence signal intensity (**b**) are shown. Data are from n = 6 mice per group. Source data are provided as a Source Data file.

3) IHC validation of macrophage infiltration and phenotype in orthotopic tumours

We agree that histologic validation would strengthen the interpretation of the tumour microenvironment changes observed by flow cytometry. In the original manuscript, we quantified TREM2$^+$ and CD86$^+$ macrophage populations by flow cytometry using suspension of tumors obtained by tissue digestion. In the revised manuscript, we additionally performed IHC on orthotopic tumours. These analyses showed that XPO1 knockdown reduced TREM2$^+$ macrophages and increased CD86$^+$ macrophages, whereas XPO1 overexpression produced the opposite trend (Fig. R25a,b). These IHC data are consistent with the flow cytometry results and support XPO1-dependent macrophage-state remodeling in vivo.

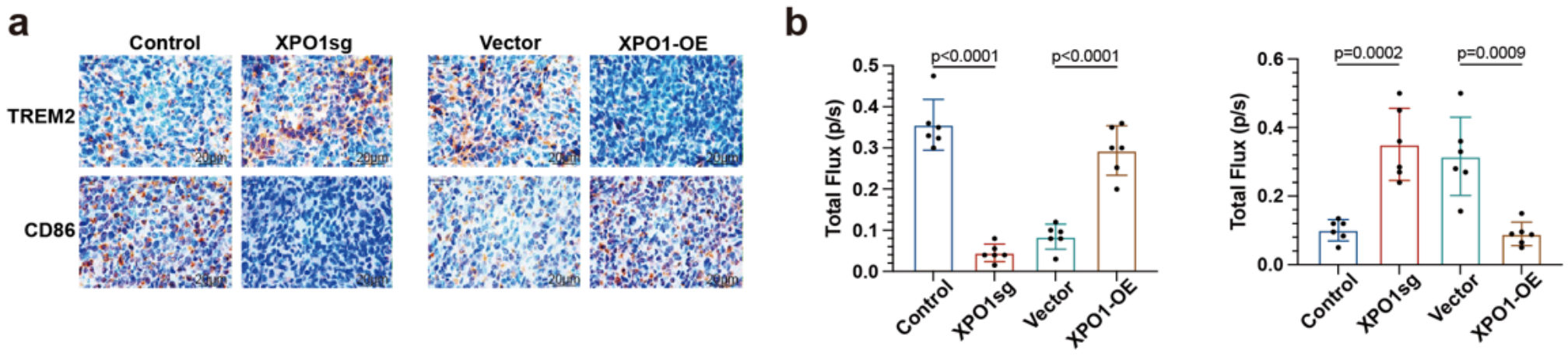

Fig. R25. a Mice bearing orthotopic xenografts derived from NCI-H1688-luc cells transduced with XPO1 sgRNA or control lentivirus, or from NCI-H1048-luc cells transduced with XPO1 or vector lentivirus. Representative immunohistochemistry images of TREM2 and CD86 staining in xenograft SCLC tissues from the indicated groups at the experimental endpoint. Scale bar, 20 μm. **b** Quantification of the staining shown in panel **a**. Data was compared using an unpaired two-side t-test. n = 6 independent experiments per group. Data are presented as mean ± s.d. Source data are provided as a Source Data file.

4) Selinexor toxicity and dose selection

We thank the reviewer for raising this important point. Selinexor was purchased from MedChemExpress, formulated for oral administration, and delivered by oral gavage at 5 mg/kg once weekly. This regimen was selected based on pilot tolerability observations in our models together with prior in vivo studies in SCLC. Previous studies have reported that Selinexor was tolerated in SCLC xenograft models at 15 mg/kg i.p. once weekly in combination with BMN673, and at 7.5 mg/kg i.p. once weekly in combination with vorinostat, while a 10 mg/kg oral regimen given for 3 consecutive days was also reported to be effective and well tolerated in other SCLC models.

In our preliminary experiments, however, higher oral dosing was associated with reduced tolerability in a subset of mice, including body-weight loss. We therefore selected 5 mg/kg once weekly for the current study, as this regimen maintained acceptable tolerability while preserving measurable antitumour activity. Body weight remained within an acceptable range throughout treatment, with losses of less than 10% (Supplementary Fig. 12n).

In addition, anti-PD-1 antibody was administered intraperitoneally at 200 μg per injection every 3 days on the basis of prior preclinical studies. We have now clarified these dosing details in the Methods and corresponding figure legends.

5) Prolonged survival of PBMC-humanized mice and avoidance of GvHD

We agree that the unexpectedly prolonged survival of PBMC-humanized mice requires clarification. In our study, this extended survival window is attributable to the use of MHC class I/II double-knockout (MHC-dKO) immunodeficient mice rather than conventional NSG/NOG strains. Because these mice lack murine MHC class I and class II molecules, xenoreactive recognition by human T cells is markedly reduced, resulting in attenuated xeno-GvHD. Consistent with previous reports, this strategy can extend the survival of PBMC-humanized mice to approximately 15 weeks[14,15]. Consistent with this, the median survival of mice in our study was 58.5 days (range, 32–112 days) (Supplementary Fig. 12l). This extended survival window enabled longitudinal assessment of tumour–immune interactions in vivo.

6) Validation in syngeneic/immunocompetent SCLC models

We fully agree that confirmation in syngeneic, immunocompetent models would strengthen the conclusions. In response, we have now added experiments using a murine syngeneic SCLC model. Specifically, we tested XPO1 silencing in RP116-luc tumours together with anti-PD-1 treatment in immunocompetent mice. The results were consistent with our findings in the humanized model, including reduced tumour burden, prolonged survival, and changes in the tumour microenvironment, thereby strengthening the generalizability of our conclusions (Fig. 26a-e).

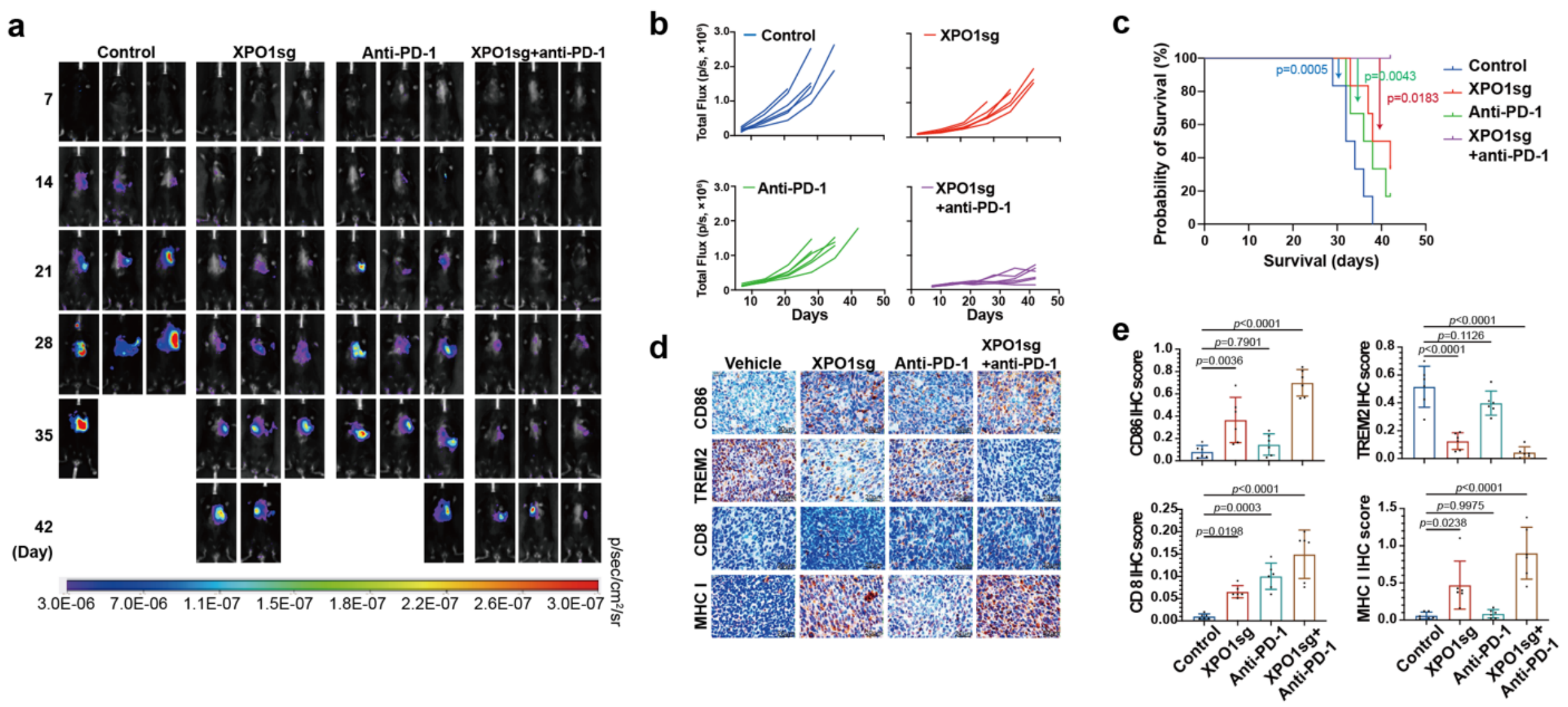

Fig. 26. a,b Mice bearing orthotopic xenografts of RP116-luc cells transduced with XPO1-silencing or vector-control lentivirus were treated by intraperitoneal injection with anti-PD-1 antibody (200 µg/injection, i.p., q3d) or isotype control antibody. Representative bioluminescence images acquired from day 7 to day 42 (a) and longitudinal quantification of bioluminescence signal intensity (**b**) are shown. **c** Kaplan–Meier analysis of survival in mice from the indicated treatment groups. **d** Representative immunohistochemistry images of TREM2, CD86, CD8, and MHC I staining in xenograft SCLC tissues from the indicated groups. Scale bar, 20 µm. **e** Quantification of the staining shown in **d**. Survival curves in c were compared using the two-sided log-rank test. Statistical significance in **e** was assessed by one-way ANOVA. Data are from n = 6 mice per group. Source data are provided as a Source Data file.

Changes in the manuscript: We revised the figure legends for Fig. 2a to specify the 24 h co-culture duration and now include longitudinal orthotopic BLI measurements over time (Fig. 2e, Fig. 7a,b, and Supplementary Fig. 12j). We added IHC validation of macrophage phenotypes in orthotopic tumours (Supplementary Fig.4l,m). We expanded the Methods to clarify Selinexor and anti-PD-1 dosing, route, and schedule. Finally, we incorporated new syngeneic/immunocompetent mouse data showing that XPO1 loss sensitizes SCLC to anti-PD-1 therapy and reshapes the tumour immune microenvironment in vivo (Fig. 7a-e).

The revised content has been added to page 20 lines 5-8, page 13 lines 29-31, and page 14 lines 1-8.

"For treatment, Selinexor (MedChemExpress, HY-17536) was formulated in corn oil and administered by oral gavage at 5 mg/kg once weekly (QW). Anti-PD-1 antibody (Bio X Cell, BE0146) was administered intraperitoneally at 200 µg/injection every 3 days. Treatment was initiated on day 7 after implantation and continued until the experimental endpoint."

"To determine whether the effects of pharmacologic XPO1 inhibition could be recapitulated by genetic XPO1 suppression in an immunocompetent setting, we established an orthotopic RP116 lung tumor model in C57BL/6 mice using control or XPO1-knockdown cells, followed by treatment with anti-PD-1 antibody (Fig. 7a). Longitudinal bioluminescence imaging showed that XPO1 knockdown significantly enhanced the antitumor effect of PD-1 blockade, resulting in a greater reduction in tumor burden than either XPO1 knockdown alone or anti-PD-1 treatment alone (Fig. 7b). Consistent with this finding, mice in the combination group showed the most favorable survival outcome among all groups (Fig. 7c). Immunohistochemical analysis of endpoint tumors further revealed decreased TREM2^{+} macrophage infiltration, increased CD86^{+} macrophage infiltration, enhanced CD8^{+}

T-cell infiltration, and elevated MHC class I expression in the combination group (Fig. 7d,e). Together, these results provide in vivo evidence that XPO1 contributes to shaping the immune microenvironment of SCLC and suggest that XPO1 targeting may improve the therapeutic response to PD-1 blockade."

Minor

Comment 3: Please label the figures including figure number.

Response: Sincerely thank you for your suggestion. All figures now include correct labels with the figure number and have been updated accordingly.

Reference:

1 Ireland, A. S. *et al.* MYC Drives Temporal Evolution of Small Cell Lung Cancer Subtypes by Reprogramming Neuroendocrine Fate. *Cancer Cell* **38**, 60-78 e12 (2020). https://doi.org/10.1016/j.ccell.2020.05.001

2 Fang, Y. *et al.* TEAD3 + high-risk melanoma cells crosstalk with GAS6 + macrophages via the GAS6-TYRO3 ligand-receptor axis to modulate propionate metabolism and drive melanoma progression. *J Exp Clin Cancer Res* **44**, 279 (2025). https://doi.org/10.1186/s13046-025-03542-0

3 Wang, Y., Qiu, F. & Yan, J. Transcription factor c-Maf-targeted cancer immunotherapy. *Trends Cancer* **9**, 265-269 (2023). https://doi.org/10.1016/j.trecan.2022.12.002

4 Pauli, J. *et al.* Single cell spatial transcriptomics integration deciphers the morphological heterogeneity of atherosclerotic carotid arteries. *Nat Commun* **16**, 11282 (2025). https://doi.org/10.1038/s41467-025-67679-4

5 Widdess, M. A. *et al.* Harnessing multivalency and FcgammaRIIB engagement to augment anti-CD27 immunotherapy. *Nat Commun* **17**, 1122 (2025). https://doi.org/10.1038/s41467-025-67882-3

6 Azizi, E. *et al.* Single-Cell Map of Diverse Immune Phenotypes in the Breast Tumor Microenvironment. *Cell* **174**, 1293-1308 e1236 (2018). https://doi.org/10.1016/j.cell.2018.05.060

7 Bentsen, M. *et al.* ATAC-seq footprinting unravels kinetics of transcription factor binding during zygotic genome activation. *Nat Commun* **11**, 4267 (2020). https://doi.org/10.1038/s41467-020-18035-1

8 Zhao, C. C. *et al.* TNFSF15 facilitates differentiation and polarization of macrophages toward M1 phenotype to inhibit tumor growth. *Oncoimmunology* **11**, 2032918 (2022). https://doi.org/10.1080/2162402X.2022.2032918

9 Zhu, Y. P. *et al.* TNFSF15 alleviates myeloid-derived suppressor cell-mediated cancer immunosuppression in mice. *Acta Pharmacol Sin* **47**, 493-503 (2026). https://doi.org/10.1038/s41401-025-01663-y

10 Wang, J. *et al.* XPO1 inhibition synergizes with PARP1 inhibition in small cell lung cancer by targeting nuclear transport of FOXO3a. *Cancer Lett* **503**, 197-212 (2021). https://doi.org/10.1016/j.canlet.2021.01.008

11 Qin, T. *et al.* Nuclear to Cytoplasmic Transport Is a Druggable Dependency in HDAC7-driven Small Cell Lung Cancer. *Adv Sci (Weinh)* **12**, e2413445 (2025). https://doi.org/10.1002/advs.202413445

12 Quintanal-Villalonga, A. *et al.* Inhibition of XPO1 Sensitizes Small Cell Lung Cancer to First- and Second-Line Chemotherapy. *Cancer Res* **82**, 472-483 (2022). https://doi.org/10.1158/0008-5472.CAN-21-2964

13 Quintanal-Villalonga, A. *et al.* Exportin 1 inhibition prevents neuroendocrine transformation through SOX2 down-regulation in lung and prostate cancers. *Sci Transl Med* **15**, eadf7006 (2023). https://doi.org/10.1126/scitranslmed.adf7006

14 Eguren-Santamaria, I. *et al.* MHC class I and II-deficient humanized mice are suitable tools to test the long-term antitumor efficacy of immune checkpoint inhibitors and T-cell engagers. *J Immunother Cancer* **12** (2024). https://doi.org/10.1136/jitc-2023-008516

15 Brehm, M. A. *et al.* Lack of acute xenogeneic graft- versus-host disease, but retention of T-cell function following engraftment of human peripheral blood mononuclear cells in NSG mice deficient in MHC class I and II expression. *FASEB J* **33**, 3137-3151 (2019). https://doi.org/10.1096/fj.201800636R

Dear Editors and Reviewers:

We sincerely thank you and the Reviewers for the careful and constructive evaluation of our manuscript, entitled "**Targeting TRIM21 nuclear export reprograms TREM2^{+} macrophage polarization to augment anti-PD-1 efficacy in small cell lung cancer**." We are grateful for the opportunity to revise our work.

In this point-by-point response letter, the original comments from the review are presented in black, our responses are highlighted in blue, and the corresponding changes marked in the manuscript are indicated in red.

Point-by-point responses to Reviewers' comments

Reviews comments:

Reviewer #1: Commending the authors for their exhaustive efforts in revising this manuscript. It is clear that significant time and energy were devoted to addressing the reviewers' feedback. The inclusion of critical new data, most notably the second in-vivo replicate and the syngeneic model addresses previous gaps in the study's validation. This high level of responsiveness has resulted in a much more impactful and scientifically sound paper.

Response: We sincerely thank the reviewer for these encouraging comments and for recognizing the efforts made during the revision process. We greatly appreciate the reviewer's careful evaluation and constructive feedback. These additions have substantially strengthened the scientific rigor and translational relevance of the study.

Reviewer #2: The authors have successfully addressed all of my comments. The article has been significantly strengthened. I recommend the article for publication.

Response: We sincerely thank the reviewer for these encouraging comments and for recognizing the efforts made during the revision process. We greatly appreciate the reviewer's careful evaluation and constructive feedback. These additions have substantially strengthened the scientific rigor and translational relevance of the study.

Reviewer #3: I appreciate the changes and additional experiments performed by the authors. However, further changes and additional data would be desirable for clarity.

Comment 1: The expression of XPO1 by WB in the cell lines should be tested in an experiment including a

positive control.

Response: We thank the reviewer for this important suggestion. To address this concern, we repeated the immunoblot analysis of XPO1 expression in the murine cell lines used for tumor modelling and included a positive control lysate in the same experiment. Specifically, NIH/3T3 cells, which are known to express XPO1 and are recommended as a positive control for the anti-XPO1 antibody, were included on the same blot together with Lewis cells, which served as a low-expression comparator, non-SMC cells, another murine SCLC cell line, and RP116 cells. The NIH/3T3 positive control showed a clear XPO1 band at the expected molecular weight, confirming the validity of the immunoblotting assay. Consistent with our previous results, XPO1 expression was detected in the murine SCLC cell line RP116 used in our in vivo studies. Densitometric quantification normalized to GAPDH has also been added.

Changes in the manuscript: We added NIH/3T3 cells as a positive control in the XPO1 immunoblot analysis, and the updated data are now shown in Supplementary Fig. 1f and described in the revised manuscript.

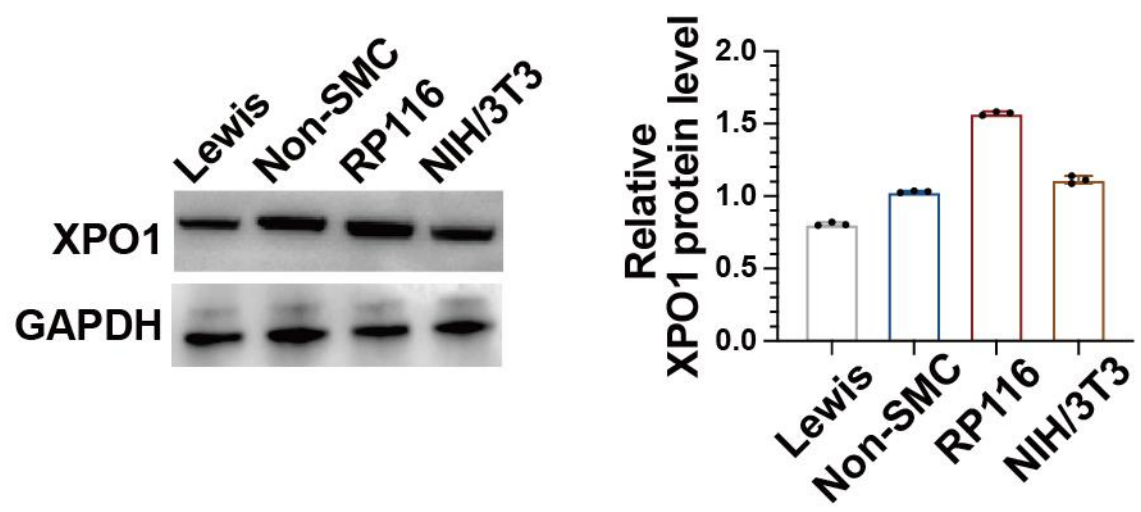

Fig. R1: Endogenous XPO1 protein expression in Lewis, non-SMC, and RP116 cells, with NIH/3T3 cells included as a positive control, as determined by immunoblotting. ImageJ-based densitometric quantification of XPO1 normalized to GAPDH is shown on the right.

Comment 2: All IHC analysis should include a pan-macrophage marker and images showing different amplifications.

Response: We thank the reviewer for this helpful suggestion. We agree that macrophage-related IHC analyses should be interpreted in the context of total macrophage infiltration. In the revised manuscript, we have added the pan-macrophage marker CD68 to both human SCLC specimens and mouse tumor tissues to provide a macrophage reference for the interpretation of TREM2- and CD86-related staining. We further quantified CD68 staining scores across the indicated groups and found no significant differences in overall CD68-positive macrophage infiltration. These results support our conclusion that the observed changes in TREM2- or CD86-positive

macrophage populations mainly reflect alterations in macrophage phenotype/polarization rather than changes in total macrophage abundance. In addition, representative IHC images are now provided at both low and high magnifications, with scale bars, to show the overall spatial distribution as well as cellular-level staining patterns.

Changes in the manuscript: We added CD68 staining, a pan-macrophage marker, to Fig. 1o, Fig. 7d, Supplementary Fig. S4i, and Supplementary Fig. 12p. Representative IHC images are now provided at both low and high magnifications with scale bars.

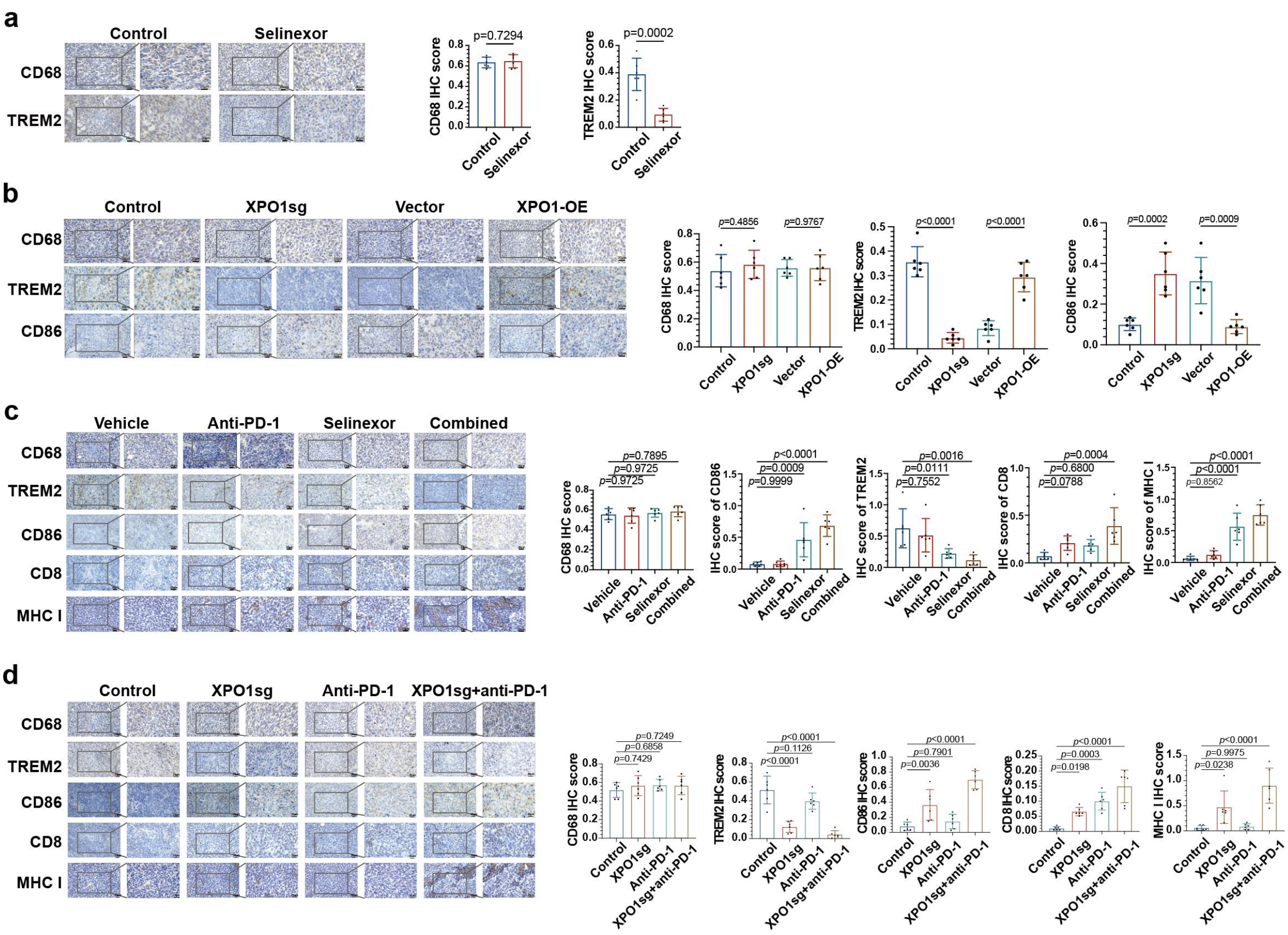

Fig. R2: a Representative immunohistochemistry images of CD68 and TREM2 staining in SCLC allograft tumours from control and Selinexor-treated mice (left). Scale bars, 20 µm. Quantification of the staining (right). **b** Mice bearing orthotopic xenografts derived from NCI-H1688-luc cells transduced with XPO1 sgRNA or control lentivirus, or from NCI-H1048-luc cells transduced with XPO1 or vector lentivirus. Representative immunohistochemistry images of CD68, TREM2, and CD86 staining in xenograft SCLC tissues from the indicated groups at the experimental endpoint (left). Scale bar, 20 µm. Quantification of the staining (right). **c** Representative immunohistochemistry images of CD68, TREM2, CD86, CD8, and MHC I staining in xenograft SCLC tissues from the indicated treatment groups (left). Scale bars, 20 µm. Quantification of the staining (right). **d** Representative immunohistochemistry images of CD68, TREM2, CD86, CD8, and MHC I staining in allograft

SCLC tissues from the indicated groups (left). Scale bars, 20 μm. Quantification of the staining (right).

Comment 3: Regarding the addition of longitudinal bioluminiscence data, I appreciate the new data showed. However, the results would be easier to interpret if the different groups compared were included in the same plot and appropriate statistical test applied. If no significant differences are observed, it should be stated in the main text.

Response: We thank the reviewer for this helpful suggestion. In the revised manuscript, we have reorganized the longitudinal bioluminescence imaging data so that the groups being compared are presented in the same plot, allowing a more direct visual comparison of tumor growth kinetics. For experiments in which all mice remained available at the indicated imaging time points, appropriate statistical analyses were applied to compare longitudinal tumor burden among groups, and the results are now indicated in the revised figures, figure legends, and main text where applicable.

For the XPO1sg plus anti-PD-1 experiment, several mice died during the observation period. As a result, the number of mice available for bioluminescence imaging differed among groups at later time points. Because this missingness was related to disease progression rather than random loss, direct group-wise comparison of bioluminescence signals at later uniform time points would be statistically inappropriate and could introduce bias by comparing only the surviving animals. Therefore, we present the longitudinal bioluminescence curves for this experiment descriptively and evaluate the overall therapeutic effect using Kaplan-Meier survival analysis with the log-rank test. This point has now been clarified in the revised main text and figure legend.

Finally, we sincerely thank the reviewer for these encouraging comments and for recognizing the efforts made during the revision process. We greatly appreciate the reviewer's careful evaluation and constructive feedback. These additions have substantially strengthened the scientific rigor and translational relevance of the study.

Changes in the manuscript: The longitudinal bioluminescence imaging data for the different comparison groups have been reorganized into the same plots in Fig. 2f, Fig. 7b, and Supplementary Fig. 12k.

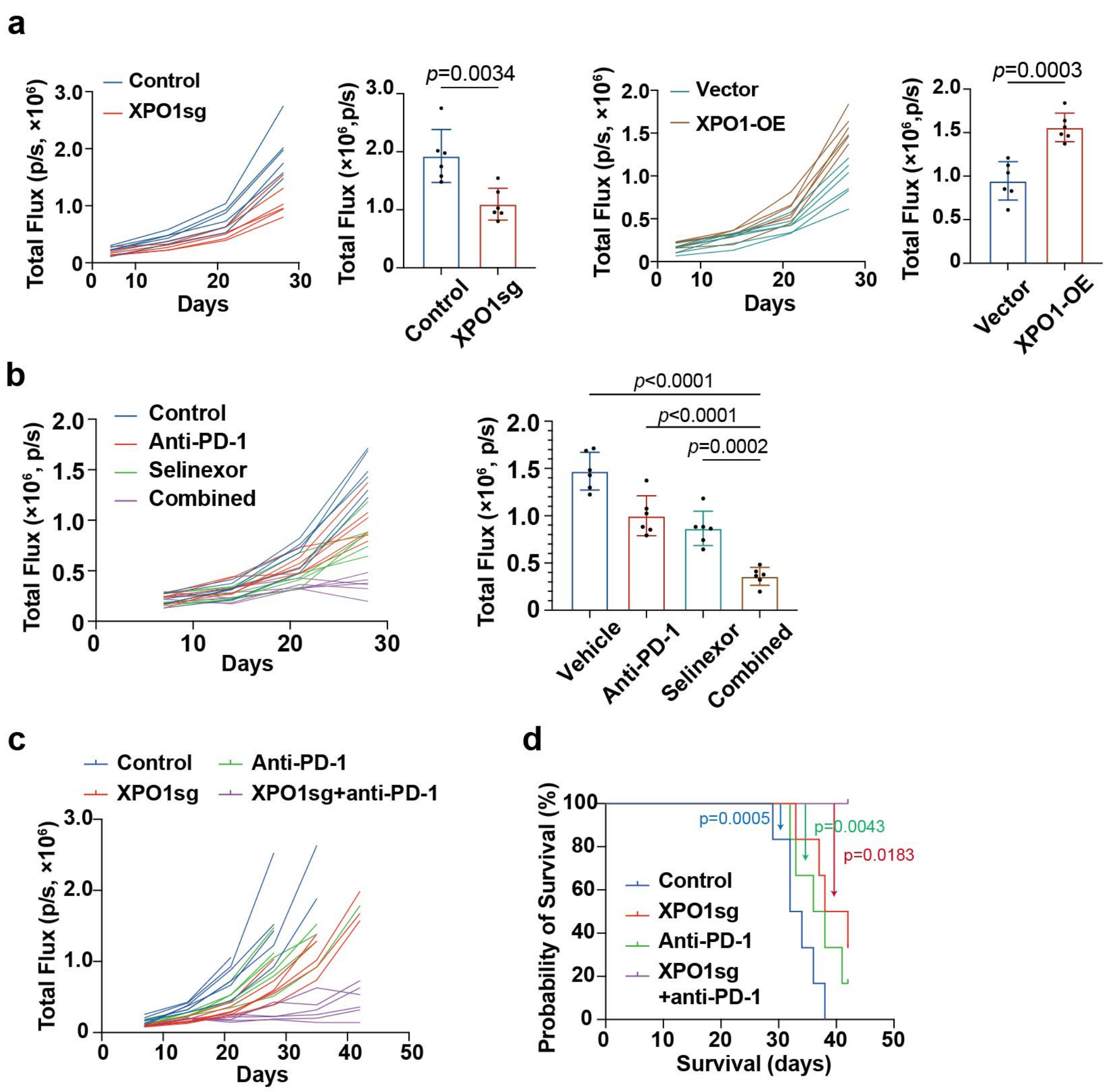

Fig. R3: **a** Orthotopic xenograft growth of luciferase-labelled indicated SCLC cells (1 × 10^6) in BALB/c nude mice. Longitudinal bioluminescence imaging (BLI) quantification of tumor burden in mice. Individual lines represent BLI signal intensity from individual mice over time. Right, quantification of BLI signal intensity at day 28. **b** Human peripheral blood mononuclear cells (PBMCs) were injected into NOG-MHC I/II-2 KO mice, followed by intrapulmonary injection of 1 × 10^6 luciferase-expressing NCI-H1688 cells to establish tumors. Mice were then treated with Selinexor (5 mg/kg, p.o., once weekly), anti-PD-1 antibody (200 μg/injection, i.p., every 3 days), or both. Longitudinal quantification of bioluminescence imaging (BLI) signal intensity in mice over time. Right, quantification of tumour burden based on BLI signal intensity at day 28. **c** Mice bearing orthotopic allografts of RP116-luc cells transduced with XPO1-silencing or vector-control lentivirus were treated by intraperitoneal injection with anti-PD-1 antibody (200 μg/injection, i.p., q3d) or isotype control antibody. Longitudinal quantification of bioluminescence signal intensity are shown. **d** Kaplan-Meier analysis of survival in mice from the indicated groups.
