# Supplementary material for: XPO1-mediated TRIM21 nuclear export reprograms TREM2+ macrophage polarization by targeting IRF3 to augment anti-PD-1 efficacy in small cell lung cancer
Source: Nat Commun. 2026 Aug 22;17:10050. doi: 10.1038/s41467-026-76862-0 (PMC13594258; doi:10.1038/s41467-026-76862-0)
Supplement: Supplementary file 3 [file 41467_2026_76862_MOESM3_ESM.pdf]

Description of Additional Supplementary Files

Supplementary Data 1. Proteins identified as XPO1-interacting candidates by immunoprecipitation–mass spectrometry.
This file contains the detailed results of the XPO1 immunoprecipitation-mass spectrometry analysis, including the proteins identified as candidate XPO1-interacting partners and the associated mass spectrometry information.

Supplementary Data 2. Differentially expressed genes identified by RNA sequencing following XPO1 knockdown in NCI-H1688 and NCI-H196 SCLC cells.
This file contains the differentially expressed genes identified by RNA sequencing in control and XPO1-knockdown NCI-H1688 and NCI-H196 SCLC cells, together with the corresponding differential expression statistics.

Supplementary Data 3. Transcription factor motif enrichment and TOBIAS footprinting-based differential binding analyses of ATAC-seq data from control and XPO1-knockdown NCI-H1688 cells.
This file contains transcription factor motif enrichment results and TOBIAS footprinting-based differential binding analyses derived from ATAC-seq data comparing control and XPO1-knockdown NCI-H1688 cells.
